# Patterns of somatic structural variation in human cancer genomes

**DOI:** 10.1038/s41586-019-1913-9

**Published:** 2020-02-05

**Authors:** Yilong Li, Nicola D. Roberts, Jeremiah A. Wala, Ofer Shapira, Steven E. Schumacher, Kiran Kumar, Ekta Khurana, Sebastian Waszak, Jan O. Korbel, James E. Haber, Marcin Imielinski, PCAWG Structural Variation Working Group, Kadir C. Akdemir, Eva G. Alvarez, Adrian Baez-Ortega, Rameen Beroukhim, Paul C. Boutros, David D. L. Bowtell, Benedikt Brors, Kathleen H. Burns, Peter J. Campbell, Kin Chan, Ken Chen, Isidro Cortés-Ciriano, Ana Dueso-Barroso, Andrew J. Dunford, Paul A. Edwards, Xavier Estivill, Dariush Etemadmoghadam, Lars Feuerbach, J. Lynn Fink, Milana Frenkel-Morgenstern, Dale W. Garsed, Mark Gerstein, Dmitry A. Gordenin, David Haan, James E. Haber, Julian M. Hess, Barbara Hutter, Marcin Imielinski, David T. W. Jones, Young Seok Ju, Marat D. Kazanov, Leszek J. Klimczak, Youngil Koh, Jan O. Korbel, Kiran Kumar, Eunjung Alice Lee, Jake June-Koo Lee, Yilong Li, Andy G. Lynch, Geoff Macintyre, Florian Markowetz, Iñigo Martincorena, Alexander Martinez-Fundichely, Matthew Meyerson, Satoru Miyano, Hidewaki Nakagawa, Fabio C. P. Navarro, Stephan Ossowski, Peter J. Park, John V. Pearson, Montserrat Puiggròs, Karsten Rippe, Nicola D. Roberts, Steven A. Roberts, Bernardo Rodriguez-Martin, Steven E. Schumacher, Ralph Scully, Mark Shackleton, Nikos Sidiropoulos, Lina Sieverling, Chip Stewart, David Torrents, Jose M. C. Tubio, Izar Villasante, Nicola Waddell, Jeremiah A. Wala, Joachim Weischenfeldt, Lixing Yang, Xiaotong Yao, Sung-Soo Yoon, Jorge Zamora, Cheng-Zhong Zhang, Joachim Weischenfeldt, Rameen Beroukhim, Peter J. Campbell, PCAWG Consortium, Lauri A. Aaltonen, Federico Abascal, Adam Abeshouse, Hiroyuki Aburatani, David J. Adams, Nishant Agrawal, Keun Soo Ahn, Sung-Min Ahn, Hiroshi Aikata, Rehan Akbani, Kadir C. Akdemir, Hikmat Al-Ahmadie, Sultan T. Al-Sedairy, Fatima Al-Shahrour, Malik Alawi, Monique Albert, Kenneth Aldape, Ludmil B. Alexandrov, Adrian Ally, Kathryn Alsop, Eva G. Alvarez, Fernanda Amary, Samirkumar B. Amin, Brice Aminou, Ole Ammerpohl, Matthew J. Anderson, Yeng Ang, Davide Antonello, Pavana Anur, Samuel Aparicio, Elizabeth L. Appelbaum, Yasuhito Arai, Axel Aretz, Koji Arihiro, Shun-ichi Ariizumi, Joshua Armenia, Laurent Arnould, Sylvia Asa, Yassen Assenov, Gurnit Atwal, Sietse Aukema, J. Todd Auman, Miriam R. R. Aure, Philip Awadalla, Marta Aymerich, Gary D. Bader, Adrian Baez-Ortega, Matthew H. Bailey, Peter J. Bailey, Miruna Balasundaram, Saianand Balu, Pratiti Bandopadhayay, Rosamonde E. Banks, Stefano Barbi, Andrew P. Barbour, Jonathan Barenboim, Jill Barnholtz-Sloan, Hugh Barr, Elisabet Barrera, John Bartlett, Javier Bartolome, Claudio Bassi, Oliver F. Bathe, Daniel Baumhoer, Prashant Bavi, Stephen B. Baylin, Wojciech Bazant, Duncan Beardsmore, Timothy A. Beck, Sam Behjati, Andreas Behren, Beifang Niu, Cindy Bell, Sergi Beltran, Christopher Benz, Andrew Berchuck, Anke K. Bergmann, Erik N. Bergstrom, Benjamin P. Berman, Daniel M. Berney, Stephan H. Bernhart, Rameen Beroukhim, Mario Berrios, Samantha Bersani, Johanna Bertl, Miguel Betancourt, Vinayak Bhandari, Shriram G. Bhosle, Andrew V. Biankin, Matthias Bieg, Darell Bigner, Hans Binder, Ewan Birney, Michael Birrer, Nidhan K. Biswas, Bodil Bjerkehagen, Tom Bodenheimer, Lori Boice, Giada Bonizzato, Johann S. De Bono, Arnoud Boot, Moiz S. Bootwalla, Ake Borg, Arndt Borkhardt, Keith A. Boroevich, Ivan Borozan, Christoph Borst, Marcus Bosenberg, Mattia Bosio, Jacqueline Boultwood, Guillaume Bourque, Paul C. Boutros, G. Steven Bova, David T. Bowen, Reanne Bowlby, David D. L. Bowtell, Sandrine Boyault, Rich Boyce, Jeffrey Boyd, Alvis Brazma, Paul Brennan, Daniel S. Brewer, Arie B. Brinkman, Robert G. Bristow, Russell R. Broaddus, Jane E. Brock, Malcolm Brock, Annegien Broeks, Angela N. Brooks, Denise Brooks, Benedikt Brors, Søren Brunak, Timothy J. C. Bruxner, Alicia L. Bruzos, Alex Buchanan, Ivo Buchhalter, Christiane Buchholz, Susan Bullman, Hazel Burke, Birgit Burkhardt, Kathleen H. Burns, John Busanovich, Carlos D. Bustamante, Adam P. Butler, Atul J. Butte, Niall J. Byrne, Anne-Lise Børresen-Dale, Samantha J. Caesar-Johnson, Andy Cafferkey, Declan Cahill, Claudia Calabrese, Carlos Caldas, Fabien Calvo, Niedzica Camacho, Peter J. Campbell, Elias Campo, Cinzia Cantù, Shaolong Cao, Thomas E. Carey, Joana Carlevaro-Fita, Rebecca Carlsen, Ivana Cataldo, Mario Cazzola, Jonathan Cebon, Robert Cerfolio, Dianne E. Chadwick, Dimple Chakravarty, Don Chalmers, Calvin Wing Yiu Chan, Kin Chan, Michelle Chan-Seng-Yue, Vishal S. Chandan, David K. Chang, Stephen J. Chanock, Lorraine A. Chantrill, Aurélien Chateigner, Nilanjan Chatterjee, Kazuaki Chayama, Hsiao-Wei Chen, Jieming Chen, Ken Chen, Yiwen Chen, Zhaohong Chen, Andrew D. Cherniack, Jeremy Chien, Yoke-Eng Chiew, Suet-Feung Chin, Juok Cho, Sunghoon Cho, Jung Kyoon Choi, Wan Choi, Christine Chomienne, Zechen Chong, Su Pin Choo, Angela Chou, Angelika N. Christ, Elizabeth L. Christie, Eric Chuah, Carrie Cibulskis, Kristian Cibulskis, Sara Cingarlini, Peter Clapham, Alexander Claviez, Sean Cleary, Nicole Cloonan, Marek Cmero, Colin C. Collins, Ashton A. Connor, Susanna L. Cooke, Colin S. Cooper, Leslie Cope, Vincenzo Corbo, Matthew G. Cordes, Stephen M. Cordner, Isidro Cortés-Ciriano, Kyle Covington, Prue A. Cowin, Brian Craft, David Craft, Chad J. Creighton, Yupeng Cun, Erin Curley, Ioana Cutcutache, Karolina Czajka, Bogdan Czerniak, Rebecca A. Dagg, Ludmila Danilova, Maria Vittoria Davi, Natalie R. Davidson, Helen Davies, Ian J. Davis, Brandi N. Davis-Dusenbery, Kevin J. Dawson, Francisco M. De La Vega, Ricardo De Paoli-Iseppi, Timothy Defreitas, Angelo P. Dei Tos, Olivier Delaneau, John A. Demchok, Jonas Demeulemeester, German M. Demidov, Deniz Demircioğlu, Nening M. Dennis, Robert E. Denroche, Stefan C. Dentro, Nikita Desai, Vikram Deshpande, Amit G. Deshwar, Christine Desmedt, Jordi Deu-Pons, Noreen Dhalla, Neesha C. Dhani, Priyanka Dhingra, Rajiv Dhir, Anthony DiBiase, Klev Diamanti, Li Ding, Shuai Ding, Huy Q. Dinh, Luc Dirix, HarshaVardhan Doddapaneni, Nilgun Donmez, Michelle T. Dow, Ronny Drapkin, Oliver Drechsel, Ruben M. Drews, Serge Serge, Tim Dudderidge, Ana Dueso-Barroso, Andrew J. Dunford, Michael Dunn, Lewis Jonathan Dursi, Fraser R. Duthie, Ken Dutton-Regester, Jenna Eagles, Douglas F. Easton, Stuart Edmonds, Paul A. Edwards, Sandra E. Edwards, Rosalind A. Eeles, Anna Ehinger, Juergen Eils, Roland Eils, Adel El-Naggar, Matthew Eldridge, Kyle Ellrott, Serap Erkek, Georgia Escaramis, Shadrielle M. G. Espiritu, Xavier Estivill, Dariush Etemadmoghadam, Jorunn E. Eyfjord, Bishoy M. Faltas, Daiming Fan, Yu Fan, William C. Faquin, Claudiu Farcas, Matteo Fassan, Aquila Fatima, Francesco Favero, Nodirjon Fayzullaev, Ina Felau, Sian Fereday, Martin L. Ferguson, Vincent Ferretti, Lars Feuerbach, Matthew A. Field, J. Lynn Fink, Gaetano Finocchiaro, Cyril Fisher, Matthew W. Fittall, Anna Fitzgerald, Rebecca C. Fitzgerald, Adrienne M. Flanagan, Neil E. Fleshner, Paul Flicek, John A. Foekens, Kwun M. Fong, Nuno A. Fonseca, Christopher S. Foster, Natalie S. Fox, Michael Fraser, Scott Frazer, Milana Frenkel-Morgenstern, William Friedman, Joan Frigola, Catrina C. Fronick, Akihiro Fujimoto, Masashi Fujita, Masashi Fukayama, Lucinda A. Fulton, Robert S. Fulton, Mayuko Furuta, P. Andrew Futreal, Anja Füllgrabe, Stacey B. Gabriel, Steven Gallinger, Carlo Gambacorti-Passerini, Jianjiong Gao, Shengjie Gao, Levi Garraway, Øystein Garred, Erik Garrison, Dale W. Garsed, Nils Gehlenborg, Josep L. L. Gelpi, Joshy George, Daniela S. Gerhard, Clarissa Gerhauser, Jeffrey E. Gershenwald, Mark Gerstein, Moritz Gerstung, Gad Getz, Mohammed Ghori, Ronald Ghossein, Nasra H. Giama, Richard A. Gibbs, Bob Gibson, Anthony J. Gill, Pelvender Gill, Dilip D. Giri, Dominik Glodzik, Vincent J. Gnanapragasam, Maria Elisabeth Goebler, Mary J. Goldman, Carmen Gomez, Santiago Gonzalez, Abel Gonzalez-Perez, Dmitry A. Gordenin, James Gossage, Kunihito Gotoh, Ramaswamy Govindan, Dorthe Grabau, Janet S. Graham, Robert C. Grant, Anthony R. Green, Eric Green, Liliana Greger, Nicola Grehan, Sonia Grimaldi, Sean M. Grimmond, Robert L. Grossman, Adam Grundhoff, Gunes Gundem, Qianyun Guo, Manaswi Gupta, Shailja Gupta, Ivo G. Gut, Marta Gut, Jonathan Göke, Gavin Ha, Andrea Haake, David Haan, Siegfried Haas, Kerstin Haase, James E. Haber, Nina Habermann, Faraz Hach, Syed Haider, Natsuko Hama, Freddie C. Hamdy, Anne Hamilton, Mark P. Hamilton, Leng Han, George B. Hanna, Martin Hansmann, Nicholas J. Haradhvala, Olivier Harismendy, Ivon Harliwong, Arif O. Harmanci, Eoghan Harrington, Takanori Hasegawa, David Haussler, Steve Hawkins, Shinya Hayami, Shuto Hayashi, D. Neil Hayes, Stephen J. Hayes, Nicholas K. Hayward, Steven Hazell, Yao He, Allison P. Heath, Simon C. Heath, David Hedley, Apurva M. Hegde, David I. Heiman, Michael C. Heinold, Zachary Heins, Lawrence E. Heisler, Eva Hellstrom-Lindberg, Mohamed Helmy, Seong Gu Heo, Austin J. Hepperla, José María Heredia-Genestar, Carl Herrmann, Peter Hersey, Julian M. Hess, Holmfridur Hilmarsdottir, Jonathan Hinton, Satoshi Hirano, Nobuyoshi Hiraoka, Katherine A. Hoadley, Asger Hobolth, Ermin Hodzic, Jessica I. Hoell, Steve Hoffmann, Oliver Hofmann, Andrea Holbrook, Aliaksei Z. Holik, Michael A. Hollingsworth, Oliver Holmes, Robert A. Holt, Chen Hong, Eun Pyo Hong, Jongwhi H. Hong, Gerrit K. Hooijer, Henrik Hornshøj, Fumie Hosoda, Yong Hou, Volker Hovestadt, William Howat, Alan P. Hoyle, Ralph H. Hruban, Jianhong Hu, Taobo Hu, Xing Hua, Kuan-lin Huang, Mei Huang, Mi Ni Huang, Vincent Huang, Yi Huang, Wolfgang Huber, Thomas J. Hudson, Michael Hummel, Jillian A. Hung, David Huntsman, Ted R. Hupp, Jason Huse, Matthew R. Huska, Barbara Hutter, Carolyn M. Hutter, Daniel Hübschmann, Christine A. Iacobuzio-Donahue, Charles David Imbusch, Marcin Imielinski, Seiya Imoto, William B. Isaacs, Keren Isaev, Shumpei Ishikawa, Murat Iskar, S. M. Ashiqul Islam, Michael Ittmann, Sinisa Ivkovic, Jose M. G. Izarzugaza, Jocelyne Jacquemier, Valerie Jakrot, Nigel B. Jamieson, Gun Ho Jang, Se Jin Jang, Joy C. Jayaseelan, Reyka Jayasinghe, Stuart R. Jefferys, Karine Jegalian, Jennifer L. Jennings, Seung-Hyup Jeon, Lara Jerman, Yuan Ji, Wei Jiao, Peter A. Johansson, Amber L. Johns, Jeremy Johns, Rory Johnson, Todd A. Johnson, Clemency Jolly, Yann Joly, Jon G. Jonasson, Corbin D. Jones, David R. Jones, David T. W. Jones, Nic Jones, Steven J. M. Jones, Jos Jonkers, Young Seok Ju, Hartmut Juhl, Jongsun Jung, Malene Juul, Randi Istrup Juul, Sissel Juul, Natalie Jäger, Rolf Kabbe, Andre Kahles, Abdullah Kahraman, Vera B. Kaiser, Hojabr Kakavand, Sangeetha Kalimuthu, Christof von Kalle, Koo Jeong Kang, Katalin Karaszi, Beth Karlan, Rosa Karlić, Dennis Karsch, Katayoon Kasaian, Karin S. Kassahn, Hitoshi Katai, Mamoru Kato, Hiroto Katoh, Yoshiiku Kawakami, Jonathan D. Kay, Stephen H. Kazakoff, Marat D. Kazanov, Maria Keays, Electron Kebebew, Richard F. Kefford, Manolis Kellis, James G. Kench, Catherine J. Kennedy, Jules N. A. Kerssemakers, David Khoo, Vincent Khoo, Narong Khuntikeo, Ekta Khurana, Helena Kilpinen, Hark Kyun Kim, Hyung-Lae Kim, Hyung-Yong Kim, Hyunghwan Kim, Jaegil Kim, Jihoon Kim, Jong K. Kim, Youngwook Kim, Tari A. King, Wolfram Klapper, Kortine Kleinheinz, Leszek J. Klimczak, Stian Knappskog, Michael Kneba, Bartha M. Knoppers, Youngil Koh, Jan Komorowski, Daisuke Komura, Mitsuhiro Komura, Gu Kong, Marcel Kool, Jan O. Korbel, Viktoriya Korchina, Andrey Korshunov, Michael Koscher, Roelof Koster, Zsofia Kote-Jarai, Antonios Koures, Milena Kovacevic, Barbara Kremeyer, Helene Kretzmer, Markus Kreuz, Savitri Krishnamurthy, Dieter Kube, Kiran Kumar, Pardeep Kumar, Sushant Kumar, Yogesh Kumar, Ritika Kundra, Kirsten Kübler, Ralf Küppers, Jesper Lagergren, Phillip H. Lai, Peter W. Laird, Sunil R. Lakhani, Christopher M. Lalansingh, Emilie Lalonde, Fabien C. Lamaze, Adam Lambert, Eric Lander, Pablo Landgraf, Luca Landoni, Anita Langerød, Andrés Lanzós, Denis Larsimont, Erik Larsson, Mark Lathrop, Loretta M. S. Lau, Chris Lawerenz, Rita T. Lawlor, Michael S. Lawrence, Alexander J. Lazar, Ana Mijalkovic Lazic, Xuan Le, Darlene Lee, Donghoon Lee, Eunjung Alice Lee, Hee Jin Lee, Jake June-Koo Lee, Jeong-Yeon Lee, Juhee Lee, Ming Ta Michael Lee, Henry Lee-Six, Kjong-Van Lehmann, Hans Lehrach, Dido Lenze, Conrad R. Leonard, Daniel A. Leongamornlert, Ignaty Leshchiner, Louis Letourneau, Ivica Letunic, Douglas A. Levine, Lora Lewis, Tim Ley, Chang Li, Constance H. Li, Haiyan Irene Li, Jun Li, Lin Li, Shantao Li, Siliang Li, Xiaobo Li, Xiaotong Li, Xinyue Li, Yilong Li, Han Liang, Sheng-Ben Liang, Peter Lichter, Pei Lin, Ziao Lin, W. M. Linehan, Ole Christian Lingjærde, Dongbing Liu, Eric Minwei Liu, Fei-Fei Fei Liu, Fenglin Liu, Jia Liu, Xingmin Liu, Julie Livingstone, Dimitri Livitz, Naomi Livni, Lucas Lochovsky, Markus Loeffler, Georgina V. Long, Armando Lopez-Guillermo, Shaoke Lou, David N. Louis, Laurence B. Lovat, Yiling Lu, Yong-Jie Lu, Youyong Lu, Claudio Luchini, Ilinca Lungu, Xuemei Luo, Hayley J. Luxton, Andy G. Lynch, Lisa Lype, Cristina López, Carlos López-Otín, Eric Z. Ma, Yussanne Ma, Gaetan MacGrogan, Shona MacRae, Geoff Macintyre, Tobias Madsen, Kazuhiro Maejima, Andrea Mafficini, Dennis T. Maglinte, Arindam Maitra, Partha P. Majumder, Luca Malcovati, Salem Malikic, Giuseppe Malleo, Graham J. Mann, Luisa Mantovani-Löffler, Kathleen Marchal, Giovanni Marchegiani, Elaine R. Mardis, Adam A. Margolin, Maximillian G. Marin, Florian Markowetz, Julia Markowski, Jeffrey Marks, Tomas Marques-Bonet, Marco A. Marra, Luke Marsden, John W. M. Martens, Sancha Martin, Jose I. Martin-Subero, Iñigo Martincorena, Alexander Martinez-Fundichely, Yosef E. Maruvka, R. Jay Mashl, Charlie E. Massie, Thomas J. Matthew, Lucy Matthews, Erik Mayer, Simon Mayes, Michael Mayo, Faridah Mbabaali, Karen McCune, Ultan McDermott, Patrick D. McGillivray, Michael D. McLellan, John D. McPherson, John R. McPherson, Treasa A. McPherson, Samuel R. Meier, Alice Meng, Shaowu Meng, Andrew Menzies, Neil D. Merrett, Sue Merson, Matthew Meyerson, William Meyerson, Piotr A. Mieczkowski, George L. Mihaiescu, Sanja Mijalkovic, Tom Mikkelsen, Michele Milella, Linda Mileshkin, Christopher A. Miller, David K. Miller, Jessica K. Miller, Gordon B. Mills, Ana Milovanovic, Sarah Minner, Marco Miotto, Gisela Mir Arnau, Lisa Mirabello, Chris Mitchell, Thomas J. Mitchell, Satoru Miyano, Naoki Miyoshi, Shinichi Mizuno, Fruzsina Molnár-Gábor, Malcolm J. Moore, Richard A. Moore, Sandro Morganella, Quaid D. Morris, Carl Morrison, Lisle E. Mose, Catherine D. Moser, Ferran Muiños, Loris Mularoni, Andrew J. Mungall, Karen Mungall, Elizabeth A. Musgrove, Ville Mustonen, David Mutch, Francesc Muyas, Donna M. Muzny, Alfonso Muñoz, Jerome Myers, Ola Myklebost, Peter Möller, Genta Nagae, Adnan M. Nagrial, Hardeep K. Nahal-Bose, Hitoshi Nakagama, Hidewaki Nakagawa, Hiromi Nakamura, Toru Nakamura, Kaoru Nakano, Tannistha Nandi, Jyoti Nangalia, Mia Nastic, Arcadi Navarro, Fabio C. P. Navarro, David E. Neal, Gerd Nettekoven, Felicity Newell, Steven J. Newhouse, Yulia Newton, Alvin Wei Tian Ng, Anthony Ng, Jonathan Nicholson, David Nicol, Yongzhan Nie, G. Petur Nielsen, Morten Muhlig Nielsen, Serena Nik-Zainal, Michael S. Noble, Katia Nones, Paul A. Northcott, Faiyaz Notta, Brian D. O’Connor, Peter O’Donnell, Maria O’Donovan, Sarah O’Meara, Brian Patrick O’Neill, J. Robert O’Neill, David Ocana, Angelica Ochoa, Layla Oesper, Christopher Ogden, Hideki Ohdan, Kazuhiro Ohi, Lucila Ohno-Machado, Karin A. Oien, Akinyemi I. Ojesina, Hidenori Ojima, Takuji Okusaka, Larsson Omberg, Choon Kiat Ong, Stephan Ossowski, German Ott, B. F. Francis Ouellette, Christine P’ng, Marta Paczkowska, Salvatore Paiella, Chawalit Pairojkul, Marina Pajic, Qiang Pan-Hammarström, Elli Papaemmanuil, Irene Papatheodorou, Nagarajan Paramasivam, Ji Wan Park, Joong-Won Park, Keunchil Park, Kiejung Park, Peter J. Park, Joel S. Parker, Simon L. Parsons, Harvey Pass, Danielle Pasternack, Alessandro Pastore, Ann-Marie Patch, Iris Pauporté, Antonio Pea, John V. Pearson, Chandra Sekhar Pedamallu, Jakob Skou Pedersen, Paolo Pederzoli, Martin Peifer, Nathan A. Pennell, Charles M. Perou, Marc D. Perry, Gloria M. Petersen, Myron Peto, Nicholas Petrelli, Robert Petryszak, Stefan M. Pfister, Mark Phillips, Oriol Pich, Hilda A. Pickett, Todd D. Pihl, Nischalan Pillay, Sarah Pinder, Mark Pinese, Andreia V. Pinho, Esa Pitkänen, Xavier Pivot, Elena Piñeiro-Yáñez, Laura Planko, Christoph Plass, Paz Polak, Tirso Pons, Irinel Popescu, Olga Potapova, Aparna Prasad, Shaun R. Preston, Manuel Prinz, Antonia L. Pritchard, Stephenie D. Prokopec, Elena Provenzano, Xose S. Puente, Sonia Puig, Montserrat Puiggròs, Sergio Pulido-Tamayo, Gulietta M. Pupo, Colin A. Purdie, Michael C. Quinn, Raquel Rabionet, Janet S. Rader, Bernhard Radlwimmer, Petar Radovic, Benjamin Raeder, Keiran M. Raine, Manasa Ramakrishna, Kamna Ramakrishnan, Suresh Ramalingam, Benjamin J. Raphael, W. Kimryn Rathmell, Tobias Rausch, Guido Reifenberger, Jüri Reimand, Jorge Reis-Filho, Victor Reuter, Iker Reyes-Salazar, Matthew A. Reyna, Sheila M. Reynolds, Esther Rheinbay, Yasser Riazalhosseini, Andrea L. Richardson, Julia Richter, Matthew Ringel, Markus Ringnér, Yasushi Rino, Karsten Rippe, Jeffrey Roach, Lewis R. Roberts, Nicola D. Roberts, Steven A. Roberts, A. Gordon Robertson, Alan J. Robertson, Javier Bartolomé Rodriguez, Bernardo Rodriguez-Martin, F. Germán Rodríguez-González, Michael H. A. Roehrl, Marius Rohde, Hirofumi Rokutan, Gilles Romieu, Ilse Rooman, Tom Roques, Daniel Rosebrock, Mara Rosenberg, Philip C. Rosenstiel, Andreas Rosenwald, Edward W. Rowe, Romina Royo, Steven G. Rozen, Yulia Rubanova, Mark A. Rubin, Carlota Rubio-Perez, Vasilisa A. Rudneva, Borislav C. Rusev, Andrea Ruzzenente, Gunnar Rätsch, Radhakrishnan Sabarinathan, Veronica Y. Sabelnykova, Sara Sadeghi, S. Cenk Sahinalp, Natalie Saini, Mihoko Saito-Adachi, Gordon Saksena, Adriana Salcedo, Roberto Salgado, Leonidas Salichos, Richard Sallari, Charles Saller, Roberto Salvia, Michelle Sam, Jaswinder S. Samra, Francisco Sanchez-Vega, Chris Sander, Grant Sanders, Rajiv Sarin, Iman Sarrafi, Aya Sasaki-Oku, Torill Sauer, Guido Sauter, Robyn P. M. Saw, Maria Scardoni, Christopher J. Scarlett, Aldo Scarpa, Ghislaine Scelo, Dirk Schadendorf, Jacqueline E. Schein, Markus B. Schilhabel, Matthias Schlesner, Thorsten Schlomm, Heather K. Schmidt, Sarah-Jane Schramm, Stefan Schreiber, Nikolaus Schultz, Steven E. Schumacher, Roland F. Schwarz, Richard A. Scolyer, David Scott, Ralph Scully, Raja Seethala, Ayellet V. Segre, Iris Selander, Colin A. Semple, Yasin Senbabaoglu, Subhajit Sengupta, Elisabetta Sereni, Stefano Serra, Dennis C. Sgroi, Mark Shackleton, Nimish C. Shah, Sagedeh Shahabi, Catherine A. Shang, Ping Shang, Ofer Shapira, Troy Shelton, Ciyue Shen, Hui Shen, Rebecca Shepherd, Ruian Shi, Yan Shi, Yu-Jia Shiah, Tatsuhiro Shibata, Juliann Shih, Eigo Shimizu, Kiyo Shimizu, Seung Jun Shin, Yuichi Shiraishi, Tal Shmaya, Ilya Shmulevich, Solomon I. Shorser, Charles Short, Raunak Shrestha, Suyash S. Shringarpure, Craig Shriver, Shimin Shuai, Nikos Sidiropoulos, Reiner Siebert, Anieta M. Sieuwerts, Lina Sieverling, Sabina Signoretti, Katarzyna O. Sikora, Michele Simbolo, Ronald Simon, Janae V. Simons, Jared T. Simpson, Peter T. Simpson, Samuel Singer, Nasa Sinnott-Armstrong, Payal Sipahimalani, Tara J. Skelly, Marcel Smid, Jaclyn Smith, Karen Smith-McCune, Nicholas D. Socci, Heidi J. Sofia, Matthew G. Soloway, Lei Song, Anil K. Sood, Sharmila Sothi, Christos Sotiriou, Cameron M. Soulette, Paul N. Span, Paul T. Spellman, Nicola Sperandio, Andrew J. Spillane, Oliver Spiro, Jonathan Spring, Johan Staaf, Peter F. Stadler, Peter Staib, Stefan G. Stark, Lucy Stebbings, Ólafur Andri Stefánsson, Oliver Stegle, Lincoln D. Stein, Alasdair Stenhouse, Chip Stewart, Stephan Stilgenbauer, Miranda D. Stobbe, Michael R. Stratton, Jonathan R. Stretch, Adam J. Struck, Joshua M. Stuart, Henk G. Stunnenberg, Hong Su, Xiaoping Su, Ren X. Sun, Stephanie Sungalee, Hana Susak, Akihiro Suzuki, Fred Sweep, Monika Szczepanowski, Holger Sültmann, Takashi Yugawa, Angela Tam, David Tamborero, Benita Kiat Tee Tan, Donghui Tan, Patrick Tan, Hiroko Tanaka, Hirokazu Taniguchi, Tomas J. Tanskanen, Maxime Tarabichi, Roy Tarnuzzer, Patrick Tarpey, Morgan L. Taschuk, Kenji Tatsuno, Simon Tavaré, Darrin F. Taylor, Amaro Taylor-Weiner, Jon W. Teague, Bin Tean Teh, Varsha Tembe, Javier Temes, Kevin Thai, Sarah P. Thayer, Nina Thiessen, Gilles Thomas, Sarah Thomas, Alan Thompson, Alastair M. Thompson, John F. F. Thompson, R. Houston Thompson, Heather Thorne, Leigh B. Thorne, Adrian Thorogood, Grace Tiao, Nebojsa Tijanic, Lee E. Timms, Roberto Tirabosco, Marta Tojo, Stefania Tommasi, Christopher W. Toon, Umut H. Toprak, David Torrents, Giampaolo Tortora, Jörg Tost, Yasushi Totoki, David Townend, Nadia Traficante, Isabelle Treilleux, Jean-Rémi Trotta, Lorenz H. P. Trümper, Ming Tsao, Tatsuhiko Tsunoda, Jose M. C. Tubio, Olga Tucker, Richard Turkington, Daniel J. Turner, Andrew Tutt, Masaki Ueno, Naoto T. Ueno, Christopher Umbricht, Husen M. Umer, Timothy J. Underwood, Lara Urban, Tomoko Urushidate, Tetsuo Ushiku, Liis Uusküla-Reimand, Alfonso Valencia, David J. Van Den Berg, Steven Van Laere, Peter Van Loo, Erwin G. Van Meir, Gert G. Van den Eynden, Theodorus Van der Kwast, Naveen Vasudev, Miguel Vazquez, Ravikiran Vedururu, Umadevi Veluvolu, Shankar Vembu, Lieven P. C. Verbeke, Peter Vermeulen, Clare Verrill, Alain Viari, David Vicente, Caterina Vicentini, K. VijayRaghavan, Juris Viksna, Ricardo E. Vilain, Izar Villasante, Anne Vincent-Salomon, Tapio Visakorpi, Douglas Voet, Paresh Vyas, Ignacio Vázquez-García, Nick M. Waddell, Nicola Waddell, Claes Wadelius, Lina Wadi, Rabea Wagener, Jeremiah A. Wala, Jian Wang, Jiayin Wang, Linghua Wang, Qi Wang, Wenyi Wang, Yumeng Wang, Zhining Wang, Paul M. Waring, Hans-Jörg Warnatz, Jonathan Warrell, Anne Y. Warren, Sebastian M. Waszak, David C. Wedge, Dieter Weichenhan, Paul Weinberger, John N. Weinstein, Joachim Weischenfeldt, Daniel J. Weisenberger, Ian Welch, Michael C. Wendl, Johannes Werner, Justin P. Whalley, David A. Wheeler, Hayley C. Whitaker, Dennis Wigle, Matthew D. Wilkerson, Ashley Williams, James S. Wilmott, Gavin W. Wilson, Julie M. Wilson, Richard K. Wilson, Boris Winterhoff, Jeffrey A. Wintersinger, Maciej Wiznerowicz, Stephan Wolf, Bernice H. Wong, Tina Wong, Winghing Wong, Youngchoon Woo, Scott Wood, Bradly G. Wouters, Adam J. Wright, Derek W. Wright, Mark H. Wright, Chin-Lee Wu, Dai-Ying Wu, Guanming Wu, Jianmin Wu, Kui Wu, Yang Wu, Zhenggang Wu, Liu Xi, Tian Xia, Qian Xiang, Xiao Xiao, Rui Xing, Heng Xiong, Qinying Xu, Yanxun Xu, Hong Xue, Shinichi Yachida, Sergei Yakneen, Rui Yamaguchi, Takafumi N. Yamaguchi, Masakazu Yamamoto, Shogo Yamamoto, Hiroki Yamaue, Fan Yang, Huanming Yang, Jean Y. Yang, Liming Yang, Lixing Yang, Shanlin Yang, Tsun-Po Yang, Yang Yang, Xiaotong Yao, Marie-Laure Yaspo, Lucy Yates, Christina Yau, Chen Ye, Kai Ye, Venkata D. Yellapantula, Christopher J. Yoon, Sung-Soo Yoon, Fouad Yousif, Jun Yu, Kaixian Yu, Willie Yu, Yingyan Yu, Ke Yuan, Yuan Yuan, Denis Yuen, Christina K. Yung, Olga Zaikova, Jorge Zamora, Marc Zapatka, Jean C. Zenklusen, Thorsten Zenz, Nikolajs Zeps, Cheng-Zhong Zhang, Fan Zhang, Hailei Zhang, Hongwei Zhang, Hongxin Zhang, Jiashan Zhang, Jing Zhang, Junjun Zhang, Xiuqing Zhang, Xuanping Zhang, Yan Zhang, Zemin Zhang, Zhongming Zhao, Liangtao Zheng, Xiuqing Zheng, Wanding Zhou, Yong Zhou, Bin Zhu, Hongtu Zhu, Jingchun Zhu, Shida Zhu, Lihua Zou, Xueqing Zou, Anna deFazio, Nicholas van As, Carolien H. M. van Deurzen, Marc J. van de Vijver, L. van’t Veer, Christian von Mering

**Affiliations:** 1 Cancer Genome Project, Wellcome Trust Sanger Institute, Hinxton, UK; 2 Totient Inc, Cambridge, MA USA; 3 The Broad Institute of Harvard and MIT, Cambridge, MA USA; 4 Bioinformatics and Integrative Genomics, Harvard University, Cambridge, MA USA; 5 Department of Cancer Biology, Dana-Farber Cancer Institute, Boston, MA USA; 6 Weill Cornell Medical College, New York, NY USA; 7 European Molecular Biology Laboratory, Genome Biology Unit, Heidelberg, Germany; 8 Department of Molecular Biology, Rosenstiel Basic Medical Sciences Research Center, Brandeis University, Waltham, MA USA; 9 New York Genome Center, New York, NY USA; 11 Biotech Research & Innovation Centre (BRIC), The Finsen Laboratory, Rigshospitalet, University of Copenhagen, Copenhagen, Denmark; 12 Department of Haematology, University of Cambridge, Cambridge, UK; 14 University of Texas MD Anderson Cancer Center, Houston, TX USA; 15 Department of Zoology, Genetics and Physical Anthropology, University of Santiago de Compostela, Santiago de Compostela, Spain; 16 Centre for Research in Molecular Medicine and Chronic Diseases (CIMUS), University of Santiago de Compostela, Santiago de Compostela, Spain; 17 The Biomedical Research Centre (CINBIO), University of Vigo, Vigo, Spain; 18 Transmissible Cancer Group, Department of Veterinary Medicine, University of Cambridge, Cambridge, UK; 19 Computational Biology Program, Ontario Institute for Cancer Research, Toronto, Ontario Canada; 20 Department of Medical Biophysics, University of Toronto, Toronto, Ontario Canada; 21 Department of Pharmacology, University of Toronto, Toronto, Ontario Canada; 22 University of California Los Angeles, Los Angeles, CA USA; 23 Peter MacCallum Cancer Centre, Melbourne, Victoria Australia; 24 Sir Peter MacCallum Department of Oncology, University of Melbourne, Melbourne, Victoria Australia; 25 National Center for Tumor Diseases (NCT) Heidelberg, Heidelberg, Germany; 26 Division of Applied Bioinformatics, German Cancer Research Center (DKFZ), Heidelberg, Germany; 27 German Cancer Genome Consortium (DKTK), Heidelberg, Germany; 28 Johns Hopkins School of Medicine, Baltimore, MD USA; 29 Faculty of Medicine, Department of Biochemistry, Microbiology and Immunology, University of Ottawa, Ottawa, Ontario Canada; 30 Centre for Molecular Science Informatics, Department of Chemistry, University of Cambridge, Cambridge, UK; 31 Department of Biomedical Informatics, Harvard Medical School, Boston, MA USA; 32 Ludwig Center, Harvard Medical School, Boston, MA USA; 33 Barcelona Supercomputing Center (BSC), Barcelona, Spain; 34 Cancer Research UK Cambridge Institute, University of Cambridge, Cambridge, UK; 35 University of Cambridge, Cambridge, UK; 36 Sidra Medicine, Doha, Qatar; 37 Barcelona Supercomputing Center (BSC), Barcelona, Spain; 38 Queensland Centre for Medical Genomics, Institute for Molecular Bioscience, The University of Queensland, Brisbane, Queensland Australia; 39 The Azrieli Faculty of Medicine, Bar-Ilan University, Safed, Israel; 40 Department of Computer Science, Princeton University, Princeton, NJ USA; 41 Department of Computer Science, Yale University, New Haven, CT USA; 42 Program in Computational Biology and Bioinformatics, Yale University, New Haven, CT USA; 43 Genome Integrity and Structural Biology Laboratory, National Institute of Environmental Health Sciences (NIEHS), Durham, NC USA; 44 Biomolecular Engineering Department, University of California, Santa Cruz, Santa Cruz, CA USA; 45 Massachusetts General Hospital Center for Cancer Research, Charlestown, MA USA; 46 Heidelberg Center for Personalized Oncology (DKFZ-HIPO), German Cancer Research Center (DKFZ), Heidelberg, Germany; 47 Hopp Children’s Cancer Center (KiTZ), Heidelberg, Germany; 48 Pediatric Glioma Research Group, German Cancer Research Center (DKFZ), Heidelberg, Germany; 49 Korea Advanced Institute of Science and Technology, Daejeon, South Korea; 50 Skolkovo Institute of Science and Technology, Moscow, Russia; 51 A. A. Kharkevich Institute of Information Transmission Problems, Moscow, Russia; 52 Dmitry Rogachev National Research Center of Pediatric Hematology, Oncology and Immunology, Moscow, Russia; 53 Integrative Bioinformatics Support Group, National Institute of Environmental Health Sciences (NIEHS), Durham, NC USA; 54 Center For Medical Innovation, Seoul National University Hospital, Seoul, South Korea; 55 Department of Internal Medicine, Seoul National University Hospital, Seoul, South Korea; 56 Division of Genetics and Genomics, Harvard Medical School, Boston, MA USA; 57 Boston Children’s Hospital, Boston, MA USA; 58 School of Medicine/School of Mathematics and Statistics, University of St Andrews, St Andrews, UK; 59 Department of Physiology and Biophysics, Weill Cornell Medicine, New York, NY USA; 60 Institute for Computational Biomedicine, Weill Cornell Medicine, New York, NY USA; 61 Englander Institute for Precision Medicine, Weill Cornell Medicine, New York, NY USA; 62 Dana-Farber Cancer Institute, Boston, MA USA; 63 The Institute of Medical Science, The University of Tokyo, Tokyo, Japan; 64 RIKEN Center for Integrative Medical Sciences, Yokohama, Japan; 65 Department of Molecular Biophysics and Biochemistry, Yale University, New Haven, CT USA; 66 Universitat Pompeu Fabra (UPF), Barcelona, Spain; 67 Centre for Genomic Regulation (CRG), The Barcelona Institute of Science and Technology, Barcelona, Spain; 68 Institute of Medical Genetics and Applied Genomics, University of Tübingen, Tübingen, Germany; 69 Department of Genetics and Computational Biology, QIMR Berghofer Medical Research Institute, Brisbane, Queensland Australia; 70 Institute for Molecular Bioscience, University of Queensland, Brisbane, Queensland Australia; 71 German Cancer Research Center (DKFZ), Heidelberg, Germany; 72 School of Molecular Biosciences and Center for Reproductive Biology, Washington State University, Pullman, WA USA; 73 Cancer Research Institute, Beth Israel Deaconess Medical Center, Boston, MA USA; 74 Faculty of Biosciences, Heidelberg University, Heidelberg, Germany; 75 Institució Catalana de Recerca i Estudis Avançats (ICREA), Barcelona, Spain; 76 Ben May Department for Cancer Research, Department of Human Genetics, The University of Chicago, Chicago, IL USA; 77 Tri-institutional PhD Program of Computational Biology and Medicine, Weill Cornell Medicine, New York, NY USA; 200 Applied Tumor Genomics Research Program, Research Programs Unit, University of Helsinki, Helsinki, Finland; 201 Wellcome Sanger Institute, Wellcome Genome Campus, Hinxton, UK; 202 Memorial Sloan Kettering Cancer Center, New York, NY USA; 203 Genome Science Division, Research Center for Advanced Science and Technology, University of Tokyo, Tokyo, Japan; 204 Department of Surgery, University of Chicago, Chicago, IL USA; 205 Department of Surgery, Division of Hepatobiliary and Pancreatic Surgery, School of Medicine, Keimyung University Dongsan Medical Center, Daegu, South Korea; 206 Department of Oncology, Gil Medical Center, Gachon University, Incheon, South Korea; 207 Hiroshima University, Hiroshima, Japan; 208 Department of Bioinformatics and Computational Biology, The University of Texas MD Anderson Cancer Center, Houston, TX USA; 209 University of Texas MD Anderson Cancer Center, Houston, TX USA; 210 King Faisal Specialist Hospital and Research Centre, Al Maather, Riyadh, Saudi Arabia; 211 Bioinformatics Unit, Spanish National Cancer Research Centre (CNIO), Madrid, Spain; 212 Bioinformatics Core Facility, University Medical Center Hamburg, Hamburg, Germany; 213 Heinrich Pette Institute, Leibniz Institute for Experimental Virology, Hamburg, Germany; 214 Ontario Tumour Bank, Ontario Institute for Cancer Research, Toronto, ON Canada; 215 Department of Pathology, The University of Texas MD Anderson Cancer Center, Houston, TX USA; 216 Laboratory of Pathology, Center for Cancer Research, National Cancer Institute, Bethesda, MD USA; 217 Department of Cellular and Molecular Medicine and Department of Bioengineering, University of California San Diego, La Jolla, CA USA; 218 UC San Diego Moores Cancer Center, San Diego, CA USA; 219 Canada’s Michael Smith Genome Sciences Centre, BC Cancer, Vancouver, BC Canada; 220 Sir Peter MacCallum Department of Oncology, Peter MacCallum Cancer Centre, University of Melbourne, Melbourne, VIC Australia; 221 Centre for Research in Molecular Medicine and Chronic Diseases (CiMUS), Universidade de Santiago de Compostela, Santiago de Compostela, Spain; 222 Department of Zoology, Genetics and Physical Anthropology, (CiMUS), Universidade de Santiago de Compostela, Santiago de Compostela, Spain; 223 The Biomedical Research Centre (CINBIO), Universidade de Vigo, Vigo, Spain; 224 Royal National Orthopaedic Hospital - Bolsover, London, UK; 225 Department of Genomic Medicine, The University of Texas MD Anderson Cancer Center, Houston, TX USA; 226 Quantitative and Computational Biosciences Graduate Program, Baylor College of Medicine, Houston, TX USA; 227 The Jackson Laboratory for Genomic Medicine, Farmington, CT USA; 228 Genome Informatics Program, Ontario Institute for Cancer Research, Toronto, ON Canada; 229 Institute of Human Genetics, Christian-Albrechts-University, Kiel, Germany; 230 Institute of Human Genetics, Ulm University and Ulm University Medical Center, Ulm, Germany; 231 Queensland Centre for Medical Genomics, Institute for Molecular Bioscience, University of Queensland, St. Lucia, Brisbane, QLD Australia; 232 Salford Royal NHS Foundation Trust, Salford, UK; 233 Department of Surgery, Pancreas Institute, University and Hospital Trust of Verona, Verona, Italy; 234 Molecular and Medical Genetics, OHSU Knight Cancer Institute, Oregon Health and Science University, Portland, OR USA; 235 Department of Molecular Oncology, BC Cancer Research Centre, Vancouver, BC Canada; 236 The McDonnell Genome Institute at Washington University, St. Louis, MO USA; 237 University College London, London, UK; 238 Division of Cancer Genomics, National Cancer Center Research Institute, National Cancer Center, Tokyo, Japan; 239 DLR Project Management Agency, Bonn, Germany; 240 Tokyo Women’s Medical University, Tokyo, Japan; 241 Center for Molecular Oncology, Memorial Sloan Kettering Cancer Center, New York, NY USA; 242 Los Alamos National Laboratory, Los Alamos, NM USA; 243 Department of Pathology, University Health Network, Toronto General Hospital, Toronto, ON Canada; 244 Nottingham University Hospitals NHS Trust, Nottingham, UK; 245 Epigenomics and Cancer Risk Factors, German Cancer Research Center (DKFZ), Heidelberg, Germany; 246 Computational Biology Program, Ontario Institute for Cancer Research, Toronto, ON Canada; 247 Department of Molecular Genetics, University of Toronto, Toronto, ON Canada; 248 Vector Institute, Toronto, ON Canada; 249 Hematopathology Section, Institute of Pathology, Christian-Albrechts-University, Kiel, Germany; 250 Department of Pathology and Laboratory Medicine, School of Medicine, University of North Carolina at Chapel Hill, Chapel Hill, NC USA; 251 Department of Cancer Genetics, Institute for Cancer Research, Oslo University Hospital, The Norwegian Radium Hospital, Oslo, Norway; 252 Pathology, Hospital Clinic, Institut d’Investigacions Biomèdiques August Pi i Sunyer (IDIBAPS), University of Barcelona, Barcelona, Spain; 253 Department of Veterinary Medicine, Transmissible Cancer Group, University of Cambridge, Cambridge, UK; 254 Alvin J. Siteman Cancer Center, Washington University School of Medicine, St. Louis, MO USA; 255 Wolfson Wohl Cancer Research Centre, Institute of Cancer Sciences, University of Glasgow, Glasgow, UK; 256 Lineberger Comprehensive Cancer Center, University of North Carolina at Chapel Hill, Chapel Hill, NC USA; 257 Broad Institute of MIT and Harvard, Cambridge, MA USA; 258 Dana-Farber/Boston Children’s Cancer and Blood Disorders Center, Boston, MA USA; 259 Department of Pediatrics, Harvard Medical School, Boston, MA USA; 260 Leeds Institute of Medical Research @ St. James’s, University of Leeds, St. James’s University Hospital, Leeds, UK; 261 Department of Pathology and Diagnostics, University and Hospital Trust of Verona, Verona, Italy; 262 Department of Surgery, Princess Alexandra Hospital, Brisbane, QLD Australia; 263 Surgical Oncology Group, Diamantina Institute, University of Queensland, Brisbane, QLD Australia; 264 Department of Population and Quantitative Health Sciences, Case Western Reserve University School of Medicine, Cleveland, OH USA; 265 Research Health Analytics and Informatics, University Hospitals Cleveland Medical Center, Cleveland, OH USA; 266 Gloucester Royal Hospital, Gloucester, UK; 267 European Molecular Biology Laboratory, European Bioinformatics Institute (EMBL-EBI), Cambridge, UK; 268 Diagnostic Development, Ontario Institute for Cancer Research, Toronto, ON Canada; 269 Barcelona Supercomputing Center (BSC), Barcelona, Spain; 270 Arnie Charbonneau Cancer Institute, University of Calgary, Calgary, AB Canada; 271 Departments of Surgery and Oncology, University of Calgary, Calgary, AB Canada; 272 Department of Pathology, Oslo University Hospital, The Norwegian Radium Hospital, Oslo, Norway; 273 PanCuRx Translational Research Initiative, Ontario Institute for Cancer Research, Toronto, ON Canada; 274 Department of Oncology, Sidney Kimmel Comprehensive Cancer Center at Johns Hopkins University School of Medicine, Baltimore, MD USA; 275 University Hospital Southampton NHS Foundation Trust, Southampton, UK; 276 Royal Stoke University Hospital, Stoke-on-Trent, UK; 277 Genome Sequence Informatics, Ontario Institute for Cancer Research, Toronto, ON Canada; 278 Human Longevity Inc, San Diego, CA USA; 279 Olivia Newton-John Cancer Research Institute, La Trobe University, Heidelberg, VIC Australia; 280 Computer Network Information Center, Chinese Academy of Sciences, Beijing, China; 281 Genome Canada, Ottawa, ON Canada; 282 CNAG-CRG, Centre for Genomic Regulation (CRG), Barcelona Institute of Science and Technology (BIST), Barcelona, Spain; 283 Universitat Pompeu Fabra (UPF), Barcelona, Spain; 284 Buck Institute for Research on Aging, Novato, CA USA; 285 Duke University Medical Center, Durham, NC USA; 286 Department of Human Genetics, Hannover Medical School, Hannover, Germany; 287 Center for Bioinformatics and Functional Genomics, Cedars-Sinai Medical Center, Los Angeles, CA USA; 288 Department of Biomedical Sciences, Cedars-Sinai Medical Center, Los Angeles, CA USA; 289 The Hebrew University Faculty of Medicine, Jerusalem, Israel; 290 Barts Cancer Institute, Barts and the London School of Medicine and Dentistry, Queen Mary University of London, London, UK; 291 Department of Computer Science, Bioinformatics Group, University of Leipzig, Leipzig, Germany; 292 Interdisciplinary Center for Bioinformatics, University of Leipzig, Leipzig, Germany; 293 Transcriptome Bioinformatics, LIFE Research Center for Civilization Diseases, University of Leipzig, Leipzig, Germany; 294 Department of Medical Oncology, Dana-Farber Cancer Institute, Boston, MA USA; 295 Department of Cancer Biology, Dana-Farber Cancer Institute, Boston, MA USA; 296 Harvard Medical School, Boston, MA USA; 297 USC Norris Comprehensive Cancer Center, University of Southern California, Los Angeles, CA USA; 298 Department of Diagnostics and Public Health, University and Hospital Trust of Verona, Verona, Italy; 299 Department of Mathematics, Aarhus University, Aarhus, Denmark; 300 Department of Molecular Medicine (MOMA), Aarhus University Hospital, Aarhus N, Denmark; 301 Instituto Carlos Slim de la Salud, Mexico City, Mexico; 302 Department of Medical Biophysics, University of Toronto, Toronto, ON Canada; 303 Cancer Division, Garvan Institute of Medical Research, Kinghorn Cancer Centre, University of New South Wales (UNSW Sydney), Sydney, NSW Australia; 304 South Western Sydney Clinical School, Faculty of Medicine, University of New South Wales (UNSW Sydney), Liverpool, NSW Australia; 305 West of Scotland Pancreatic Unit, Glasgow Royal Infirmary, Glasgow, UK; 306 Center for Digital Health, Berlin Institute of Health and Charitè - Universitätsmedizin Berlin, Berlin, Germany; 307 Heidelberg Center for Personalized Oncology (DKFZ-HIPO), German Cancer Research Center (DKFZ), Heidelberg, Germany; 308 The Preston Robert Tisch Brain Tumor Center, Duke University Medical Center, Durham, NC USA; 309 Massachusetts General Hospital, Boston, MA USA; 310 National Institute of Biomedical Genomics, Kalyani, West Bengal India; 311 Institute of Clinical Medicine and Institute of Oral Biology, University of Oslo, Oslo, Norway; 312 University of North Carolina at Chapel Hill, Chapel Hill, NC USA; 313 ARC-Net Centre for Applied Research on Cancer, University and Hospital Trust of Verona, Verona, Italy; 314 The Institute of Cancer Research, London, UK; 315 Centre for Computational Biology, Duke-NUS Medical School, Singapore, Singapore; 316 Programme in Cancer and Stem Cell Biology, Duke-NUS Medical School, Singapore, Singapore; 317 Division of Oncology and Pathology, Department of Clinical Sciences Lund, Lund University, Lund, Sweden; 318 Department of Pediatric Oncology, Hematology and Clinical Immunology, Heinrich-Heine-University, Düsseldorf, Germany; 319 Laboratory for Medical Science Mathematics, RIKEN Center for Integrative Medical Sciences, Yokohama, Japan; 320 RIKEN Center for Integrative Medical Sciences, Yokohama, Japan; 321 Department of Internal Medicine/Hematology, Friedrich-Ebert-Hospital, Neumünster, Germany; 322 Departments of Dermatology and Pathology, Yale University, New Haven, CT USA; 323 Centre for Genomic Regulation (CRG), The Barcelona Institute of Science and Technology, Barcelona, Spain; 324 Radcliffe Department of Medicine, University of Oxford, Oxford, UK; 325 Canadian Center for Computational Genomics, McGill University, Montreal, QC Canada; 326 Department of Human Genetics, McGill University, Montreal, QC Canada; 327 Department of Human Genetics, University of California Los Angeles, Los Angeles, CA USA; 328 Department of Pharmacology, University of Toronto, Toronto, ON Canada; 329 Faculty of Medicine and Health Technology, Tampere University and Tays Cancer Center, Tampere University Hospital, Tampere, Finland; 330 Haematology, Leeds Teaching Hospitals NHS Trust, Leeds, UK; 331 Translational Research and Innovation, Centre Léon Bérard, Lyon, France; 332 Fox Chase Cancer Center, Philadelphia, PA USA; 333 International Agency for Research on Cancer, World Health Organization, Lyon, France; 334 Earlham Institute, Norwich, UK; 335 Norwich Medical School, University of East Anglia, Norwich, UK; 336 Department of Molecular Biology, Faculty of Science, Radboud Institute for Molecular Life Sciences, Radboud University, Nijmegen, HB The Netherlands; 337 CRUK Manchester Institute and Centre, Manchester, UK; 338 Department of Radiation Oncology, University of Toronto, Toronto, ON Canada; 339 Division of Cancer Sciences, Manchester Cancer Research Centre, University of Manchester, Manchester, UK; 340 Radiation Medicine Program, Princess Margaret Cancer Centre, Toronto, ON Canada; 341 Department of Pathology, Brigham and Women’s Hospital, Harvard Medical School, Boston, MA USA; 342 Department of Surgery, Division of Thoracic Surgery, The Johns Hopkins University School of Medicine, Baltimore, MD USA; 343 Division of Molecular Pathology, The Netherlands Cancer Institute, Oncode Institute, Amsterdam, CX The Netherlands; 344 Department of Biomolecular Engineering, University of California Santa Cruz, Santa Cruz, CA USA; 345 UC Santa Cruz Genomics Institute, University of California Santa Cruz, Santa Cruz, CA USA; 346 Division of Applied Bioinformatics, German Cancer Research Center (DKFZ), Heidelberg, Germany; 347 German Cancer Consortium (DKTK), German Cancer Research Center (DKFZ), Heidelberg, Germany; 348 National Center for Tumor Diseases (NCT) Heidelberg, Heidelberg, Germany; 349 Center for Biological Sequence Analysis, Department of Bio and Health Informatics, Technical University of Denmark, Lyngby, Denmark; 350 Novo Nordisk Foundation Center for Protein Research, University of Copenhagen, Copenhagen, Denmark; 351 Institute for Molecular Bioscience, University of Queensland, St. Lucia, Brisbane, QLD Australia; 352 Biomedical Engineering, Oregon Health and Science University, Portland, OR USA; 353 Division of Theoretical Bioinformatics, German Cancer Research Center (DKFZ), Heidelberg, Germany; 354 Institute of Pharmacy and Molecular Biotechnology and BioQuant, Heidelberg University, Heidelberg, Germany; 355 Federal Ministry of Education and Research, Berlin, Germany; 356 Melanoma Institute Australia, University of Sydney, Sydney, NSW Australia; 357 Pediatric Hematology and Oncology, University Hospital Muenster, Muenster, Germany; 358 Department of Pathology, Johns Hopkins University School of Medicine, Baltimore, MD USA; 359 McKusick-Nathans Institute of Genetic Medicine, Sidney Kimmel Comprehensive Cancer Center at Johns Hopkins University School of Medicine, Baltimore, MD USA; 360 Foundation Medicine, Inc, Cambridge, MA USA; 361 Department of Biomedical Data Science, Stanford University School of Medicine, Stanford, CA USA; 362 Department of Genetics, Stanford University School of Medicine, Stanford, CA USA; 363 Bakar Computational Health Sciences Institute and Department of Pediatrics, University of California, San Francisco, CA USA; 364 Institute of Clinical Medicine, Faculty of Medicine, University of Oslo, Oslo, Norway; 365 National Cancer Institute, National Institutes of Health, Bethesda, MD USA; 366 Royal Marsden NHS Foundation Trust, London and Sutton, UK; 367 Genome Biology Unit, European Molecular Biology Laboratory (EMBL), Heidelberg, Germany; 368 Department of Oncology, University of Cambridge, Cambridge, UK; 369 Li Ka Shing Centre, Cancer Research UK Cambridge Institute, University of Cambridge, Cambridge, UK; 370 Institut Gustave Roussy, Villejuif, France; 371 Cambridge University Hospitals NHS Foundation Trust, Cambridge, UK; 372 Department of Haematology, University of Cambridge, Cambridge, UK; 373 Anatomia Patológica, Hospital Clinic, Institut d’Investigacions Biomèdiques August Pi i Sunyer (IDIBAPS), University of Barcelona, Barcelona, Spain; 374 Spanish Ministry of Science and Innovation, Madrid, Spain; 375 University of Michigan Comprehensive Cancer Center, Ann Arbor, MI USA; 376 Department for BioMedical Research, University of Bern, Bern, Switzerland; 377 Department of Medical Oncology, Inselspital, University Hospital and University of Bern, Bern, Switzerland; 378 Graduate School for Cellular and Biomedical Sciences, University of Bern, Bern, Switzerland; 379 University of Pavia, Pavia, Italy; 380 University of Alabama at Birmingham, Birmingham, AL USA; 381 UHN Program in BioSpecimen Sciences, Toronto General Hospital, Toronto, ON Canada; 382 Department of Urology, Icahn School of Medicine at Mount Sinai, New York, NY USA; 383 Centre for Law and Genetics, University of Tasmania, Sandy Bay Campus, Hobart, TAS Australia; 384 Faculty of Biosciences, Heidelberg University, Heidelberg, Germany; 385 Department of Biochemistry, Microbiology and Immunology, Faculty of Medicine, University of Ottawa, Ottawa, ON Canada; 386 Division of Anatomic Pathology, Mayo Clinic, Rochester, MN USA; 387 Division of Cancer Epidemiology and Genetics, National Cancer Institute, National Institutes of Health, Bethesda, MD USA; 388 Illawarra Shoalhaven Local Health District L3 Illawarra Cancer Care Centre, Wollongong Hospital, Wollongong, NSW Australia; 389 BioForA, French National Institute for Agriculture, Food, and Environment (INRAE), ONF, Orléans, France; 390 Department of Biostatistics, Bloomberg School of Public Health, Johns Hopkins University, Baltimore, MD USA; 391 University of California San Diego, San Diego, CA USA; 392 Division of Experimental Pathology, Mayo Clinic, Rochester, MN USA; 393 Centre for Cancer Research, The Westmead Institute for Medical Research, University of Sydney, Sydney, NSW Australia; 394 Department of Gynaecological Oncology, Westmead Hospital, Sydney, NSW Australia; 395 PDXen Biosystems Inc, Seoul, South Korea; 396 Korea Advanced Institute of Science and Technology, Daejeon, South Korea; 397 Electronics and Telecommunications Research Institute, Daejeon, South Korea; 398 Institut National du Cancer (INCA), Boulogne-Billancourt, France; 399 Department of Genetics, Informatics Institute, University of Alabama at Birmingham, Birmingham, AL USA; 400 Division of Medical Oncology, National Cancer Centre, Singapore, Singapore; 401 Medical Oncology, University and Hospital Trust of Verona, Verona, Italy; 402 Department of Pediatrics, University Hospital Schleswig-Holstein, Kiel, Germany; 403 Hepatobiliary/Pancreatic Surgical Oncology Program, University Health Network, Toronto, ON Canada; 404 School of Biological Sciences, University of Auckland, Auckland, New Zealand; 405 Department of Surgery, University of Melbourne, Parkville, VIC Australia; 406 The Murdoch Children’s Research Institute, Royal Children’s Hospital, Parkville, VIC Australia; 407 Walter and Eliza Hall Institute, Parkville, VIC Australia; 408 Vancouver Prostate Centre, Vancouver, Canada; 409 Lunenfeld-Tanenbaum Research Institute, Mount Sinai Hospital, Toronto, ON Canada; 410 University of East Anglia, Norwich, UK; 411 Norfolk and Norwich University Hospital NHS Trust, Norwich, UK; 412 Victorian Institute of Forensic Medicine, Southbank, VIC Australia; 413 Department of Biomedical Informatics, Harvard Medical School, Boston, MA USA; 414 Department of Chemistry, Centre for Molecular Science Informatics, University of Cambridge, Cambridge, UK; 415 Ludwig Center at Harvard Medical School, Boston, MA USA; 416 Human Genome Sequencing Center, Baylor College of Medicine, Houston, TX USA; 417 Peter MacCallum Cancer Centre, University of Melbourne, Melbourne, VIC Australia; 418 Physics Division, Optimization and Systems Biology Lab, Massachusetts General Hospital, Boston, MA USA; 419 Department of Medicine, Baylor College of Medicine, Houston, TX USA; 420 University of Cologne, Cologne, Germany; 421 International Genomics Consortium, Phoenix, AZ USA; 422 Genomics Research Program, Ontario Institute for Cancer Research, Toronto, ON Canada; 423 Barking Havering and Redbridge University Hospitals NHS Trust, Romford, UK; 424 Children’s Hospital at Westmead, University of Sydney, Sydney, NSW Australia; 425 Department of Medicine, Section of Endocrinology, University and Hospital Trust of Verona, Verona, Italy; 426 Computational Biology Center, Memorial Sloan Kettering Cancer Center, New York, NY USA; 427 Department of Biology, ETH Zurich, Zürich, Switzerland; 428 Department of Computer Science, ETH Zurich, Zurich, Switzerland; 429 SIB Swiss Institute of Bioinformatics, Lausanne, Switzerland; 430 Weill Cornell Medical College, New York, NY USA; 431 Academic Department of Medical Genetics, University of Cambridge, Addenbrooke’s Hospital, Cambridge, UK; 432 MRC Cancer Unit, University of Cambridge, Cambridge, UK; 433 Departments of Pediatrics and Genetics, University of North Carolina at Chapel Hill, Chapel Hill, NC USA; 434 Seven Bridges Genomics, Charlestown, MA USA; 435 Annai Systems, Inc, Carlsbad, CA USA; 436 Department of Pathology, General Hospital of Treviso, Department of Medicine, University of Padua, Treviso, Italy; 437 Department of Computational Biology, University of Lausanne, Lausanne, Switzerland; 438 Department of Genetic Medicine and Development, University of Geneva Medical School, Geneva, CH Switzerland; 439 Swiss Institute of Bioinformatics, University of Geneva, Geneva, CH Switzerland; 440 The Francis Crick Institute, London, UK; 441 University of Leuven, Leuven, Belgium; 442 Institute of Medical Genetics and Applied Genomics, University of Tübingen, Tübingen, Germany; 443 Computational and Systems Biology, Genome Institute of Singapore, Singapore, Singapore; 444 School of Computing, National University of Singapore, Singapore, Singapore; 445 Big Data Institute, Li Ka Shing Centre, University of Oxford, Oxford, UK; 446 Biomedical Data Science Laboratory, Francis Crick Institute, London, UK; 447 Bioinformatics Group, Department of Computer Science, University College London, London, UK; 448 The Edward S. Rogers Sr. Department of Electrical and Computer Engineering, University of Toronto, Toronto, ON Canada; 449 Breast Cancer Translational Research Laboratory JC Heuson, Institut Jules Bordet, Brussels, Belgium; 450 Department of Oncology, Laboratory for Translational Breast Cancer Research, KU Leuven, Leuven, Belgium; 451 Institute for Research in Biomedicine (IRB Barcelona), The Barcelona Institute of Science and Technology, Barcelona, Spain; 452 Research Program on Biomedical Informatics, Universitat Pompeu Fabra, Barcelona, Spain; 453 Division of Medical Oncology, Princess Margaret Cancer Centre, Toronto, ON Canada; 454 Department of Physiology and Biophysics, Weill Cornell Medicine, New York, NY USA; 455 Institute for Computational Biomedicine, Weill Cornell Medicine, New York, NY USA; 456 Department of Pathology, UPMC Shadyside, Pittsburgh, PA USA; 457 Independent Consultant, Wellesley, USA; 458 Department of Cell and Molecular Biology, Science for Life Laboratory, Uppsala University, Uppsala, Sweden; 459 Department of Medicine and Department of Genetics, Washington University School of Medicine, St. Louis, St. Louis, MO USA; 460 Hefei University of Technology, Anhui, China; 461 Translational Cancer Research Unit, GZA Hospitals St.-Augustinus, Center for Oncological Research, Faculty of Medicine and Health Sciences, University of Antwerp, Antwerp, Belgium; 462 Simon Fraser University, Burnaby, BC Canada; 463 University of Pennsylvania, Philadelphia, PA USA; 464 Faculty of Science and Technology, University of Vic—Central University of Catalonia (UVic-UCC), Vic, Spain; 465 The Wellcome Trust, London, UK; 466 The Hospital for Sick Children, Toronto, ON Canada; 467 Department of Pathology, Queen Elizabeth University Hospital, Glasgow, UK; 468 Department of Genetics and Computational Biology, QIMR Berghofer Medical Research Institute, Brisbane, QLD Australia; 469 Department of Oncology, Centre for Cancer Genetic Epidemiology, University of Cambridge, Cambridge, UK; 470 Department of Public Health and Primary Care, Centre for Cancer Genetic Epidemiology, University of Cambridge, Cambridge, UK; 471 Prostate Cancer Canada, Toronto, ON Canada; 472 University of Cambridge, Cambridge, UK; 473 Department of Laboratory Medicine, Translational Cancer Research, Lund University Cancer Center at Medicon Village, Lund University, Lund, Sweden; 474 Heidelberg University, Heidelberg, Germany; 475 New BIH Digital Health Center, Berlin Institute of Health (BIH) and Charité - Universitätsmedizin Berlin, Berlin, Germany; 476 CIBER Epidemiología y Salud Pública (CIBERESP), Madrid, Spain; 477 Research Group on Statistics, Econometrics and Health (GRECS), UdG, Barcelona, Spain; 478 Quantitative Genomics Laboratories (qGenomics), Barcelona, Spain; 479 Icelandic Cancer Registry, Icelandic Cancer Society, Reykjavik, Iceland; 480 State Key Laboratory of Cancer Biology, and Xijing Hospital of Digestive Diseases, Fourth Military Medical University, Shaanxi, China; 481 Department of Medicine (DIMED), Surgical Pathology Unit, University of Padua, Padua, Italy; 482 Rigshospitalet, Copenhagen, Denmark; 483 Center for Cancer Genomics, National Cancer Institute, National Institutes of Health, Bethesda, MD USA; 484 Department of Biochemistry and Molecular Medicine, University of Montreal, Montreal, QC Canada; 485 Australian Institute of Tropical Health and Medicine, James Cook University, Douglas, QLD Australia; 486 Department of Neuro-Oncology, Istituto Neurologico Besta, Milano, Italy; 487 Bioplatforms Australia, North Ryde, NSW Australia; 488 Department of Pathology (Research), University College London Cancer Institute, London, UK; 489 Department of Surgical Oncology, Princess Margaret Cancer Centre, Toronto, ON Canada; 490 Department of Medical Oncology, Josephine Nefkens Institute and Cancer Genomics Centre, Erasmus Medical Center, Rotterdam, CN The Netherlands; 491 The University of Queensland Thoracic Research Centre, The Prince Charles Hospital, Brisbane, QLD Australia; 492 CIBIO/InBIO - Research Center in Biodiversity and Genetic Resources, Universidade do Porto, Vairão, Portugal; 493 HCA Laboratories, London, UK; 494 University of Liverpool, Liverpool, UK; 495 The Azrieli Faculty of Medicine, Bar-Ilan University, Safed, Israel; 496 Department of Neurosurgery, University of Florida, Gainesville, FL USA; 497 Department of Pathology, Graduate School of Medicine, University of Tokyo, Tokyo, Japan; 498 University of Milano Bicocca, Monza, Italy; 499 BGI-Shenzhen, Shenzhen, China; 500 Department of Pathology, Oslo University Hospital Ulleval, Oslo, Norway; 501 Center for Biomedical Informatics, Harvard Medical School, Boston, MA USA; 502 Department Biochemistry and Molecular Biomedicine, University of Barcelona, Barcelona, Spain; 503 Office of Cancer Genomics, National Cancer Institute, National Institutes of Health, Bethesda, MD USA; 504 Cancer Epigenomics, German Cancer Research Center (DKFZ), Heidelberg, Germany; 505 Department of Cancer Biology, The University of Texas MD Anderson Cancer Center, Houston, TX USA; 506 Department of Surgical Oncology, The University of Texas MD Anderson Cancer Center, Houston, TX USA; 507 Department of Computer Science, Yale University, New Haven, CT USA; 508 Department of Molecular Biophysics and Biochemistry, Yale University, New Haven, CT USA; 509 Program in Computational Biology and Bioinformatics, Yale University, New Haven, CT USA; 510 Center for Cancer Research, Massachusetts General Hospital, Boston, MA USA; 511 Department of Pathology, Massachusetts General Hospital, Boston, MA USA; 512 Department of Pathology, Memorial Sloan Kettering Cancer Center, New York, NY USA; 513 Division of Gastroenterology and Hepatology, Mayo Clinic, Rochester, MN USA; 514 University of Sydney, Sydney, NSW Australia; 515 University of Oxford, Oxford, UK; 516 Department of Surgery, Academic Urology Group, University of Cambridge, Cambridge, UK; 517 Department of Medicine II, University of Würzburg, Wuerzburg, Germany; 518 Sylvester Comprehensive Cancer Center, University of Miami, Miami, FL USA; 519 Institut Hospital del Mar d’Investigacions Mèdiques (IMIM), Barcelona, Spain; 520 Genome Integrity and Structural Biology Laboratory, National Institute of Environmental Health Sciences (NIEHS), Durham, NC USA; 521 St. Thomas’s Hospital, London, UK; 522 Osaka International Cancer Center, Osaka, Japan; 523 Department of Pathology, Skåne University Hospital, Lund University, Lund, Sweden; 524 Department of Medical Oncology, Beatson West of Scotland Cancer Centre, Glasgow, UK; 525 National Human Genome Research Institute, National Institutes of Health, Bethesda, MD USA; 526 Centre for Cancer Research, Victorian Comprehensive Cancer Centre, University of Melbourne, Melbourne, VIC Australia; 527 Department of Medicine, Section of Hematology/Oncology, University of Chicago, Chicago, IL USA; 528 German Center for Infection Research (DZIF), Partner Site Hamburg-Borstel-Lübeck-Riems, Hamburg, Germany; 529 Bioinformatics Research Centre (BiRC), Aarhus University, Aarhus, Denmark; 530 Department of Biotechnology, Ministry of Science and Technology, Government of India, New Delhi, Delhi India; 531 National Cancer Centre Singapore, Singapore, Singapore; 532 Brandeis University, Waltham, MA USA; 533 Department of Urologic Sciences, University of British Columbia, Vancouver, BC Canada; 534 Department of Internal Medicine, Stanford University, Stanford, CA USA; 535 The University of Texas Health Science Center at Houston, Houston, TX USA; 536 Imperial College NHS Trust, Imperial College, London, INY UK; 537 Senckenberg Institute of Pathology, University of Frankfurt Medical School, Frankfurt, Germany; 538 Department of Medicine, Division of Biomedical Informatics, UC San Diego School of Medicine, San Diego, CA USA; 539 Center for Precision Health, School of Biomedical Informatics, The University of Texas Health Science Center, Houston, TX USA; 540 Oxford Nanopore Technologies, New York, NY USA; 541 Institute of Medical Science, University of Tokyo, Tokyo, Japan; 542 Howard Hughes Medical Institute, University of California Santa Cruz, Santa Cruz, CA USA; 543 Wakayama Medical University, Wakayama, Japan; 544 Department of Internal Medicine, Division of Medical Oncology, Lineberger Comprehensive Cancer Center, University of North Carolina at Chapel Hill, Chapel Hill, NC USA; 545 University of Tennessee Health Science Center for Cancer Research, Memphis, TN USA; 546 Department of Histopathology, Salford Royal NHS Foundation Trust, Salford, UK; 547 Faculty of Biology, Medicine and Health, University of Manchester, Manchester, UK; 548 BIOPIC, ICG and College of Life Sciences, Peking University, Beijing, China; 549 Peking-Tsinghua Center for Life Sciences, Peking University, Beijing, China; 550 Children’s Hospital of Philadelphia, Philadelphia, PA USA; 551 Department of Bioinformatics and Computational Biology and Department of Systems Biology, The University of Texas MD Anderson Cancer Center, Houston, TX USA; 552 Karolinska Institute, Stockholm, Sweden; 553 The Donnelly Centre, University of Toronto, Toronto, ON Canada; 554 Department of Medical Genetics, College of Medicine, Hallym University, Chuncheon, South Korea; 555 Department of Experimental and Health Sciences, Institute of Evolutionary Biology (UPF-CSIC), Universitat Pompeu Fabra, Barcelona, Spain; 556 Health Data Science Unit, University Clinics, Heidelberg, Germany; 557 Massachusetts General Hospital Center for Cancer Research, Charlestown, MA USA; 558 Hokkaido University, Sapporo, Japan; 559 Department of Pathology and Clinical Laboratory, National Cancer Center Hospital, Tokyo, Japan; 560 Department of Genetics, University of North Carolina at Chapel Hill, Chapel Hill, NC USA; 561 Computational Biology, Leibniz Institute on Aging - Fritz Lipmann Institute (FLI), Jena, Germany; 562 University of Melbourne Centre for Cancer Research, Melbourne, VIC Australia; 563 University of Nebraska Medical Center, Omaha, NE USA; 564 Syntekabio Inc, Daejeon, South Korea; 565 Department of Pathology, Academic Medical Center, Amsterdam, AZ The Netherlands; 566 China National GeneBank-Shenzhen, Shenzhen, China; 567 Division of Molecular Genetics, German Cancer Research Center (DKFZ), Heidelberg, Germany; 568 Division of Life Science and Applied Genomics Center, Hong Kong University of Science and Technology, Clear Water Bay, Hong Kong, China; 569 Icahn School of Medicine at Mount Sinai, New York, NY USA; 570 Geneplus-Shenzhen, Shenzhen, China; 571 School of Computer Science and Technology, Xi’an Jiaotong University, Xi’an, China; 572 AbbVie, North Chicago, IL USA; 573 Institute of Pathology, Charité – University Medicine Berlin, Berlin, Germany; 574 Centre for Translational and Applied Genomics, British Columbia Cancer Agency, Vancouver, BC Canada; 575 Edinburgh Royal Infirmary, Edinburgh, UK; 576 Berlin Institute for Medical Systems Biology, Max Delbrück Center for Molecular Medicine, Berlin, Germany; 577 Department of Pediatric Immunology, Hematology and Oncology, University Hospital, Heidelberg, Germany; 578 German Cancer Research Center (DKFZ), Heidelberg, Germany; 579 Heidelberg Institute for Stem Cell Technology and Experimental Medicine (HI-STEM), Heidelberg, Germany; 580 Institute for Computational Biomedicine, Weill Cornell Medical College, New York, NY USA; 581 New York Genome Center, New York, NY USA; 582 Department of Urology, James Buchanan Brady Urological Institute, Johns Hopkins University School of Medicine, Baltimore, MD USA; 583 Department of Preventive Medicine, Graduate School of Medicine, The University of Tokyo, Tokyo, Japan; 584 Department of Molecular and Cellular Biology, Baylor College of Medicine, Houston, TX USA; 585 Department of Pathology and Immunology, Baylor College of Medicine, Houston, TX USA; 586 Michael E. DeBakey Veterans Affairs Medical Center, Houston, TX USA; 587 Technical University of Denmark, Lyngby, Denmark; 588 Department of Pathology, College of Medicine, Hanyang University, Seoul, South Korea; 589 Academic Unit of Surgery, School of Medicine, College of Medical, Veterinary and Life Sciences, University of Glasgow, Glasgow Royal Infirmary, Glasgow, UK; 590 Department of Pathology, Asan Medical Center, College of Medicine, Ulsan University, Songpa-gu, Seoul South Korea; 591 Science Writer, Garrett Park, MD USA; 592 International Cancer Genome Consortium (ICGC)/ICGC Accelerating Research in Genomic Oncology (ARGO) Secretariat, Ontario Institute for Cancer Research, Toronto, ON Canada; 593 University of Ljubljana, Ljubljana, Slovenia; 594 Department of Public Health Sciences, University of Chicago, Chicago, IL USA; 595 Research Institute, NorthShore University HealthSystem, Evanston, IL USA; 596 Department for Biomedical Research, University of Bern, Bern, Switzerland; 597 Centre of Genomics and Policy, McGill University and Génome Québec Innovation Centre, Montreal, QC Canada; 598 Carolina Center for Genome Sciences, University of North Carolina at Chapel Hill, Chapel Hill, NC USA; 599 Hopp Children’s Cancer Center (KiTZ), Heidelberg, Germany; 600 Pediatric Glioma Research Group, German Cancer Research Center (DKFZ), Heidelberg, Germany; 601 Cancer Research UK, London, UK; 602 Indivumed GmbH, Hamburg, Germany; 603 Genome Integration Data Center, Syntekabio, Inc, Daejeon, South Korea; 604 University Hospital Zurich, Zurich, Switzerland; 605 Clinical Bioinformatics, Swiss Institute of Bioinformatics, Geneva, Switzerland; 606 Institute for Pathology and Molecular Pathology, University Hospital Zurich, Zurich, Switzerland; 607 Institute of Molecular Life Sciences, University of Zurich, Zurich, Switzerland; 608 MRC Human Genetics Unit, MRC IGMM, University of Edinburgh, Edinburgh, UK; 609 Women’s Cancer Program at the Samuel Oschin Comprehensive Cancer Institute, Cedars-Sinai Medical Center, Los Angeles, CA USA; 610 Department of Biology, Bioinformatics Group, Division of Molecular Biology, Faculty of Science, University of Zagreb, Zagreb, Croatia; 611 Department for Internal Medicine II, University Hospital Schleswig-Holstein, Kiel, Germany; 612 Genetics and Molecular Pathology, SA Pathology, Adelaide, SA Australia; 613 Department of Gastric Surgery, National Cancer Center Hospital, Tokyo, Japan; 614 Department of Bioinformatics, Division of Cancer Genomics, National Cancer Center Research Institute, Tokyo, Japan; 615 A.A. Kharkevich Institute of Information Transmission Problems, Moscow, Russia; 616 Oncology and Immunology, Dmitry Rogachev National Research Center of Pediatric Hematology, Moscow, Russia; 617 Skolkovo Institute of Science and Technology, Moscow, Russia; 618 Department of Surgery, The George Washington University, School of Medicine and Health Science, Washington, DC USA; 619 Endocrine Oncology Branch, Center for Cancer Research, National Cancer Institute, National Institutes of Health, Bethesda, MD USA; 620 Melanoma Institute Australia, Macquarie University, Sydney, NSW Australia; 621 MIT Computer Science and Artificial Intelligence Laboratory, Massachusetts Institute of Technology, Cambridge, MA USA; 622 Tissue Pathology and Diagnostic Oncology, Royal Prince Alfred Hospital, Sydney, NSW Australia; 623 Cholangiocarcinoma Screening and Care Program and Liver Fluke and Cholangiocarcinoma Research Centre, Faculty of Medicine, Khon Kaen University, Khon Kaen, Thailand; 624 Controlled Department and Institution, New York, NY USA; 625 Englander Institute for Precision Medicine, Weill Cornell Medicine, New York, NY USA; 626 National Cancer Center, Gyeonggi, South Korea; 627 Department of Biochemistry, College of Medicine, Ewha Womans University, Seoul, South Korea; 628 Health Sciences Department of Biomedical Informatics, University of California San Diego, La Jolla, CA USA; 629 Research Core Center, National Cancer Centre Korea, Goyang-si, South Korea; 630 Department of Health Sciences and Technology, Sungkyunkwan University School of Medicine, Seoul, South Korea; 631 Samsung Genome Institute, Seoul, South Korea; 632 Breast Oncology Program, Dana-Farber/Brigham and Women’s Cancer Center, Boston, MA USA; 633 Department of Surgery, Memorial Sloan Kettering Cancer Center, New York, NY USA; 634 Division of Breast Surgery, Brigham and Women’s Hospital, Boston, MA USA; 635 Integrative Bioinformatics Support Group, National Institute of Environmental Health Sciences (NIEHS), Durham, NC USA; 636 Department of Clinical Science, University of Bergen, Bergen, Norway; 637 Center For Medical Innovation, Seoul National University Hospital, Seoul, South Korea; 638 Department of Internal Medicine, Seoul National University Hospital, Seoul, South Korea; 639 Institute of Computer Science, Polish Academy of Sciences, Warsawa, Poland; 640 Functional and Structural Genomics, German Cancer Research Center (DKFZ), Heidelberg, Germany; 641 Laboratory of Translational Genomics, Division of Cancer Epidemiology and Genetics, National Cancer Institute, , National Institutes of Health, Bethesda, MD USA; 642 Institute for Medical Informatics Statistics and Epidemiology, University of Leipzig, Leipzig, Germany; 643 Morgan Welch Inflammatory Breast Cancer Research Program and Clinic, The University of Texas MD Anderson Cancer Center, Houston, TX USA; 644 Department of Hematology and Oncology, Georg-Augusts-University of Göttingen, Göttingen, Germany; 645 Institute of Cell Biology (Cancer Research), University of Duisburg-Essen, Essen, Germany; 646 King’s College London and Guy’s and St. Thomas’ NHS Foundation Trust, London, UK; 647 Center for Epigenetics, Van Andel Research Institute, Grand Rapids, MI USA; 648 The University of Queensland Centre for Clinical Research, Royal Brisbane and Women’s Hospital, Herston, QLD Australia; 649 Department of Pediatric Oncology and Hematology, University of Cologne, Cologne, Germany; 650 University of Düsseldorf, Düsseldorf, Germany; 651 Department of Pathology, Institut Jules Bordet, Brussels, Belgium; 652 Institute of Biomedicine, Sahlgrenska Academy at University of Gothenburg, Gothenburg, Sweden; 653 Children’s Medical Research Institute, Sydney, NSW Australia; 654 ILSbio, LLC Biobank, Chestertown, MD USA; 655 Division of Genetics and Genomics, Boston Children’s Hospital, Harvard Medical School, Boston, MA USA; 656 Institute for Bioengineering and Biopharmaceutical Research (IBBR), Hanyang University, Seoul, South Korea; 657 Department of Statistics, University of California Santa Cruz, Santa Cruz, CA USA; 658 National Genotyping Center, Institute of Biomedical Sciences, Academia Sinica, Taipei, Taiwan; 659 Department of Vertebrate Genomics/Otto Warburg Laboratory Gene Regulation and Systems Biology of Cancer, Max Planck Institute for Molecular Genetics, Berlin, Germany; 660 McGill University and Genome Quebec Innovation Centre, Montreal, QC Canada; 661 biobyte solutions GmbH, Heidelberg, Germany; 662 Gynecologic Oncology, NYU Laura and Isaac Perlmutter Cancer Center, New York University, New York, NY USA; 663 Division of Oncology, Stem Cell Biology Section, Washington University School of Medicine, St. Louis, MO USA; 664 Department of Systems Biology, The University of Texas MD Anderson Cancer Center, Houston, TX USA; 665 Harvard University, Cambridge, MA USA; 666 Urologic Oncology Branch, Center for Cancer Research, National Cancer Institute, National Institutes of Health, Bethesda, MD USA; 667 University of Oslo, Oslo, Norway; 668 University of Toronto, Toronto, ON Canada; 669 Peking University, Beijing, China; 670 School of Life Sciences, Peking University, Beijing, China; 671 Leidos Biomedical Research, Inc, McLean, VA USA; 672 Hematology, Hospital Clinic, Institut d’Investigacions Biomèdiques August Pi i Sunyer (IDIBAPS), University of Barcelona, Barcelona, Spain; 673 Second Military Medical University, Shanghai, China; 674 Chinese Cancer Genome Consortium, Shenzhen, China; 675 Department of Medical Oncology, Beijing Hospital, Beijing, China; 676 Laboratory of Molecular Oncology, Key Laboratory of Carcinogenesis and Translational Research (Ministry of Education), Peking University Cancer Hospital and Institute, Beijing, China; 677 School of Medicine/School of Mathematics and Statistics, University of St. Andrews, St, Andrews, Fife UK; 678 Institute for Systems Biology, Seattle, WA USA; 679 Department of Biochemistry and Molecular Biology, Faculty of Medicine, University Institute of Oncology-IUOPA, Oviedo, Spain; 680 Institut Bergonié, Bordeaux, France; 681 Cancer Unit, MRC University of Cambridge, Cambridge, UK; 682 Department of Pathology and Laboratory Medicine, Center for Personalized Medicine, Children’s Hospital Los Angeles, Los Angeles, CA USA; 683 John Curtin School of Medical Research, Canberra, ACT Australia; 684 MVZ Department of Oncology, PraxisClinic am Johannisplatz, Leipzig, Germany; 685 Department of Information Technology, Ghent University, Ghent, Belgium; 686 Department of Plant Biotechnology and Bioinformatics, Ghent University, Ghent, Belgium; 687 Institute for Genomic Medicine, Nationwide Children’s Hospital, Columbus, OH USA; 688 Computational Biology Program, School of Medicine, Oregon Health and Science University, Portland, OR USA; 689 Department of Surgery, Duke University, Durham, NC USA; 690 Institució Catalana de Recerca i Estudis Avançats (ICREA), Barcelona, Spain; 691 Institut Català de Paleontologia Miquel Crusafont, Universitat Autònoma de Barcelona, Barcelona, Spain; 692 University of Glasgow, Glasgow, UK; 693 Institut d’Investigacions Biomèdiques August Pi i Sunyer (IDIBAPS), Barcelona, Spain; 694 Division of Oncology, Washington University School of Medicine, St. Louis, MO USA; 695 Department of Surgery and Cancer, Imperial College, London, INY UK; 696 Applications Department, Oxford Nanopore Technologies, Oxford, UK; 697 Department of Obstetrics, Gynecology and Reproductive Services, University of California San Francisco, San Francisco, CA USA; 698 Department of Biochemistry and Molecular Medicine, University California at Davis, Sacramento, CA USA; 699 STTARR Innovation Facility, Princess Margaret Cancer Centre, Toronto, ON Canada; 700 Discipline of Surgery, Western Sydney University, Penrith, NSW Australia; 701 Yale School of Medicine, Yale University, New Haven, CT USA; 702 Department of Genetics, Lineberger Comprehensive Cancer Center, University of North Carolina at Chapel Hill, Chapel Hill, NC USA; 703 Departments of Neurology and Neurosurgery, Henry Ford Hospital, Detroit, MI USA; 704 Precision Oncology, OHSU Knight Cancer Institute, Oregon Health and Science University, Portland, OR USA; 705 Institute of Pathology, University Medical Center Hamburg-Eppendorf, Hamburg, Germany; 706 Department of Health Sciences, Faculty of Medical Sciences, Kyushu University, Fukuoka, Japan; 707 Heidelberg Academy of Sciences and Humanities, Heidelberg, Germany; 708 Department of Clinical Pathology, University of Melbourne, Melbourne, VIC, Australia; 709 Department of Pathology, Roswell Park Cancer Institute, Buffalo, NY USA; 710 Department of Computer Science, University of Helsinki, Helsinki, Finland; 711 Institute of Biotechnology, University of Helsinki, Helsinki, Finland; 712 Organismal and Evolutionary Biology Research Programme, University of Helsinki, Helsinki, Finland; 713 Department of Obstetrics and Gynecology, Division of Gynecologic Oncology, Washington University School of Medicine, St. Louis, MO USA; 714 Penrose St. Francis Health Services, Colorado Springs, CO USA; 715 Institute of Pathology, Ulm University and University Hospital of Ulm, Ulm, Germany; 716 National Cancer Center, Tokyo, Japan; 717 Genome Institute of Singapore, Singapore, Singapore; 718 32Program in Computational Biology and Bioinformatics, Yale University, New Haven, CT USA; 719 German Cancer Aid, Bonn, Germany; 720 Programme in Cancer and Stem Cell Biology, Centre for Computational Biology, Duke-NUS Medical School, Singapore, Singapore; 721 The Chinese University of Hong Kong, Shatin, NT, Hong Kong China; 722 Fourth Military Medical University, Shaanxi, China; 723 The University of Cambridge School of Clinical Medicine, Cambridge, UK; 724 St. Jude Children’s Research Hospital, Memphis, TN USA; 725 University Health Network, Princess Margaret Cancer Centre, Toronto, ON Canada; 726 Center for Biomolecular Science and Engineering, University of California Santa Cruz, Santa Cruz, CA USA; 727 Department of Medicine, University of Chicago, Chicago, IL USA; 728 Department of Neurology, Mayo Clinic, Rochester, MN USA; 729 Cambridge Oesophagogastric Centre, Cambridge University Hospitals NHS Foundation Trust, Cambridge, UK; 730 Department of Computer Science, Carleton College, Northfield, MN USA; 731 Institute of Cancer Sciences, College of Medical Veterinary and Life Sciences, University of Glasgow, Glasgow, UK; 732 Department of Epidemiology, University of Alabama at Birmingham, Birmingham, AL USA; 733 HudsonAlpha Institute for Biotechnology, Huntsville, AL USA; 734 O’Neal Comprehensive Cancer Center, University of Alabama at Birmingham, Birmingham, AL USA; 735 Department of Pathology, Keio University School of Medicine, Tokyo, Japan; 736 Department of Hepatobiliary and Pancreatic Oncology, National Cancer Center Hospital, Tokyo, Japan; 737 Sage Bionetworks, Seattle, WA USA; 738 Lymphoma Genomic Translational Research Laboratory, National Cancer Centre, Singapore, Singapore; 739 Department of Clinical Pathology, Robert-Bosch-Hospital, Stuttgart, Germany; 740 Department of Cell and Systems Biology, University of Toronto, Toronto, ON Canada; 741 Department of Biosciences and Nutrition, Karolinska Institutet, Stockholm, Sweden; 742 Center for Liver Cancer, Research Institute and Hospital, National Cancer Center, Gyeonggi, South Korea; 743 Division of Hematology-Oncology, Samsung Medical Center, Sungkyunkwan University School of Medicine, Seoul, South Korea; 744 Samsung Advanced Institute for Health Sciences and Technology, Sungkyunkwan University School of Medicine, Seoul, South Korea; 745 Cheonan Industry-Academic Collaboration Foundation, Sangmyung University, Cheonan, South Korea; 746 NYU Langone Medical Center, New York, NY USA; 747 Department of Hematology and Medical Oncology, Cleveland Clinic, Cleveland, OH USA; 748 Department of Radiation Oncology, University of California San Francisco, San Francisco, CA USA; 749 Department of Health Sciences Research, Mayo Clinic, Rochester, MN USA; 750 Helen F. Graham Cancer Center at Christiana Care Health Systems, Newark, DE USA; 751 Heidelberg University Hospital, Heidelberg, Germany; 752 CSRA Incorporated, Fairfax, VA USA; 753 Research Department of Pathology, University College London Cancer Institute, London, UK; 754 Department of Research Oncology, Guy’s Hospital, King’s Health Partners AHSC, King’s College London School of Medicine, London, UK; 755 Faculty of Medicine and Health Sciences, Macquarie University, Sydney, NSW Australia; 756 University Hospital of Minjoz, INSERM UMR 1098, Besançon, France; 757 Spanish National Cancer Research Centre, Madrid, Spain; 758 Center of Digestive Diseases and Liver Transplantation, Fundeni Clinical Institute, Bucharest, Romania; 759 Cureline, Inc, South San Francisco, CA USA; 760 St. Luke’s Cancer Centre, Royal Surrey County Hospital NHS Foundation Trust, Guildford, UK; 761 Cambridge Breast Unit, Addenbrooke’s Hospital, Cambridge University Hospital NHS Foundation Trust and NIHR Cambridge Biomedical Research Centre, Cambridge, UK; 762 East of Scotland Breast Service, Ninewells Hospital, Aberdeen, UK; 763 Department of Genetics, Microbiology and Statistics, University of Barcelona, IRSJD, IBUB, Barcelona, Spain; 764 Department of Obstetrics and Gynecology, Medical College of Wisconsin, Milwaukee, WI USA; 765 Hematology and Medical Oncology, Winship Cancer Institute of Emory University, Atlanta, GA USA; 766 Department of Computer Science, Princeton University, Princeton, NJ USA; 767 Vanderbilt Ingram Cancer Center, Vanderbilt University, Nashville, TN USA; 768 Ohio State University College of Medicine and Arthur G. James Comprehensive Cancer Center, Columbus, OH USA; 769 Department of Surgery, Yokohama City University Graduate School of Medicine, Kanagawa, Japan; 770 Division of Chromatin Networks, German Cancer Research Center (DKFZ) and BioQuant, Heidelberg, Germany; 771 Research Computing Center, University of North Carolina at Chapel Hill, Chapel Hill, NC USA; 772 School of Molecular Biosciences and Center for Reproductive Biology, Washington State University, Pullman, WA USA; 773 Finsen Laboratory and Biotech Research and Innovation Centre (BRIC), University of Copenhagen, Copenhagen, Denmark; 774 Department of Laboratory Medicine and Pathobiology, University of Toronto, Toronto, ON Canada; 775 Department of Pathology, Human Oncology and Pathogenesis Program, Memorial Sloan Kettering Cancer Center, New York, NY USA; 776 University Hospital Giessen, Pediatric Hematology and Oncology, Giessen, Germany; 777 Oncologie Sénologie, ICM Institut Régional du Cancer, Montpellier, France; 778 Institute of Clinical Molecular Biology, Christian-Albrechts-University, Kiel, Germany; 779 Institute of Pathology, University of Wuerzburg, Wuerzburg, Germany; 780 Department of Urology, North Bristol NHS Trust, Bristol, UK; 781 SingHealth, Duke-NUS Institute of Precision Medicine, National Heart Centre Singapore, Singapore, Singapore; 782 Department of Computer Science, University of Toronto, Toronto, ON Canada; 783 Bern Center for Precision Medicine, University Hospital of Bern, University of Bern, Bern, Switzerland; 784 Englander Institute for Precision Medicine, Weill Cornell Medicine and New York Presbyterian Hospital, New York, NY USA; 785 Meyer Cancer Center, Weill Cornell Medicine, New York, NY USA; 786 Pathology and Laboratory, Weill Cornell Medical College, New York, NY USA; 787 Vall d’Hebron Institute of Oncology: VHIO, Barcelona, Spain; 788 General and Hepatobiliary-Biliary Surgery, Pancreas Institute, University and Hospital Trust of Verona, Verona, Italy; 789 National Centre for Biological Sciences, Tata Institute of Fundamental Research, Bangalore, India; 790 Indiana University, Bloomington, IN USA; 791 Department of Pathology, GZA-ZNA Hospitals, Antwerp, Belgium; 792 Analytical Biological Services, Inc, Wilmington, DE USA; 793 Sydney Medical School, University of Sydney, Sydney, NSW Australia; 794 cBio Center, Dana-Farber Cancer Institute, Harvard Medical School, Boston, MA USA; 795 Department of Cell Biology, Harvard Medical School, Boston, MA USA; 796 Advanced Centre for Treatment Research and Education in Cancer, Tata Memorial Centre, Navi Mumbai, Maharashtra India; 797 School of Environmental and Life Sciences, Faculty of Science, The University of Newcastle, Ourimbah, NSW Australia; 798 Department of Dermatology, University Hospital of Essen, Essen, Germany; 799 Bioinformatics and Omics Data Analytics, German Cancer Research Center (DKFZ), Heidelberg, Germany; 800 Department of Urology, Charité Universitätsmedizin Berlin, Berlin, Germany; 801 Martini-Clinic, Prostate Cancer Center, University Medical Center Hamburg-Eppendorf, Hamburg, Germany; 802 Department of General Internal Medicine, University of Kiel, Kiel, Germany; 803 German Cancer Consortium (DKTK), Partner site Berlin, Berlin, Germany; 804 Cancer Research Institute, Beth Israel Deaconess Medical Center, Boston, MA USA; 805 University of Pittsburgh, Pittsburgh, PA USA; 806 Department of Ophthalmology and Ocular Genomics Institute, Massachusetts Eye and Ear, Harvard Medical School, Boston, MA USA; 807 Center for Psychiatric Genetics, NorthShore University HealthSystem, Evanston, IL USA; 808 Van Andel Research Institute, Grand Rapids, MI USA; 809 Laboratory of Molecular Medicine, Human Genome Center, Institute of Medical Science, University of Tokyo, Tokyo, Japan; 810 Japan Agency for Medical Research and Development, Tokyo, Japan; 811 Korea University, Seoul, South Korea; 812 Murtha Cancer Center, Walter Reed National Military Medical Center, Bethesda, MD USA; 813 Human Genetics, University of Kiel, Kiel, Germany; 814 Department of Oncologic Pathology, Dana-Farber Cancer Institute, Harvard Medical School, Boston, MA USA; 815 Oregon Health and Science University, Portland, OR USA; 816 Center for RNA Interference and Noncoding RNA, The University of Texas MD Anderson Cancer Center, Houston, TX USA; 817 Department of Experimental Therapeutics, The University of Texas MD Anderson Cancer Center, Houston, TX USA; 818 Department of Gynecologic Oncology and Reproductive Medicine, The University of Texas MD Anderson Cancer Center, Houston, TX USA; 819 University Hospitals Coventry and Warwickshire NHS Trust, Coventry, UK; 820 Department of Radiation Oncology, Radboud University Nijmegen Medical Centre, Nijmegen, GA The Netherlands; 821 Institute for Genomics and Systems Biology, University of Chicago, Chicago, IL USA; 822 Clinic for Hematology and Oncology, St.-Antonius-Hospital, Eschweiler, Germany; 823 Computational and Systems Biology Program, Memorial Sloan Kettering Cancer Center, New York, NY USA; 824 University of Iceland, Reykjavik, Iceland; 825 Division of Computational Genomics and Systems Genetics, German Cancer Research Center (DKFZ), Heidelberg, Germany; 826 Dundee Cancer Centre, Ninewells Hospital, Dundee, UK; 827 Department for Internal Medicine III, University of Ulm and University Hospital of Ulm, Ulm, Germany; 828 Institut Curie, INSERM Unit 830, Paris, France; 829 Department of Gastroenterology and Hepatology, Yokohama City University Graduate School of Medicine, Kanagawa, Japan; 830 Department of Laboratory Medicine, Radboud University Nijmegen Medical Centre, Nijmegen, GA The Netherlands; 831 Division of Cancer Genome Research, German Cancer Research Center (DKFZ), Heidelberg, Germany; 832 Department of General Surgery, Singapore General Hospital, Singapore, Singapore; 833 Cancer Science Institute of Singapore, National University of Singapore, Singapore, Singapore; 834 Department of Medical and Clinical Genetics, Genome-Scale Biology Research Program, University of Helsinki, Helsinki, Finland; 835 East Anglian Medical Genetics Service, Cambridge University Hospitals NHS Foundation Trust, Cambridge, UK; 836 Irving Institute for Cancer Dynamics, Columbia University, New York, NY USA; 837 Institute of Molecular and Cell Biology, Singapore, Singapore; 838 Laboratory of Cancer Epigenome, Division of Medical Science, National Cancer Centre Singapore, Singapore, Singapore; 839 Universite Lyon, INCa-Synergie, Centre Léon Bérard, Lyon, France; 840 Department of Urology, Mayo Clinic, Rochester, MN USA; 841 Royal National Orthopaedic Hospital - Stanmore, Stanmore, Middlesex UK; 842 Department of Biochemistry, Genetics and Immunology, University of Vigo, Vigo, Spain; 843 Giovanni Paolo II / I.R.C.C.S. Cancer Institute, Bari, BA Italy; 844 Neuroblastoma Genomics, German Cancer Research Center (DKFZ), Heidelberg, Germany; 845 Fondazione Policlinico Universitario Gemelli IRCCS, Rome, Italy, Rome, Italy; 846 University of Verona, Verona, Italy; 847 Centre National de Génotypage, CEA - Institute de Génomique, Evry, France; 848 CAPHRI Research School, Maastricht University, Maastricht, ER The Netherlands; 849 Department of Biopathology, Centre Léon Bérard, Lyon, France; 850 Université Claude Bernard Lyon 1, Villeurbanne, France; 851 Core Research for Evolutional Science and Technology (CREST), JST, Tokyo, Japan; 852 Department of Biological Sciences, Laboratory for Medical Science Mathematics, Graduate School of Science, University of Tokyo, Yokohama, Japan; 853 Department of Medical Science Mathematics, Medical Research Institute, Tokyo Medical and Dental University (TMDU), Tokyo, Japan; 854 Cancer Ageing and Somatic Mutation Programme, Wellcome Sanger Institute, Hinxton, UK; 855 University Hospitals Birmingham NHS Foundation Trust, Birmingham, UK; 856 Centre for Cancer Research and Cell Biology, Queen’s University, Belfast, UK; 857 Breast Medical Oncology, The University of Texas MD Anderson Cancer Center, Houston, TX USA; 858 Department of Surgery, Johns Hopkins University School of Medicine, Baltimore, MD USA; 859 Department of Oncology-Pathology, Science for Life Laboratory, Karolinska Institute, Stockholm, Sweden; 860 School of Cancer Sciences, Faculty of Medicine, University of Southampton, Southampton, UK; 861 Department of Gene Technology, Tallinn University of Technology, Tallinn, Estonia; 862 Genetics and Genome Biology Program, SickKids Research Institute, The Hospital for Sick Children, Toronto, ON Canada; 863 Departments of Neurosurgery and Hematology and Medical Oncology, Winship Cancer Institute and School of Medicine, Emory University, Atlanta, GA USA; 864 Department of Clinical and Molecular Medicine, Faculty of Medicine and Health Sciences, Norwegian University of Science and Technology, Trondheim, Norway; 865 Argmix Consulting, North Vancouver, BC Canada; 866 Department of Information Technology, Ghent University, Interuniversitair Micro-Electronica Centrum (IMEC), Ghent, Belgium; 867 Nuffield Department of Surgical Sciences, John Radcliffe Hospital, University of Oxford, Oxford, UK; 868 Institute of Mathematics and Computer Science, University of Latvia, Riga, LV Latvia; 869 Discipline of Pathology, Sydney Medical School, University of Sydney, Sydney, NSW Australia; 870 Department of Applied Mathematics and Theoretical Physics, Centre for Mathematical Sciences, University of Cambridge, Cambridge, UK; 871 Department of Epidemiology and Biostatistics, Memorial Sloan Kettering Cancer Center, New York, NY USA; 872 Department of Statistics, Columbia University, New York, NY USA; 873 Department of Immunology, Genetics and Pathology, Science for Life Laboratory, Uppsala University, Uppsala, Sweden; 874 School of Electronic and Information Engineering, Xi’an Jiaotong University, Xi’an, China; 875 Department of Histopathology, Cambridge University Hospitals NHS Foundation Trust, Cambridge, UK; 876 Oxford NIHR Biomedical Research Centre, University of Oxford, Oxford, UK; 877 Georgia Regents University Cancer Center, Augusta, GA USA; 878 Wythenshawe Hospital, Manchester, UK; 879 Department of Genetics, Washington University School of Medicine, St.Louis, MO USA; 880 Department of Biological Oceanography, Leibniz Institute of Baltic Sea Research, Rostock, Germany; 881 Wellcome Centre for Human Genetics, University of Oxford, Oxford, UK; 882 Department of Molecular and Human Genetics, Baylor College of Medicine, Houston, TX USA; 883 Thoracic Oncology Laboratory, Mayo Clinic, Rochester, MN USA; 884 Institute for Genomic Medicine, Nationwide Children’s Hospital, Columbus, OH USA; 885 Department of Obstetrics and Gynecology, Division of Gynecologic Oncology, Mayo Clinic, Rochester, MN USA; 886 International Institute for Molecular Oncology, Poznań, Poland; 887 Poznan University of Medical Sciences, Poznań, Poland; 888 Genomics and Proteomics Core Facility High Throughput Sequencing Unit, German Cancer Research Center (DKFZ), Heidelberg, Germany; 889 NCCS-VARI Translational Research Laboratory, National Cancer Centre Singapore, Singapore, Singapore; 890 Edison Family Center for Genome Sciences and Systems Biology, Washington University, St. Louis, MO USA; 891 MRC-University of Glasgow Centre for Virus Research, Glasgow, UK; 892 Department of Medical Informatics and Clinical Epidemiology, Division of Bioinformatics and Computational Biology, OHSU Knight Cancer Institute, Oregon Health and Science University, Portland, OR USA; 893 School of Electronic Information and Communications, Huazhong University of Science and Technology, Wuhan, China; 894 Department of Applied Mathematics and Statistics, Johns Hopkins University, Baltimore, MD USA; 895 Department of Cancer Genome Informatics, Graduate School of Medicine, Osaka University, Osaka, Japan; 896 Institute of Computer Science, Heidelberg University, Heidelberg, Germany; 897 School of Mathematics and Statistics, University of Sydney, Sydney, NSW Australia; 898 Ben May Department for Cancer Research, University of Chicago, Chicago, IL USA; 899 Department of Human Genetics, University of Chicago, Chicago, IL USA; 900 Tri-Institutional PhD Program in Computational Biology and Medicine, Weill Cornell Medicine, New York, NY USA; 901 The First Affiliated Hospital, Xi’an Jiaotong University, Xi’an, China; 902 Department of Medicine and Therapeutics, The Chinese University of Hong Kong, Shatin, NT, Hong Kong China; 903 Department of Biostatistics, The University of Texas MD Anderson Cancer Center, Houston, TX USA; 904 Duke-NUS Medical School, Singapore, Singapore; 905 Department of Surgery, Ruijin Hospital, Shanghai Jiaotong University School of Medicine, Shanghai, China; 906 School of Computing Science, University of Glasgow, Glasgow, UK; 907 Division of Orthopaedic Surgery, Oslo University Hospital, Oslo, Norway; 908 Eastern Clinical School, Monash University, Melbourne, VIC Australia; 909 Epworth HealthCare, Richmond, VIC Australia; 910 Department of Biostatistics and Computational Biology, Dana-Farber Cancer Institute and Harvard Medical School, Boston, MA USA; 911 Department of Biomedical Informatics, College of Medicine, The Ohio State University, Columbus, OH USA; 912 The Ohio State University Comprehensive Cancer Center (OSUCCC – James), Columbus, OH USA; 913 The University of Texas School of Biomedical Informatics (SBMI) at Houston, Houston, TX USA; 914 Department of Biostatistics, University of North Carolina at Chapel Hill, Chapel Hill, NC USA; 915 Department of Biochemistry and Molecular Genetics, Feinberg School of Medicine, Northwestern University, Chicago, IL USA; 916 Faculty of Medicine and Health, University of Sydney, Sydney, NSW Australia; 917 Department of Pathology, Erasmus Medical Center Rotterdam, Rotterdam, GD The Netherlands; 918 Division of Molecular Carcinogenesis, The Netherlands Cancer Institute, Amsterdam, CX The Netherlands; 919 Institute of Molecular Life Sciences and Swiss Institute of Bioinformatics, University of Zurich, Zurich, Switzerland

**Keywords:** Cancer genomics, Genomic instability

## Abstract

A key mutational process in cancer is structural variation, in which rearrangements delete, amplify or reorder genomic segments that range in size from kilobases to whole chromosomes^1–7^. Here we develop methods to group, classify and describe somatic structural variants, using data from the Pan-Cancer Analysis of Whole Genomes (PCAWG) Consortium of the International Cancer Genome Consortium (ICGC) and The Cancer Genome Atlas (TCGA), which aggregated whole-genome sequencing data from 2,658 cancers across 38 tumour types^8^. Sixteen signatures of structural variation emerged. Deletions have a multimodal size distribution, assort unevenly across tumour types and patients, are enriched in late-replicating regions and correlate with inversions. Tandem duplications also have a multimodal size distribution, but are enriched in early-replicating regions—as are unbalanced translocations. Replication-based mechanisms of rearrangement generate varied chromosomal structures with low-level copy-number gains and frequent inverted rearrangements. One prominent structure consists of 2–7 templates copied from distinct regions of the genome strung together within one locus. Such cycles of templated insertions correlate with tandem duplications, and—in liver cancer—frequently activate the telomerase gene *TERT*. A wide variety of rearrangement processes are active in cancer, which generate complex configurations of the genome upon which selection can act.

## Abstract (web summary)

Whole-genome sequencing data from more than 2,500 cancers of 38 tumour types reveal 16 signatures that can be used to classify somatic structural variants, highlighting the diversity of genomic rearrangements in cancer.

## Main

Mutations that arise in somatic cells are the driving force of cancer development. Structural variation—in which genomic rearrangement acts to amplify, delete or reorder chromosomal material at scales that range from single genes to entire chromosomes—is an especially important class of somatic mutation. Previous analyses of both cancer and germline genomes have enabled the description of several distinctive patterns of structural variants^1–7^, and hypotheses about the underlying basis of several of these patterns have been proposed on the basis of their clustering, orientation and associated copy-number changes. Hypothesis-driven in vitro studies are now beginning to reveal some of the mechanistic processes that generate these structures^9–13^, and generate further predictions that can be assessed in the genomic data. However, the landscape of structural variation in human cancer remains incompletely mapped and there are many complex structures that elude formal description.

The PCAWG Consortium aggregated whole-genome sequencing data from 2,658 cancers across 38 tumour types, generated by the ICGC and TCGA projects. These sequencing data were aligned to the human genome (reference build hs37d5) and analysed with standardized, high-accuracy pipelines to call somatic and germline variants of all classes^8^. Here, we analyse the patterns and signatures of structural variants across the PCAWG data. We propose a working classification scheme that encompasses known and newly identified classes of structural variants. We develop methods for annotating the observed structural variants in a given cancer genome, identifying a class of replication-based rearrangement processes that generate clusters of several structural variants. We explore the size, activity and genome-wide distribution of classifiable structural variant types across the cohort, using signature analysis to define how they correlate within patients. Other papers produced by PCAWG address complementary aspects of structural variants, including inference of positive selection acting on recurrently rearranged regions of the genome^14^, how structural variants affect the transcriptome^15^ and chromosome topology^16^, patterns of somatic retrotransposition^17^ and distribution of chromothripsis across cancer types^18^.

## Classification of structural variants

**Fig. 1:**
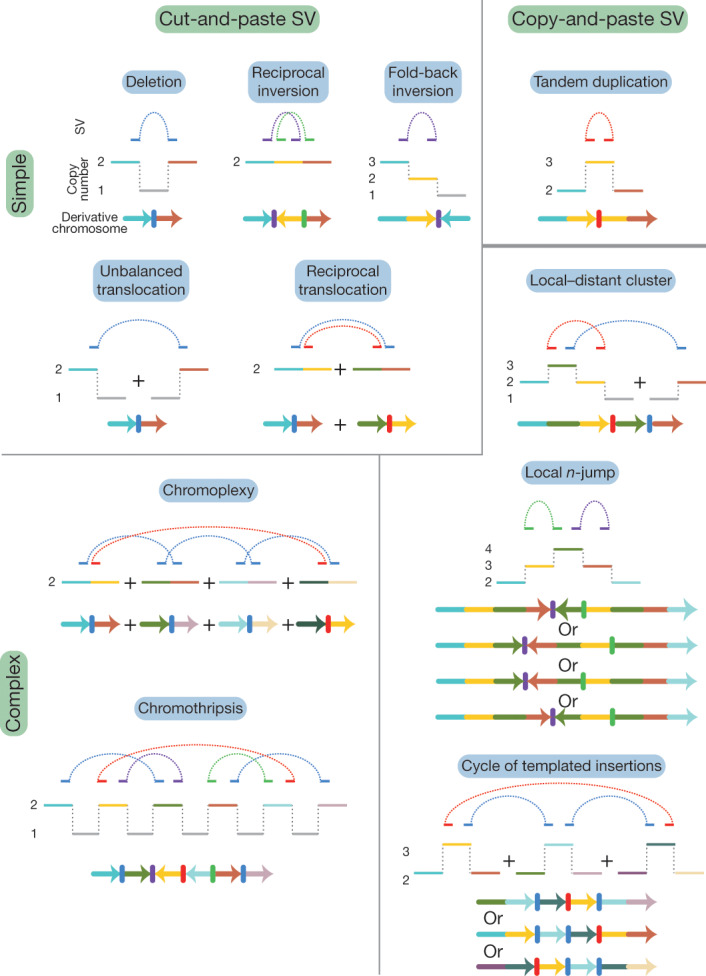
Classification of structural variants in cancer genomes. Schematics of major structural-variant (SV) classes, grouped according to whether they are simple or complex and arise through cut-and-paste or copy-and-paste processes. Each schematic comprises three parts. The top segment shows dotted arcs for each rearrangement junction that joins two chromosomal segments together. The middle segment shows the copy number of genomic segments that are involved. The bottom segment shows the configuration of the final derivative chromosome that results from the structural variant; the colour of the segments corresponds to the colour of that segment in the copy-number schematic. + indicates the different derivative chromosomes created for some of the classes: that is, the structural variants are not phased to a single derivative.

A ‘structural variant’ manifests as a ‘junction’ between two ‘breakpoints’ in the genome (terms in inverted commas here and below refer to those defined in the glossary in Extended Data Table 1). Generally, there will be a change in copy number across a given breakpoint if only one side of the break is rescued by a structural variant; if both sides of a double-stranded DNA break are rescued, a ‘reciprocal’ or ‘balanced’ structural variant will result, without substantial copy-number change. We sometimes observe ‘clusters of structural variants’ in which several breakpoints occur close together, in time or in genomic space—usually both. Such spatial and/or temporal proximity generally, but not always, implies that the structural variants within a cluster are mechanistically linked. Clusters can be ‘phased’ (in which case all structural variants in the cluster resolve to a single derivative chromosome) or ‘unphased’, in which case the structural variants are carried on different derivative chromosomes. An example of the latter is a reciprocal translocation that results in two derivative chromosomes, each with a single interchromosomal breakpoint junction (Fig. 1).

We recognize distinct ‘classes of structural variant’ from the orientation of the two segments at the junction and associated copy-number changes (Fig. 1, Supplementary Fig. 1). Some classes of structural variant (such as isochromosomes and rearrangements between extended, highly homologous sequences) are difficult to detect with short-read sequencing data; these classes are not considered further here. We propose categorizing classes of structural variant across two facets: the number of breakpoints involved (simple or complex) and by whether the patterns are likely to arise from ‘cut-and-paste’ or ‘copy-and-paste’ rearrangement processes. A cut-and-paste process generates a cluster of structural variants consistent with reshuffling or loss of extant genomic segments, and a copy-and-paste process is one in which copies of genomic ‘templates’ are newly replicated or synthesized and inserted during the rearrangement process. Deletions, reciprocal inversions, unbalanced translocations and reciprocal translocations are examples of simple cut-and-paste structural variants, as they can be reconstructed from the incorrect religation of chromosomal breaks. Tandem duplications are simple copy-and-paste structural variants, as they arise through the local insertion of a newly generated extra copy of a genomic template.

More-complex cut-and-paste processes that produce structural variants also occur in cancer. ‘Breakage–fusion–bridge’ events result from cycles of DNA breakage, end-to-end sister chromatid fusions, mitotic bridges and further DNA breakage. These events manifest as one or a few proximate, inverted breakpoint junctions with associated copy-number change, which we call ‘fold-back inversions’^1,2,19^ (Fig. 1). ‘Chromoplexy’^5,20^—which is particularly frequent in prostate cancers—results from several simultaneous double-stranded DNA breaks in several chromosomes that are rejoined incorrectly, leading to balanced chains of rearrangements. ‘Chromothripsis’^3^, in which chromosome shattering and rearrangement occur in a single catastrophic event^9,21^, leads to a pattern of oscillating copy-number changes and localized clustering of tens to hundreds of breakpoints^22^.

In the germline, more-complex copy-and-paste classes of structural variant have previously been described, which involve small duplications and triplications and are thought to arise from the stalling of the replication fork leading to template switching^4,23,24^. Here we describe a wide range of complex copy-and-paste types of somatic structural variant that occur in human cancers, and that are typically characterized by copy-number gains and frequent inverted rearrangements.

## Annotation of structural-variant classes

We analysed 2,559 whole cancer genomes across 38 tumour types (alongside matched germline DNA) that passed the most stringent PCAWG quality-control criteria: 1 or more somatic structural variants were detected in 2,429 tumours^8^. As described in an accompanying Article^8^, structural variants were identified using aberrantly mapping and/or split reads in paired-end sequencing data^25^. We used four somatic structural-variant callers^20,25–27^, and the final structural-variant dataset comprised events that were returned by ≥2 callers, merged by a graph-based consensus method^8^. We consider only somatically acquired structural variants in this analysis, and exclude somatic retrotransposition events. Validation of structural-variant calls was undertaken using both manual inspection and pull-down with resequencing of breakpoints. With these approaches, we estimate the sensitivity of the consensus structural-variant call set to be 90% for true calls generated by any 1 of the 4 callers; specificity was estimated as 97.5%^8^. A mean of 3.22 algorithms of the 4 that we used called each structural variant in the consensus set genome-wide, and this differed little across repetitive elements: the mean for short interspersed nuclear elements was 3.22, and the mean for long interspersed nuclear elements was 3.21.

Because the structural variants from a given cancer are often highly clustered, we grouped rearrangements into clusters on the basis of the proximity of breakpoints, the overall number of events in that genome and the size distribution of these events (Supplementary Methods). Essentially, a particular cluster contains structural variants that are significantly closer together than expected by chance, given the overall number and orientation of structural variants in that patient. Alongside the clustering, we computed an in silico library of all possible genomic configurations that result from sequential simple structural variants (deletions, tandem duplications, inversions, translocations, and chromosome duplications or losses), to a depth of five rearrangements. We could then compare the genomic configuration of each observed cluster of structural variants against the library to determine how it might have arisen.

**Fig. 2:**
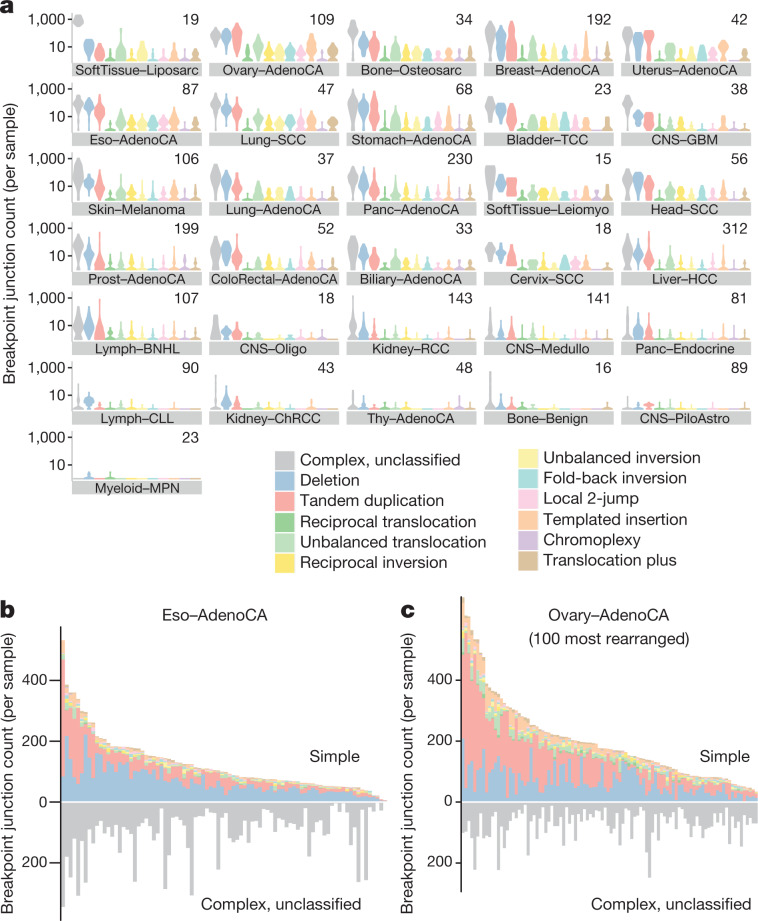
Frequency of structural-variant classes across tumour types. **a**, Violin plots of density of classified structural-variant categories across patients within each histology group. Tumour type panels are sorted in descending order of the average number of structural-variant breakpoints per sample. Within each tumour type, the frequency distribution (*y* axis) of different structural-variant categories (*x* axis) across patients is shown as a density: regions of highest density have the greatest width of shaded area. In each panel, the number of patients is indicated at the top right. AdenoCA, adenocarcinoma; BNHL, B-cell non-Hodgkin lymphoma; ChRCC, chromophobe renal cell carcinoma; CLL, chronic lymphocytic leukaemia; CNS, central nervous system; GBM, glioblastoma; HCC, hepatocellular carcinoma; leiomyo, leiomyosarcoma; medullo, medulloblastoma; MPN, myeloproliferative neoplasm; eso, oesophageal; oligo, oligodendrocytic; panc, pancreatic; piloastro, pilocytic astrocytoma; prost, prostate; RCC, renal cell carcinoma; sarc, sarcoma; SCC, squamous cell carcinoma; TCC, transitional cell carcinoma; thy, thyroid. **b**, Per-sample counts of complex (bottom) and classified (top) structural-variant breakpoint junctions for oesophageal adenocarcinoma. **c**, Per-sample counts of complex (bottom) and classified (top) structural-variant breakpoint junctions for ovarian adenocarcinoma.

This methodology has the advantage that breakpoint junctions are classified according to the wider genomic context in which they occur. This means that, for example, true deletions will be identifiably different from breakpoint junctions that happen to have a deletion-type orientation but arise within (for instance) a chromothripsis event of markedly different mechanism and properties. Over half the breakpoint junctions that we observed arise within clusters of several or many structural variants (Fig. 2a): removing these junctions from the catalogues of true deletions, tandem duplications and inversions enables a more-precise description of the properties of simple structural variants.

**Extended Data Fig. 1:**
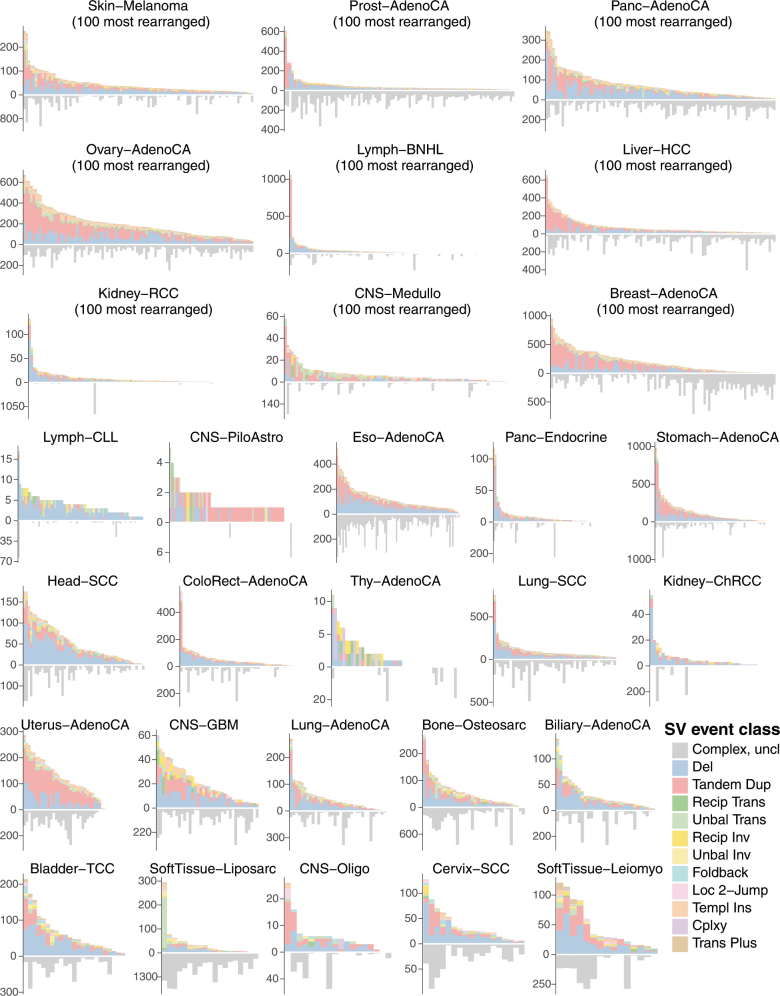
Per-sample counts of structural-variant breakpoint junctions by histology group. Counts of simple, classified structural variants are shown above the *x* axis and counts of complex breakpoint junctions below the *x* axis. Patients within each tumour type are ranked by frequency of simple structural variants.

Among the classes of simple structural variants, deletion was the most common, followed by tandem duplication and then unbalanced translocation. Reciprocal translocations and reciprocal inversions were uncommon events (Fig. 2a). There was considerable variability in the overall numbers and distribution of classes of structural variant across tumour types and across patients within a given tumour type (Extended Data Fig. 1). For example, oesophageal adenocarcinomas were characterized by many deletions and a large number of complex clustered rearrangements (Fig. 2b), and ovarian cancers often carried high numbers of tandem duplications and/or deletions with moderate numbers of unbalanced translocations (Fig. 2c).

## Cycles of templated insertions

**Fig. 3:**
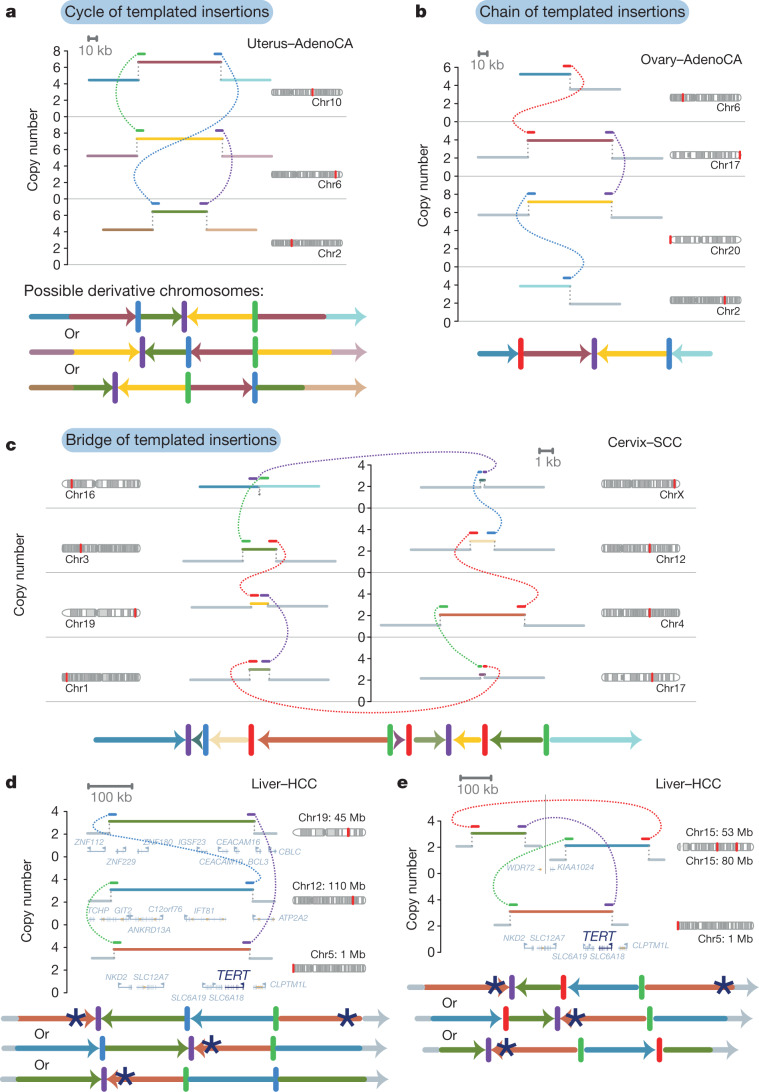
Chains, cycles and bridges of templated insertions. **a**–**c**, Examples of a typical cycle (**a**), chain (**b**) and bridge (**c**) of templated insertions. The estimated copy-number profile is shown as in Fig. 1, with structural variants shown as dotted arcs linking two copy-number segments. The derivative chromosome(s) that could explain the copy-number and structural-variant profile is shown below. **d**, **e**, Cycles of templated insertions that affect the *TERT* gene, in two hepatocellular carcinomas. *KIAA1024* is also known as *MINAR1*.

**Extended Data Fig. 2:**
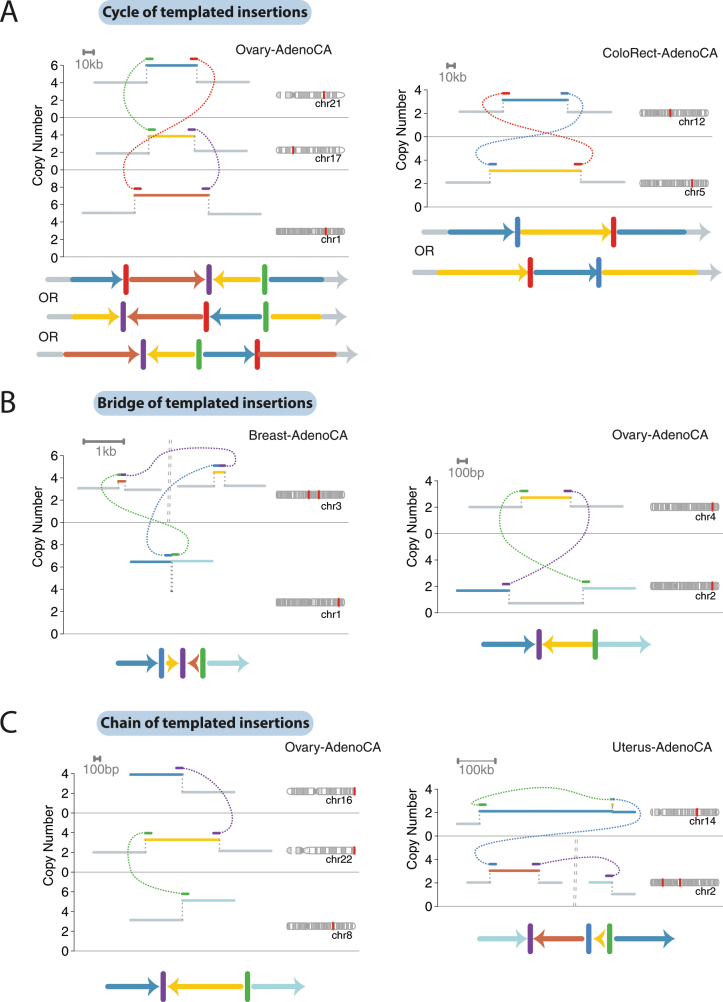
Further examples of templated insertion chains, cycles and bridges. Schematics follow the same structure as in Fig. 3.

We next examined clusters that contain 2–10 structural variants. One newly identified configuration consisted of several segments of copy-number gains, typically on different reference chromosomes, linked together through structural variants (Fig. 3, Extended Data Fig. 2). A sequential path through consecutive segments can be formed by following the breakpoint junctions, which suggests that each cluster represents a string of duplicated templates inserted into a single derivative chromosome, probably acquired concurrently. Although it is theoretically possible that the structural variants in such clusters are not phased on the same derivative chromosome or do not occur concurrently, we think this is unlikely for several reasons. First, we found examples of RNA transcripts that spliced together exons separated by two junctions in the structural-variant cluster (Supplementary Fig. 2), which suggests that they are phased on the same derivative chromosome. Second, long-read sequencing data (reported in an accompanying Article^8^) supported the phasing of structural variants that link templated insertions. Third, we found that the clonal fraction of tumour cells tended to be more similar for structural variants within these clusters than for randomly chosen structural variants in each patient (Supplementary Fig. 3), which suggests that they co-occur in evolutionary time. Fourth, the level of copy-number gain for individual segments in the cluster tended to be identical (Fig. 3, Extended Data Fig. 2).

We define three basic categories on the basis of whether or not the string of inserted segments returns to the original chromosome: we term strings of inserted segments that do not return ‘chains’ of templated insertions and those strings that do return ‘bridges’ (which leave a gap on the host chromosome) or ‘cycles’ (which rereplicate a segment on the host chromosome). In the PCAWG dataset overall, we observed 1,467 cycles and 1,275 bridges of templated insertions (Fig. 3a, b, Extended Data Fig. 2). In chains of templated insertions, the string of genomic segments does not return to the chromosome of departure (Fig. 3c, Extended Data Fig. 2) but it is similarly associated with copy-number gains at each templated segment. There were 285 instances of such chains in the dataset, commonly manifesting as unbalanced translocations joined through one or more intermediary templated insertions.

**Extended Data Fig. 3:**
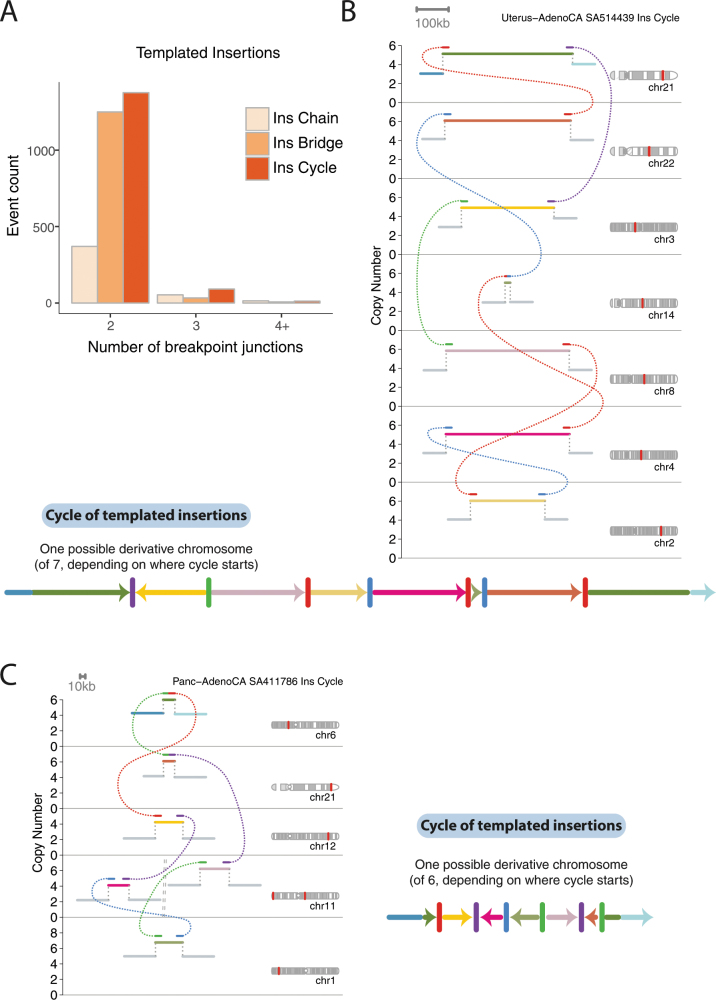
Number of breakpoint junctions in cycles, bridges and chains of templated insertions. **a**, Histogram of numbers of breakpoint junctions in templated insertion cycles, chains and bridges across all samples in all tumour types in the cohort. **b**, **c**, Two examples of particularly long cycles of templated insertions in the cohort. Examples are depicted in a similar manner to those in Fig. 3.

Most templated insertion events involve only two breakpoint junctions, but this can extend to three, four or more linked rearrangements (Extended Data Fig. 3a). The longest such event—from a cervical squamous cell cancer—had seven templated insertions strung together on an eighth host chromosome (Fig. 3c; other examples of long templated insertion events are shown in Extended Data Fig. 3).

## Templated insertions that affect *TERT*

**Extended Data Fig. 4:**
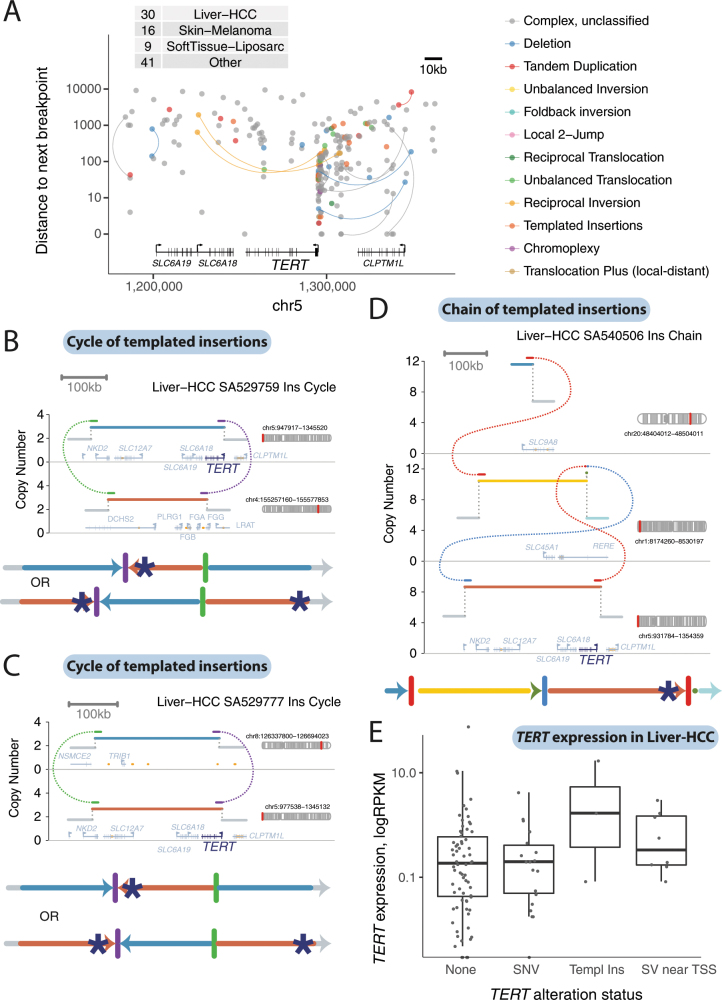
Templated insertion events that activate *TERT* in hepatocellular carcinoma. **a**, The positions of all structural-variant breakpoints in the *TERT* region in the PCAWG cohort (including 50-kb flanks either side of *TERT*), coloured by classification and vertically spaced by the distance to the next breakpoint in the cohort. If the two sides of a breakpoint junction are contained within the plotting window, they are joined by a curved line. The number of samples with a breakpoint in the plotting window is annotated in the table in the top left. **b**–**d**, Examples of two cycles and a chain of templated insertions that affect *TERT* in hepatocellular carcinomas. **e**, Expression levels of *TERT* in patients with hepatocellular carcinoma (*n* = 187 patients), separated by whether *TERT* was wild type, had an activating promoter point mutation, structural variants in a templated insertion or other class. Individual patient data are shown as points. The box shows the median expression level as a thick black line, with the range of the box denoting the interquartile range. The whiskers show the range of data or 1.5× the interquartile range (whichever is lower).

Structural variants drive tumour development through their effects on cancer genes, whether by altering gene copy number, disrupting tumour-suppressor genes, creating fusion genes or juxtaposing the coding sequence of one gene with the regulatory apparatus of another. We found that many liver cancers had cycles of templated insertions that affect *TERT* (Fig. 3d, e, Extended Data Fig. 4). Point mutations in the *TERT* promoter are present in 54% of liver cancers, and a further 5–10% of liver cancers have structural variants that activate the gene^28^. Of the 30 patients with liver cancer that had structural variants that affect *TERT*, we find that 10 of these variants were templated insertion events (mostly cycles). All of these events duplicated the entire *TERT* gene and linked it to duplications of whole genes, fragments of genes or regulatory elements from elsewhere in the genome, and led to increased expression of *TERT* (Extended Data Fig. 4e). Thus, this particular rearrangement process is distinctive for the precision with which cancer copy-and-pastes normally disparate functional elements of its genome together without wholesale instability.

**Extended Data Fig. 5:**
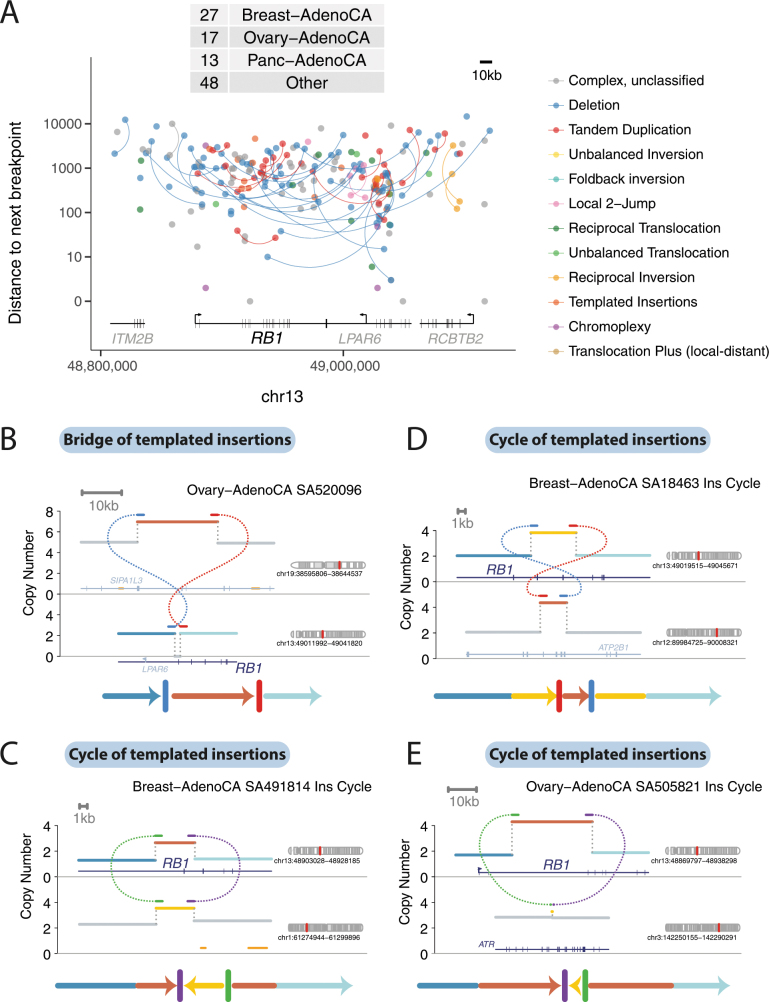
Templated insertion events inactivating *RB1* in breast and ovarian carcinomas. **a**, The positions of all structural-variant breakpoints in the *RB1* region in the PCAWG cohort (including 50-kb flanks either side of *RB1*), coloured by classification and vertically spaced by the distance to the next breakpoint in the cohort. If the two sides of a breakpoint junction are contained within the plotting window, they are joined by a curved line. The number of samples with a breakpoint in the plotting window is annotated in the table in the top left. **b**–**e**, Examples of three cycles and a bridge of templated insertions that affect *RB1* in breast and ovarian carcinomas.

Tumour-suppressor genes were also inactivated by templated insertions (Extended Data Fig. 5). For example, among many straightforward deletions, *RB1* was hit by cycles of templated insertions, a templated insertion with deletion and one instance of the linked, inverted duplications detailed in ‘Local *n*-jumps and local–distant clusters’. These events typically generated duplications of internal exons in *RB1* and/or insertions of exons from other genes, all of which presumably rendered a non-functional transcript.

## Local *n*-jumps and local–distant clusters

**Fig. 4:**
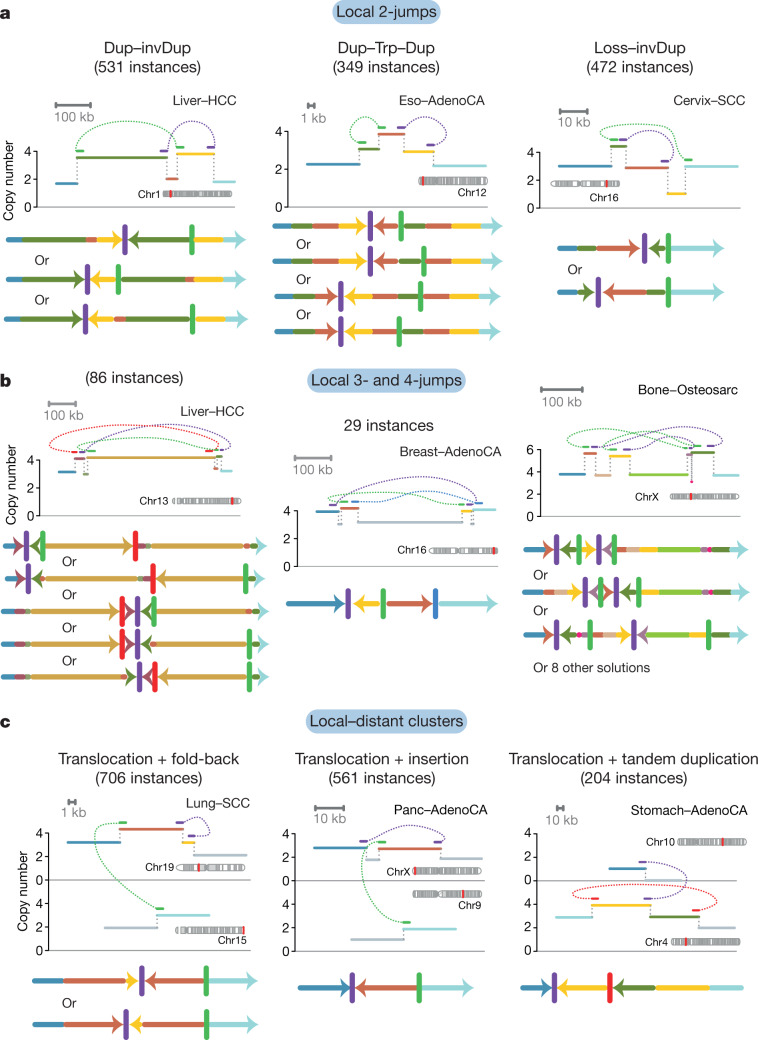
Examples of clusters of 2–5 rearrangements seen in human cancers. **a**, Structures created by two local rearrangements that cannot easily be explained by simple structural-variant classes (which we call local 2-jumps). The estimated copy-number profile is shown as in Fig. 1, with structural variants shown as dotted arcs linking two copy-number segments. Possible configurations of the derivative chromosome are shown below; multiple solutions are possible for each example. Dup, duplication; invDup, duplication linked by inverted rearrangement; trp, triplication. **b**, Structures created by 3–4 local rearrangements that cannot easily be explained by simple structural-variant categories. **c**, Structures created by one local rearrangement and one rearrangement that reaches elsewhere in the genome (local–distant clusters).

Many clusters of 2–10 structural variants in the dataset were confined to a single genomic region. Of those clusters that comprised two local rearrangements, some had straightforward explanations, such as nested or adjacent tandem duplications. However, many did not have a trivial explanation (Fig. 4a). These included a duplication–inverted-triplication–duplication structure that has previously been observed in germline structural variants^24^ (349 instances); a structure of two duplications linked by inverted rearrangements (531 instances); and structures of copy-number loss plus nearby duplication linked by inverted rearrangements (472 instances). All of these patterns had solutions in which breakpoints were phased to a single derivative chromosome (Fig. 4a), although non-phased solutions are theoretically possible (if unlikely). Beyond clusters of two rearrangements (two-jumps), we also found examples involving three, four or more rearrangements confined to one genomic locale (Fig. 4b). All of these configurations of clusters of structural variants can be phased to a single derivative chromosome, with tightly grouped breakpoints.

Beyond clusters confined to a single genomic region, we found clusters of 2–10 structural variants that combined local jumps with rearrangements that reach into one or more distant regions of the genome (Fig. 4c). Simple examples of these events include unbalanced translocations or large deletions with a locally derived fragment inserted at the breakpoint, but there was also an extensive range of more-complex patterns. In some cases, the source of the inserted fragment was distal to the major break, and the structural variant could feasibly result from several concurrent DNA breaks in close spatial proximity to the capture of a short DNA fragment during repair (cut-and-paste). In other cases, the origin of the inserted fragment was proximal to the major break and associated with a gain in copy number. This pattern is difficult to explain by a cut-and-paste mechanism, because the copy-number gain implies the inserted segment was a duplicate of the original template rather than a separated fragment redistributed from its original locus. Instead, a copy-and-paste mechanism may be the more parsimonious explanation for these events.

A comparison of local footprints linked together through distant rearrangements revealed a strong connectivity of footprints with the same or similar structure, often enriched tenfold or more than expected by chance (see ‘Footprint connectivity analysis’ in Supplementary Results). The reasons for this are unclear, but it may reflect innate structural symmetry introduced through the generation or the resolution of rearrangements, or through the repeated action of a mechanism that imparts consistent structural motifs.

## Copy-and-paste patterns of clusters

The diverse patterns of 2–10 clustered structural variants (Figs. 3, 4) share important morphological features: (1) genomic configurations that can be phased to a single derivative chromosome; (2) low-level gains in copy number, especially duplications and triplications; (3) a high frequency of inverted rearrangements in addition to noninverted rearrangements; (4) occurrence on a chromosome background with similar average copy number to the tumour overall; and (5) tight proximity of breakpoints within the local footprint (typically <1 Mb).

Using our in silico library of genomic configurations, we could define all possible routes by which sequential structural variants could generate these structures through the classically defined repertoire of deletion, tandem duplication, inversion and translocation (Supplementary Fig. 4). These routes typically would require implausible machinations of chromosomes (Supplementary Results). In particular, the high prevalence of inverted breakpoint junctions and local copy-number gains is difficult to recreate using sequential simple rearrangements. Simple inversion events are uncommon in cancers (Fig. 1d) and they tend not to generate copy-number gains, except through breakage–fusion–bridge cycles: these latter also cause terminal deletions^2^, which are not seen in the events discussed here.

If these events cannot be satisfactorily explained by sequential simple rearrangements, another possible explanation is a complex cut-and-paste mechanism such as chromothripsis, chromoplexy or repeated breakage–fusion–bridge cycles. However, the patterns of the 2–10 clustered structural variants do not fit with these processes either (Supplementary Results). Although chromothripsis with copy-number gain has previously been described^3,11,19,22^, the resulting copy number and rearrangement patterns have different properties to those we observed. Chromoplexy, in which chromosome breaks lead to a balanced interchange at multiple breakpoint junctions^5,20^, typically generates unphased solutions. Repeated breakage–fusion–bridge cycles tend to cause high-level copy-number gains associated with inverted, fold-back rearrangements^1,2^, unlike the structures reported here.

Instead, we believe that many of these locally complex clusters of structural variants with low-level copy-number gains are generated in a single event by a copy-and-paste process. That is, the copying of genomic templates is an intrinsic aspect of the structural variation process in these events, with the extra copies being inserted in the resulting derivative chromosome. If the genomic templates all originate locally, we would observe local *n*-jumps (such as in Fig. 3a, b) with a tight clustering of breakpoints, phased solutions, frequent copy-number gains and a mix of inverted and noninverted breakpoint junctions. If the original templates for the copied segments derive from across the genome, chains, cycles and bridges of templated insertions would arise (Fig. 2).

## Genomic properties of structural variants

**Fig. 5:**
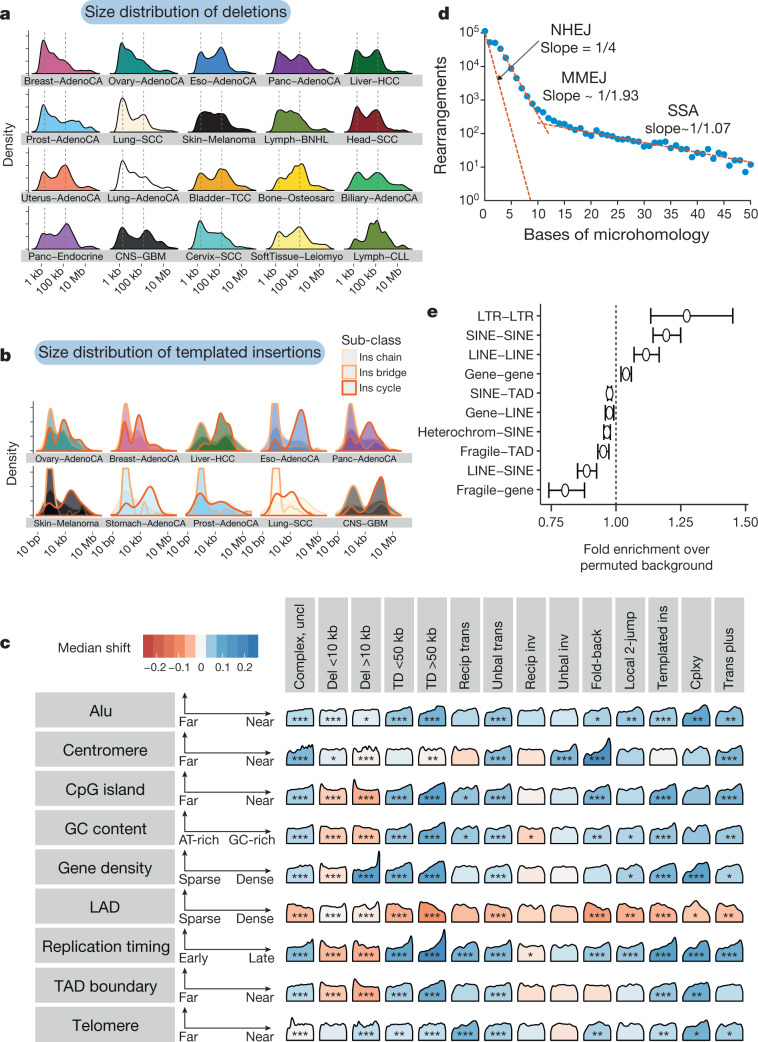
Size distribution and genomic properties of classified structural variants. **a**, Size distribution of deletions per histology group, with tumour types ordered according to total number of events seen. Vertical dashed lines represent the two prominent modes. **b**, Size distribution of segments of templated insertion per histology group. For each tumour type, the three distributions for cycles, bridges and chains of templated insertions are superimposed. Ins, insertion. **c**, Associations between a subset of the genomic properties (rows) and classes of structural variant (columns). Each density curve represents the quantile distribution of the genomic property values at observed breakpoints compared to random genome positions. Asterisks indicate a significant departure from uniform quantiles after multiple hypothesis correction on a one-sided Kolmogorov–Smirnov test based on a sample size of 2,559 genomes containing structural variants: *false-discovery rate < 0.01, **false-discovery rate < 0.001, ***false-discovery rate < 10^−6^. Cells with significant property associations are shaded by the magnitude of the shift of the median observed quantile above (blue) or below (red) 0.5. The interpretation of each property from left to right is indicated by the axes to the right of the property label. Complex uncl, complex clusters unclassified; cplxy, chromoplexy; del, deletion; inv, inversion; ins, insertion; LAD, lamina-associated domain; recip, reciprocal; TAD, topologically associated domain; TD, tandem duplication; trans, translocation; unbal, unbalanced. **d**, Rearrangement counts as a function of bases of junction microhomology, fit to three linear functions consistent with different formation mechanisms. NHEJ, non-homologous end joining; MMEJ, microhomology-mediated end joining; SSA, single-strand annealing. **e**, Enrichment or depletion of breakpoint junctions between regions of the genome with particular annotations, compared with a permuted background that preserves breakpoint positions but swaps breakpoint partners. Centre points are the mean fold change over the permuted background; error bars represent three s.d. Analysis is based on a sample size of 2,559 genomes containing structural variants. LTR, long terminal repeat; SINE, short interspersed nuclear element; LINE, long interspersed nuclear element; heterochrom, heterochromatin.

**Extended Data Fig. 6:**
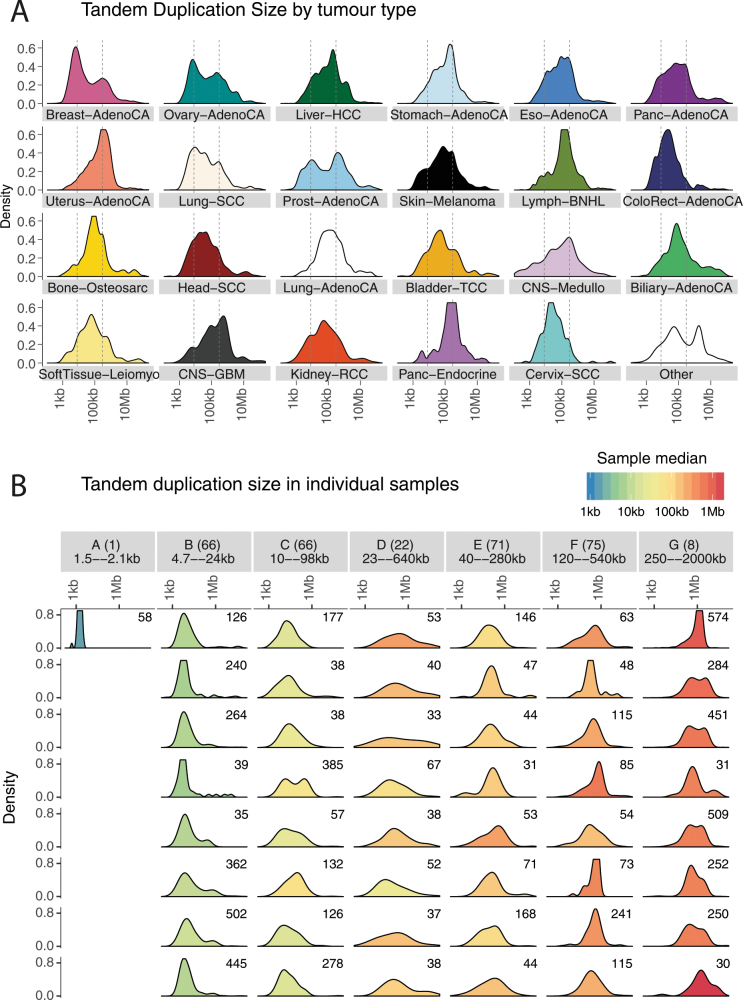
Size distribution of tandem duplications. **a**, Size distribution of tandem duplications per histology group. **b**, Samples with more than 20 tandem duplications were grouped using hierarchical clustering according to the within-patient distribution of tandem-duplication size. Seven clusters emerged, with the size distribution of up to eight randomly chosen samples per cluster illustrated. The numbers in the top right of each panel denote the number of tandem duplications in that sample.

**Extended Data Fig. 7:**
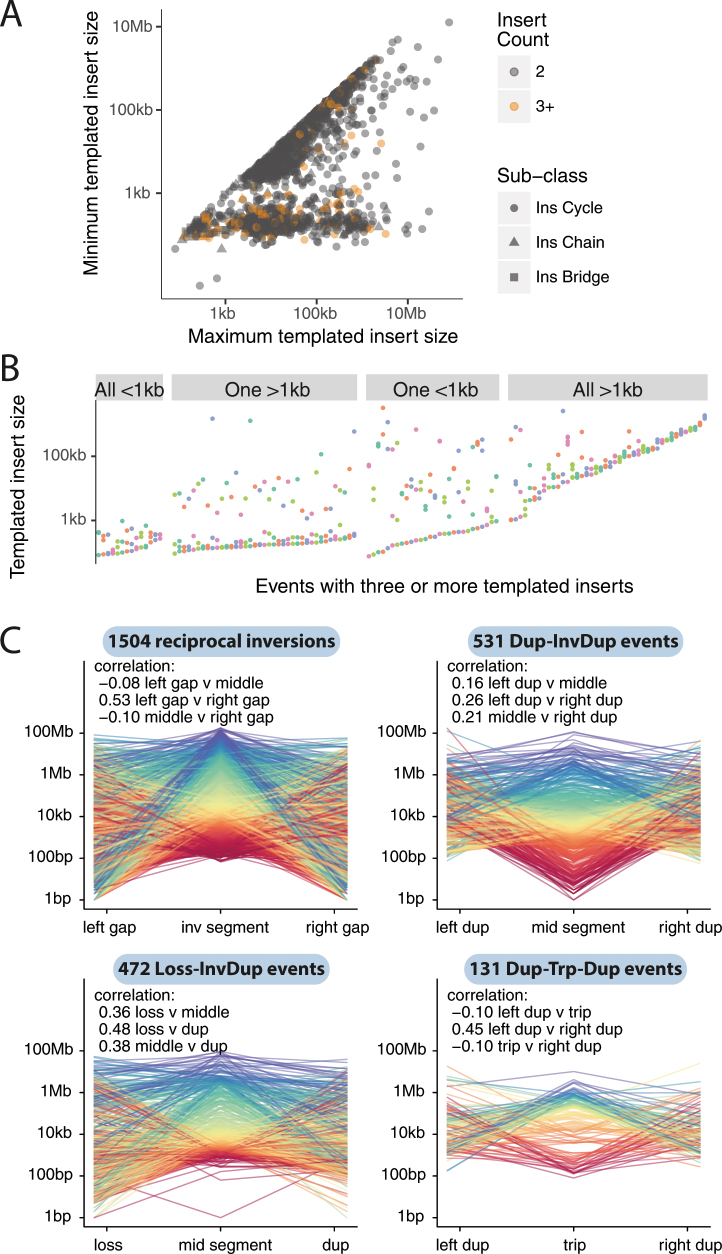
Size properties of clustered structural-variant classes. **a**, Comparison of the minimum and maximum templated-insert size for multi-insert cycles, chains and bridges of templated insertions. **b**, All events with three or more templated inserts, grouped by combination of insert sizes. **c**, Correlations (Pearson’s correlation coefficient) and raw sizes of individual genomic segments for reciprocal inversions and local two-jumps. Each individual event is shown as a line that links the size of the individual segments in that event. The sample sizes for each event class are shown in the labels for each panel.

The size of tandem duplications and deletions followed complex—often multimodal—distributions across tumour types (Fig. 5a, Extended Data Fig. 6a). However, as previously reported^6,29^, individual patients tend to have a simpler—usually unimodal—distribution of deletions or tandem duplications (Extended Data Fig. 6b), which implies that the complexity seen in a given tumour type results from combining samples with different profiles. The sizes of individual fragments in templated insertion events were also distinctly multimodal, with varying peak heights across tumour types (Fig. 5b). When correlating template sizes within a given event, two patterns emerged: one in which template sizes were closely correlated with one another, and one in which a small (<1 kb) template was linked with one of any size (Extended Data Fig. 7a, b). Likewise, the sizes of segments within a given local two-jump event showed moderately strong correlations with one another (Extended Data Fig. 7c).

**Extended Data Fig. 8:**
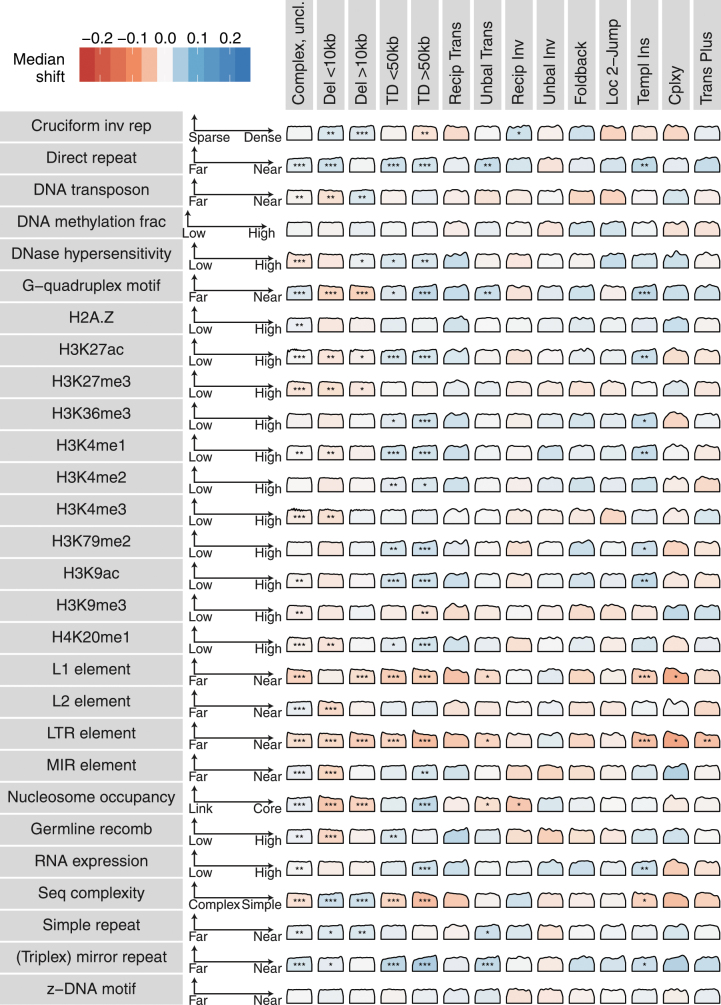
Relationship of an extended panel of genomic properties with structural-variant categories. Associations between a subset of the genomic properties (rows) and classes of structural variant (columns). Each density curve represents the quantile distribution of the genomic property values at observed breakpoints, compared to random genome positions. Asterisks indicate significant departures from uniform quantiles after multiple hypothesis correction by the Benjamini–Yekutieli method on a one-sided Kolmogorov–Smirnov test, based on a sample size of 2,559 genomes containing structural variants: *false-discovery rate < 0.01, **false-discovery rate < 0.001, ***false-discovery rate < 10^−6^. Cells with significant property associations are shaded by the magnitude of the shift of the median observed quantile above (blue) or below (red) 0.5. The interpretation of each property from left to right is indicated by the axes to the right of the property label.

A number of genomic properties (such as replication timing, transcriptional activity and chromatin state) influence the density of point mutations^30,31^ and copy-number alterations^32^, but how this relates to individual classes of structural variant is unclear. From the literature, we compiled a library of the genome-wide distribution of 38 features including replication timing, GC content, repeat density, gene density and distance to G-quadruplex motifs, among others. Replication timing had the strongest association with the occurrence of structural variants; deletions are enriched in late-replicating regions, and tandem duplications and unbalanced translocations occur preferentially in early-replicating regions (Fig. 5c, Extended Data Fig. 8). For individual patients with high numbers of deletions or tandem duplications, we observed notable heterogeneity in the distribution of these structural variants according to replication timing: some had events that occurred predominantly in late-replicating regions, others had events that occurred exclusively in early-replicating regions, and in others events were distributed more evenly (Supplementary Fig. 5). Regions of active chromatin and increased gene density correlated positively with the rate of rearrangement.

A structural variant requires DNA repair pathways to join two sequences together, and several repair mechanisms are available to somatic cells. Some require sequence homology between the two ends, and others can operate to join non-homologous sequences. As previously reported^2,25,33^, we find across the PCAWG data that many structural variants do not have sequence homology at the breakpoint junction (Fig. 5d) and therefore arise through non-homologous end joining. Nonetheless, a sizable fraction of structural variants has more microhomology than expected by chance, with an apparently bimodal distribution of microhomology lengths. One set of structural variants has 2–7 bp of microhomology, probably generated by microhomology-mediated end joining, and a second set of structural variants has 10–30 bp of microhomology, probably generated through single-strand annealing or other forms of homologous recombination (including microhomology-mediated break-induced replication). Repetitive sequences in the genome, such as short and long interspersed nuclear elements, are the likely substrate of such structural variants, and we find enrichment for structural variants joining such elements (Fig. 5e, Supplementary Fig. 6).

## Signatures of structural variation

The heterogeneous spectrum of point mutations across cancers can be reconstructed from the differential action of a relatively limited repertoire of mutational processes, each with a characteristic signature^34^. The differences across patients in the size distribution of tandem duplication and deletion—together with the widely varying frequency and patterns of structural variant across tumour types and genome topology—suggested that we could similarly learn such correlations across individual classes of structural variant.

We divided the set of structural variants of each patient into mutually exclusive categories. We split the most frequent classes of simple structural variant (deletions and tandem duplications) into 11 categories according to size, replication timing and occurrence at fragile sites. Other configurations of structural variants and copy-number changes seen more than 50 times in the cohort were included as further categories, including cycles, chains and bridges of templated insertions (also split by size), local *n*-jumps and local–distant clusters.

**Fig. 6:**
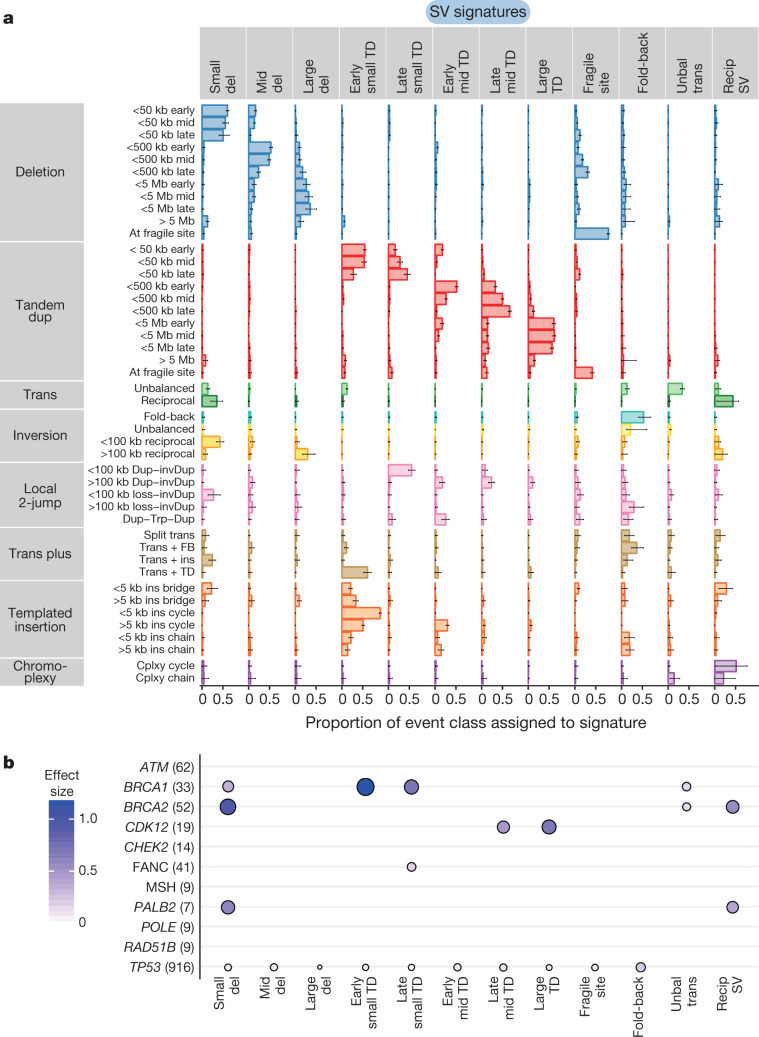
Structural-variant signatures in human cancers. **a**, The 12 most distinctive structural-variant signatures extracted by the Bayesian hierarchical Dirichlet process algorithm, run on a sample size of 2,559 genomes containing structural variants. Here the lengths of the bars represent the estimated proportion of each event class assigned to each signature (rows sum to one); the black line segments represent the 95% posterior interval for bar length from the Markov chain. FB, fold-back; mid, mid-sized. **b**, Association of pathogenic mutations (germline and somatic combined) in key DNA repair genes with structural-variant signatures. The sample size of patients who have pathogenic variants in the specific genes assessed is shown in brackets after each gene label (*y* axis). Hypothesis tests and effect sizes for each gene are derived from linear models for signature intensity after correction for histology. Significant associations from two-sided tests with correction for multiple hypothesis testing are shown. The colour and size of the points represent the estimated effect sizes. MSH refers to *MSH2*, *MSH3*, *MSH4* and *MSH6*, genes in the mismatch repair pathway; FANC refers to genes associated with Fanconi anaemia, namely *FANCA*, *FANCC*, *FANCD2*, *FANCE*, *FANCF*, *FANCG*, *FANCI*, *FANCL* and *FANCM*.

We applied two methods for signature discovery, which yielded comparable results. We identified 16 structural-variant signatures: the 12 most prevalent of these signatures are shown in Fig. 6a. Signature extraction on the cohort randomly split into two halves identified ten highly correlated signatures (Supplementary Fig. 7), which closely matched the signatures called in the full cohort despite the lower power. Three signatures of deletions emerged, split by size: the signature of small (<50-kb) deletions included small reciprocal inversions and the signature of large (>500-kb) deletions included large reciprocal inversions. This implies that the frequencies of deletions and reciprocal inversions are correlated across the cohort, and both follow similar size distributions within an individual patient.

We identified five signatures of tandem duplications, split by size and replication timing. Cycles, bridges and chains of templated insertions were particularly prominent in signatures of early-replicating tandem duplications, whereas local two-jump structures were more closely associated with late-replicating tandem duplications. All of these patterns exemplify the copy-and-paste concept, in which extra copies of genomic templates are produced and inserted as an integral feature of the structural-variant process.

**Extended Data Fig. 9:**
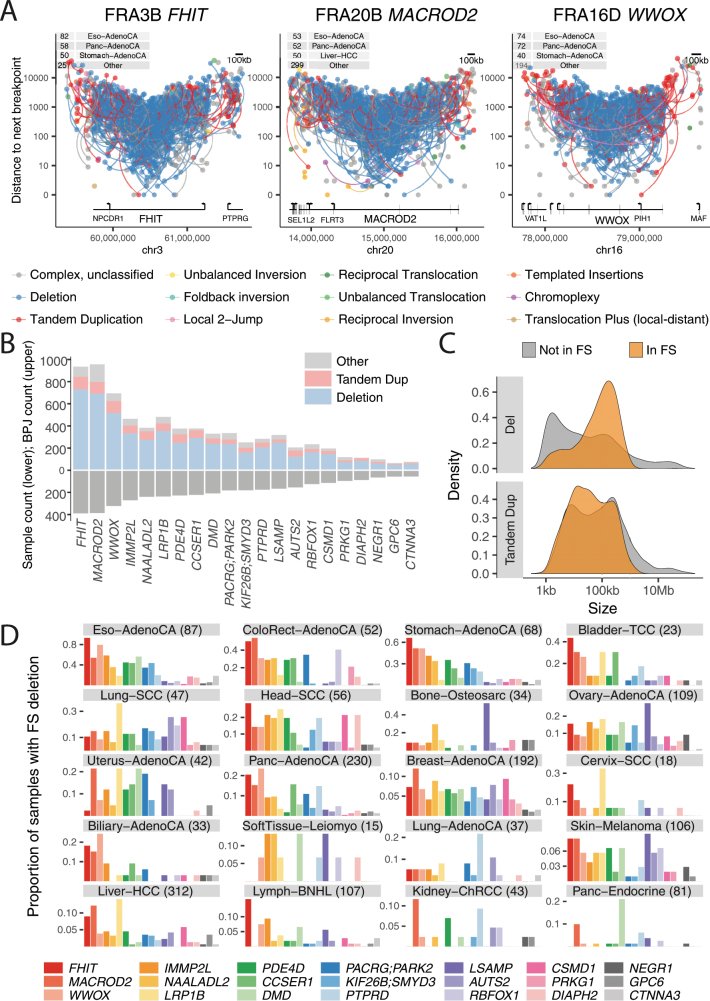
Properties of structural variants at chromosomal fragile sites. **a**, Structural-variant breakpoints in the most affected fragile sites: *FHIT*, *MACROD2* and *WWOX*. These are coloured by classification and vertically spaced by the distance to the next breakpoint in the cohort. If the two sides of a breakpoint junction are contained within the plotting window, they are joined by a curved line. The number of samples with a breakpoint in the plotting window is annotated in the tables at the top left. **b**, Number of deletions and tandem duplications (top) and number of affected samples (bottom) for the 18 fragile sites considered in this analysis. **c**, Size distribution of deletions and tandem duplications in fragile sites (FS) compared to the rest of the genome. **d**, Fragile-site preference for 20 cancer histology groups as indicated by the proportion of samples that contains a deletion in each of the 18 fragile sites considered here. The number of samples is indicated in parentheses.

Another signature was characterized by deletions and tandem duplications at chromosomal fragile sites^35^. Tandem duplications were more prominent at the edges of the fragile site, and deletions were concentrated in the centre (Extended Data Fig. 9a, b). The size range of fragile site deletions peaked at around 100 kb, similar to the larger deletion signature, whereas the rarer fragile-site tandem duplications showed no strong size peak (Extended Data Fig. 9c). Sites of fragility varied extensively across tumour types (Extended Data Fig. 9d).

Unbalanced translocations comprised their own signature, which suggests that they derive from a distinct rearrangement process in cancer genomes. A further signature comprised both the fold-back inversions that are a hallmark of breakage–fusion–bridge cycles and similar structures such as translocations adjacent to fold-back inversions. Finally, there was a signature of balanced rearrangements, including reciprocal translocations and chromoplexy clusters^5^. This signature probably arises from several double-stranded DNA breaks (potentially occurring in interphase), in which both sides of the break are incorrectly repaired through ligation to other, simultaneously broken regions of the genome.

## DNA repair genes and tumour type

**Extended Data Fig. 10:**
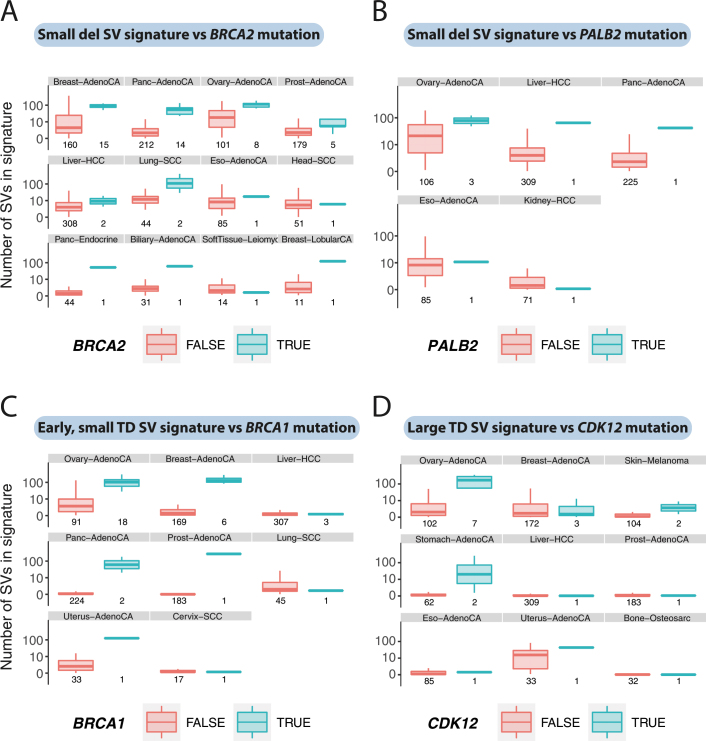
Consistency of associations between signatures and mutations in DNA-repair genes. **a**, Box-and-whisker plots showing the number of structural variants attributed to the small-deletion signature in different types of tumour, split by *BRCA2* status (*BRCA2* wild type in orange; *BRCA2* mutant in cyan). The box denotes the interquartile range, with the median marked as a horizontal line. The whiskers extend as far as the range or 1.5× the interquartile range, whichever is lower. Outlier patients are shown as points. There is an increase in events attributed to the small-deletion signature when *BRCA2* is mutated, across multiple types of tumour (breast, pancreatic, ovarian, prostate, lung squamous and so on). **b**, Box-and-whisker plots as for **a**, showing the number of structural variants attributed to the small-deletion signature in different types of tumour, split by *PALB2* status. **c**, Box-and-whisker plots as for **a**, showing the number of structural variants attributed to the early-replicating, small-tandem-duplication signature in different types of tumour, split by *BRCA1* status. **d**, Box-and-whisker plots as for **a**, showing the number of structural variants attributed to the large-tandem-duplication signature in different types of tumour, split by *CDK12* status.

We grouped annotations of pathogenic germline variants and somatic driver mutations in DNA-repair genes across the cohort^8^, correlating their presence with activity of the structural-variant signatures (Fig. 6b). As previously described for breast and ovarian cancers^6,29^, *BRCA1* mutations are significantly associated with small tandem duplication signatures, the mechanistic basis of which is increasingly well understood^10^. As previously described^6,36^, *CDK12* variants predicted signatures of mid-sized-to-large tandem duplications. *BRCA2* variants correlated with small deletions, as expected from previous work^29^, and also with the reciprocal structural-variant signature that includes chromoplexy. *PALB2* variants showed the same correlations with signatures of small deletions and reciprocal structural variants as does *BRCA2*: PALB2 colocalizes with, stabilizes and assists BRCA2 during homologous recombination^37^, so we might have predicted that inactivation of either gene would lead to a similar structural-variant signature. These associations between driver mutations and structural-variant signatures were consistently evident across many types of tumour (Extended Data Fig. 10).

The structural-variant signatures showed considerable heterogeneity in their activity across tumour types and among patients within a given tumour type (Supplementary Fig. 8). Tumours of the gastrointestinal tract—including colorectal and oesophageal adenocarcinomas—showed high rates of the fragile-site signature. Prostate cancer was notable for the prevalence of the chromoplexy signature, as previously reported^5,20^, and squamous cell carcinomas of the lung were characterized by the fold-back inversion signature.

**Extended Data Fig. 11:**
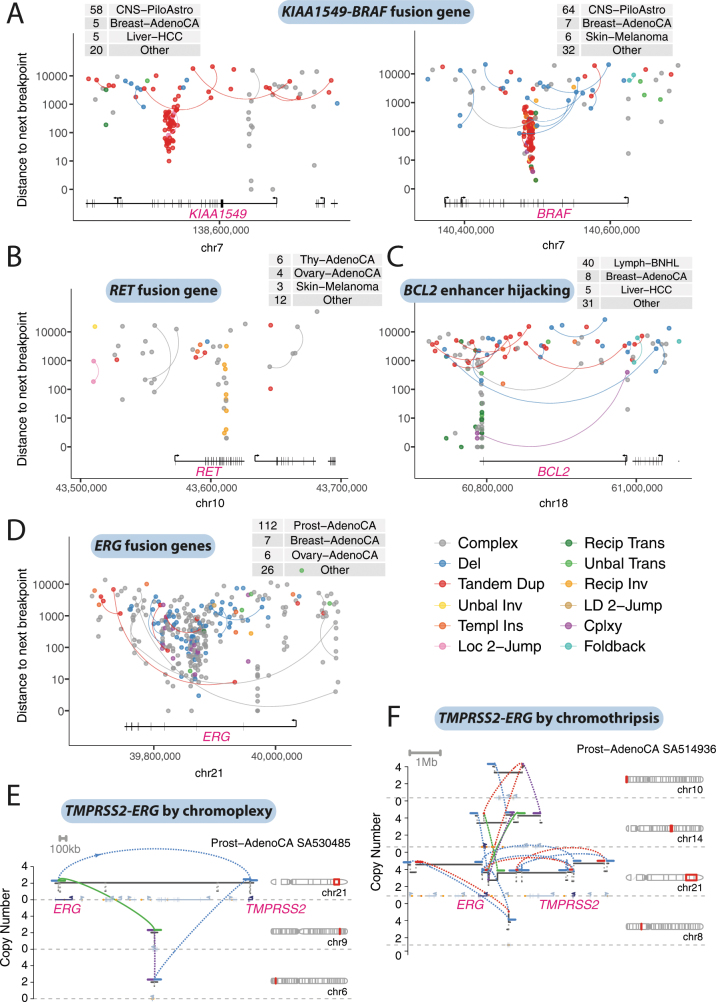
Patterns of structural variants causing fusion genes and enhancer hijacking. **a**, Rainfall plot of structural-variant breakpoints in the genes *KIAA1549* and *BRAF*, commonly fused together through a tandem duplication in pilocytic astrocytomas. Structural variants are coloured by classification and arranged vertically by the distance to the next breakpoint in the cohort. If the two sides of a breakpoint junction are contained within the plotting window, they are joined by a curved line. The number of samples with a breakpoint in the plotting window is annotated in the table at the top of each panel. **b**, Rainfall plot of structural-variant breakpoints that affect *RET*, commonly fused to *CCDC6* by inversion in papillary thyroid cancer. **c**, Rainfall plot of structural-variant breakpoints that affect *BCL2*, commonly hijacked to the *IGH* immunoglobulin locus by translocations in B cell lymphomas. **d**, Rainfall plot of structural-variant breakpoints that affect *ERG*, commonly fused with *TMPRSS2* by deletion or more-complex events in prostate adenocarcinoma. **e**, Example of a *TMPRSS2-ERG* fusion gene in a prostate adenocarcinoma created by a chromoplexy cycle. The estimated copy-number profile is shown as black horizontal segments, with structural variants shown as dotted arcs linking the edges of two copy-number segments. **f**, Example of a *TMPRSS2-ERG* fusion gene in a prostate adenocarcinoma created by chromothripsis.

We assessed how classes of structural variant altered known cancer genes (Supplementary Table 1). Some cancer genes acquire oncogenic potential only with specific structural events, such as fusion genes or enhancer hijacking. Not surprisingly, these genes typically showed little variability in which classes of structural variant could generate such events (Extended Data Fig. 11a–c)—although there were exceptions. The *TMPRSS2-ERG* fusion gene of prostate cancer, for example, was generated by a range of processes (including simple deletions, chromoplexy and chromothripsis), all of which are prevalent signatures in this tumour type (Extended Data Fig. 11d–f).

**Extended Data Fig. 12:**
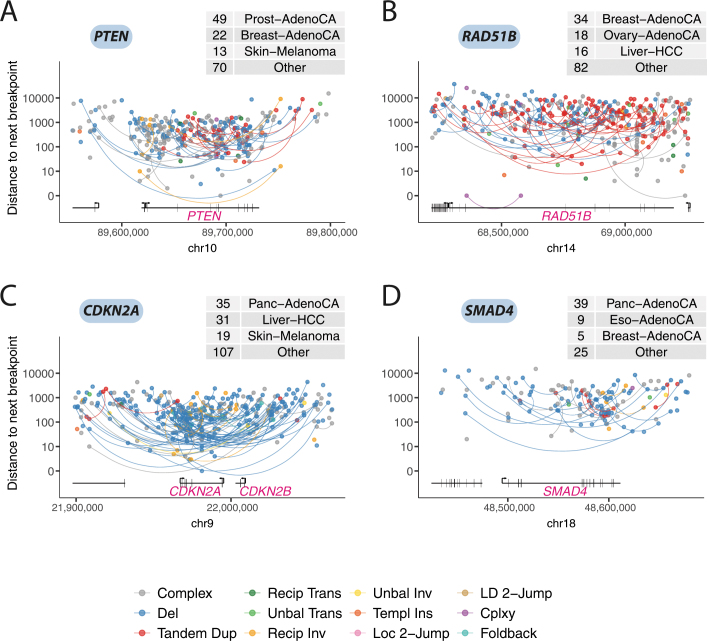
Patterns of structural variants that affect selected tumour-suppressor genes. **a**, Rainfall plot of structural-variant breakpoints in the gene *PTEN*, commonly inactivated in breast and ovarian adenocarcinomas, in which tandem-duplication signatures are frequent. Structural variants are coloured by classification and arranged vertically by the distance to the next breakpoint in the cohort. If the two sides of a breakpoint junction are contained within the plotting window, they are joined by a curved line. The number of samples with a breakpoint in the plotting window is annotated in the table at the top of each panel. **b**, Rainfall plot of structural-variant breakpoints that affect *RAD51B*, commonly inactivated in breast and ovarian adenocarcinomas. **c**, Rainfall plot of structural-variant breakpoints that affect *CDKN2A*, commonly inactivated in tumours of the gastrointestinal tract, in which deletion signatures are common. **d**, Rainfall plot of structural-variant breakpoints that affect *SMAD4*, commonly inactivated in tumours of the gastrointestinal tract.

**Extended Data Fig. 13:**
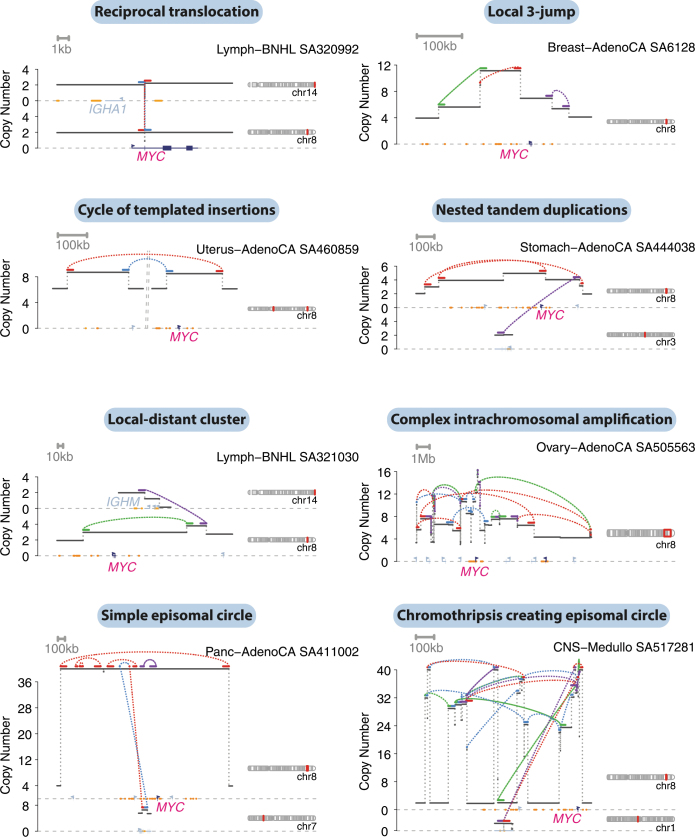
Examples of structural variants increasing the copy number of *MYC*. The estimated copy-number profile is shown as black horizontal segments, with structural variants shown as dotted arcs linking the edges of two copy-number segments.

Tumour-suppressor genes and recurrently amplified genes showed more variability in which types of structural variant were observed, and these were shaped by signatures active in the relevant tumour types. For example, the tumour-suppressor genes, *PTEN* and *RAD51B*, which are commonly inactivated in breast and ovarian cancers, were often targeted by tandem duplications generating out-of-frame exon duplications (Extended Data Fig. 12a, b). By contrast, deletions were the predominant events that inactivated *SMAD4* and *CDKN2A*, in keeping with their prevalence in cancers of the gastrointestinal tract (Extended Data Fig. 12c, d). *MYC*, one of the most commonly amplified genes across all types of cancer, showed considerable diversity in the mechanisms of its rearrangement: nested tandem duplications in breast cancer, translocations or chromoplexy with *IGH* in lymphoma, as well as chromothripsis, cycles of templated insertions, local *n*-jumps and local–distant clusters in other types of tumour (Extended Data Fig. 13).

## Discussion

We have described the patterns and signatures of structural variation in a large cohort of uniformly analysed cancer genomes. A major grouping of patterns in structural variants that emerges from our study is one in which extra copies of genomic templates are inserted during the rearrangement process. This includes simple events such as tandem duplications, as well as a range of more-complex events with duplications and triplications that are rearranged locally as well as inserted distantly. Our signature analysis grouped a large proportion of these more-complex events together with tandem duplications, which suggests that they represent a continuum of processes that share underlying properties. A replication-based mechanism has previously been proposed to explain local two-jumps^4,23,24^, in which stalled replication forks or other DNA lesions cause the DNA polymerase to switch templates and continue replication in a new location. Studies in experimental models are now revealing that a wide range of mechanisms and DNA lesions can result in templated insertions: these mechanisms include tandem duplications in *BRCA1* deficiency^10^, translocations with templated insertions caused by dysregulated strand invasion^38^ and distant templated insertions in the absence of replication helicases^39^.

Genomic instability in cancer is not a single phenomenon. Instead, many different mutational processes can act to restructure the genome and, in doing so, generate a notably flexible array of possible structures. Any given tumour draws on a subset of the available processes, shaped by the cell of origin, germline predisposition and other, unknown, factors: selection then does the rest, promoting the clone that has chanced on the structure that increases its potential for self-determination.

## Methods

No statistical methods were used to predetermine sample size. The experiments were not randomized and investigators were not blinded to allocation during experiments and outcome assessment.

A detailed description of the methods used in this paper and many additional results are described in Supplementary Information. Here, we summarize the key aspects of the analysis.

### Generation of the structural-variant call set

The final set of structural variants used in this Article was generated by the Technical Working Group of the PCAWG Consortium and is described in the main PCAWG paper^8^. In brief, four variant callers were used to identify somatically acquired structural variants from matched tumour and germline whole genome sequencing data: SvABA (Broad pipeline), DELLY (DKFZ pipeline), BRASS (Sanger pipeline) and dRanger (Broad pipeline). These were merged into a final call set using a graph-based algorithm to identify overlapping breakpoint junctions across algorithms. Detailed visual inspection of structural-variant calls suggested that a simple approach of accepting all structural-variant calls made by two or more of the four algorithms gave the best trade-off between sensitivity and specificity.

### Structural-variant clustering and annotation

To identify clusters of structural variants, we developed a method for grouping structural variants into clusters and footprints to allow structural and mechanistic inferences to be made systematically. In parallel, we processed the somatic copy-number data and merged it with structural-variant junctions to enable us produce rearrangement patterns from the generated structural-variant clusters and footprints. We produced normalized representations of structural-variant cluster patterns, which enable us to tabulate the number of different cluster and footprint patterns and analyse their features. Finally, we performed manual and simulation-assisted interpretation of the recurrently observed cluster and footprint patterns. The individual steps of the structural-variant classification pipeline are outlined below and detailed in the subsequent subsections: (1) computing the exact breakpoint coordinates from clipped reads; (2) removing redundant ‘segment-bypassing’ structural variants; (3) merging rearrangement breakpoints with copy-number data to yield structural-variant breakpoint-demarcated, normalized, absolute copy-number data; (4) clustering individual structural variants into structural-variant clusters and footprints; (5) heuristically refining structural-variant clusters and footprints; (6) filtering artefactual fold-back-type structural variants with insufficient support; (7) determining balanced overlapping breakpoints (this step is to distinguish very short templated insertions from mutually overlapping balanced breakpoints); and (8) computing rearrangement patterns and categories.

### Distribution of structural variants across the genome

We divided the hg19 human reference genome (autosomes and chromosome X) into 3,036,315 pixels of 1 kb, and calculated a suite of metrics per pixel to summarize a variety of genome properties with potential relevance to the distribution of rearrangements, as listed in the Supplementary Information. Properties were matched as closely as possible to the tissue of origin for cancer samples from the PCAWG data. All other genome properties were held fixed across all tissues. To test for associations between structural-variant event classes and the library of genome properties, the genome property metrics were compared between real structural-variant positions (randomly choosing one side of each breakpoint junction to reduce dependence between observations) and one million uniform random positions from the callable genome space. To compare the tissue-specific properties, each random position was assigned a random tissue type, drawing from the observed tissue-type distribution in the structural-variant call set. For each genome property and each event class, the real observations were pooled amongst the random ones, and then rank-transformed and normalized on a scale from 0 to 1. Under the null hypothesis of no event-versus-property association, the ranks of the real observations would follow a uniform distribution. We tested this in each case with a Kolmogorov–Smirnov test then applied a Benjamini–Yekutieli correction for false-discovery rate across the entire suite of tests and set the threshold for significance reporting at 0.01.

### Structural-variant-signature analysis

We used two algorithms for extracting structural-variant signatures. Both used the same input files, comprising a matrix of counts per patient (across all patients) of structural-variant clusters falling into a number of mutually exclusive categories. These categories included the major classes of structural variants, with the more-common events (deletions, tandem duplications and inversions) split by size and/or replication timing. The two algorithms that were  used for extracting the signatures were (1) a hierarchical Dirichlet process and (2) non-negative matrix factorization. Further details on the implementation of these algorithms are available in the Supplementary Information.

### Reporting summary

Further information on research design is available in the Nature Research Reporting Summary linked to this paper.

## Online content

Any methods, additional references, Nature Research reporting summaries, source data, extended data, supplementary information, acknowledgements, peer review information; details of author contributions and competing interests; and statements of data and code availability are available at 10.1038/s41586-019-1913-9.

## Supplementary information

Supplementary Information This file contains Supplementary Figures 1-8, Supplementary Methods, Supplementary Results, References and a list of participants in ICGC/TCGA Pan-Cancer Analysis of Whole Genomes Consortium.

Reporting Summary

Supplementary Table Supplementary Table 1: Counts of patients with SVs in different classes affecting genes in the Cancer Gene Census.

## Figures and Tables

**Extended Data Table 1:**
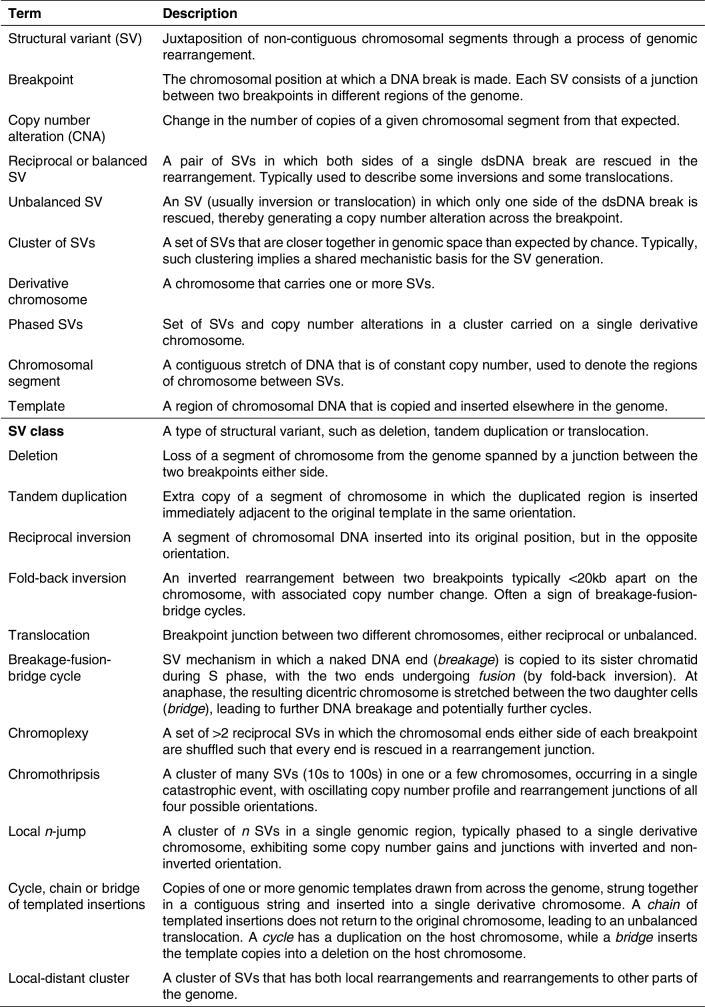
Glossary of key terms

## Data Availability

Somatic and germline variant calls, mutational signatures, subclonal reconstructions, transcript abundance, splice calls and other core data generated by the ICGC/TCGA PCAWG Consortium are described in an accompanying Article^8^ and are available for download at https://dcc.icgc.org/releases/PCAWG. Additional information on accessing the data, including raw read files, can be found at https://docs.icgc.org/pcawg/data/. In accordance with the data access policies of the ICGC and TCGA projects, most molecular, clinical and specimen data are in an open tier that does not require access approval. To access information that could potentially identify participants, such as germline alleles and the underlying sequencing data, researchers will need to apply to the TCGA data access committee via dbGaP (https://dbgap.ncbi.nlm.nih.gov/aa/wga.cgi?page=login) for access to the TCGA portion of the dataset, and to the ICGC data access compliance office (http://icgc.org/daco) for the ICGC portion of the dataset. In addition, to access somatic single-nucleotide variants derived from TCGA donors, researchers will also need to obtain dbGaP authorization.

## Code availability

The core computational pipelines used by the PCAWG Consortium for alignment, quality control and variant calling are available to the public at https://dockstore.org/search?search=pcawg under the GNU General Public License v.3.0, which allows for reuse and distribution. These are described in detail in an accompanying Article^8^. The code for grouping structural variants into structural-variant clusters and footprints is available at https://github.com/cancerit/ClusterSV/ (version 1.0). The code for simulating rearrangements can be found at https://github.com/cancerit/SimSvGenomes (version 1.0). The code for sampling from the hierarchical Dirichlet process for identification of mutational signatures is implemented as an R package at https://github.com/nicolaroberts/hdp (version 0.1.1).
