# Supplementary material for: Patterns of somatic structural variation in human cancer genomes
Source: Nature. 2020 Feb 5;578(7793):112–21. doi: 10.1038/s41586-019-1913-9 (PMC7025897; doi:10.1038/s41586-019-1913-9)
Supplement: Supplementary file 1 — This file contains Supplementary Figures 1-8, Supplementary Methods, Supplementary Results, References and a list of participants in ICGC/TCGA Pan-Cancer Analysis of Whole Genomes Consortium. [file 41586_2019_1913_MOESM1_ESM.pdf]

nature

https://doi.org/10.1038/s41586-019-1913-9

Supplementary information

Patterns of somatic structural variation in human cancer genomes

In the format provided by the authors and unedited

Supplementary information

Patterns of somatic structural variation in human cancer genomes

Authors: Yilong Li [1,2] *; Nicola D Roberts [1] *; Jeremiah A Wala [3,4,5] *; Ofer Shapira [3,4,5] *; Steven E. Schumacher [3,4,5]; Ekta Khurana [6]; Sebastian Waszak [7]; Jan Korbel [7]; Marcin Imielinski [8]; Joachim Weischenfeldt [7,9] §; Rameen Beroukhim [3,4,5] §; Peter J Campbell [1,10] § on behalf of the PCAWG-Structural Variation Working Group ^ and the PCAWG Network.

Table of Contents

Supplementary Figures 1-8 .... **3**

Supplementary Methods **12**
Terminology and definitions **12**
Telomere and centromere copy number 12
Rearrangement end 13
Copy numbers associated with a rearrangement end 14
Rearrangement patterns 14
Copy number relative to chromosomal arm 15
An object-oriented framework for handling somatic SV and CNA data **15**
The SV clustering and classification pipeline **16**
1. Computing exact breakpoint coordinates from clipped reads 17
2. Removing redundant “segment-bypassing” SVs 21
3. Merging rearrangement breakpoints with copy number data 22
4. Clustering SVs into SV clusters and their constituent footprints 27
5. Heuristic refinement of SV clusters and footprints 30
6. Filtering artefactual fold-back-type SVs with insufficient support 31
7. Determining balanced overlapping breakpoints 33
8. Computing rearrangement patterns 33
A string representation for entire rearranged somatic genomes 33
A string representation for rearrangement patterns 36
Library of all possible rearrangement patterns **38**
Interpretation and analysis of rearrangement patterns **41**
Inverted copy number gain patterns 41
Signatures of somatic rearrangements **42**
Event and footprint types included in the analysis 42
Nonnegative matrix factorisation of SV event counts 44
Library of genome properties **45**
Callable genome space **48**
Genome property association testing for SV event classes **49**
Defining the major fragile sites in the PCAWG dataset **49**
HDP method for SV signature discovery **50**

Supplementary Results **51**
Rearrangement clustering and interpretation statistics **51**
Rearrangement footprints in cancer **52**
Footprints with single breakpoints 52
Footprints of two breakpoints from different rearrangements **53**

Balanced rearrangements and chromoplexy..56
Templated insertions..60
Footprints with four breakpoints..63
Local events involving two inversion-type SVs..64
Footprints with four breakpoints from different SVs..70
Footprints with breakpoints from three SVs..71
Footprints with three breakpoints..76
Analysis of footprint A+/C-/C+..80
Five breakpoint and six breakpoint footprints..81
Analysis of somatic rearrangement mutational signatures..83
Footprint connectivity analysis..86

References..90

List of PCAWG participants..91

Supplementary Figure 1. Basic concepts of calling and classifying SVs.

(A) Short-read sequencing used in PCAWG comprises sequencing of reads ~100bp in length from both ends of DNA molecules 300-500bp in length. When that DNA molecule spans a breakpoint junction, the reads have non-standard orientation or separation when mapped to the reference genome.

(B) Breakpoint junctions can occur in four possible orientations, depending on how the two DNA ends are joined (whether the 5' end or 3' end of the break). 'Rearrangement-side' versus 'Non-rearrangement side' denotes which side of a given breakpoint is captured in the breakpoint junction. 'Low end' versus 'high end' distinguishes the two segments that get juxtaposed in the breakpoint junction.

(C) Adjacent breakpoints when mapped to the reference genome can also follow four possible orientations, each with different implications for the underling SV process (see Supplementary Methods).

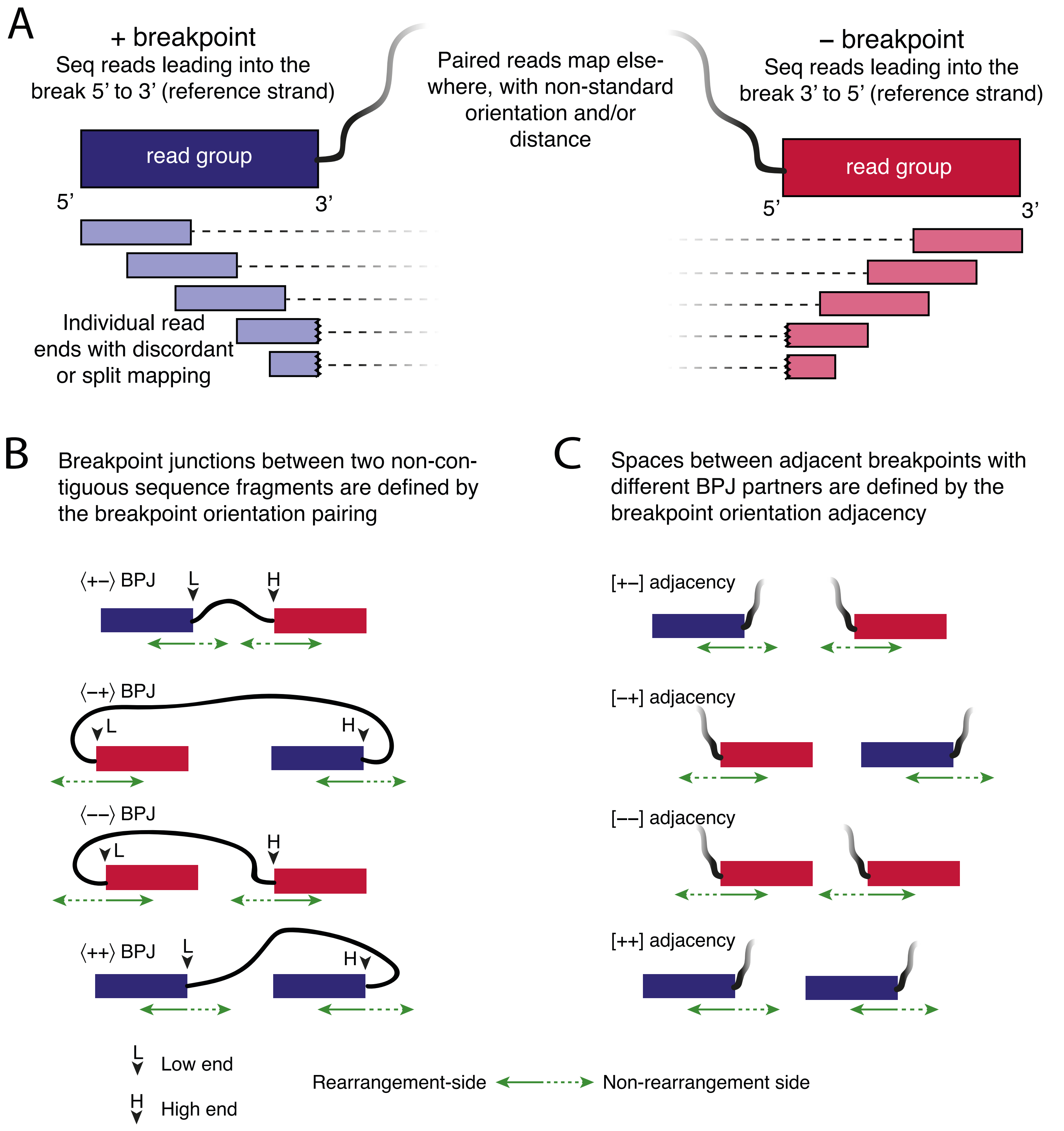

Supplementary Figure 2. Transcriptional evidence for phasing of chains and cycles of templated insertions. For (A) a chain of templated insertions and (B) a cycle of templated insertions, we find evidence in the RNA-sequencing data of transcripts that span two breakpoint junctions between templates. This would only be possible if the two breakpoint junctions were phased to the same derivative chromosome.

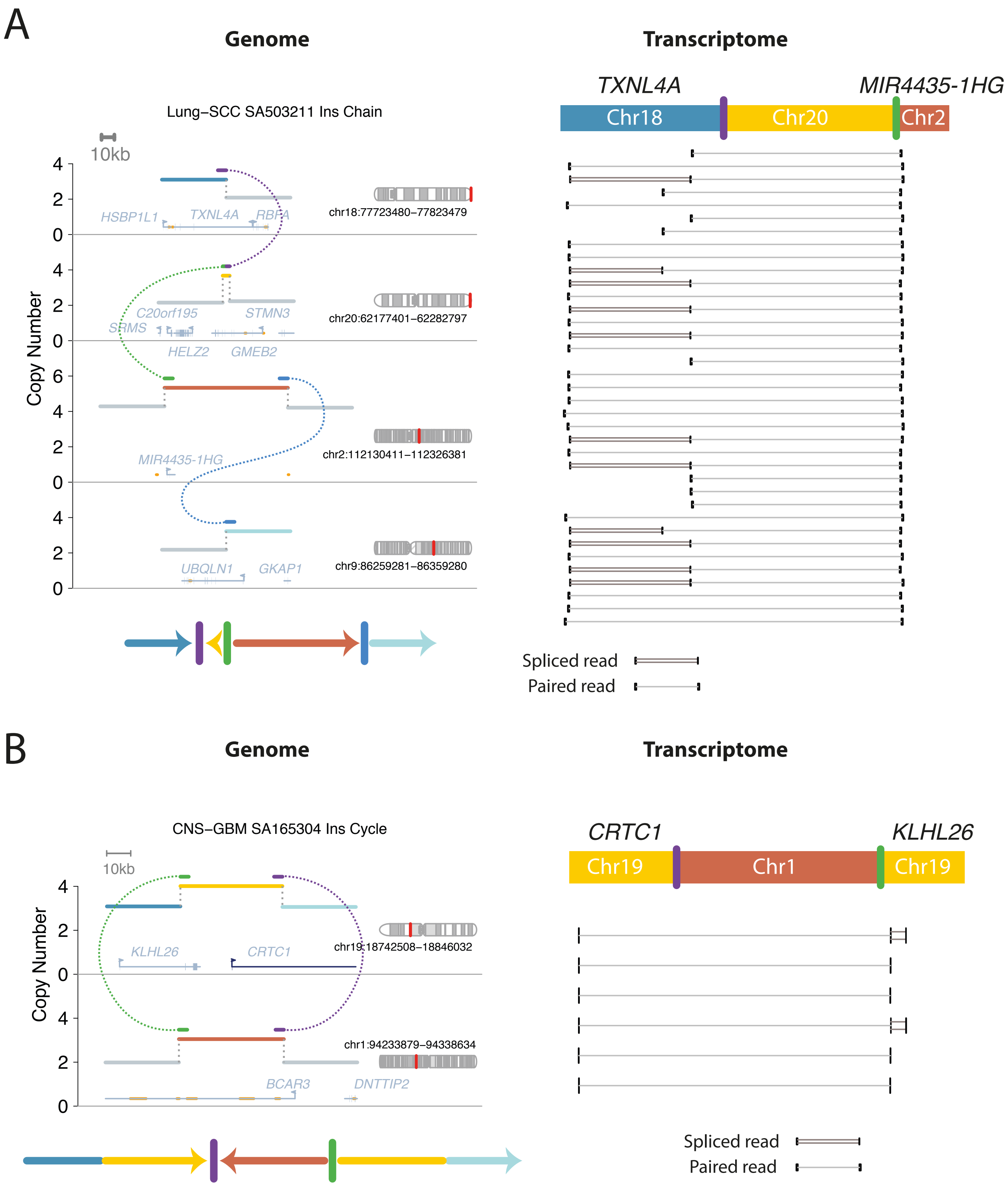

Supplementary Figure 3. Distribution of clonal cell fractions for SVs in the same cluster.

For (A) templated insertions (n=1877 events), (B) local 2-jumps (n=533 events) and (C) chromoplexy (n=136 events), we considered samples with at least two estimated mutation clusters, at least one SV event of the given classification, and at least fifteen BPJs in the sample as a whole. Each SV has a vector denoting probabilities of which clonal/subclonal cancer cell fraction cluster it belongs to. For each pair of SVs in a given patient, we then estimate the cosine similarity between the cluster assignment probability vectors. The histograms show the distribution of quantiles for observed intra-event SVs relative to the distribution calculated for all inter-event SVs in that patient. Under the null hypothesis (that cancer cell fraction of intra-event SVs is a random draw from all SVs in that patient), we would expect the histogram to represent a uniform distribution. The p values are calculated by a one-sided Wilcoxon test against the null hypothesis of the average observed quantile being 0.5 (or less). These data demonstrate that individual breakpoint junctions within a given patient's event are much more likely to share the same clonal fraction than expected by chance, therefore supporting the hypothesis that they occurred simultaneously.

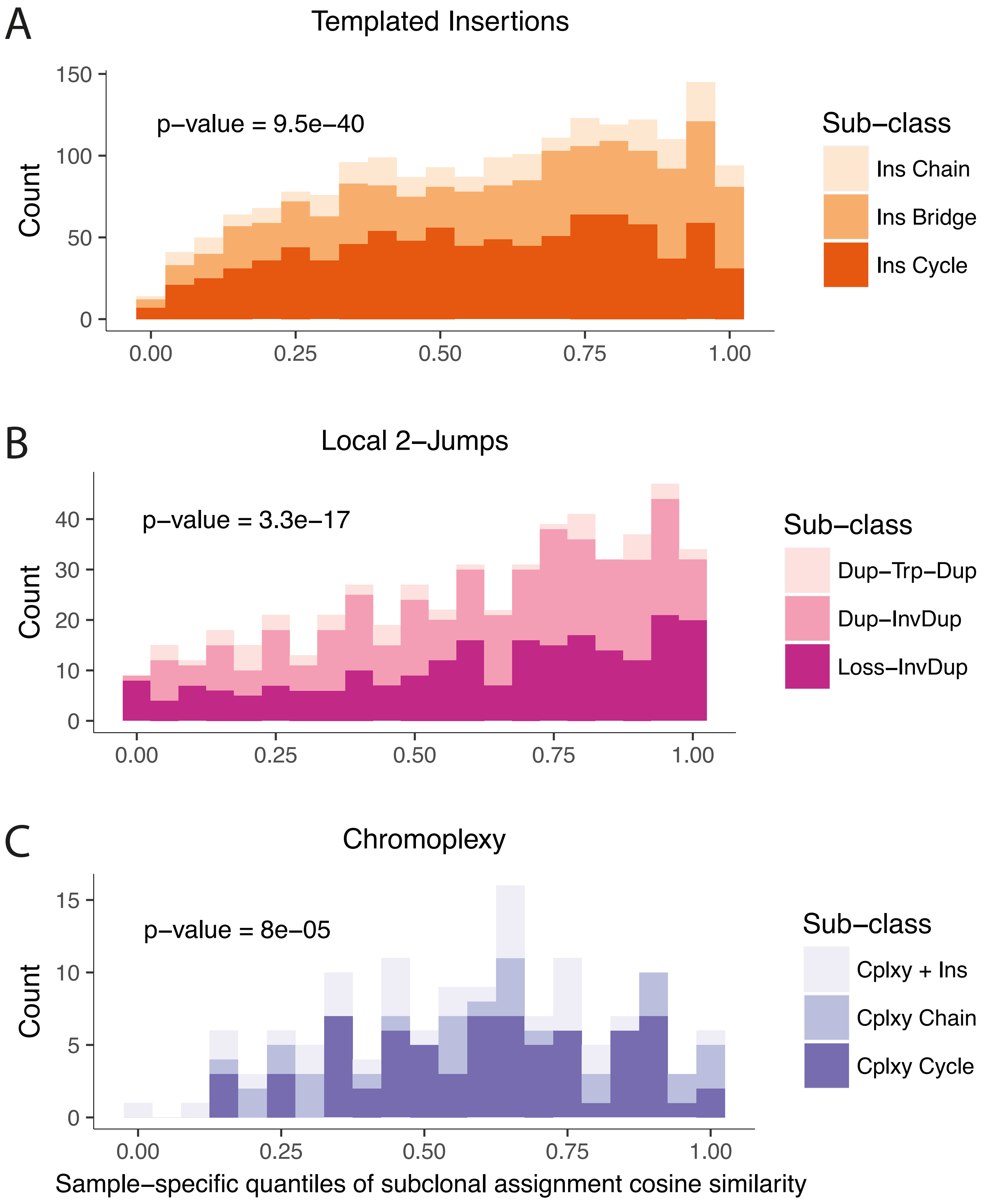

Supplementary Figure 4. Possible alternative routes to generating Dup-invDup structures.

(Figure panels on next page)

There were a number of recurrent footprints in the dataset which solely contained rearrangements confined to one genomic region. Of those comprising two local rearrangements, some had straightforward explanations such as nested or adjacent tandem duplications. Many, however, did not have a trivial explanation (Figure 4A, main text). These included a duplication–inverted-triplication–duplication structure that has been observed in germline SVs (349 instances); a structure of two duplications linked by inverted rearrangements (531 instances); and structures of copy number loss plus nearby duplication linked by inverted rearrangements (472 instances). These patterns all had theoretical solutions recapitulating the observed copy number profiles with breakpoints phased to a single haplotype (Figure 4A, main text), but these configurations could not plausibly be generated by the sequential operation of simple SVs.

To exemplify our reasoning, consider the rearrangement structure of two duplications linked by inverted breakpoint junctions (Figure 4A, main text; left-hand panel). Using our genomic configuration library of all possible sequential SV combinations, we can define four possible routes to this structure (see figure on the next page of ths Supplementary Note). The first is an episomal circle comprising the two amplified segments, but this is an unlikely mechanism because the absence of a centromere leads to random episome segregation at mitosis and instability of copy number per cell. In contrast, most of our examples were at stable, integer copy numbers. The second possible explanation is two foldback rearrangements on different copies of the chromosome, but this cannot explain all instances because linked, inverted duplications were sometimes found in tumours with only one copy of that chromosome. Thirdly, two unbalanced translocations between sister or homologous chromosomes, while formally possible, is unlikely because the average copy number on each side of the event for affected chromosomes is no lower than the rest of the genome on average. Finally, a tandem duplication followed by a reciprocal inversion and then a deletion could create the observed structure, but, if so, we would expect to see many more instances of the intermediate stage of tandem duplication with inversion. In fact, the linked, inverted duplication structure is far more common in this cohort (531 instances) than an inversion within a tandem duplication (33 instances).

Legend for figure on next page.
(A) Two unphased fold-back inversions (through, for example, breakage-fusion-bridge events) could generate the structure. This cannot explain all instances because Dup-invDup events were sometimes found in tumours with only one copy of that chromosome (example in part (E)).
(B) An extrachromomal (episomal) ring comprising the two segments linked by inverted rearrangements would recapitulate the rearrangements, but this is an unlikely mechanism because the absence of a centromere leads to random episome segregation at mitosis and instability of copy number per cell.
(C) A series of unbalanced translocations between duplicated copies of the same chromosome is formally possible, but unlikely because of the close proximity of the rearrangements and stable background copy number of the chromosome.
(D) A tandem duplication, followed by inversion, followed by deletion could generate the structure, but, if so, we would expect to see many more instances of the intermediate stage of tandem duplication with inversion. In fact, the Dup-invDup is far more common in this cohort (531 instances) than an inversion within a tandem duplication (33 instances).
(E) An example of such an event in a stomach cancer, occurring on the background of a single copy of the relevant chromosome arm. That this occurs within a single copy implies that the two inverted rearrangements must be phased, excluding the two fold-back inversion structure shown in (A).

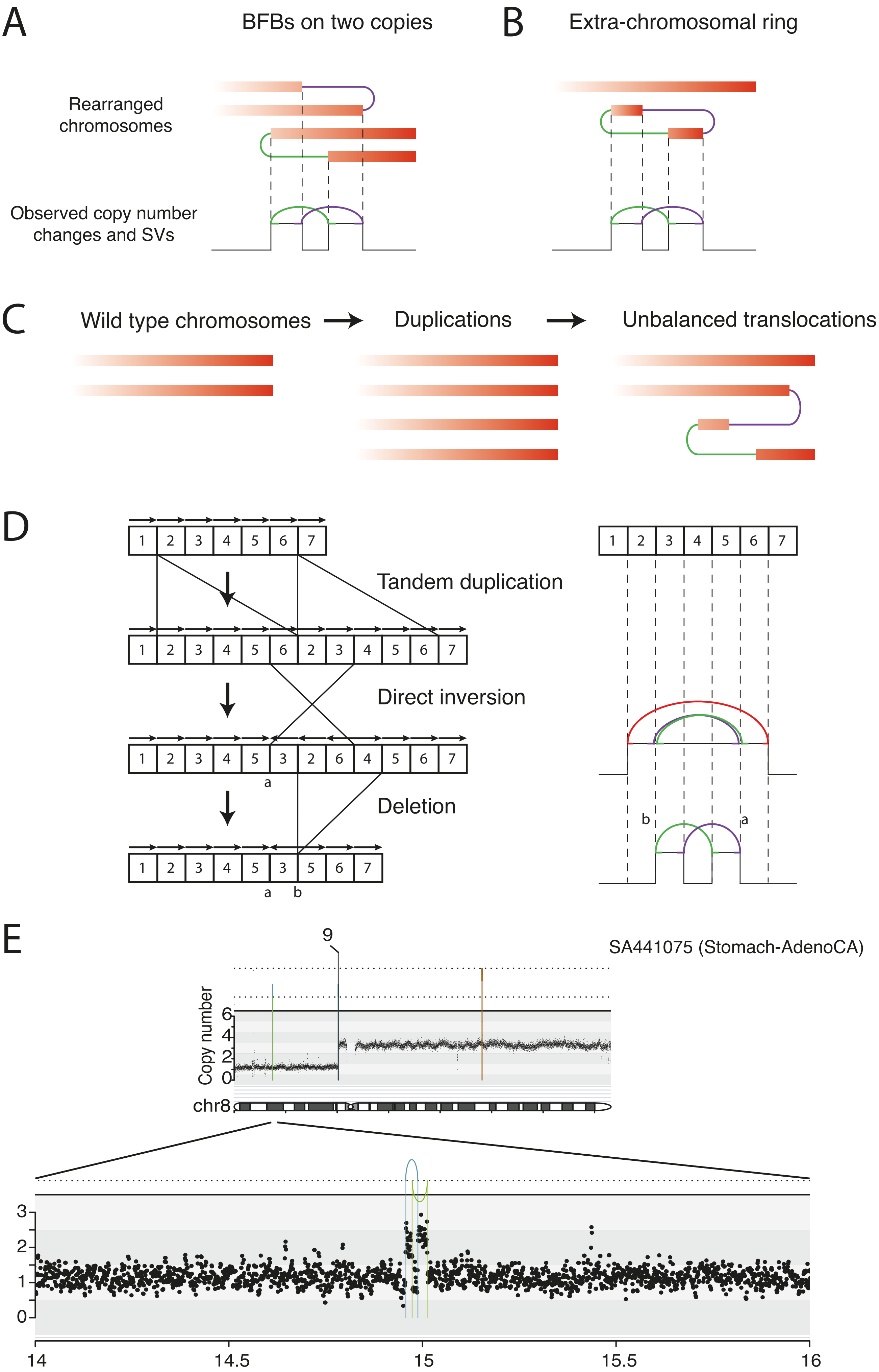
A
BFBs on two copies
B
Extra-chromosomal ring
Rearranged chromosomes
Observed copy number changes and SVs
C
Wild type chromosomes
Duplications
Unbalanced translocations
D
Tandem duplication
Direct inversion
Deletion
a
b
E
9
SA441075 (Stomach-AdenoCA)
Copy number
chr8
14
14.5
15
15.5
16

Supplementary Figure 5. Clusters of patients with distinct profiles of replication timing.
(A) Patients with high numbers of tandem duplications (number per patient shown in upper right corner of each density distribution) were clustered according to the profile of replication timing. Five clusters were identified, with numbers of patients in each cluster shown in the facet label. Individual density distributions of up to 8 representative patients in each cluster are shown in each column.
(B) Clustering of replication timing profiles for deletions, shown as for panel (A).

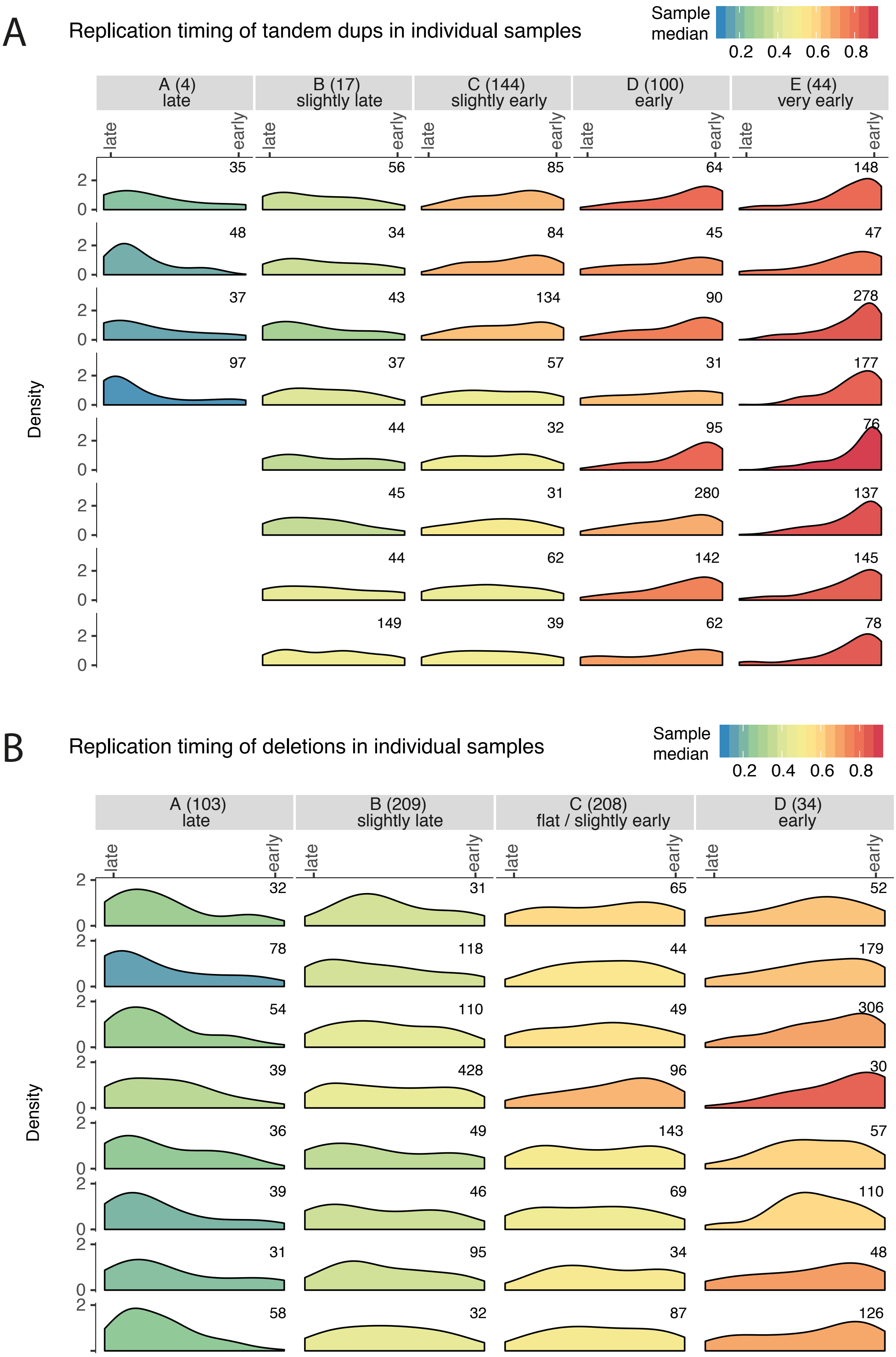

Supplementary Figure 6. Features of segments at breakpoint junctions. (A) Distribution of sequence homology between the ends joined at a breakpoint junction (y axis is on a log scale). The area shaded in orange represents the expected frequency of homology occurring by chance between two joined DNA sequences, were homology not relevant to the repair process. The area shaded in blue represents observed rearrangements that exceed the expected distribution of homology.
(B) Enrichment or depletion of breakpoint junctions between regions of the genome with particular annotations, compared with a permuted background that preserves breakpoint positions but swaps breakpoint partners. Centre points are the mean fold-change over the permuted background; error bars represent three standard deviations. Analysis is based on a sample size of 2,559 genomes containing SVs. Complex uncl., complex clusters unclassified; LTR, long terminal repeat; SINE, short interspersed nuclear element; LINE, long interspersed nuclear element; ENH, enhancer; heterochrom, heterochromatin.
(C) Breakpoint microhomology for rearrangements connecting repetitive elements of the same class (green) or rearrangements with only one breakpoint in a repetitive element (orange). Analysis is based on a sample size of 2,559 genomes containing SVs. The box shows the median level of microhomology as a thick black line, with the box's range denoting the interquartile range. The whiskers show the range of data or 1.5x the interquartile range, whichever is lesser. Comparisons with four stars indicate p<0.0001; two-sided t-test.

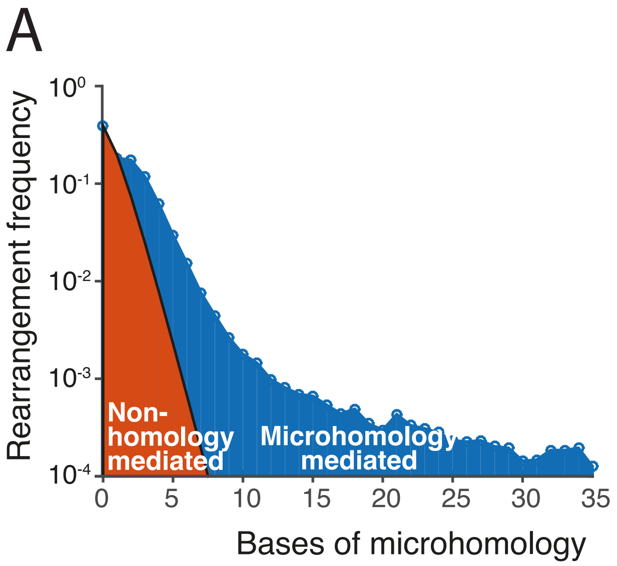

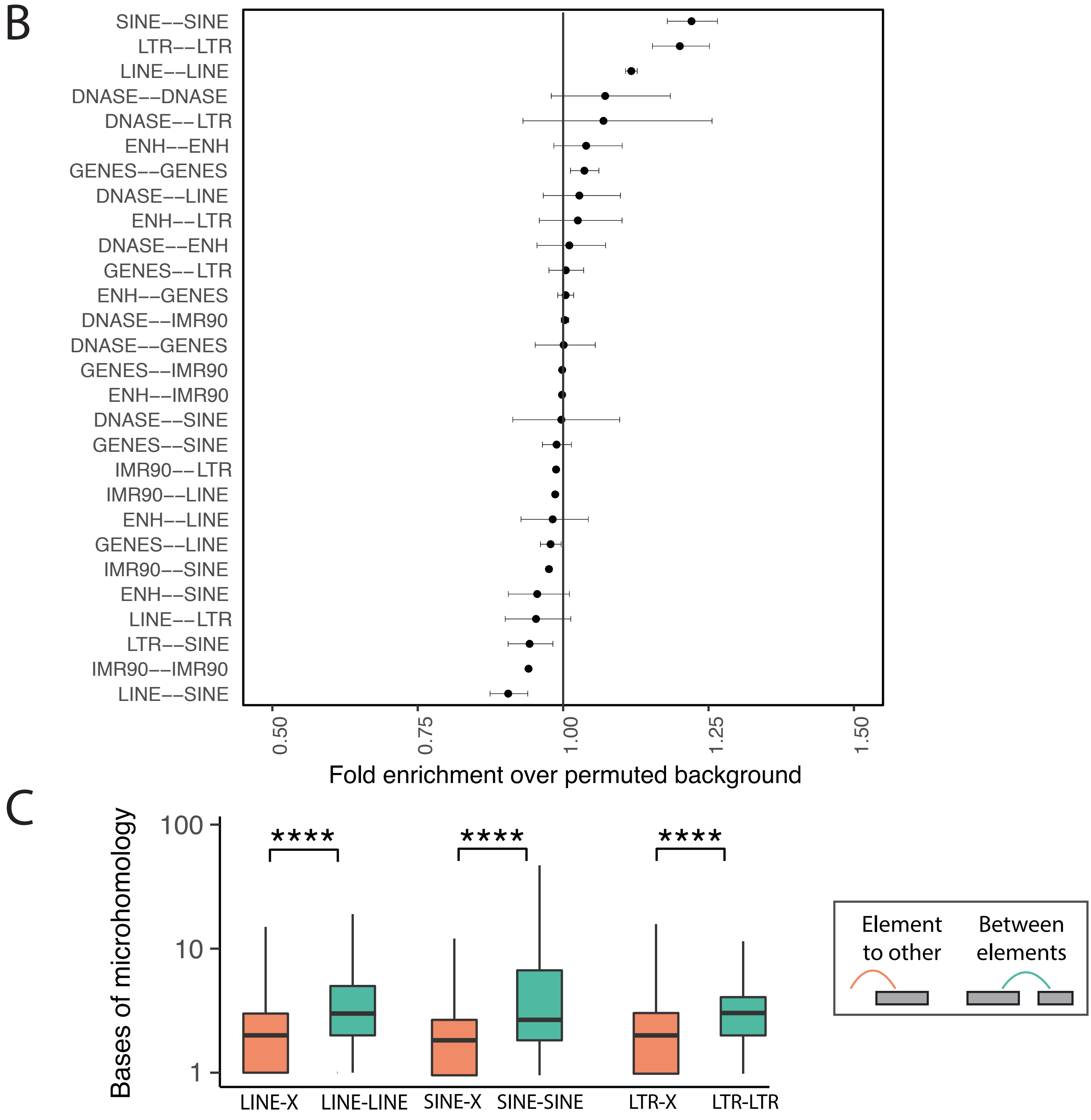

Supplementary Figure 7. SV signatures extracted from a random split of the cohort into two equal-sized halves.
The PCAWG cohort was randomly split into two halves and signatures extracted independently on each. Ten signatures showed strong concordance between the two halves (red and cyan) and indeed the full cohort.

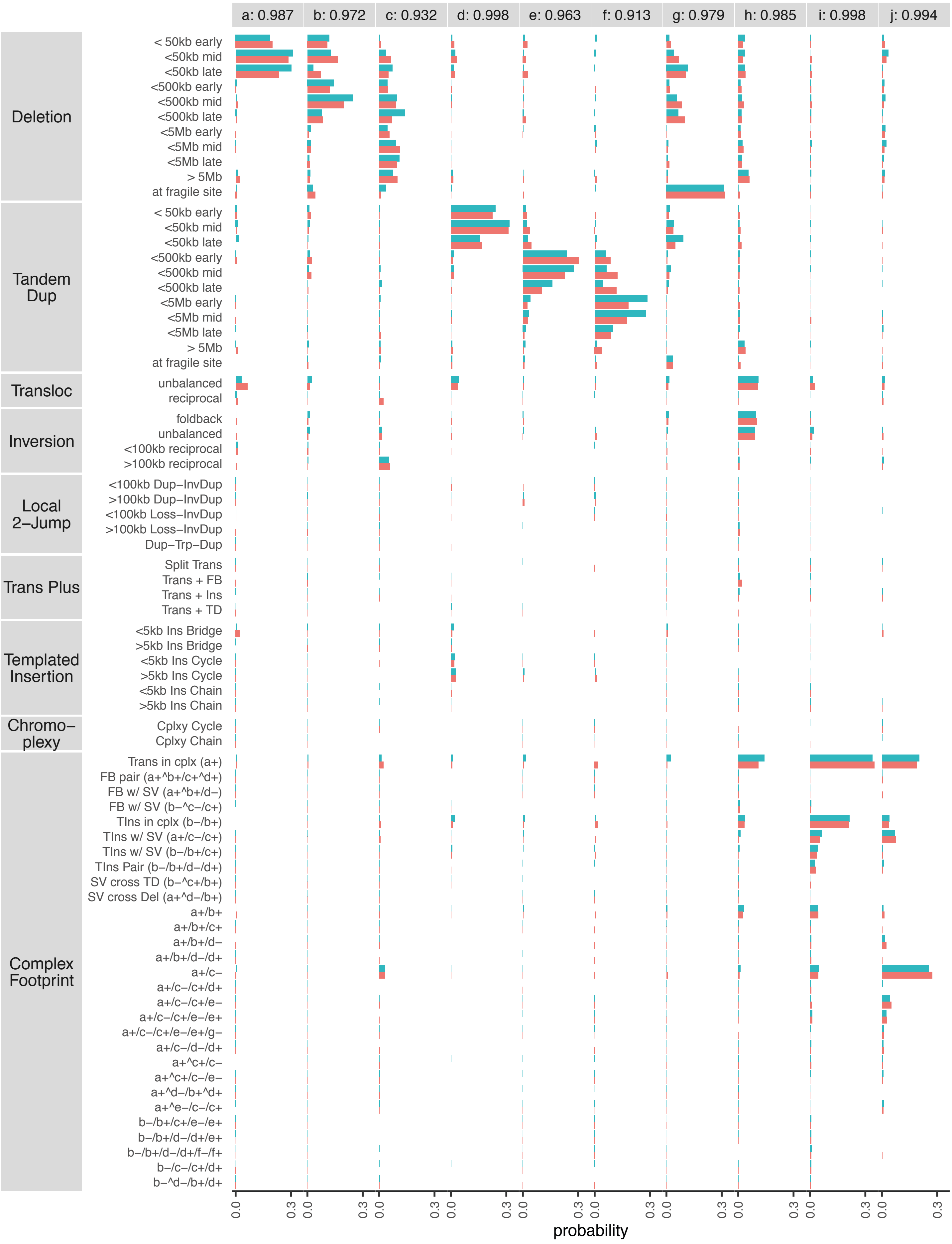

Supplementary Figure 8. Distribution of SV signatures across tumour types.
Per-sample signature contributions across different tumour types in PCAWG. Within each tumour type, patients are ordered along the x axis by the total number of SV events they have (grey bar charts). The bottom panel for each tumour type is a stacked bar chart showing the proportion of those SV events attributed to each of the SV signatures identified.

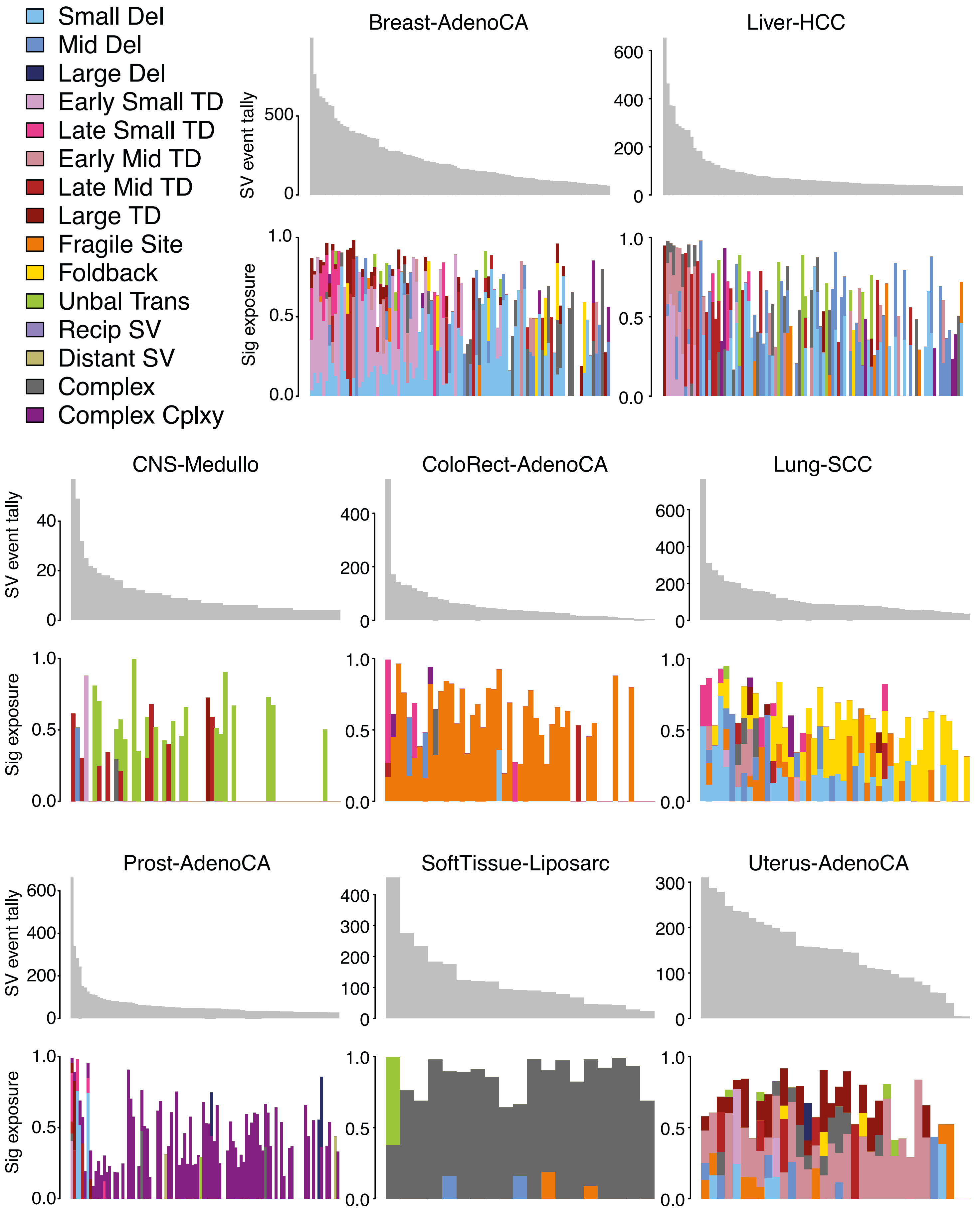

Supplementary Methods

Terminology and definitions

This section will introduce and define some concepts and terms used in the remainder of the supplementary materials.

Telomere and centromere copy number

Telomere copy numbers are defined as the median copy number of the first 1Mb from the telomere after the telomeric assembly gap in GRCh37. Centromere copy numbers are defined as the median copy number of the first 1Mb from the centromere assembly gap in GRCh37. Centromeres are separated into two chromosomal arms: p-arm and q-arm centromere copy number are estimated using the 1Mb closest to the centromeric gap from the p-arm and the q-arm, respectively (Supplementary Figure 9).

Because of how telomere and centromere copy numbers are defined, they are not necessarily the same. For example, an unbalanced translocation will cause the loss of a telomere or a centromere. Even when the telomere and centromere copy numbers of a chromosomal arm are the same, there can still be interstitial copy number changes in a chromosomal arm. A chromosomal arm is considered *stable* if all the following conditions are true.

- The respective telomere and centromere copy numbers are within 0.5 of each other.
- The 0.2 and 0.8 quantiles of the copy number on the chromosomal arm are within 0.5 copies from the average copy number of the respective telomere and centromere.

For example, in Supplementary Figure 9, the P-arm centromere and telomere CN are both at ~3.5. However, the 0.2 quantile of the copy number is at ~2.5, caused by the large deletion, and therefore the P-arm is not considered stable. In the Q arm, the centromere CN is different to the telomere CN, and therefore the Q arm is also not considered stable.

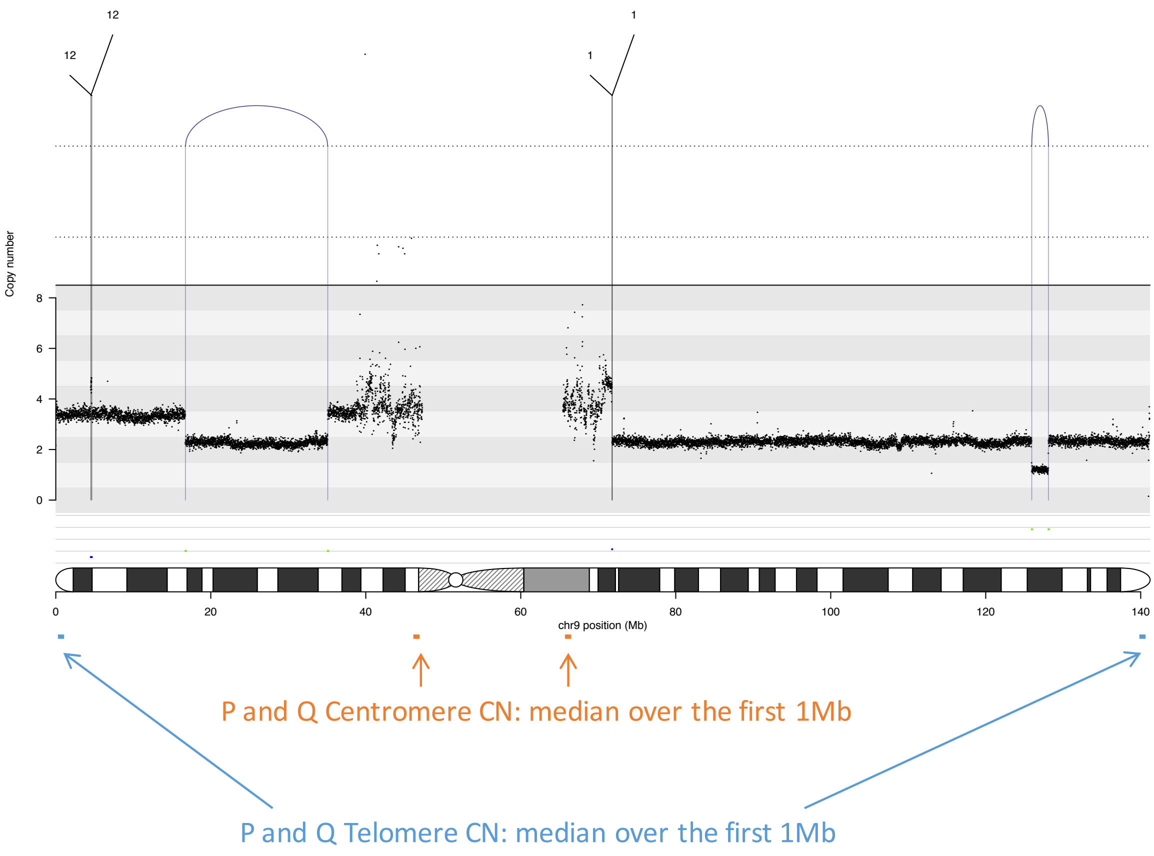

Supplementary Figure 9: Centromere and telomere copy numbers. P and Q telomere copy numbers are defined as the median copy number in the 1Mb closest to the respective telomere assembly gap. P and Q-arm centromere copy number is defined as the median copy number in the first 1Mb on the P and Q-arm side of the centromere assembly gap, respectively.

Rearrangement end

A rearrangement end is one of two breakpoints of an SV junction. Rearrangement ends are the genomic locations and orientations through which a rearrangement links two genomic loci together.

The *low end* of an SV is the one with a lower chromosome name (lexicographically) or chromosomal position (Supplementary Figure 10).

By definition, an SV indicates that a chromosome is broken from one rearrangement end of the SV and joined to the locus of the second rearrangement end. At a break, the side with a rearrangement end is termed *rearrangement side*, as that is the side that participates in the rearrangement. The other side of a break that is not linked in the SV is called *non-rearrangement side* (Supplementary Figure 10).

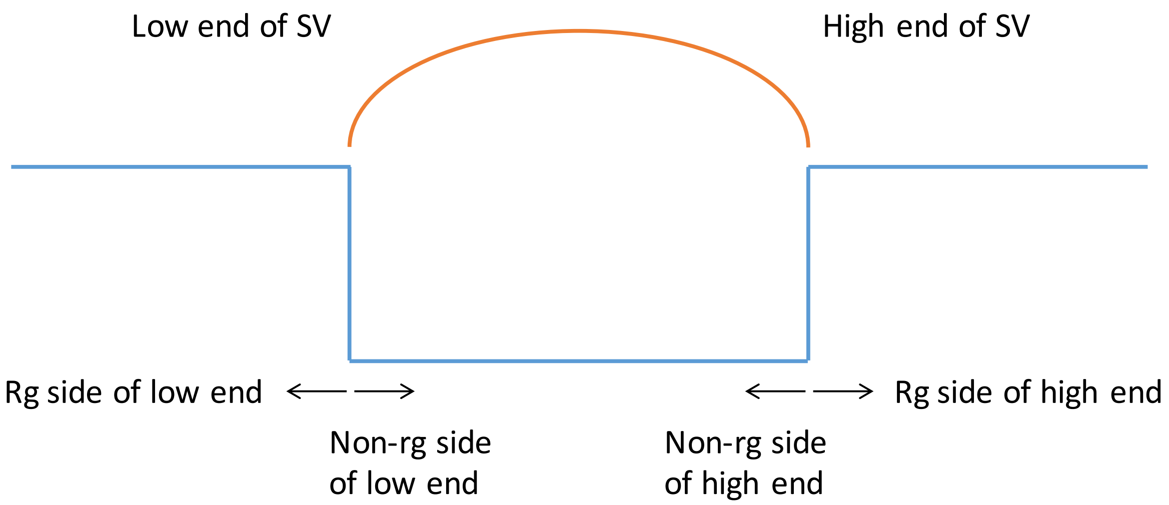

Supplementary Figure 10: Definition of rearrangement and non-rearrangement side, and definition of low and high ends of SVs. The arc shows the SV breakpoint junction, the blue lines show the copy number in the local region.

Copy numbers associated with a rearrangement end

Rearrangement side and non-rearrangement side copy number of a rearrangement end is defined as the copy number of the segment on the rearrangement side and non-rearrangement side of the SV, respectively.

Rearrangement patterns

Much of the supplementary materials will discuss different constellations of SVs and their associated copy number segments. We use the term 'rearrangement pattern' to refer to either a set of mechanistically or positionally connected SVs, or the combination of both SVs and their copy numbers. Several simple rearrangement patterns and trivial rearrangement mechanisms generating them are illustrated in Supplementary Figure 11.

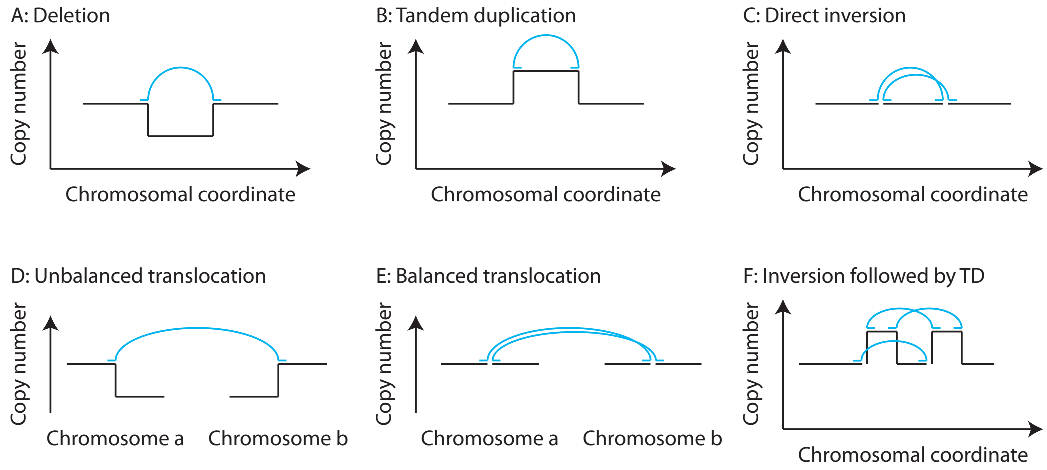

Supplementary Figure 11: Examples of simple rearrangement patterns. Rearrangement patterns are schematic models of the observed SV and copy number information through paired-end whole-genome sequencing. Deletions (A), tandem duplications (B), direct inversions (C), unbalanced translocations (D), balanced translocations (E) all have their characteristic rearrangement can copy number pattern. Panel F shows an example of a pattern generated through two overlapping rearrangements, a direct inversion followed by a tandem duplication that overlaps with one of the inversion breakpoints. Here the two rearrangements that are balanced were part of the initial inversion, and the tandem duplication manifests as the unbalanced rearrangement in the inverted orientation (both rearrangement ends are in + orientation). This is not expected from a simple tandem duplication (B), which should have a minus-orientation breakpoint followed by a plus-orientation breakpoint. Instead, in the secondary tandem duplication one of the breakpoints got inverted because it occurred on the chromosomal segment that was inverted in the initial direct inversion event.

Copy number relative to chromosomal arm

Due to genomic instability, chromosomes in tumour cells can have differing ploidies. In order to study the copy number consequence of SVs, the background copy number of the chromosome on which the SVs occurred must be estimated accurately.

The background copy number level of a rearrangement end is defined based on its orientation. If the rearrangement side of a rearrangement end is oriented towards a telomere (centromere), then its background copy number is defined as the copy number of the telomere (centromere) it is oriented towards.

In other words, forward (+) and reverse (-) oriented rearrangement ends on the P-arm of a chromosome have their background copy number estimated using the P-arm telomere and centromere, respectively. Similarly, background copy numbers for forward and reverse oriented rearrangement ends on the Q-arm are estimated from the Q-arm centromere and telomere, respectively.

An object-oriented framework for handling somatic SV and CNA data

Due to the intricate relationship between SVs, their rearrangement ends and copy number segment breakpoints, they often have to be considered together in somatic SV analysis. We developed an object-oriented (OO) framework in Perl in order to facilitate the handling of copy number and rearrangement data jointly. The overarching purpose of the framework is to have a powerful way of detecting, managing and analysing rearrangement patterns such as those presented in Supplementary Figure 11.

A common task is to estimate the copy number of an SV. This should be estimated from the observed CN changes at two rearrangement ends of the SV, weighted by their relative uncertainty. The CN change of a rearrangement end in turn derives from the CN difference between the two CN segments demarcated by it. The uncertainty of this CN change depends on the lengths of the two involved CN segments. Thanks to the OO framework linking these different abstract concepts together, obtaining the needed segment CN and length values for a given SV becomes a much more tractable task.

A more challenging task is to compute the normalised rearrangement pattern for rearrangement clusters, which involves reordering and permuting footprints in order to obtain the lexicographically smallest string representation of the SV cluster (see details below). Without a flexible way of navigating between CN segments, rearrangement ends, clusters and footprints, computing normalised rearrangement patterns would have been a daunting task.

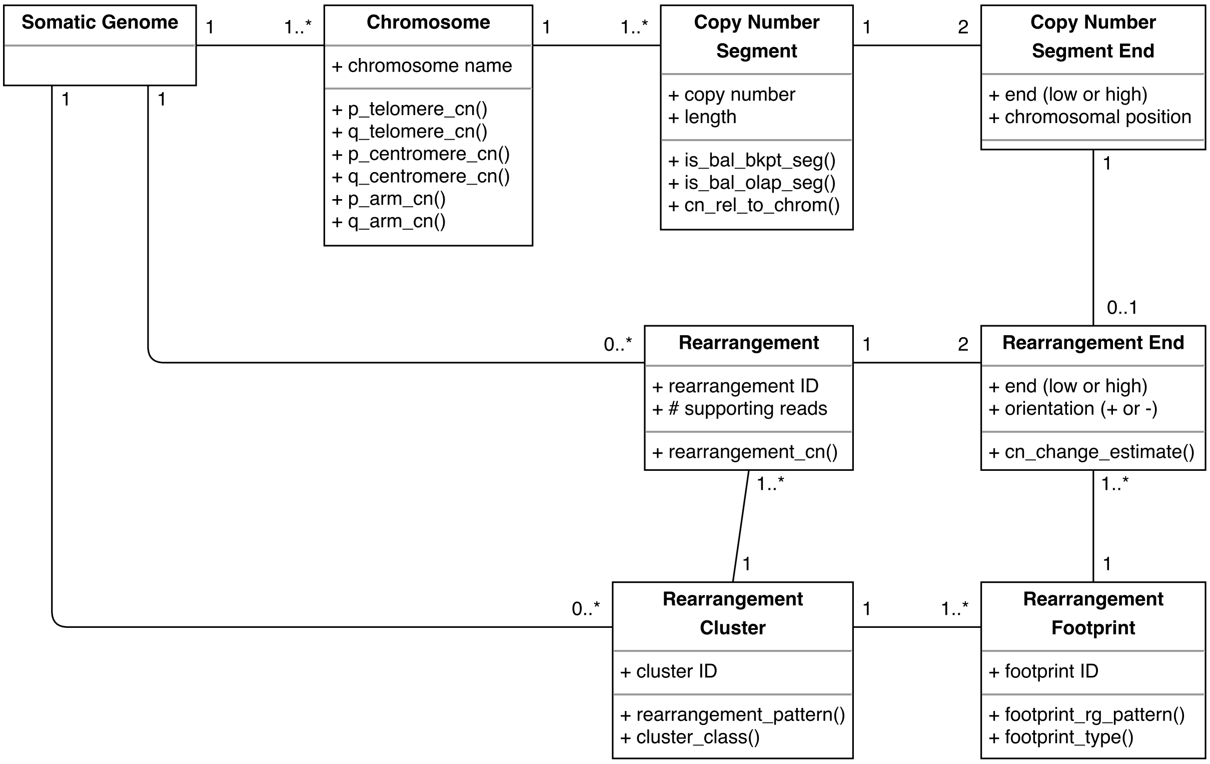

Supplementary Figure 12: The object-oriented framework for handling somatic rearrangements and copy number segments. The figure highlights main classes, their relationships and their key attributes and example methods.

The SV clustering and classification pipeline

Rearrangement events often generate multiple junctions. A direct inversion generates two balanced inversion-type SVs, chromoplexy generates a chain or a cycle of balanced breakpoints and chromothripsis can generates hundreds of SVs at once. Mechanistic inference of SV junctions has to be done considering all involved junctions jointly. For example, what differentiates a fold-back inversion SV generated as part of a breakage-fusion-bridge cycle from an inversion SV generated as part of a direct inversion is the fact that the direct inversion SVs are balanced. Similarly, the chaotic break and join process of a chromothripsis event could generate dozens of SVs that on face value are consistent with simple deletions or tandem duplications when interpreted individually.

A rearrangement event can involve multiple loci in the genome. For example, we previously reported an instance of a BFB-associated fold-back inversion that had an intervening templated insertion on a different chromosome[1]. Again, to correctly interpret the nature of these sub-cluster level events, all related SV junctions must be considered simultaneously. For example, templated insertions involve two rearrangement ends with their *rearrangement sides* oriented towards each other. Were these rearrangement ends considered independently, they would support two independent unbalanced translocations instead. Therefore, in addition to grouping SVs into clusters that imply *temporal or mechanistic association* between the SVs, the involved rearrangement ends must also be clustered into footprints that imply *positional association*.

Motivated by these challenges in interpreting individual SVs, we developed a method for grouping SVs into clusters and footprints in order to allow structural and mechanistic inferences to be made systematically. In parallel, we process the somatic CN data and merge

it with SV junctions in order to allow us produce rearrangement patterns from the generated SV clusters and footprints. We produce normalised representations of SV cluster patterns, which allows us to tabulate the number of different cluster and footprint patterns and analyse their features. Finally, we performed manual and simulation-assisted interpretation of the recurrently observed cluster and footprint patterns.

The individual steps of the SV classification pipeline are outlined below and detailed in the subsequent subsections.

1. Computing exact breakpoint coordinates from clipped reads.
2. Removing redundant "segment-bypassing" SVs.
3. Merging rearrangement breakpoints with copy number data to yield SV breakpoint-demarcated normalized absolute copy number data.
4. Clustering individual SVs into SV clusters and footprints
5. Heuristically refining SV clusters and footprints
6. Filtering artefactual fold-back-type SVs with insufficient support
7. Determining balanced overlapping breakpoints. This step is to distinguish very short templated insertions from mutually overlapping balanced breakpoints.
8. Computing rearrangement patterns and categories

1. Computing exact breakpoint coordinates from clipped reads

Exact breakpoints enable more accurate copy number estimation, in particular when breakpoints are clustered and the involved copy number segments are very short.

The raw read data in this project was aligned using BWA MEM, which is able align reads partially by soft-clipping them[2]. This feature is particularly useful around rearrangement breakpoints, where partially mapped reads soft-clipped at the exactly same genomic base can often be seen (Supplementary Figure 13). The most straightforward interpretation of these clipped reads is that the reads were aligned to the region until they split into the partner region of the SV. Thus, the reference genome positions of soft-clipped reads can be used to infer exact breakpoints of rearrangement ends. Moreover, short segments are expected to harbour soft-clipped reads on both of its breakpoints. This feature is sometimes crucial in differentiating between templated insertions and balanced breakpoints with extended homology (Supplementary Figures 13-14).

We wrote a Perl script for computing the absolute breakpoints of each rearrangement by looking at the presence of clipped reads at both sides of a read group (i.e. one side of a read pair group that supports a given SV). If there are clipped reads, the exact breakpoint position is inferred from the median of read soft-clipping positions. Otherwise, the alignment position of the read furthers into the breakpoint is used instead (Supplementary Figure 13).

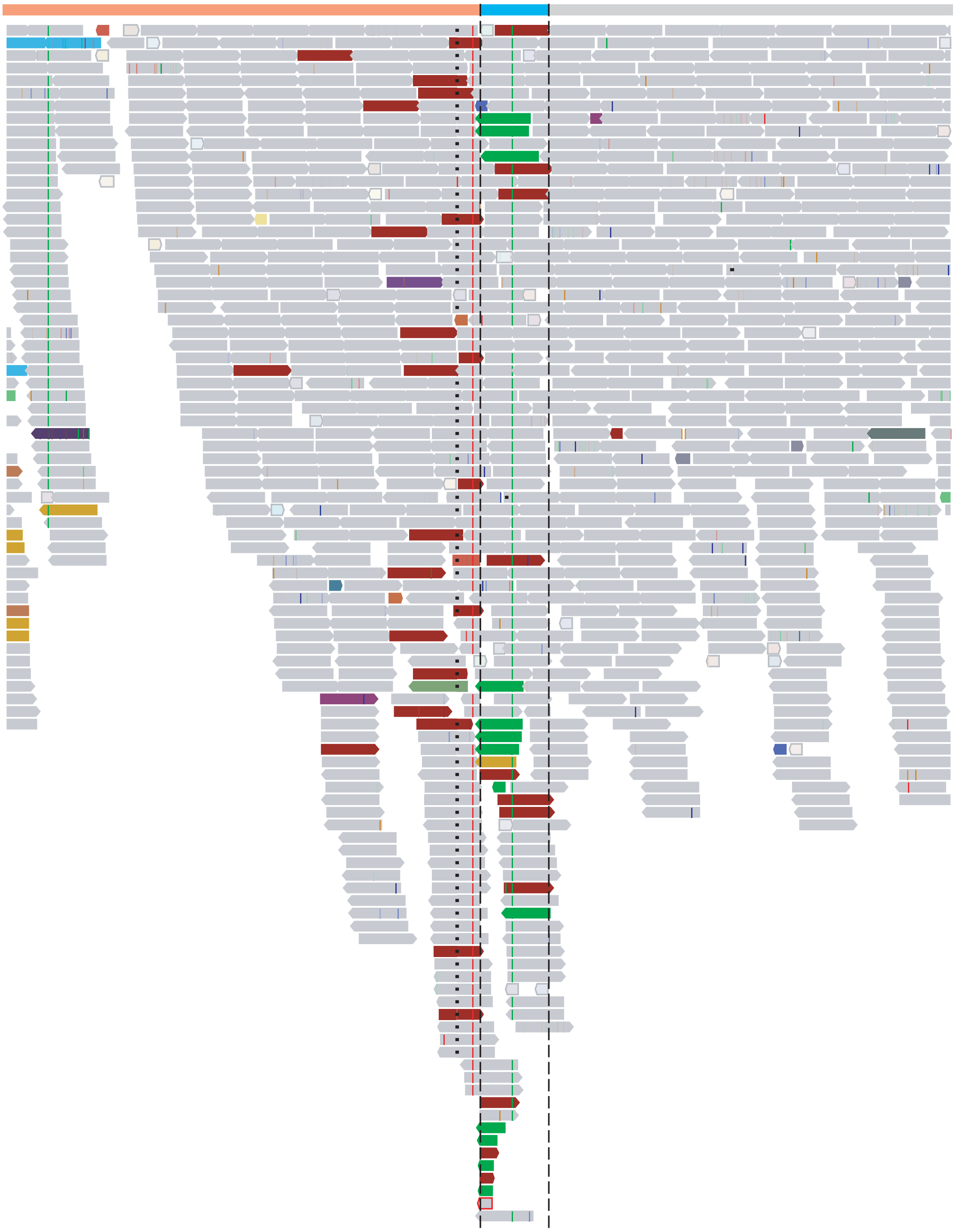

Supplementary Figure 13: Estimation of exact rearrangement breakpoint from clipped reads. The IGV screenshot shows a genomic region with the low end of three rearrangement calls, two of deletion type (supported by red reads) and one of tandem duplication type (supported by green reads). There are two rearrangement breakpoints as shown by the vertical dashed lines going across the entire figure. In the left-most rearrangement supported by the '+' orientation red reads in the region under the orange bar on top, there are multiple reads whose alignment end with a soft-clip precisely at the first breakpoint. Similarly, the rearrangement breakpoint supported by the '-' orientation green reads also has several reads whose alignment end right at the same breakpoint. This indicates that the first and the second rearrangements share a balanced breakpoint. The third rearrangement, which involves the same segment as the green reads, indicated by a blue bar on top, has a breakpoint at the second dashed line as evidenced by the soft-clipped red reads in this region. Note that in the blue segment, some of the red reads are not clipped from the 3'-end, but from the 5'-end at the same breakpoint as the soft-clipped green reads. This observation is crucial as explained in Supplementary Figure 14, because the 5'-end soft-clipping of the red reads in the blue region indicates that this chromosomal segment does not extend into the orange region, but there is another SV junction at the left side end of the blue segment. Indeed, this is supported by the rearrangement of the green reads, and indicates that the two rearrangements junctions demarcating the blue region are phased together.

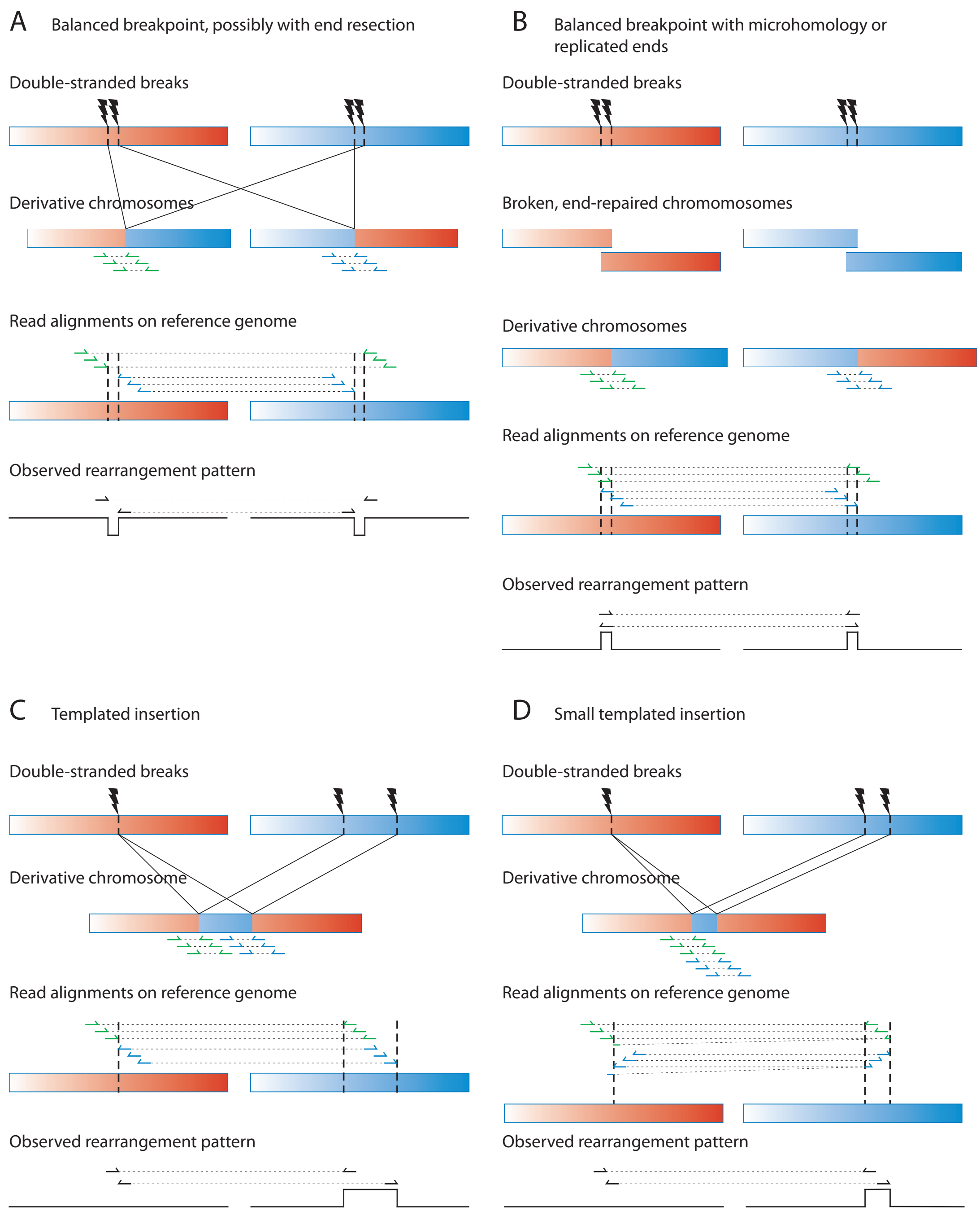
A
Balanced breakpoint, possibly with end resection
Double-stranded breaks
Derivative chromosomes
Read alignments on reference genome
Observed rearrangement pattern
B
Balanced breakpoint with microhomology or replicated ends
Double-stranded breaks
Broken, end-repaired chromosomes
Derivative chromosomes
Read alignments on reference genome
Observed rearrangement pattern
C
Templated insertion
Double-stranded breaks
Derivative chromosome
Read alignments on reference genome
Observed rearrangement pattern
D
Small templated insertion
Double-stranded breaks
Derivative chromosome
Read alignments on reference genome
Observed rearrangement pattern

Supplementary Figure 14: Distinguishing balanced breakpoints and templated insertions using read clipping patterns. In each panel, rectangles with blue and red gradient represent two different chromosomes. Lightning symbols denote double-stranded breaks. Blue and red arrows joined by dotted lines are read pairs derived from the breakpoint regions. A: In a typical balanced rearrangement breakpoint with or without end resection but without homology-mediated end filling, the non-rearrangement ends of the balanced breakpoints are oriented towards each other. When there is end resection at the breakpoints, a small section of deletion will be created between the breakpoints. B: Sometimes with homology-mediated double-stranded break repair, there may be a net gain of DNA material as, supposedly, homology-mediated replication replicates DNA on both breakpoints. If this happens, the breakpoints of the two rearrangements, as indicated by the supporting reads nearest to the breakpoints, may actually extend beyond each other, resulting in a small segment with ostensible copy number gain. C: In a templated insertion that is much larger than the read length, the rearrangement-side of both rearrangements point towards each other demarcating the gained segment that is inserted. D: When a templated insertion is very short, the rearrangement pattern at the insertion footprint can be very similar to what is observed in a balanced breakpoint with homology-mediated end filling (B). However, the two cases can be distinguished by the fact that in a balanced breakpoint, the rearrangement ends of the supporting reads are never soft-clipped. In contrast, in a small templated insertion, reads from both orientations will be soft-clipped on both ends of the inserted segment.

2. Removing redundant "segment-bypassing" SVs

Occasionally templated insertions can be much shorter than the average insert size of a sequencing library. In such cases read pairs can frequently bypass short templated insertions, with individual reads mapping to the rearrangement partner on either side of the templated insertion but not on the templated insertion itself (Supplementary Figure 15). When this happens, a cluster of read pairs supporting a redundant rearrangement may be called. Such a rearrangement call is misleading because there is no direct contact between the genomic regions on the two ends of the supporting read pairs. Instead the genomic contact happens through the templated insertion (Supplementary Figure 15).

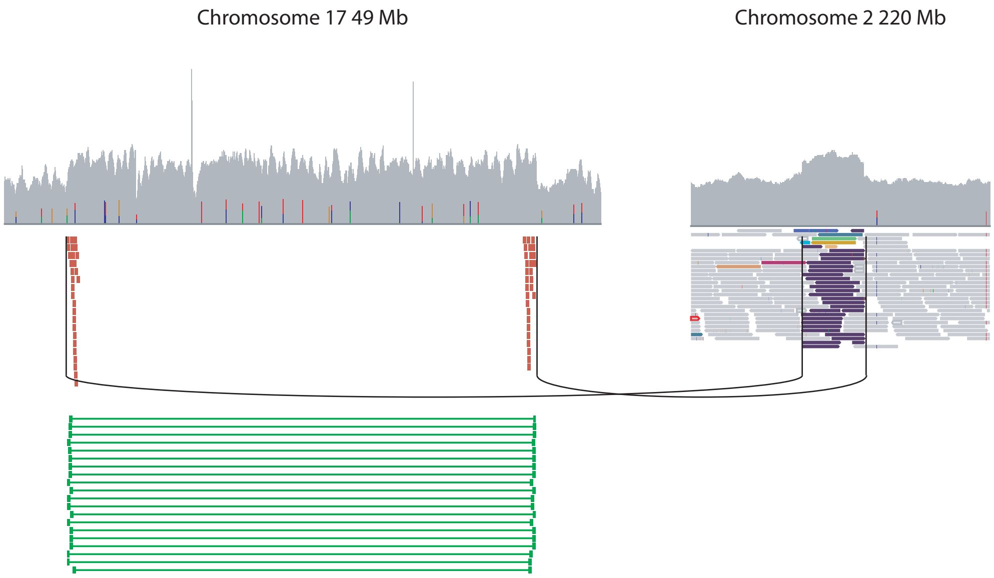

Supplementary Figure 15: Segment-bypassing rearrangements. Two chromosomal regions of an example case are shown. The grey density plot indicates the overall coverage at the two regions. At chromosome 17, relevant abnormally paired read pairs are shown. On chromosome 2, both properly mapped (grey) and abnormally mapped (purple) read pairs are shown. In this example, there is a copy number gained region at chromosome 17. This gain is associated with a small inserted region derived from chromosome 2. Two read groups, two clusters of red reads on chr17 and two clusters of purple reads on chr2, link the duplicated region with the insertion region. However, the chromosome 17 region also has a cluster of tandem-duplication-type read pairs (green) spanning the duplicated region. These are read pairs that completely bypass the small inserted region on chromosome 2. Thus, the rearrangement call supported by the tandem duplication-type read pairs are bypassing the inserted segment derived from the chromosome 2.

We detect and remove segment-bypassing SVs using the following procedure. We first look for an SV's low end's "neighbouring rearrangement ends", which have the same orientation as and are within 200bp of the SV's low end. Then, for each neighbouring rearrangement end that has a mate on a templated insertion (≤ 10,000kb), then we "jump" to the second rearrangement end of the templated insertion. We continue jumping until a rearrangement end's mate is not on a templated insertion. If at this point we reach a neighbouring rearrangement end of the high end of our initial SV, then we deem that the initial SV is segment-bypassing and remove it. Note that the large templated insertion size cutoff here does not matter, since in order for an SV to be segment-bypassing, the total length of all the bypassed segments have to be less than insert size. Thus, a large cutoff just ensures that this algorithm works regardless of the sample's insert size.

3. Merging rearrangement breakpoints with copy number data

Sample-specific ploidy and purity estimates were obtained using ascatNgs[3]. Other outputs of ascatNgs were not used. Instead, somatic copy number levels were estimated as described below.

Reference genome was divided into non-overlapping 500 base pair windows. Per-window coverage log ratios were computed by dividing the tumour read depths by their respective read depths in the matching normal sample. A pseudocount of 0.1 was added to the both the tumour and matching normal per-window read depths.

GC-content is a known covariate to read depth in many samples, and is generally corrected for in somatic copy number estimation. In our analysis, we noticed that the rate of local inverted read pairs (++ or -- read pairs with distance <5kb) also covaried with read depth independent of GC-content (Supplementary Figure 16). The high frequency of such read pairs probably reflect a previously reported of Illumina library preparation artefact[4].

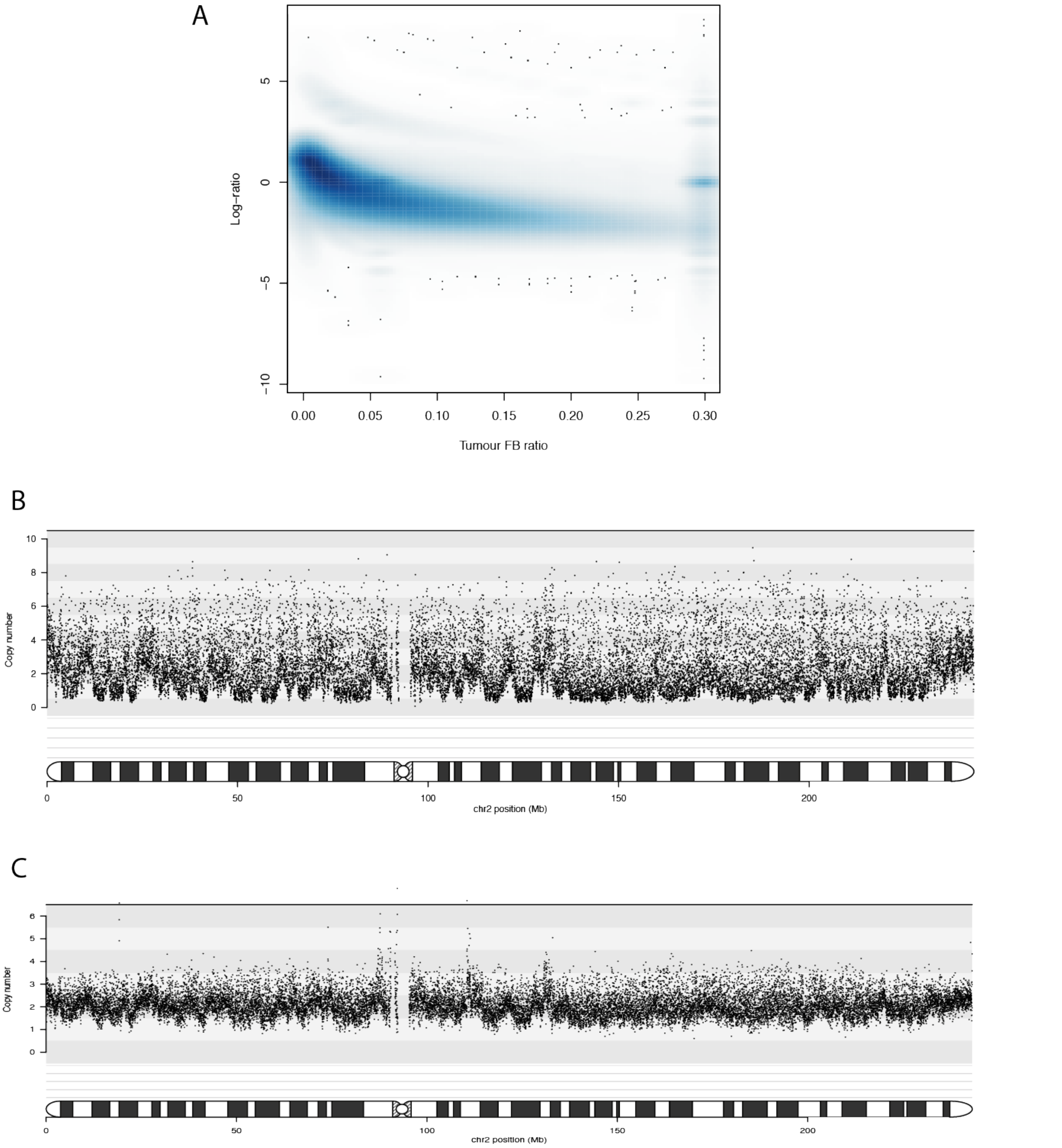

Supplementary Figure 16: Normalisation of read depth using fold-back read pair density. The data shown are based on a sample with high frequency of fold-back read pair artefacts. Panel A shows the density plot of per-window fold-back read pair density against $\log_2$(tumour/normal) copy number ratio. The fold-back read pair density is computed as the number of fold-back type reads divided by the total read coverage of each window and capped at 0.3. Note that the association between fold-back read pair density and copy number is not linear in this sample. Panel B and C show the copy number estimates obtained by statistically correcting the $\log_2$-ratio data using GC-content alone (B) or with both GC-content and fold-back read pair density (C).

Samples with a high number of fold-back inversion read pairs had an extremely high variance in their read depth (Supplementary Figure 16). Given the association between fold-back inversion read pair density and overall read depth, we thus used a generalised additive model (GAM) to model the additive but non-linear read depth effect of both GC content and fold-back read pair density. GAM is a multi-dimensional generalisation to single-variable non-linear regression such as loess, which can be more appropriate than purely linear regression-

based normalisation (Supplementary Figure 16). In certain samples, somatic copy number estimates stabilised significantly when fold-back read pair rate was factored in (Supplementary Figure 16).

Normalised per-window $\log_2$-ratio values were segmented using the R package 'copynumber'[5] with a 'gamma' parameter of value 200. Per-window and per-segment absolute copy number estimates were computed from the normalised $\log_2$-ratio values using the following formula.

$$\begin{aligned}\text{purity-adjusted log-ratio} \\ &= \text{normalised log-ratio} - \text{median}(\text{normalised log-ratio}) \\ &+ \log_2((\text{purity} \times \text{ploidy} + (1 - \text{purity}) \times 2)/2)\end{aligned}$$

$$\text{absolute CN} = (2^{1+\text{purity-adjusted log-ratio}} - 2 \times (1 - \text{purity}))/\text{purity}$$

The copy number segmentation and rearrangement breakpoints were merged in order to 1) associate copy number segments with rearrangements and 2) increase the accuracy of both the copy number and the rearrangement calls. Point 2 is particularly important with respect to small copy number segments such as small deletions, because the copy number changes of these small regions are often missed during copy number segmentation due to limited resolution but can be rescued by adding rearrangement calls as extra copy number breakpoints. Rearrangement and copy number breakpoint merging were perfomed as described below.

1. A combined breakpoint set was generated by combining all the copy number segmentation and rearrangement breakpoints.
2. Copy number segmentation breakpoints that were within 20kb of any rearrangement breakpoints were removed. The rationale to this step is that breakpoints from copy number segmentation and rearrangement calling that are in the vicinity of each other likely represent the same somatic rearrangement event and this step thus removes this redundancy from the breakpoint set. Breakpoints in rearrangement data are prioritised over copy number breakpoints, because their localisation on the genome should be more accurate (Supplementary Figure 13). The threshold of 20kb was chosen so that copy numbers are accurate as they are estimated using segments of at least 20kb in size on both side of the rearrangement breakpoints.
3. Of the remaining copy number segmentation breakpoints (that are not within 20kb of rearrangement breakpoints), those that demarcate segments of less than 10 genomic windows (typically equivalent to 5kb) were removed.
4. Of the remaining copy number segmentation breakpoints, those that demarcate an absolute copy number change of less than 0.3 copies were removed.
5. Rearrangement and copy number segmentation breakpoints located within sub-telomeric or centromeric regions (Supplementary Table 2) were removed. Copy number segments that partially overlapped with the defined sub-telomeric or centromeric boundaries are truncated to these boundaries.
6. After redefinition of breakpoints through steps 1-5, the copy number of each segment defined by the new breakpoint set is re-estimated by taking the median absolute copy number of the overlapping windows of each segment.
7. Estimating absolute copy number for *small segments* (here defined as segments with fewer than 10 overlapping windows, which typically corresponds to 5kb) using copy

number windows is very noisy because only few windows are located within them. Therefore, instead of using per-window read depth data, copy number for small segments is estimated using base-resolution read depth data as described below, summarised in Supplementary Figure 17.

i. A small segment is occasionally directly adjacent to other 'small' segments. In this case, all small segments were first grouped together and recorded.
ii. Following the definition of (i), the two segments immediately upstream and downstream of this sequence of small segments are not small segments. These are taken as anchor segments. The idea is that anchor segments are large enough for their copy number to be accurately estimated from per-window copy number data. If a small segment sequence is immediately next to a telomere or a centromere, then it only has one anchor segment (the other side being a telomere or centromere gap).
iii. The read depths of the anchor segments and the intervening small segments are computed using BedTools. If an anchor segment is larger than 10kb, then only the 10kb of the anchor region closest to the intervening small segments is included.
iv. Based on the read depth of each small segment and the read depths and estimated copy numbers of the anchor segments, the absolute copy number of the small segments is then estimated as follows.

$$o_1 = f \times cn_1 + (1-f) \times 2$$
$$o_2 = f \times cn_2 + (1-f) \times 2$$

$$\text{CN estimate} = \sqrt{\frac{c_{seg} \times \frac{o_1}{c_1} - (1-f) \times 2}{f} \times \frac{c_{seg} \times \frac{o_2}{c_2} - (1-f) \times 2}{f}},$$

where o_1 and o_2 are observed copy numbers of the anchor segments, f is aberrant cell fraction (i.e. $1 - \text{purity}$), cn_1 and cn_2 are the previously estimated absolute copy numbers of the anchor segments and c_1, c_2 and c_{seg} are the read depths of the anchor segments and the small segment, respectively. If only one anchor segment is available, then the copy number estimate is simply obtained using one anchor segment rather than taking the geometric mean of the estimates of the anchor segments.

Supplementary Table 2: Definition of chromosomal arm coordinates. Rearrangement and copy number breakpoints outside these boundaries are removed.

Chr	P-arm to centromere		Centromere to q-arm	
1	750000	121270000	150000000	249220000
2	10000	89330000	95390000	242950000
3	60000	90500000	93510000	197820000
4	40000	49090000	52680000	190910000
5	10000	46400000	49440000	180720000
6	200000	58770000	61880000	170920000
7	80000	58050000	61980000	159130000
8	160000	43790000	46880000	146300000
9	200000	38770000	70990000	141090000
10	100000	39150000	42400000	135230000
11	190000	51580000	54800000	134940000
12	180000	34850000	37860000	133840000
13	-	-	19360000	115110000
14	-	-	20190000	107290000
15	-	-	20030000	102280000
16	80000	35240000	46490000	90160000
17	0	22240000	25270000	81110000
18	130000	15410000	18540000	78010000
19	250000	24600000	27740000	59100000
20	120000	26290000	29420000	62920000
21	-	-	14340000	48100000
22	-	-	16850000	51200000
X	310000	58500000	61730000	155240000

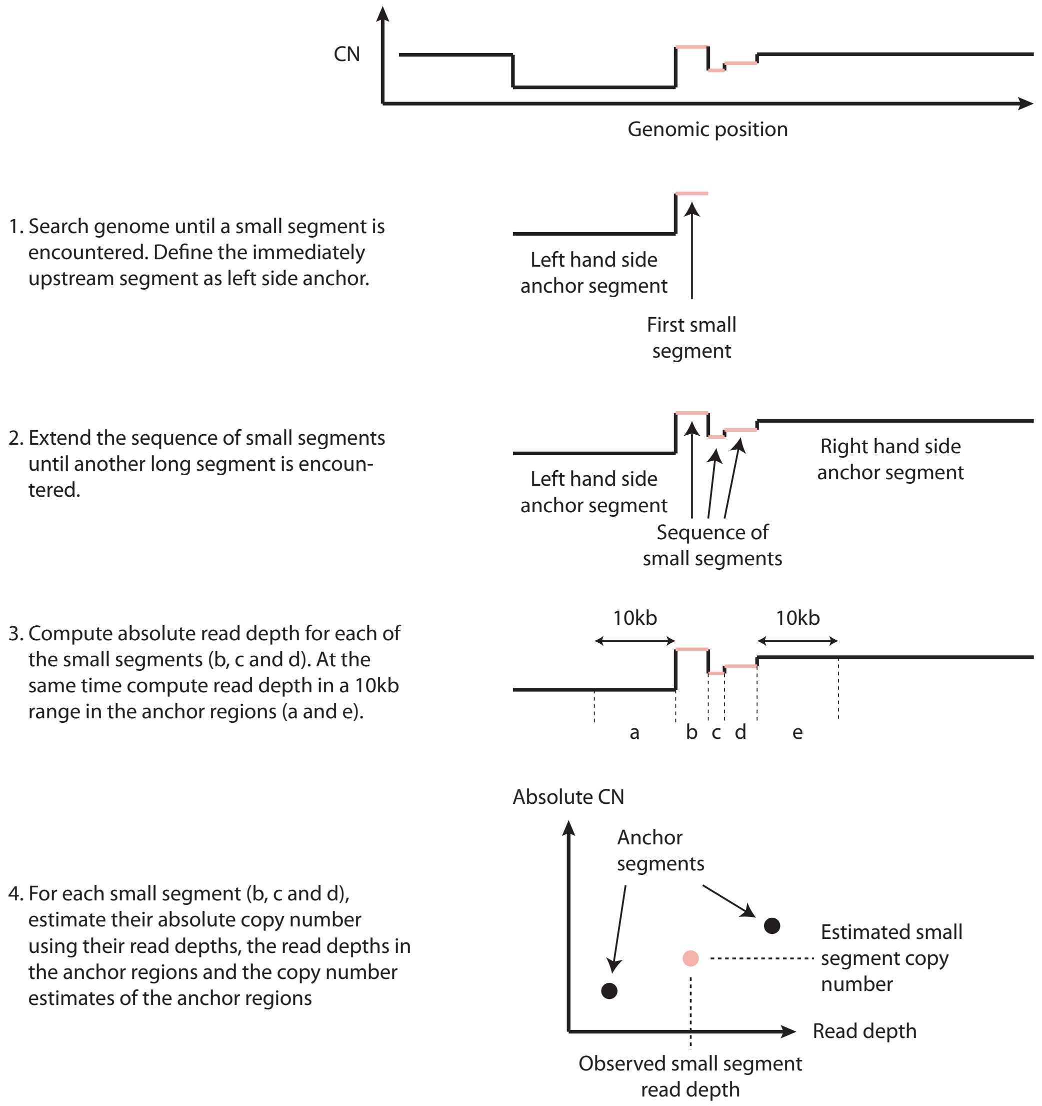

Supplementary Figure 17: Schematic representation of the algorithm for estimating the absolute copy number of small segments that only include few copy number windows.

4. Clustering SVs into SV clusters and their constituent footprints

SV clustering consists of three steps. First SVs are clustered together in an agglomerative manner as long as any pair of SVs are closer than expected based on background SV rate. Background SV rate estimation takes into account the background rate of different SV types (del, TD, inversion and inter-chromosomal) as well as their empirical size distributions. SV clusters are then divided into footprints, than can be considered independent contiguous regions that rearranged as part of the SV cluster event. Finally, we employ several heuristic steps to refine SV and footprint clustering.

Rearrangement clustering

The main challenge with rearrangement clustering lies with hypermutator samples with a high frequency of a particular SV type, such as tandem duplication. In these samples many SVs may cluster together simply by chance. More critically, the mere high frequency of certain basic SV types would cause them to frequently cluster together with real SV clusters such as

direct inversions. This contamination would detrimentally change the rearrangement patterns obtained from the real SV clusters. We therefore sought to develop an algorithm that considers the background rate and size distribution of each SV type (del, TD, inversion and inter-chromosomal) in a sample-specific manner.

An overview of the SV clustering algorithm is as follows.

1. We defined a distance metric *D* for pairs of SVs.
2. We implemented a method for computing the expected false positive (FP) number of SV pairs with a distance shorter than a given observed value under the assumption that the all SVs occurred independently without clustering. We accounted for sample-specific background rates and size distributions for each SV type when computing these expected numbers.
3. We computed the distance expected FP numbers for the distances computed between each pair of SVs in a sample.
4. We performed agglomerative hierarchical clustering on these expected FP numbers.

The distance metric. Let $d(a, b) = \text{abs}(a - b)/3\text{e}9$ be the distance between breakpoints *a* and *b* divided by genome length.

$$D(SV_i, SV_j) = \min(d(SV_{i,low}, SV_{j,low}) \times d(SV_{i,high}, SV_{j,high}), d(SV_{i,low}, SV_{j,high}) \times d(SV_{i,high}, SV_{j,low})),$$

where $SV_{i,low}$ and $SV_{i,high}$ refer to the low and high end positions of SV_i.

The intuition behind this distance metric is that given SV_i and SV_j are unrelated, the P-value for a given breakpoint in SV_j, say $SV_{j,low}$ being closer than $d(SV_{i,low}, SV_{j,low})$ from $SV_{i,low}$ should follow the exponential distribution. That is, $\Pr(d(SV_{i,low}, SV_{j,low}) < x) = 1 - e^{-\lambda x}$. Since we are mostly interested in situations when breakpoints are very close to each other ($x \approx 0$), we can use the well-known approximation $\log(1 + x) \approx x$ to yield $1 - e^{-\lambda x} \approx x$.

The expected FP number of SVs with observed distance. This is computed a given pairs of SVs. Suppose for the sake of an example that SV_i is of deletion type and SV_j is of tandem duplication type, and they have a SV distance of $D_{i,j}$. First SV_i is treated as an "anchor SV", and the following pseudocode is used to compute the expected number of *deletion-type* SVs that would yield a distance with $SV_i \leq D_{i,j}$.

```
expected_FP_number = 0
for other_sv in all deletion-type SVs:
    expected_FP_number += Prob(D(SVi, other_sv) ≤ Di,j)
```

The crucial part, Prob(D(SV_i, other_sv) ≤ $D_{i,j}$), can be computed analytically while considering the SV sizes of SV_i and *other_sv* (see code for details). Thus, since the for loop traverses all SVs of the relevant type (here: deletion), this method of computing the expected FP number of low distance SVs takes into account both the rate and the empirical size distribution of the SVs of relevant type: the number of SVs of the relevant type in the sample affects how many *other_svs* are considered, and sizes each *other_sv* affects Prob(D(SV_i, *other_sv*) ≤ $D_{i,j}$).

After the $\text{expected\_FP\_number}_{i,j}$ is computed with SV_i considered the anchor SV, the same computation is performed with the second SV, SV_j, considered the anchor SV. The final expected FP number for an SV pair (requiring the exact SV types as SV_i and SV_j) is computed by averaging the obtained expected FP numbers when considering each SV as the anchor SV.

This expected_FP_number is now an estimate for the expected number of SVs of the exact types as SV_i and SV_j that would yield a distance ≤ $D_{i,j}$ under the null hypothesis of the SVs being independent and while accounting for the respective SV counts and size distributions.

Agglomerative clustering. The *expected_FP_number* values computed between each pair of SVs reflect the expected number of pairs with a distance lower than the one observed given the SV types of each pair of SVs. However, the pairwise *expected_FP_number* value does not indicate how many SVs regardless of SV type would be expected to yield the observed SV distance.

Since there are four SV basic types (del, TD, inversion and inter-chromosome), there are ten ways in which they can pair together (four ways of two SVs having the same SV type, six ways of having pairs of two different SV types). Since we are performing agglomerative clustering, by definition SVs are clustered together based on their pairwise *expected_FP_number* values in ascending order. Therefore, when time comes to decide whether SV_i and SV_j should be merged, the expected number of SV distances ≤ $D_{i,j}$ has to be smaller than any remaining un-agglomerated expected number regardless of SV type. Thus, we can conservatively estimate that

$$\mathrm{E}\left[D_{\cdot,\cdot} < D_{i,j} \text{ regardless of SV type}\right] \leq 10 \times \mathrm{E}[D_{\cdot,\cdot} < D_{i,j} \mid \text{SV types of } D_i \text{ and } D_j]$$

Therefore, we converted by original *expected_FP_number* that only apply for specific SV types to the *expected_FP_number* regardless of SV type by simply multiplying the former by 10. These expected FP numbers were then transformed into FDR values by dividing them with the number of SV pairs accepted clustered.

We used agglomerative clustering with minimum as the distance involving already clustered clusters of SVs:

$$D(cluster\ 1, cluster\ 2) = min(D(SV_i, SV_j);\ SV_i\ is\ in\ cluster\ 1\ and\ SV_j\ is\ in\ cluster\ 2).$$

While visually examining initial clustering results, it was clear that there were some samples with very low rates of simple rearrangements but with massive chromothripsis events involving hundreds or even thousands of SVs. The high overall rate of SVs of each basic SV type in these samples led to an overestimation of the background rates of each SV type. To mitigate this, we applied a two-phase clustering approach. In the first phase, we aimed to capture and mark the high confidence clustered SVs by using an FDR cutoff of 0.01 for clustering. After this initial clustering step, all clustered SVs were removed from the background estimation, and the *expected_FP_number* values were recomputed for all the SVs, which were so far still not clustered. SV clustering was then continued for the still unclustered SVs using the new *expected_FP_number* values. In the final round of clustering, the FDR cutoff of 0.05 was used.

Determination of rearrangement cluster footprints

The SV clustering algorithm described above clusters individual rearrangements into clusters with the assumption that the unexpected level of clustering arises from the SVs having been generated through a rearrangement event involving multiple SV junctions.

Every SV cluster involves one or more chromosomes. Within each chromosome, SVs can be clustered (1) throughout the chromosome, (2) throughout a chromosomal arm or (3) interstitially. Moreover, a SV cluster can form multiple disparate clusters of breakpoints, or

footprints, on a chromosome. In other words, a footprint is a genomic interval that is assumed to have undergone complex rearrangement event involving potentially more than one footprint. The steps below describe how the boundaries and widths of the footprints are determined. Note that while the SV clustering algorithm groups SVs together, the footprint determination algorithm breaks an SV cluster down on the basis of its individual SV breakpoints. Two rearrangement ends of an SV can belong to the same or to different footprints.

SV clusters with only a single SV are defined to be formed by two footprints each containing one of the breakpoints of the SV. That is, singleton all SVs are defined to have two footprints each of which is exactly one base pair wide.

For non-singleton SV clusters, the idea behind footprint determination is to model inter-breakpoint distances using exponential distribution. The following steps are applied to a given SV cluster to determine its footprint coordinates. First, all inter-breakpoint distances of an SV cluster across all involved chromosomes is computed. Each interval between successive SV breakpoints is a candidate divider between individual footprints. Initially every chromosome involved in an SV cluster is assumed to contain only one footprint. The following steps are then iterated to divide individual SV breakpoints into footprints.

1. Use all inter-breakpoint distances d_i over $i = 1, 2, \ldots\ n$ intervals between breakpoints not (yet) classified as footprint dividers. Initially this includes all inter-breakpoint distances across all chromosomes of a SV cluster. Calculate the maximum likelihood mean inter-breakpoint distance under exponential distribution: $\lambda_0 = n/(\Sigma_i d_i)$ and $L_0 = \prod_i \lambda_0 e^{-\lambda_0/d_i}$.
2. Take the largest inter-breakpoint distance d_M and compute the alternate model maximum likelihood: $\lambda_1 = n/(\Sigma_{i\neq M} d_i)$ and $L_1 = \frac{1}{d_M} e^{-d_M/d_M} \prod_{i\neq M} \lambda_1 e^{-\lambda_1/d_i}$.
3. Compute the log-likelihood ratio $\Lambda = 2 \times (\log(L_1) - \log(L_0))$.
4. Compute the likelihood ratio test P-value using $\mathrm{P}(X \geq \Lambda)$, where X follows the Chi-squared distribution with one degrees of freedom.
5. Perform multiple testing adjustment on the P-value using the method by Benjamini and Hochberg. The number of tests is the number of inter-breakpoint intervals currently not yet classified as a footprint divider.
6. If the adjusted P-value < 0.01, then declare the location with inter-breakpoint distance of d_M as a footprint divider that separates two footprints on either side of it. After that iterate from step 1. If P-value ≥ 0.01, then stop iterating.

The code for grouping SVs into SV clusters and footprints is available at https://github.com/cancerit/ClusterSV/.

5. Heuristic refinement of SV clusters and footprints

The clustering and footprint determination algorithm used above was not perfect, and we applied several straightforward heuristic steps to improve the results.

- For each footprint, if there is a peripheral deletion or TD that does not overlap with any other breakpoints in the footprint, the deletion or TD was separated out from the footprint and put into its own cluster. This eliminates cases when a simple deletion or a simple TD gets clustered together with a real SV cluster by chance.

- Complete cycles of templated insertions or balanced breakpoints (see main text) were split out and put into their own clusters. This rescues some templated insertion and balanced breakpoint cycles that got accidentally clustered together with nearby unrelated SV clusters because they were located near each other by chance.
- Sometimes a local two-jump event (see main text) was split into two footprints, because there is a particularly sort inter-breakpoint distance. For example, in some Loss-InvDup events the breakpoints at the insertion point can be within <100bp from each other, causing the other inter-breakpoints intervals to be considered footprint dividers in the footprint determination algorithm. We merged all SV clusters involving two inversion-type SVs over a total genomic interval of ≤5Mb into a single footprint. This step rescued some local two-jump events that were otherwise considered distal templated insertions based on footprint analysis.
- Analogous to above, merge breakpoints within 5Mb forming the pattern A+^C+/C- (see below for the rearrangement pattern coding scheme) that got split into multiple footprints. This rescues some of these footprints.
- For the same reason as above, sometimes the two breakpoints forming a templated insertion or a balanced breakpoint may be split into two footprints each consisting of a single breakpoint. We merged all successive single-breakpoint footprints, if all of the following were true.
    - Each footprint consisted of a single breakpoint.
    - The footprints were within 5Mb from each other.
    - The two breakpoints had rearrangement orientations consistent with either a templated insertion (-+) or a balanced breakpoint (+-).
    - The next closest footprint for the two footprints considered were further than 15Mb away.
- Finally, if an SV cluster only has a single footprint and only includes deletion or tandem duplication-type SVs, then these deletions and tandem duplications are considered simple deletions and tandem duplications and separated into their own clusters. This increases the number of correctly clustered deletions and tandem duplications, as they can sometimes inadvertently form SV clusters at deletion (fragile sites) and tandem duplications (certain genomic loci) hotspots despite the involved deletions and tandem duplications being independent.

6. Filtering artefactual fold-back-type SVs with insufficient support

While we performed the initial rearrangement analysis, we noticed some samples with a high number of fold-back-type rearrangements. Two patterns suggested that most of these SVs were false positive rearrangements that got through the initial SV set generated through merging the Broad, DKFZ and Sanger SV calls. Firstly, the fold-back SVs in these samples were rarely supported by consistent copy number change (Supplementary Figure 18). Secondly, in samples with an exceptionally large number of fold-back-type SVs, majority of them were called by Snowman and Delly but not by the dRanger nor the Sanger pipelines.

Based on these observations, we added a post-hoc step to remove likely artefactual fold-back-type SVs. All solo fold-back SVs, i.e. those that did not cluster with other SVs, were removed if they were supported by only two of the four SV pipelines.

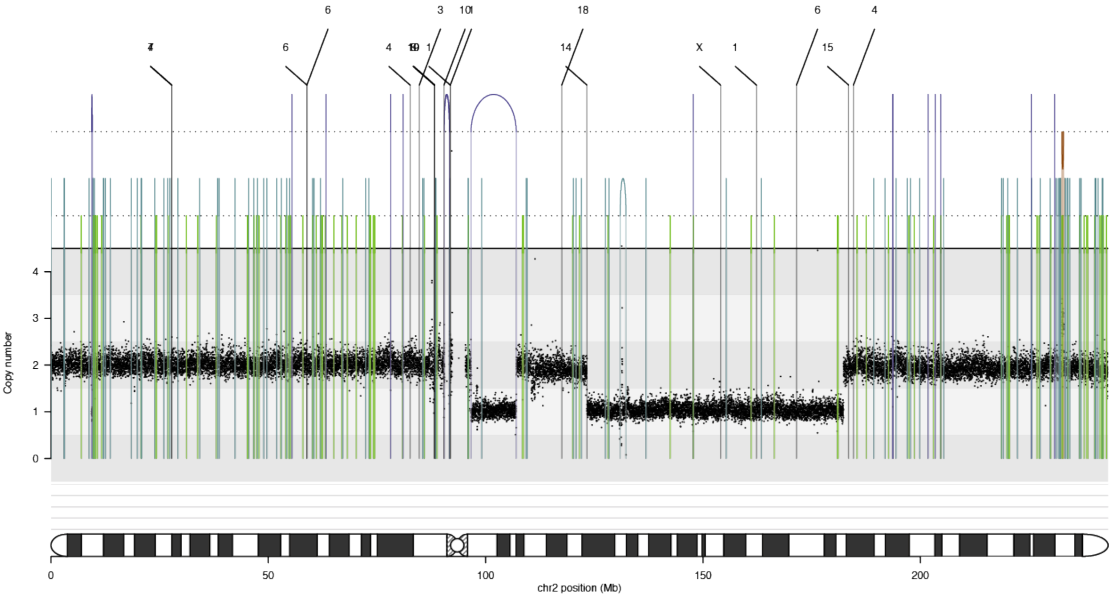

Supplementary Figure 18: An illustrative chromosome from a sample with a high number of fold-back-type SVs (green and cyan). None of the supposed fold-back rearrangements are associated with consistent copy number change. 7. Detecting mutually overlapping balanced breakpoints

As explained in Supplementary Figure 14, both balanced breakpoints and small templated insertions can have supporting reads from reciprocal rearrangements extending over each other, forming a small region of copy number gain. Therefore, without this step, some balanced breakpoints would be erroneously classified as templated insertions.

We used the exact breakpoint position values to check whether the reads supporting the first rearrangement end are soft-clipped from the rearrangement side at the same position as the reads supporting its reciprocal rearrangement end (Supplementary Figures 13-14). If such soft-clipping was found, the segment between the rearrangement ends was classified as a templated insertion. However, if the reads of the reciprocal rearrangement ends extended without soft-clipping further than where their mates' supporting reads were soft-clipped, then the segment was classified as a balanced breakpoint with overhang due to microhomology instead. The details of the method are as follows.

1. We only considered footprints comprising a single CN segment ≤100bp (and thus one at each end of the segment), as 100bp was the read length used in this study, and thus if the region of microhomology was larger than 100bp, then the reads would be multimapped anyways.
2. The following conditions must be met for the footprint to be considered a balanced breakpoint with microhomology. Otherwise the footprint classification was defaulted to templated insertion.
    - Rearrangement end orientations at the footprint are reverse (-) at the 5'-end and forward (+) at the 3'-end of the segment.
    - At the 5'-end of the footprint, the 5'-end of the reads supporting the forward rearrangement junction extend more than 5bp upstream of where the reads supporting the reverse rearrangement are soft-clipped.
    - At the 3'-end of the footprint, the 3'-end of the reads supporting the reverse rearrangement junction extend more than 5bp downstream of where the reads supporting the forward rearrangement are soft-clipped.

7. Determining balanced overlapping breakpoints

Sometimes breakpoints involved in a balanced rearrangement can include microhomology that causes their supporting reads to align beyond each other, creating an appearance consistent with a templated insertion (Supplementary Figure 14). We implemented a method using the object-oriented framework to determine based on the soft-clipping patterns of supporting reads whether a locus with a forward and a reverse breakpoint were consistent with a small templated insertion or with a balanced breakpoint (Supplementary Figure 14).

8. Computing rearrangement patterns

A string representation for entire rearranged somatic genomes

This method was developed in order to prune the rearrangement library search (described below) by avoiding searching for evolutions from the same somatic genome intermediates more than once.

In the context of structural variation analysis, the actual DNA sequence of a chromosome is mostly unknown. Instead, what is known is that the chromosome was derived from a wild type state through a sequence of rearrangement events, leaving observable rearrangement junctions. Therefore, having an encoding system for denoting SV junctions should be sufficient to describe the information available to rearranged derivative chromosomes.

For example, a chromosome with a tandem duplication and a deletion could be encoded by dividing the wild type chromosome into five segments, $\overrightarrow{\mathrm{A}}\overrightarrow{\mathrm{B}}\overrightarrow{\mathrm{C}}\overrightarrow{\mathrm{D}}\overrightarrow{\mathrm{E}}$, and then reporting the segment sequence of the derivative chromosome, e.g. $\overrightarrow{\mathrm{A}}\overrightarrow{\mathrm{B}}\overrightarrow{\mathrm{B}}\overrightarrow{\mathrm{C}}\overrightarrow{\mathrm{E}}$. Alternatively, one could represent the same derivative chromosome using an *equivalent* representation of $\overleftarrow{\mathrm{E}}\overleftarrow{\mathrm{C}}\overleftarrow{\mathrm{B}}\overleftarrow{\mathrm{B}}\overleftarrow{\mathrm{A}}$. In a given encoding scheme, a *single* derivative chromosome always has two equivalent representations. In order to systematically perform statistical analysis on rearrangement patterns, we need a way to equate them when they represent the same pattern with a different but equivalent representation, e.g. when they are $\overrightarrow{\mathrm{A}}\overrightarrow{\mathrm{B}}\overrightarrow{\mathrm{B}}\overrightarrow{\mathrm{C}}\overrightarrow{\mathrm{E}}$ in one place and $\overleftarrow{\mathrm{E}}\overleftarrow{\mathrm{C}}\overleftarrow{\mathrm{B}}\overleftarrow{\mathrm{B}}\overleftarrow{\mathrm{A}}$ in another.

A genome can be represented as an ordered list of its chromosomes, each with one of its two equivalent orientations. One can arrange the chromosomes and chromosomal orientations in any given way and still produce the same equivalent genome. This yields for a given genome a total of $(n!) \times 2^n$ representations, where n is the number of derivative chromosomes (Supplementary Figure 19). We say that different representations of the same derivative genome belong to the same representational equivalence class.

One way to tell if two different representations describe the same (or equivalent) genome is to take one of the two representations, enumerate all possible permutations (in terms of chromosomal ordering and orientation) of that representation and check whether any of them match exactly with the second representation. This brute force approach requires $(n!) \times 2^n$ iterations which can quickly become prohibitive when the number of genomes grow.

We therefore implemented a faster approach based on a function $m(\cdot)$ such that for genomic representations r_1 and r_2, $m(r_1) = m(r_2)$ if and only if r_1 and r_2 are equivalent representations of the same genome under our somatic genome encoding scheme.

We start with a string representation for genomic configuration. Given an array of derivative chromosomes with associated orientations representing a genome, the segments in each chromosome can be written out and concatenated to produce a 'genome string representation' (Supplementary Figure 19). Strings can naturally be lexically sorted, so $m(r)$ could be implemented by mapping r to the *lexicographically smallest genome string* among configurations of the genome represented by r. As chromosomal segments do not have an inherent identity, we simply label them numerically in the order in which they are encountered in a genomic representation (Supplementary Figure 19).

From the design of the genomic representation, one can see that the string representation is constructed left to right chromosome by chromosome. Therefore, instead of having to stringify all genomic configurations of a given genome, one can dynamically exclude strings that are guaranteed not to be lexicographically smallest based on how they start. This is achieved as follows. In the first iteration, every chromosome of the given derivative genome in both possible orientations is put as the first chromosome of the string representation. The stubs of the genome strings with one chromosome only can then be compared and those that are not lexicographically smallest at this point can then be ignored. The remaining genome configurations can then be iteratively extended and each time the genome string is extended by adding a new chromosome, those that result in non-smallest genome strings so far are removed. This procedure is repeated for each subsequent chromosome until all chromosomes have been included in the final representation. At this point the final representation is guaranteed to be lexicographically smallest.

Note that this method generalises naturally to breakpoint footprints of SV clusters. The only change needed is to ignore the host chromosomes of the footprints and instead consider each footprint as an independent 'chromosome'. For example, a templated insertion from chromosome 1 to chromosome 2 or from chromosome 2 to chromosome 1 share the same rearrangement pattern and mechanism. Such rearrangement pattern equivalences between SV clusters can be systematically computed using this rearrangement pattern encoding scheme.

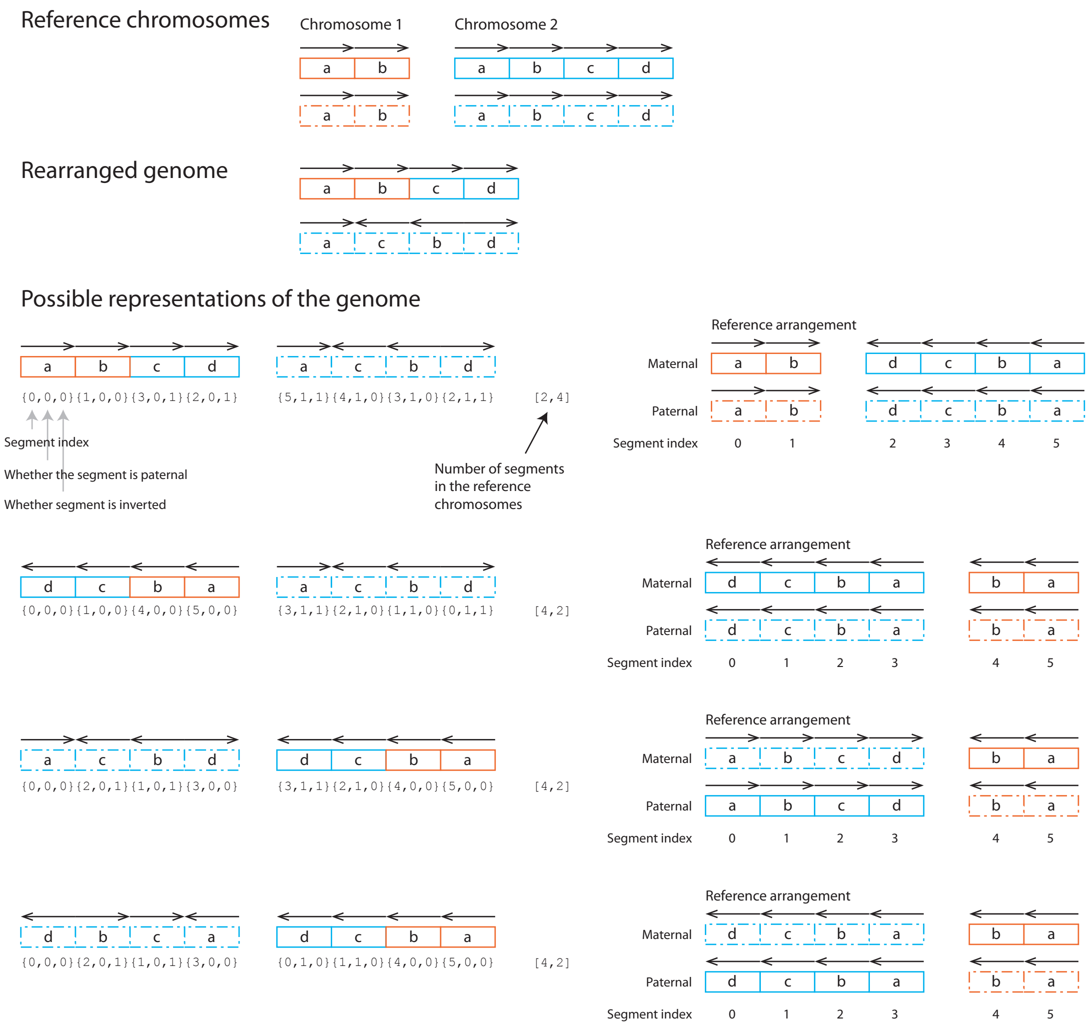
Reference chromosomes
Chromosome 1
Chromosome 2
Rearranged genome
Possible representations of the genome
{0,0,0}{1,0,0}{3,0,1}{2,0,1}
{5,1,1}{4,1,0}{3,1,0}{2,1,1}
[2,4]
Segment index
Whether the segment is paternal
Whether segment is inverted
Number of segments in the reference chromosomes
{0,0,0}{1,0,0}{4,0,0}{5,0,0}
{3,1,1}{2,1,0}{1,1,0}{0,1,1}
[4,2]
{0,0,0}{2,0,1}{1,0,1}{3,0,0}
{3,1,1}{2,1,0}{4,0,0}{5,0,0}
[4,2]
{0,0,0}{2,0,1}{1,0,1}{3,0,0}
{0,1,0}{1,1,0}{4,0,0}{5,0,0}
[4,2]
Reference arrangement
Maternal
Paternal
Segment index

Supplementary Figure 19: A string representation for a rearranged genome and finding the lexicographically smallest string representation for a rearranged genome. A schematic representation of a diploid genome consisting of two chromosomes is shown on top. Parental origin of the segments is denoted by their dashed or solid outline. The rearranged chromosome is generated through an unbalanced translocation between chromosomes 1 and 2, a whole-chromosome loss of a copy of chromosome 1 and an inversion in chromosome 2, resulting in two derivative chromosomes. The two derivative chromosomes can be put in two different orders and within each ordering each derivative chromosome can be represented in two different orientations. This yields a total of eight different possible combinations of arranging the derivative chromosomes in an array, four of which are shown in the diagram in the bottom section. For each derivative chromosome ordering and orientation combination, a string can be used to describe the segments of each derivative chromosome. Every segment is indexed, and reference chromosomes are rearranged and oriented in such a way that the resulting genome string is lexicographically smallest for the derivative chromosome arrangement in question. This way, a lexicographically smallest representation is obtained for each derivative chromosome arrangement. In order to find the overall lexicographically smallest representation for the derivative genome, one only has to find the derivative chromosome arrangement that produces the lexicographically smallest string representation. The naïve way to find the derivative chromosome arrangement that produces the lexicographically smallest genome representation is to enumerate all possible arrangements and compute the string representation for each of them. A more efficient way is to build the genome string representations using essentially a prefix tree in a breadth-first approach (i.e. chromosome by chromosome) and iteratively remove representations (branches) that are guaranteed to not be lexicographically smallest. The example arrangements shown in the figure illustrate all four possible derivative chromosome choices and orientations arranged as the first chromosome. From these arrangements one can see that by having the derivative chromosome generated through a translocation first and orienting it with the orange segments first produces the lexicographically smallest string for the first derivative chromosome, and therefore by starting a derivative chromosome arrangement with any other chromosome or orientation is guaranteed to produce a lexicographically larger genome string. This stepwise string extension can then be continued until all derivative chromosomes are placed in the arrangement, at which point the resulting genome string is guaranteed to be lexicographically smallest for the entire genome.

A string representation for rearrangement patterns

The actual segment structure of individual chromosomes is not observed through NGS-based rearrangement analysis. Instead, only copy numbers and rearrangements associated with the segments are observed. This process causes loss of information, and sometimes two non-equivalent genomes can produce the exactly same breakpoint and copy number patterns (Supplementary Figure 20). The key difference between somatic genome representations and rearrangement patterns is that the former contains complete information of the somatic karyotype, but the latter only provides the somatic rearrangement junctions and copy number.

Similar to the section above, we used the lexicographically smallest rearrangement pattern string representation to describe a rearrangement pattern. Furthermore, generating normalised rearrangement patterns for both real SV clusters and SV patterns generated through the rearrangement library (described below) allowed us match rearrangement patterns in real data to their equivalent simulated patterns (Supplementary Figure 20).

Copy number estimates in real data are often too noisy to obtain accurate integer copy numbers. Since rearrangement pattern strings start with rearrangement junctions followed by CNs, if is possible to use normalised rearrangement pattern strings without the CN part.

Note that the numbering and of segments is only used internally in the algorithm. In this manuscript, segment numbers are replaced by alphabets (A, B, C…) and rearrangement junctions are indicated by a caret as opposed to a comma used in Supplementary Figure 20.

Thus, the internal representation for Loss-InvDup is “0+,3+/2-,3- “, but the one used in this manuscript is “A+^D+/C-^D-“.

It is possible to describe individual footprints using the rearrangement pattern string. A breakpoint and its partner breakpoint do not always belong to the same footprint. In this case, the rearrangement junction section of an orphan breakpoint will simply not have the partner breakpoint part, i.e. the caret and the breakpoint after the caret. For example, the rearrangement string representation for a unbalanced translocation breakpoint footprint is simply “A+”.

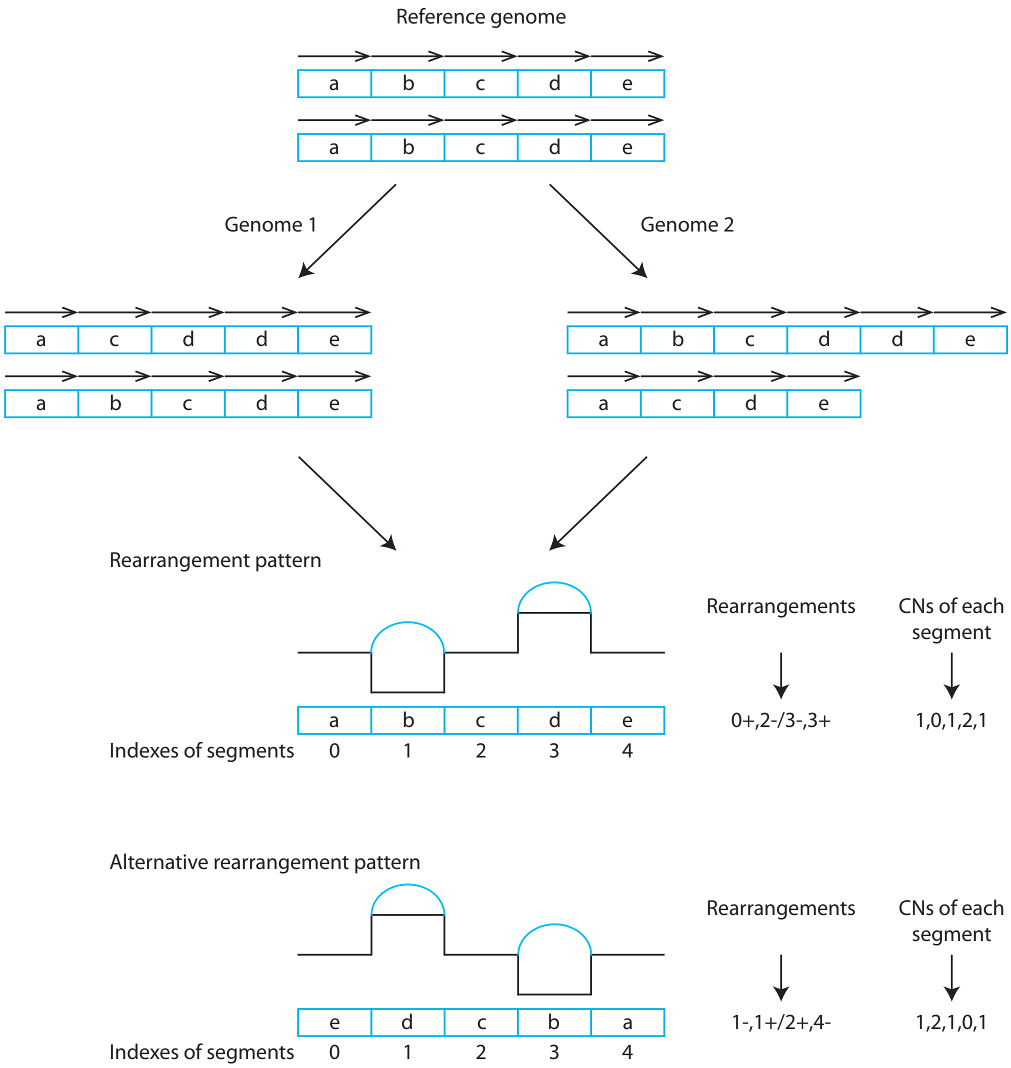

Supplementary Figure 20: Rearrangement pattern strings for describing rearrangement patterns. Top part of the figure shows two different rearranged genomes consisting of two copies of one chromosome. In genome 1 one copy of the chromosome is wild type and the other copy has a deletion and a tandem duplication. In genome 2 one copy has the tandem duplication and the other copy has the deletion. Both derivative genomes produce the exactly same rearrangement pattern. The same rearrangement pattern can also be inverted to produce a different, but equivalent description of the rearrangements and copy numbers. The rearrangement pattern strings used to describe the rearrangement patterns are shown on the right hand side of the rearrangement patterns. Note that first orientation of the rearrangement pattern produces a lexicographically smaller string representation compared to the second one, and is thus the normalised representation for this rearrangement pattern.

Library of all possible rearrangement patterns

We wrote a C program for simulating the structures of somatic genomes following exhaustively enumerated sequences of basic rearrangement types. The core of the framework is simple. First a wild type genome, defined as an array of one or more chromosomes each in either a single copy or in diploid pairs is created. Rearrangement events from an array of known rearrangement mechanisms are applied one at a time on the wild type genome in all possible positions and orientations. The event types included in the framework are the following.

- Internal deletion
- Tandem duplication
- Direct inversion
- Unbalanced translocation
- Balanced translocation
- Terminal deletion
- Terminal deletion + telomeric fusion (breakage-fusion-bridge)
- Whole-chromosome gain
- Whole-chromosome loss
- Whole-genome duplication

The same process can now be repeated recursively, applying a second rearrangement event on the derivative genomes that underwent one event so far, and so on, until the desired depth, i.e. the total number of rearrangements to be simulated, is reached. The simulation process can be modelled as a tree, where the root is the wild type genome and branches are child genomes derived from an application of an additional rearrangement event (Supplementary Figure 21).

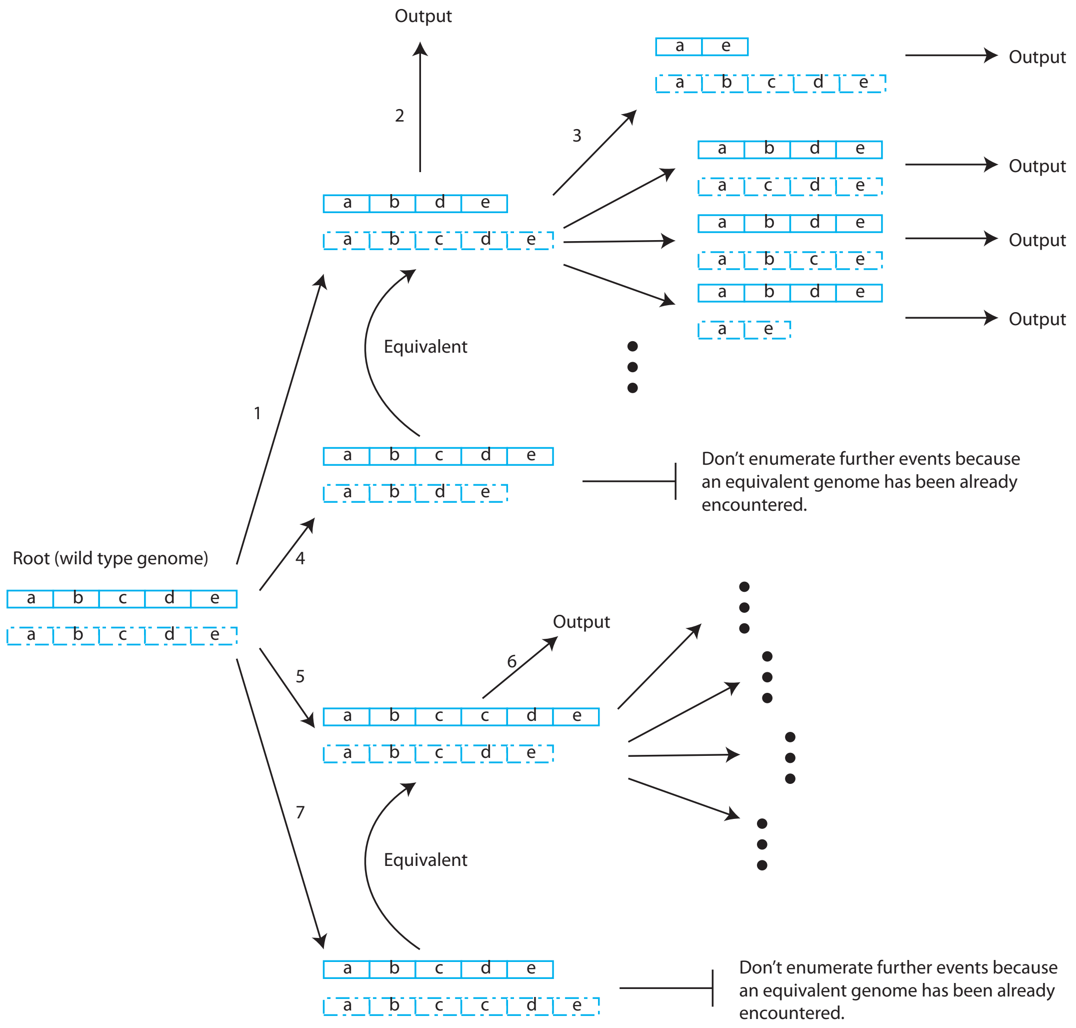
Output
a e
a b c d e
Output
3
2
a b d e
a c d e
Output
a b d e
a b c d e
a b d e
Output
a b c e
a b d e
Output
a e
Equivalent
1
a b c d e
a b d e
Don't enumerate further events because an equivalent genome has been already encountered.
Root (wild type genome)
4
a b c d e
a b c d e
Output
6
5
a b c c d e
a b c d e
7
Equivalent
a b c d e
a b c c d e
Don't enumerate further events because an equivalent genome has been already encountered.

Supplementary Figure 21: Schematic representation of the rearrangement simulation algorithm. In this example the wild type genome contains two parental copies of one chromosome, and the only simulated rearrangement event types are internal deletion and tandem duplication. The depth of search in this example is two events. The search algorithm is depth first. First, one rearrangement event, in this case a deletion, is simulated onto the wild type genome (1). This generates a derivative genome, whose genome string, rearrangement pattern and evolution is printed as output (2). In addition, the genome string representation of the derivative genome is stored in a hash table. Since the desired depth has not been reached yet, the rearranged genome with a deletion is subjected to further rearrangements. An additional deletion can be simulated onto the rearranged genome in multiple places now (3). The secondary deletion can overlap the original deletion. Alternatively, the secondary deletion could happen on the wild type copy of the chromosome, in which case the deletion could land before, over or after the initial deletion. In addition, simulations with a secondary tandem duplication in all possible locations are also carried out. In each of these cases, the secondary event reaches the desired depth of so events, so each of the rearranged genomes with two events are output and their respective genome string representations are stored in the genome string hash table, but no further rearrangements are applied to them. So far the algorithm has exhaustively enumerated all evolutions starting with a deletion on one copy of the chromosome. Next, the algorithm retracts back to the wild type genome, and searches for the next possible place for a deletion, which in this case will be in the other parental copy of the chromosome (denoted with a dashed outline) (4). After applying a deletion on this chromosomal copy, the genome string representation for the rearranged genome is computed. By querying the hash table with this string, it will then be noted that a somatic genome with an equivalent structure has already been encountered during the search, namely that belonging to the product of (1). Therefore, all leaves derived from the current rearranged genome (4) will be identical to those generated from the first genome (1). Instead of enumerating all rearranged genomes from (4), the algorithm simply prints a statement that the evolution of (4) produces an identical rearranged genome to that of the evolution of (1). Now the algorithm backtracks to the wild type genome again, and since all placements of deletions have been visited, the algorithm will now place tandem duplications on the wild type genome. The first rearranged genome with a tandem duplication (5) has not been encountered yet as it is not found in the genome string representation hash table, so it will have its genome string, rearrangement pattern string and evolution printed as output (6) and its genome string stored in the genome string hash. Then further patterns are searched starting from this rearranged genome. Subsequently when a tandem duplication is applied to the chromosomal copy with dashed outline (7), the algorithm again finds out that a genome with a genome representation string has already been encountered, and thus the algorithm simply states this and does not proceed to enumerate evolutions and patterns from this rearranged genome.

The number of distinct genomic evolutions, and thus the search space, explodes after just a few simulated events. However, two distinct evolutions can produce derivative genomes that are equivalent, as defined above (Supplementary Figure 21). We thus prune the simulation tree whenever the genome string indicates duplicate representations. This is implemented by maintaining a hash table that of all encountered genomic configuration with the rearrangement evolution that produced the derivative genome.

The simulations are performed depth first, and every time a new simulated chromosome is generated, the subsequent action is selected from the following.

- Compute the normalised genome representation string for the current rearranged genome.
- Lookup the normalised genomic representation from the hash table.
- If an equivalent genome representation has already been generated through an evolution traversed earlier, then simply print a statement indicating that the current evolution converges to a structure generated from an earlier evolutionary sequence, which is retrieved from the hash table. After that skip the remaining steps.
- If an equivalent genome representation string has not yet been encountered, then output the evolutionary history of the current derivative genome together with the rearrangement pattern and the rearrangement evolution that produced the current

pattern. After that store the current genomic representation into the genome string hash.
- If the desired depth is not reached, then simulate all possible events starting from the current rearranged genome. Otherwise backtrack to the next genome that needs to be simulated.

The code for simulating rearrangements can be found at https://github.com/cancerit/SimSvGenomes.

Interpretation and analysis of rearrangement patterns

This section describes the methods used in statistical analysis of rearrangement patterns.

Inverted copy number gain patterns

Stepwise generation of inverted copy number gain events

We searched for sequences of up to four simple rearrangement events that generate the following rearrangement patterns:

- A+^D+/C-^D- (Loss-InvDup)
- B-^D-/B+^D+ (Dup-InvDup)
- B-^C-/C+^D+ (Dup-Trp-Dup)

In order to find inversions nested within tandem duplications, we searched for either

- Direct inversions that are nested within tandem duplications; or
- Rearrangement clusters containing exactly three rearrangements on the same chromosome, and have the rearrangement pattern compatible with a direct inversion nested within a tandem duplication, i.e. B-^F+/B+^D+/D-^F-.

In both cases, the tandem duplication part of the rearrangement was required to be at most 10Mb. This was to avoid situations when large tandem duplication-type intra-chromosomal rearrangements are misclassified as tandem duplications and thus are spuriously interpreted as direct inversion nesting tandem duplications.

Relative copy numbers of chromosomes with inverted copy number gain patterns

The following is done for each sample and each pattern, i.e. inverted copy number gain patterns, direct inversion, inter-chromosomal unbalanced translocation and fold-back inversion.

For each chromosome with one or more instances of the pattern under analysis, the copy number difference between the chromosome and the average chromosomal copy number of the entire genome was computed. After that, the average copy number difference over all chromosomes with the respective pattern is stored. If a sample had more than five chromosomes with the pattern, then the respective pattern for the sample was not counted. This is to avoid bias when most chromosomes have at least one instance of the pattern.

After the above data is stored, for each rearrangement pattern per sample, we plotted the average chromosomal copy number differences compared to the chromosomes without an instance of the pattern.

Relative copy numbers of chromosomes with footprints of two breakpoints involving two rearrangements

Relative copy numbers for different footprints involving two breakpoints from two rearrangements (A+^B+, A+^C- and B-^B+) were computed the same way as for the inverted copy number gain patterns described above.

Finding chains and cycles of templated insertion and balanced breakpoint footprints

This was implemented in the object-oriented somatic rearrangement handling framework. A randomly selected templated insertion footprint is used as the anchor. The lower end breakpoint of the footprint is then used to check whether the mate of the lower end is also on a templated insertion footprint. If yes, then the other breakpoint of the second templated insertion is selected and the “walking” of templated insertions is thus continued.

If the original templated insertion footprint is reached through this process of footprint walking, then the footprint (and all other traversed footprints) lie on a footprint cycle.

Tandem duplications are technically templated insertion footprint cycles of length 1, but they are annotated as tandem duplications.

If the original templated insertion footprint is not on a templated insertion cycle, then the length of the chain starting from the lower end breakpoint is noted, and the length of the chain starting from the higher end breakpoint can be computed in a similar way. Finally, the total length can be summed up.

Chains and cycles of balanced breakpoint footprints are computed in an analogous manner.

Signatures of somatic rearrangements

Event and footprint types included in the analysis

The size of tandem duplications is distributed over several clusters especially in cohorts with high rates of tandem duplications (Supplementary Figure 22). We therefore divided tandem duplications into four classes based on size, with size thresholds chosen somewhat arbitrarily at $5.5{\times}10^4$bp, $2{\times}10^6$bp and 10^7bp.

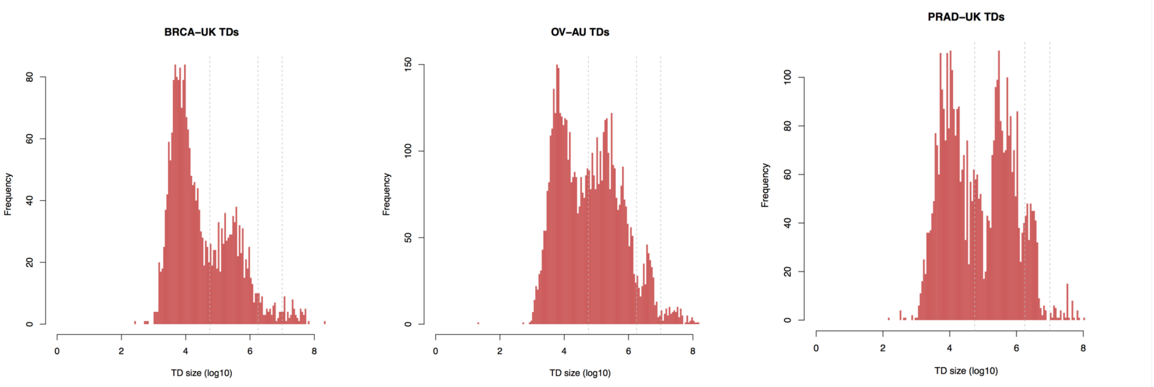

Supplementary Figure 22: Tandem duplication size histograms from three cohorts. Size distribution of tandem duplications from different tumour types (breast cancer, ovarian cancer and prostate cancer respectively). Vertical lines are at 4.75, 6.25 and 7.

Similarly, there was clear clustering of deletions by size in cohorts with high rates of deletions (Supplementary Figure 23). We therefore divided deletions into three different size classes separated by thresholds of 10^4bp and 3×10^6bp.

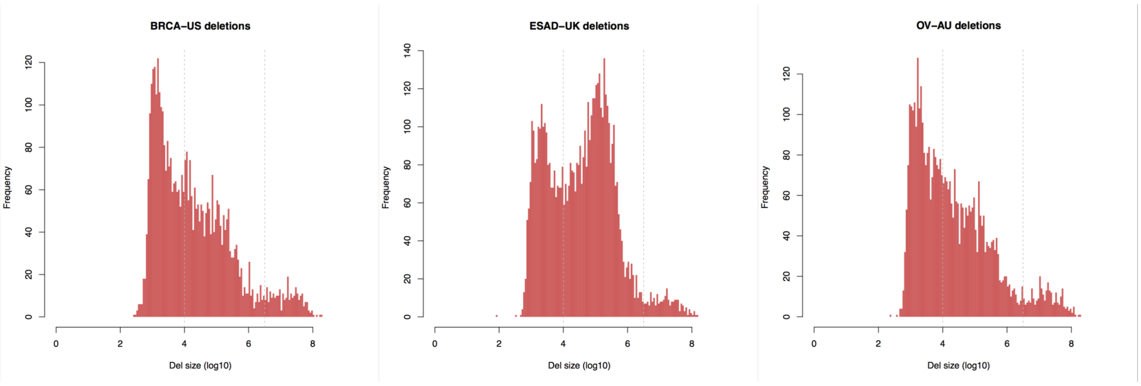

Supplementary Figure 23: Deletion size histograms from three cohorts. Size distribution of tandem duplications from different tumour types (breast cancer, oesophageal cancer and ovarian cancer respectively). Vertical lines are at 4 and 6.5.

For both deletions and tandem duplications, those located entirely within a fragile site were classified as fragile site deletions and tandem duplications and not split by size.

With the similar reasoning, templated insertions (Supplementary Figure 24) and balanced breakpoints (Supplementary Figure 25) were divided into three groups using cutoffs of 10^3 bp and 10^5 bp for templated insertions and 10^2 bp and 10^5 bp for balanced breakpoints. Templated insertions and balanced breakpoints were further categorized by whether they were present in a chain or in a cycle.

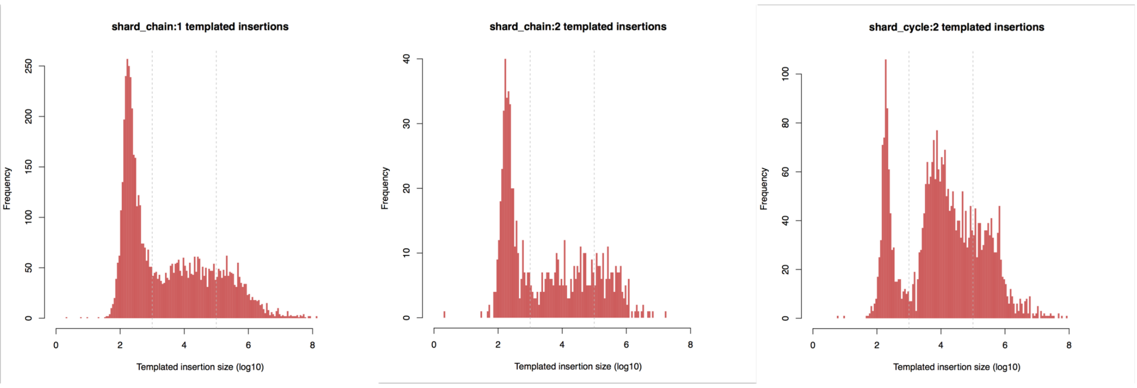

Supplementary Figure 24: Templated insertion size distributions. Size distribution of templated insertions of different classes (cycles of templated insertions containing one template; chains of templated insertions containing 2 templates; and cycles of templated insertions containing two templates respectively).

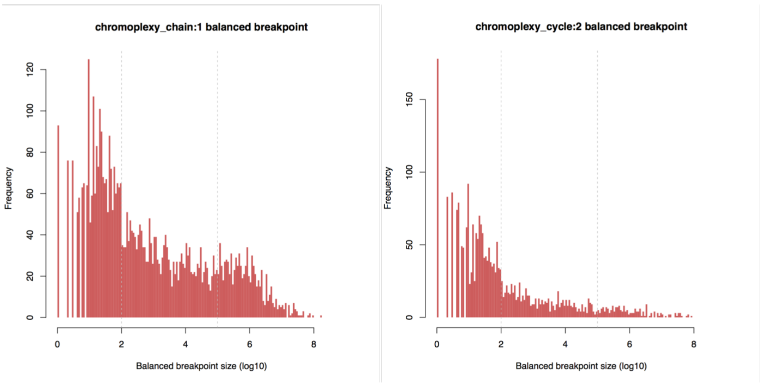

Supplementary Figure 25: Balanced breakpoint size distributions. Histogram of distances between the two ends in balanced chromoplexy chains (length 1 and 2 respectively).

Finally, direct inversion and inverted gain-loss footprints appeared to fall into two groups of sizes (Supplementary Figure 26). Based on this, these two footprint types were separated by size using threshold 10^5bp.

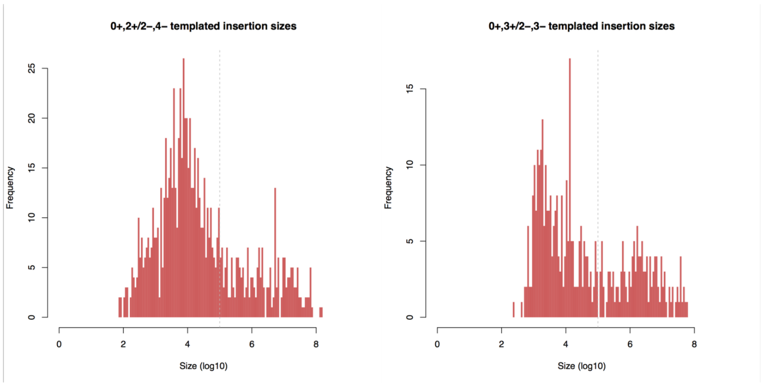

Supplementary Figure 26: Size distribution of direct inversions and Loss-InvDup SV clusters. Histogram of sizes of the inserted fragments in balanced inversions and Loss-InvDup 2-jumps respectively.

In addition to the aforementioned event or footprint types, we included all footprints with five or fewer breakpoints, if the footprints had a total incidence of at least 50 instances in the entire PCAWG cohort, with the following further adjustments.

- Footprints corresponding to simple unbalanced breakpoints were considered separately from footprints corresponding to unbalanced breakpoints of an SV that was part of a more complex SV cluster.
- Inversion-type SVs that were not clustered with any other SVs were divided into different categories by size. Inversion SVs with a distance ≤50kb were classified as fold-back inversions and other inversion SVs were classified as intra-chromosomal inversion SVs.
- Reciprocal balanced translocation (i.e. chromoplexy cycle of two footprints) breakpoints and unreciprocal balanced translocation (i.e. chromoplexy chain of one footprint) breakpoints were put into separate classes from other chromoplexy chains and cycles.

Nonnegative matrix factorisation of SV event counts

We ran nonnegative matrix factorisation (NMF) using the R package 'NMF'. We used Kullback-Leibler distance as the cost function, which converges towards the maximum Poisson

likelihood[6], which is a natural choice for nonnegative count data. Since we are modelling SV event counts with a Poisson generative process, we can naturally derive a Poisson likelihood for our fit, which allowed us to use Akaike information criterion for model selection.

Library of genome properties

We divided the hg19 human reference genome (autosomes and chromosome X) into 3,036,315 pixels of 1kb, and calculated a suite of metrics per-pixel to summarise a variety of genome properties with potential relevance to the distribution of rearrangements (Supplementary Table 3). Properties from ROADMAP were matched as closely as possible to the tissue of origin for PCAWG cancer samples (Supplementary Table 4). All other genome properties were held fixed across all tissues.

Supplementary Table 3: Library of genome properties. Each property is listed in the left column, how it is quantified in the third column, where the data was accessed from in the fourth column and the reference (where applicable) in the final column.

<table>
<tr><th>Property</th><th>Note</th><th>Metric for 1kb pixel</th><th>Source</th><th>Reference</th></tr>
<tr><td>Centromere</td><td rowspan="2"></td><td rowspan="2">$\log_{10}$ (Mb distance +1)</td><td rowspan="2">UCSC GB hg19 gap track</td><td rowspan="2">doi:10.1093/nar/gkt1168</td></tr>
<tr><td>Telomere</td></tr>
<tr><td>GC content</td><td></td><td>Proportion of GC bases in pixel</td><td>Hg19 FASTA</td><td></td></tr>
<tr><td>Sequence complexity</td><td>Custom metric inspired by DUST, high values indicate low sequence complexity.</td><td>Sum of squares of trinuc. motif counts in pixel, divided by square of non-N pixel width</td><td>Hg19 FASTA</td><td></td></tr>
<tr><td>H3K9me3</td><td rowspan="12">Raw data from ROADMAP are the p-value signal tracks from ChromImpute (doi:10.1038/nbt.3157)</td><td rowspan="12">Average imputed value in pixel, matched for cell type.</td><td rowspan="12">ROADMAP</td><td rowspan="12">doi:10.1038/nature14248</td></tr>
<tr><td>H3K36me3</td></tr>
<tr><td>H4K20me1</td></tr>
<tr><td>H3K79me2</td></tr>
<tr><td>H3K4me1</td></tr>
<tr><td>H3K27ac</td></tr>
<tr><td>DNase</td></tr>
<tr><td>H3K9ac</td></tr>
<tr><td>H3K4me3</td></tr>
<tr><td>H3K4me2</td></tr>
<tr><td>H2A.Z</td></tr>
<tr><td>H3K27me3</td></tr>
</table>

RNA expression	Imputed logRPKM track from RNA-seq data	Average imputed value in pixel, matched for cell type.	ROADMAP	doi:10.1038/nature14248
DNA methylation	Imputed fractional methylation track from DNAMethylSBS data	Average imputed value in pixel, matched for cell type.	ROADMAP	doi:10.1038/nature14248
Replication time	Cell lines: NHEK (normal skin, ectoderm), GM12878 (normal blood, mesoderm), IMR90 (normal lung, endoderm)	Wavelet-smoothed signal value averaged across three cell lines. High values are early replicating.	ENCODE / University of Washington Repli-Seq	doi:10.1038/nature11247 doi:10.1073/pnas.0912402107
Recombination rate	2011-01_phaseII_B37 version	Value at nearest point.	HAPMAP phase II	doi:10.1038/nature06258
Genes	Protein-coding genes	Density in 1Mb windows, sliding every 1kb to centre on the pixel	GENCODE v19	doi:10.1101/gr.135350.111
Lamina associated domains	Tig3ET normal human embryonic lung fibroblasts	Density in 1Mb windows, sliding every 1kb to centre on the pixel	Supp Data from Guelen et al, lifted over to hg19	doi:10.1038/nature06947
CpG islands		$\log_{10}$ (kb distance +1)	UCSC GB cpgislands track	doi:10.1093/nar/gkt1168
Direct repeats	repeats of 10—300bp repeated directly one or more times 0—10bp away	$\log_{10}$ (kb distance +1),	non-B DB v2	doi:10.1093/nar/gks955
G-quadruplex motif	Subset of non-B DB v2 definition – chose motifs with four runs of three Gs, with 1bp between each run	$\log_{10}$ (kb distance +1)		

Cruciform inverted repeats	repeats of 6 or more bp repeated inversely up to 4bp away	Density in 3kb windows, sliding every 1kb to centre on the pixel		
Triplex mirror repeats	repeats of 10 or more bp with 90% of one strand made of pyrimidines and repeated as a mirror up to 8bp away	$\log_{10}$ (kb distance +1)		
Short tandem repeats	repeats of 1—9bp repeated perfectly three or more times with no bases between	Density in 3kb windows, sliding every 1kb to centre on the pixel		
Z-DNA motifs	alternating purine-pyrimidine tracts of 10 or more bp, excluding AT/TA dinucleotide repeats	$\log_{10}$(kb distance +1)		
ALU repeats		$\log_{10}$ (kb distance +1)	Repeatmasker 20140131	http://www.repeatmasker.org
MIR repeats				
L1 repeats				
L2 repeats				
LTR repeats				
DNA repeats	DNA transposons			
Simple repeats	Microsatellites			
TAD boundaries	IMR90 cell line	$\log_{10}$ (kb distance +1)	Supp Data Dixon et al, lift over to hg19	doi:10.1038/nature11082
Nucleosome occupancy	Cell line K562, MNase experiment	Raw value per **base** rather than per 1kb pixel (only exception)	ENCODE	doi:10.1038/nature11247

Supplementary Table 4: ROADMAP cell lines averaged over to estimate a match to the cancer sample tissue of origin. The tissue of origin is shown in the left column, and the relevant cell lines in the right column.

Tissue	EIDs of Roadmap cell lines
Biliary	E028,E065,E076,E079,E094,E096,E098,E109,E126,E127
Bladder	E028,E065,E076,E079,E094,E096,E098,E109,E126,E127
BoneSoftTissue	E025,E107,E108,E129
Breast	E027,E028,E119
Cervix	E117
CNS	E067,E068,E069,E070,E071,E072,E073,E074
ColonRectum	E075,E076,E102,E103
Esophagus	E079
HeadNeck	E079
Kidney	E086
Liver	E066
Lung	E088,E096,E128
Lymphoid	E032,E034
Myeloid	E029,E030
Ovary	E097
Pancreas	E087,E098
Prostate	E028,E065,E076,E079,E094,E096,E098,E109,E126,E127
Skin	E059,E061,E126,E127
Stomach	E094,E110,E111
Thyroid	E080
Uterus	E028,E065,E076,E079,E094,E096,E098,E109,E126,E127

Callable genome space

To estimate the 'callable' subset of the hg19 reference genome (regions in which variants are able to be detected), we considered a random collection of 200 normal (not cancer) sample BAM files from the ICGC PCAWG project. We ran the GATK v3.3-0 CallableLoci tool with options maxFractionOfReadsWithLowMAPQ=0.25, maxDepth=1000, and otherwise default settings. Summarising the results across these 200 normal samples, we defined the callable genome space to be positions callable in >=40% of samples (>=20% on chrY), such that the length of non-callable regions must be at least 100bp, and the length of callable regions must be at least 300bp. The resulting callable genome covered 95.3% of non-N bases in hg19 (2.76Gb).

Genome property association testing for SV event classes

To test for association between SV event classes and the library of genome properties recorded in Supplementary Table 3, the genome property metrics were compared between real SV positions (randomly choosing one side of each breakpoint junction to reduce dependence between observations) and 1 million uniform random positions from the callable genome space. To compare the tissue-specific ROADMAP properties, each random position was assigned a random tissue type, drawing from the observed tissue type distribution in the SV call set. Note that the distance-type metrics were flipped to a negative scale so that positions close to the feature of interest scored higher than positions far away, and thus higher values correspond to signal enrichment.

For each genome property and each event class, the real observations were pooled amongst the random ones, then rank transformed and normalised on a scale from 0 to 1. Under the null hypothesis of no event-vs-property association, the ranks of the real observations would follow a uniform distribution. We tested this in each case with a Kolmogorov-Smirnov test then applied a Benjamini-Yekutieli correction for false discovery rate across the entire suite of tests and set the threshold for significance reporting at 0.01.

Defining the major fragile sites in the PCAWG dataset

We considered 109 literature-defined common fragile sites (ldCFS) defined in the Supplementary Materials from Bignell et al[7] and Le Tallec et al[8], lifting over to hg19 co-ordinates and using UCSC Genome Browser to find co-ordinates of cytogenic bands where necessary. Then we identified the longest protein-coding transcript overlapping each ldCFS (pctxCFS) and, pooling all samples in the cohort, calculated the observed deletion breakpoint density inside each ldCFS and pctxCFS, accounting for the number of bases considered 'callable' (see above). Given that more than 99% of 2Mb genomic bins outside the ldCFS had a deletion density <1e-4, we chose a fragile-site defining threshold of deletion density >1e-4 and absolute deletion breakpoint count >100. These criteria identified 17 fragile pctxCFS, and 1 fragile ldCFS with no significantly fragile pctxCFS.

For the significantly fragile ldCFS with a significantly fragile pctxCFS inside, when the pctxCFS was removed from the ldCFS they all ceased to meet the criteria of deletion density >1e-4 and deletion count >100. Thus, fragile site definition is taken to be the pctxCFS in these cases. The one fragile ldCFS without an explanatory transcript is the FRAXB/ HDHD1;STS locus. We defined the 18 major fragile sites in the PCAWG dataset as: a) the 17 significantly fragile protein-coding transcripts - rounding outwards either side to the nearest 100kb, and b) manual definition for FRAXB guided by the local deletion distribution.

Supplementary Table 5: Major fragile sites defined for the PCAWG dataset, in hg19 co-ordinates. Locations (chromosome and co-ordinates) of major fragile sites, the genes containing the fragile site, and the name of the fragile site. CFS, common fragile site.

chrom	start	end	width	gene name	CFS name
chr1	71800000	72800000	1000001	NEGR1	FRA1L
chr1	245800000	246800000	1000001	SMYD3	FRA1I
chr2	140900000	143000000	2100001	LRP1B	FRA2F

chr2	205300000	206600000	1300001	PARD3B	FRA2I
chr3	59600000	61300000	1700001	FHIT	FRA3B
chr3	115400000	117800000	2400001	LSAMP	FRA3L
chr3	174100000	175600000	1500001	NAALADL2	FRA3O
chr4	90900000	92600000	1700001	CCSER1	FRA4F
chr5	58200000	59900000	1700001	PDE4D	FRA5H
chr6	161700000	163200000	1500001	PARK2	FRA6E
chr7	69000000	70400000	1400001	AUTS2	FRA7J
chr7	110200000	111300000	1100001	IMMP2L	FRA7K
chr10	52700000	54200000	1500001	PRKG1	FRA10G;FRA10C
chr16	78000000	79300000	1300001	WWOX	FRA16D
chr20	13900000	16100000	2200001	MACROD2	FRA20B
chrX	6500000	8000000	1500001	HDHD1;STS	FRAXB
chrX	31000000	33500000	2500001	DMD	FRAXC
chrX	95800000	97000000	1200001	DIAPH2	FRAXL

HDP method for SV signature discovery

As input into the NMF method, the per-sample SV burdens were tallied by footprint type. The Hierarchical Dirichlet Process is a nonparametric Bayesian model that can perform mutational signature discovery across a tree of DP nodes organised to reflect sample groupings, and automatically learns the optimal number of signatures. Using hdp version 0.1.1 (an R package we developed applying HDP to signature analysis, available at https://github.com/nicolaroberts/hdp), we initialised a HDP structure with one common grandparent node, a parent node for each cancer histology type, and a child node for each cancer sample. Concentration parameters were shared between the children of each parent node, and were all drawn from gamma priors with hyperparameters at 1. We ran seven separate MCMC posterior sampling chains with 10,000 burn-in iterations and collected 250 posterior samples off each chain at intervals of 100. Results were pooled and summarised as signatures using the post-processing functions available in https://github.com/nicolaroberts/hdp.

Supplementary Results

Rearrangement clustering and interpretation statistics

After all filtering, the final datasets consisted of 274,515 SV calls. These were clustered into 130,438 SV clusters and 278,745 SV breakpoint footprints. Note that every SV has two breakpoints, and SVs residing in their own clusters without being clustered with any other SVs have two footprints by the convention of our clustering algorithm.

Out of all SV junctions, 111,273 (41%) formed their own single-SV cluster. Therefore, the remaining 163,242 SVs were grouped into multi-SV clusters, forming 19,165 SV clusters and 60,223 footprints. On average, one multi-SV cluster contains 8.5 SVs and 2.9 footprints.

A considerable proportion of SVs were classified differently to their naïve interpretation (Supplementary Table 6). Roughly one third of deletion and tandem duplication-type SVs were classified as another SV event type than a simple deletion or tandem duplication, respectively. For inversion and inter-chromosomal translocation-type SVs, only about one in ten of the SVs were classified as immediately suggested by their straightforward interpretation.

Supplementary Table 6: Raw SV junction counts and their final classification tallies in the PCAWG SV dataset. Counts of the common classes of SV junctions and possible subclassifications. Note that patterns in this table are counted regardless of footprints; e.g. an SV event was considered a direct inversion regardless of whether it was composed of one or two footprints.

SV junction type	Frequency	Classified as	Frequency	Percentage
Deletion (+-)	80,123	Deletion	54,363	67.8 %
		Other	25,760	32.2 %
Tandem duplication (-+)	69,096	Tandem duplication	45,758	66.2 %
		Other	23,338	33.8 %
Inversion (++ or --)	63,801	Reciprocal inversion	2,998	4.7 %
		Simple fold-back	1,791	2.8 %
		Dup-InvDup and Dup-Trp-Dup	1,324	2.1 %
		Other	57,688	90.4 %
Inter-chromosomal	61,495	Simple unreciprocal translocation	7,158	11.6 %
		Balanced translocation	1,232	2.0 %
		Other	53,105	86.4 %

Rearrangement footprints in cancer

In this section we identify and characterise the most recurrent somatic rearrangement patterns in cancer.

Footprints with single breakpoints

Footprints with a single breakpoint are the simplest footprints by definition. By our classification convention, simple deletions, tandem duplications, unbalanced inversions and unbalanced translocations are the only SV event types that have single-breakpoint footprint. Due to the simplicity of single breakpoint footprints, they serve well in demonstrating mechanistic inferences using statistical analysis of rearrangement patterns.

A deletion by definition deletes the section between its breakpoints. Thus, the rearrangement side CN of a deletion should on average have the same copy number as the respective chromosome arm copy number, whereas the non-rearrangement side copy number should be below the chromosome arm copy number. In contrast, tandem duplication rearrangement side and non-rearrangement side CN should be above and on the level of the overall background copy number. These expected patterns can indeed be seen in relative copy number analysis (Supplementary Figure 27). An unbalanced translocation retains the rearrangement side of the chromosomal arm but loses the non-rearrangement side, which maintains a relative CN of 0 on both sides. This pattern can also be seen in relative CN analysis (Supplementary Figure 27).

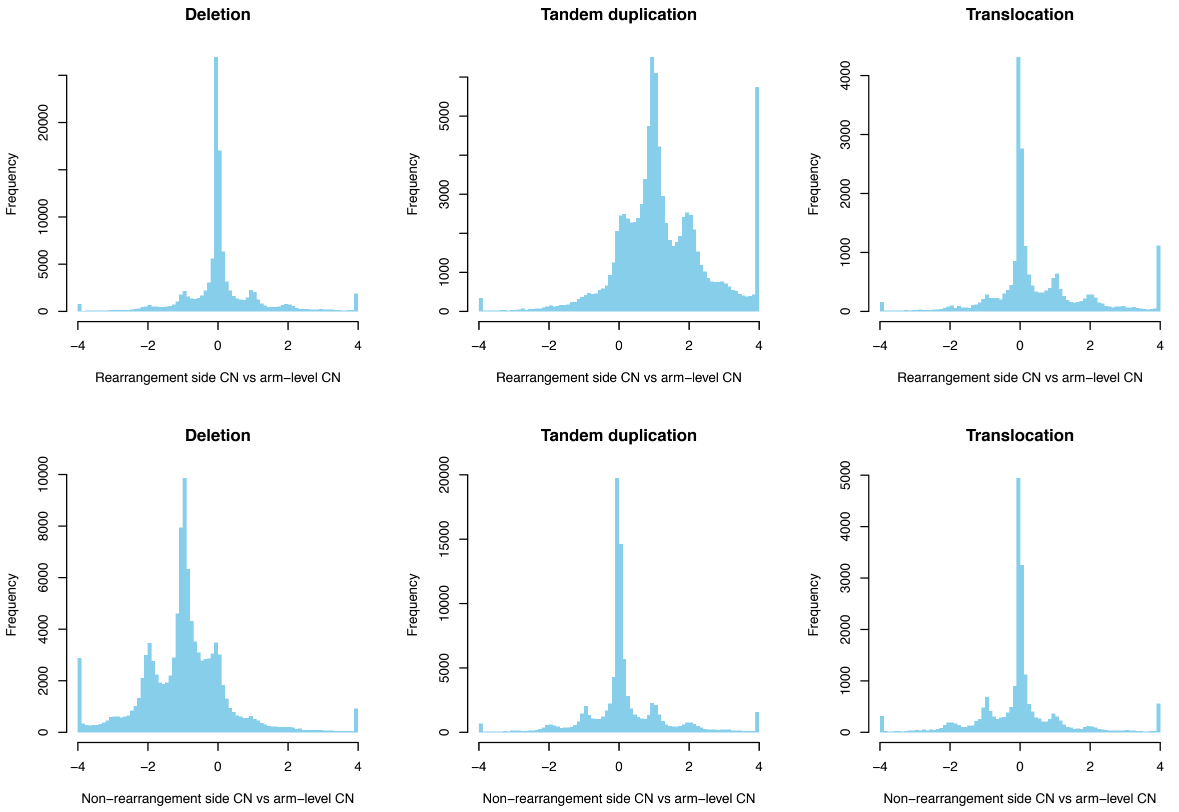

Supplementary Figure 27: Relative copy numbers of rearrangement ends of deletions, tandem duplications and translocations. The rearrangement side of deletions is on average on the same level as the background arm-level copy number, whereas the non-rearrangement side tends to be one copy below the background. For tandem duplications, rearrangement side CN is one copy above and non-rearrangement side CN is on the same level as the background CN. Unbalanced translocations tend to lead to a partial loss of a chromosomal arm. The rearrangement side of the arm is not lost, and thus rearrangement side CN averages the same CN as the background CN. Similarly, non-rearrangement side CN causes the loss of the non-rearrangement side of the chromosomal arm, and thus the non-rearrangement side CN of a translocation is on average at the same level as the respective background arm level. See Supplementary Methods for the definitions of rearrangement and non-rearrangement side and arm-level CN.

Footprints of two breakpoints from different rearrangements

Two breakpoints can be arranged into four different pairs of orientations: +-, -+, ++ and --. Deletions and tandem duplications by convention of our classification scheme have two single-breakpoint footprints. Footprints of rearrangement end orientations ++ or -- with a single SV are fold-back SVs.

The rest of the two-breakpoint footprints have footprints have each of their footprints derived from a different SV. The A+/C- and B-/B+ footprints are consistent with *balanced breakpoints* and *templated insertions*, respectively. The A+/A+ footprints are unphased, since a single rearrangement event cannot create two distinct breakpoints of the same orientation without additional breakpoints in between. Note that the balanced breakpoint footprint also has two obligatorily unphased breakpoints. The difference is that balanced rearrangements are a well-known mechanism for generating 'A+/C-'-type footprints.

When the frequencies of different pairs of orientations are plotted out (only footprints involving two different SVs), the enrichment of +- and -+ type footprints is evident (Supplementary Figure 28). If the rearrangement breakpoints formed the footprints randomly, one would expect a uniform distribution of orientation pairs. The enrichment of '+-' and '-+'-type footprints suggests that the SV and footprint clustering algorithm is capturing real biological SV patterns.

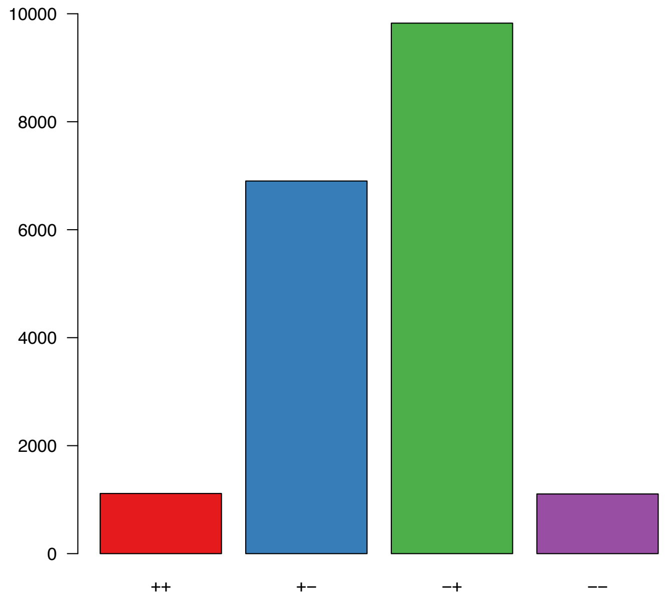

Supplementary Figure 28: Frequency of footprints with two rearrangement junctions from distinct SVs. The orientation of the two joined ends in the breakpoint junction are shown on the x axis, and counts on the y axis.

If the ‘B-/B+’-type footprints really represent templated insertions, they should have an increased relative copy number compared to the chromosome arm-level background level. Indeed, relative copy number shows that footprints classified as templated insertion has a strong tendency to have one or more copies above the background arm-level CN (Supplementary Figure 29). In contrast, balanced rearrangement breakpoints tend to have the same copy number as the background chromosomal arm (Supplementary Figure 29).

Another prediction of a templated insertion is that the two breakpoints are phased and generated in a single rearrangement event. If this was the case, they should have the same rearrangement CN vast majority of the time. Indeed, we found this to be the case, as the difference in CN between the low end and high end of B-/B+ footprint breakpoints is clearly unimodal and centred at 0 (Supplementary Figure 30).

Crucially, the relative CN of unbalanced translocation breakpoints are centred at around 0 (Supplementary Figure 27), meaning that unbalanced translocations, as expected, tend to have the same copy number as the background arm-level CN. This is clearly in contrast to the relative CN of the breakpoints that are part of B-/B+ footprints, as they most commonly have one copy above the background (Supplementary Figures 29, 31). The enrichment of ‘B-/B+’-type footprints (Supplementary Figure 28) together with the tendency for copy number gain in these footprints strongly support the argument that the B-/B+ footprints we have found are not truly reflect a replicated, templated insertions as opposed to unrelated unbalanced translocations being merely inadvertently grouped together as footprints.

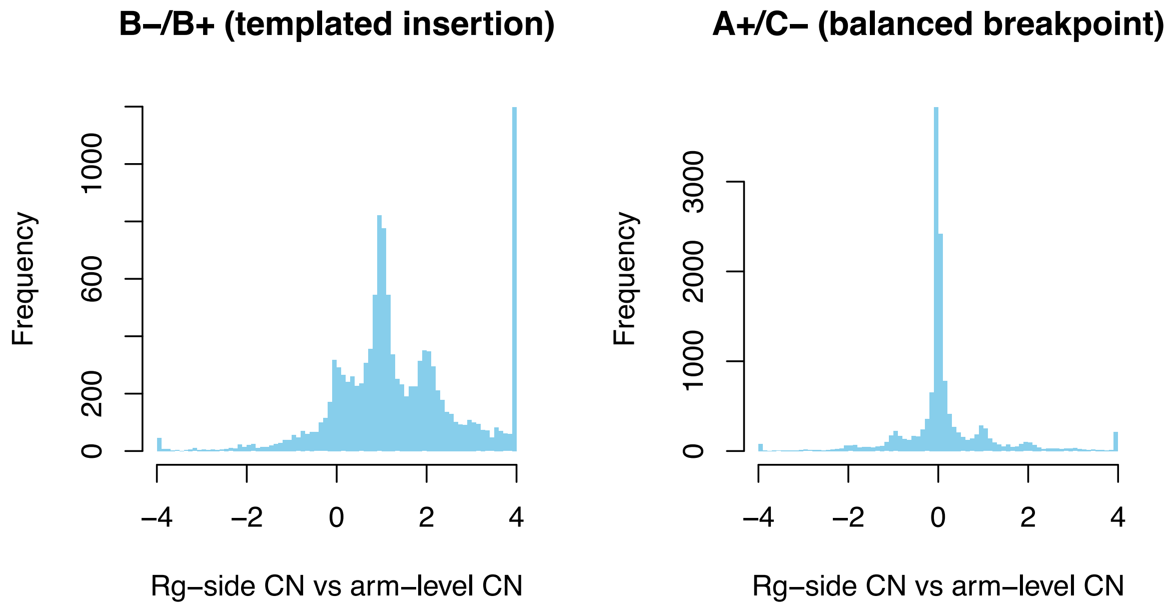

Supplementary Figure 29: Relative copy number of templated insertion and balanced breakpoint-type footprints. For B-/B+ type footprints, only those with a distance of ≥1kb between the breakpoints were considered, as copy number estimates become very noisy for segments smaller than that.

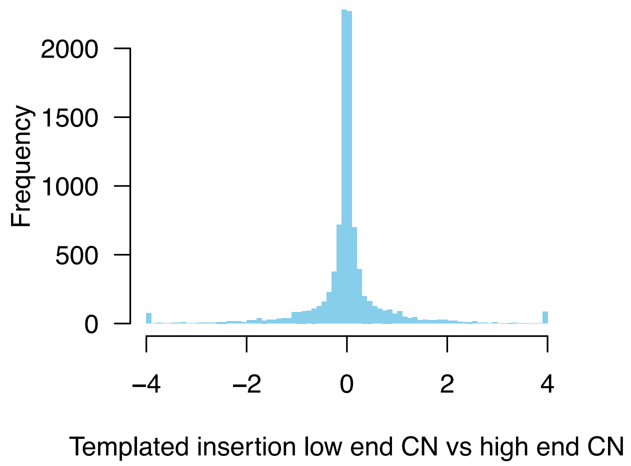

Supplementary Figure 30: CN difference between low end and high end SVs of templated insertion (B-/B+) footprints. The x axis shows the difference in copy number between the two ends of a templated insertion. Note that the difference is typically 0, as would be expected if these are templates inserted into the genome.

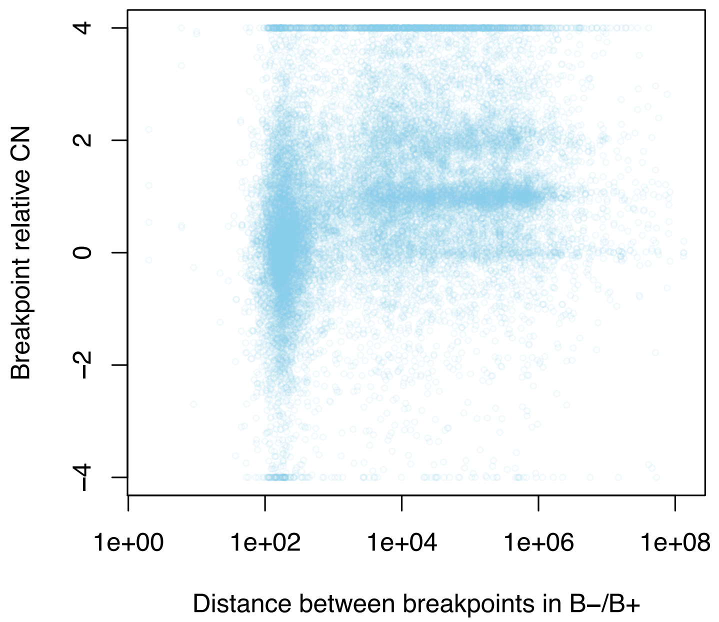

Supplementary Figure 31: Distance between the demarcating breakpoints in B-/B+ footprints vs relative CN of the segment. At smaller segment sizes the CN estimates are very noisy, but as the segment size increases, one can see that the rearrangement CN tends most commonly to be one copy above, and almost never below, the background arm-level CN.

Balanced rearrangements and chromoplexy

We found a total of 6,902 balanced breakpoint footprints in the cohort. Of these, 907 (13%) had an overlap in the supporting reads of the two balanced breakpoints due to breakpoint homology, and thus had a breakpoint pattern consistent with templated insertions (Supplementary Figure 14). By considering the exact positions of the supporting reads' soft-clips, we were able to correctly classify these 907 footprints as balanced breakpoints as opposed to templated insertions.

Balanced breakpoints are typically attributed to balanced translocations. However, we found that a substantial fraction of balanced breakpoints in isolation, not as part of balanced translocations (Supplementary Figures 32-33). The two types of balanced breakpoints also do not always correlate. For example, whereas Breast-DCIS has a large number of non-reciprocal balanced breakpoints (Supplementary Figure 33), it has very few actual balanced translocations (Supplementary Figure 34).

As reported previously[9], we found long chains or cycles of balanced breakpoints in prostate cancer but also in other cancer types. In addition, thyroid cancer appears to have particularly long cycles of balanced breakpoints (Supplementary Figure 32), suggesting that chromoplexy might occur relatively frequently in this cancer type.

In the entire PCAWG cohort, 1,622 out of 4,793 unreciprocal balanced breakpoint footprints are simple unreciprocal balanced breakpoints. 623 unreciprocal balanced breakpoint footprints are part of SV clusters containing ≥50 SVs.

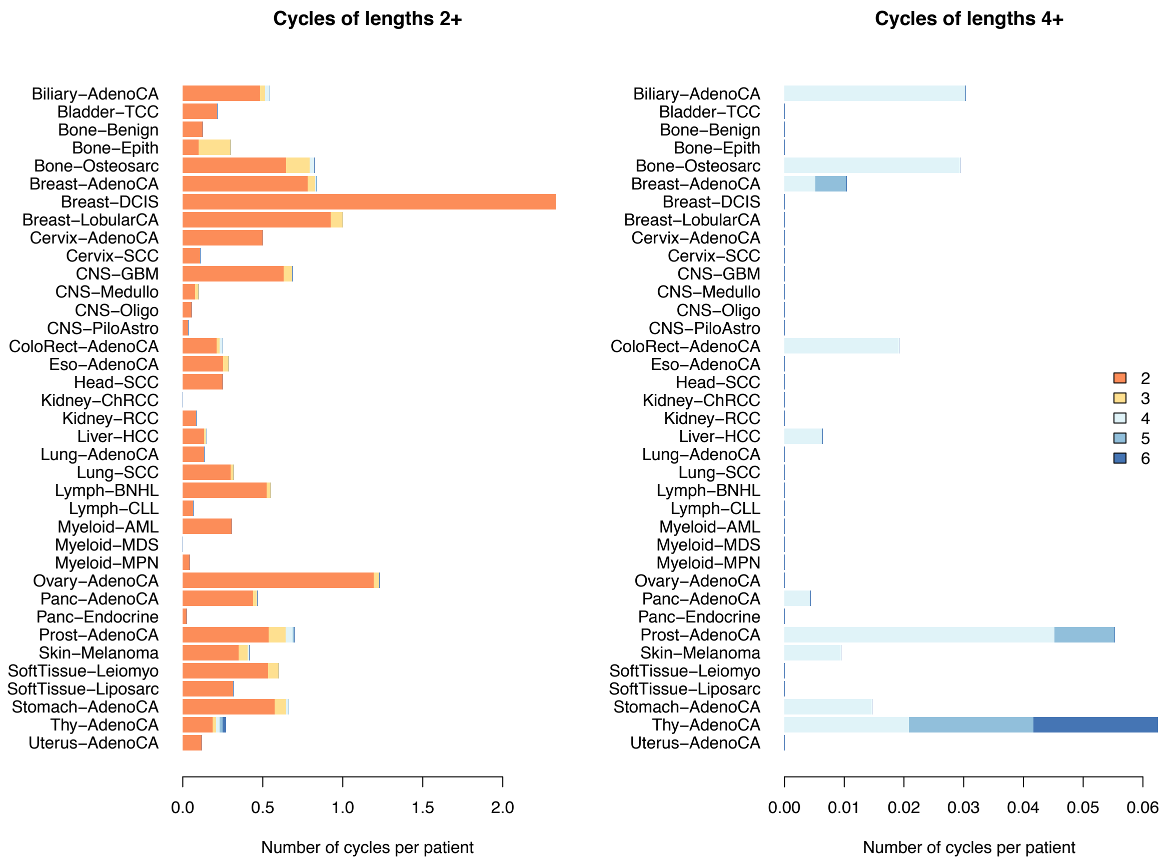

Supplementary Figure 32: Per-patient balanced breakpoint cycle lengths. Stacked bar chart showing the average number of balanced breakpoint cycles of different lengths per patient, split by tumour type (y axis). The right-hand side panel is a zoomed-in version of the left-hand side panel.

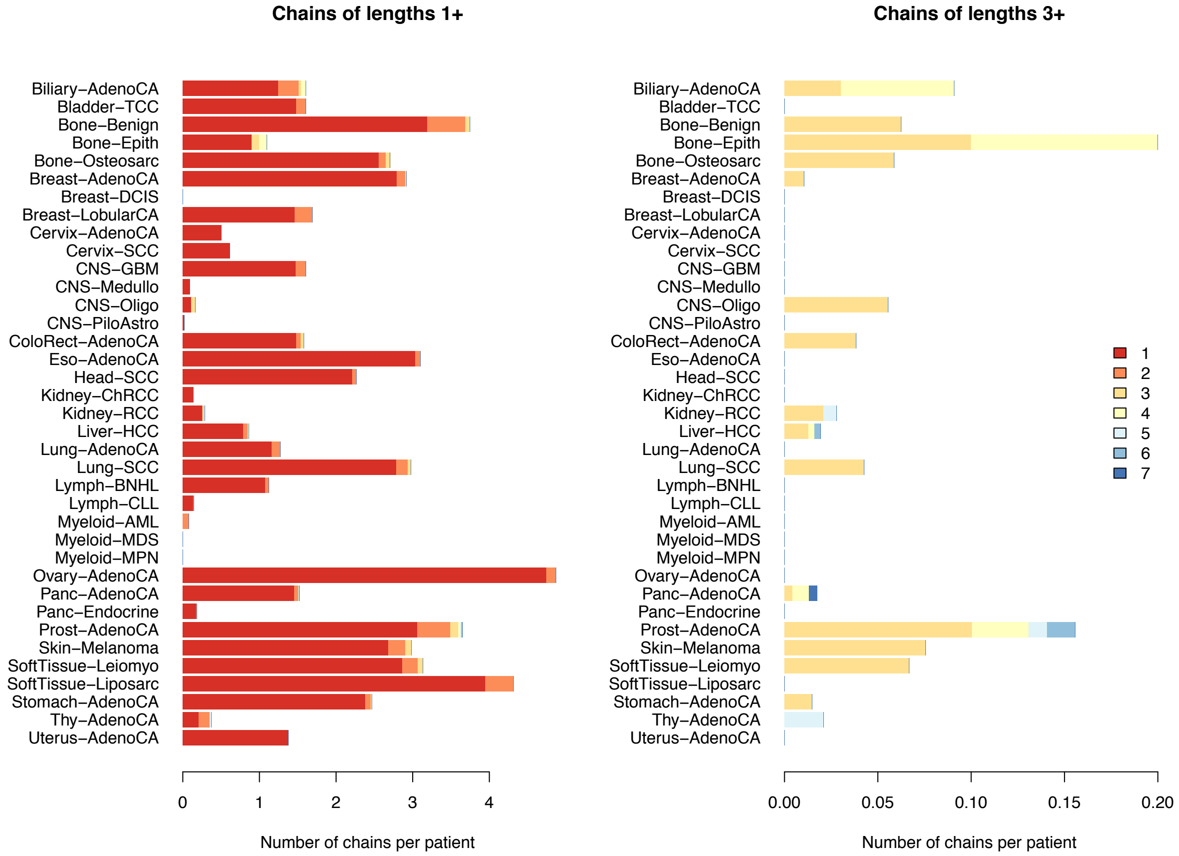

Supplementary Figure 33: Per-patient balanced breakpoint chain lengths. Stacked bar chart showing the average number of balanced breakpoint chains of different lengths per patient, split by tumour type (y axis). The right-hand side panel is a zoomed-in version of the left-hand side panel.

The distance between balanced breakpoints at balanced breakpoint footprints peaks at around 10-100bp, in particular in singleton unreciprocal balanced translocations. In certain histologies, another smaller peak of balanced breakpoint distances can be seen at around 100kb, e.g. in prostate cancer, pancreatic adenocarcinoma and oesophageal adenocarcinoma (Supplementary Figure 34).

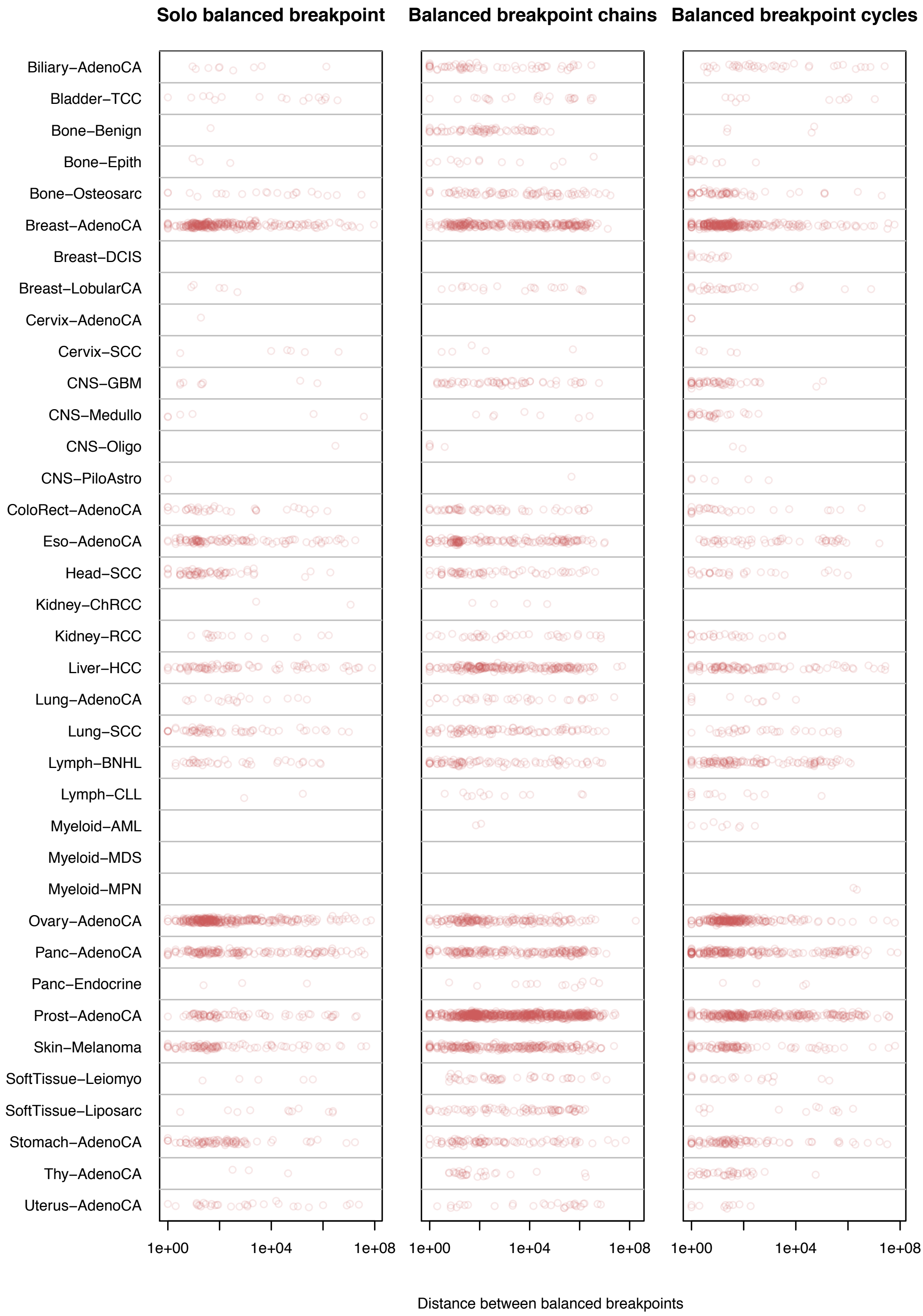

Solo balanced breakpoint
Balanced breakpoint chains
Balanced breakpoint cycles
Biliary–AdenoCA
Bladder–TCC
Bone–Benign
Bone–Epith
Bone–Osteosarc
Breast–AdenoCA
Breast–DCIS
Breast–LobularCA
Cervix–AdenoCA
Cervix–SCC
CNS–GBM
CNS–Medullo
CNS–Oligo
CNS–PiloAstro
ColoRect–AdenoCA
Eso–AdenoCA
Head–SCC
Kidney–ChRCC
Kidney–RCC
Liver–HCC
Lung–AdenoCA
Lung–SCC
Lymph–BNHL
Lymph–CLL
Myeloid–AML
Myeloid–MDS
Myeloid–MPN
Ovary–AdenoCA
Panc–AdenoCA
Panc–Endocrine
Prost–AdenoCA
Skin–Melanoma
SoftTissue–Leiomyo
SoftTissue–Liposarc
Stomach–AdenoCA
Thy–AdenoCA
Uterus–AdenoCA
1e+00
1e+04
1e+08
Distance between balanced breakpoints

Supplementary Figure 34: Distance distributions between balanced breakpoints at balanced breakpoint footprints. Shown are SV clusters classified as solo balanced breakpoints comprise of two SVs forming a non-reciprocal translocation and not clustering with any other SVs. Each point comprises one such event, with distance on the x axis, split by tumour type on the y axis.

Templated insertions

Most templated insertions occur in isolation without being part of a larger templated insertion chain or cycle. However, sometimes chaining of templated insertions can reach a length of 7 templated insertions (Supplementary Figure 35).

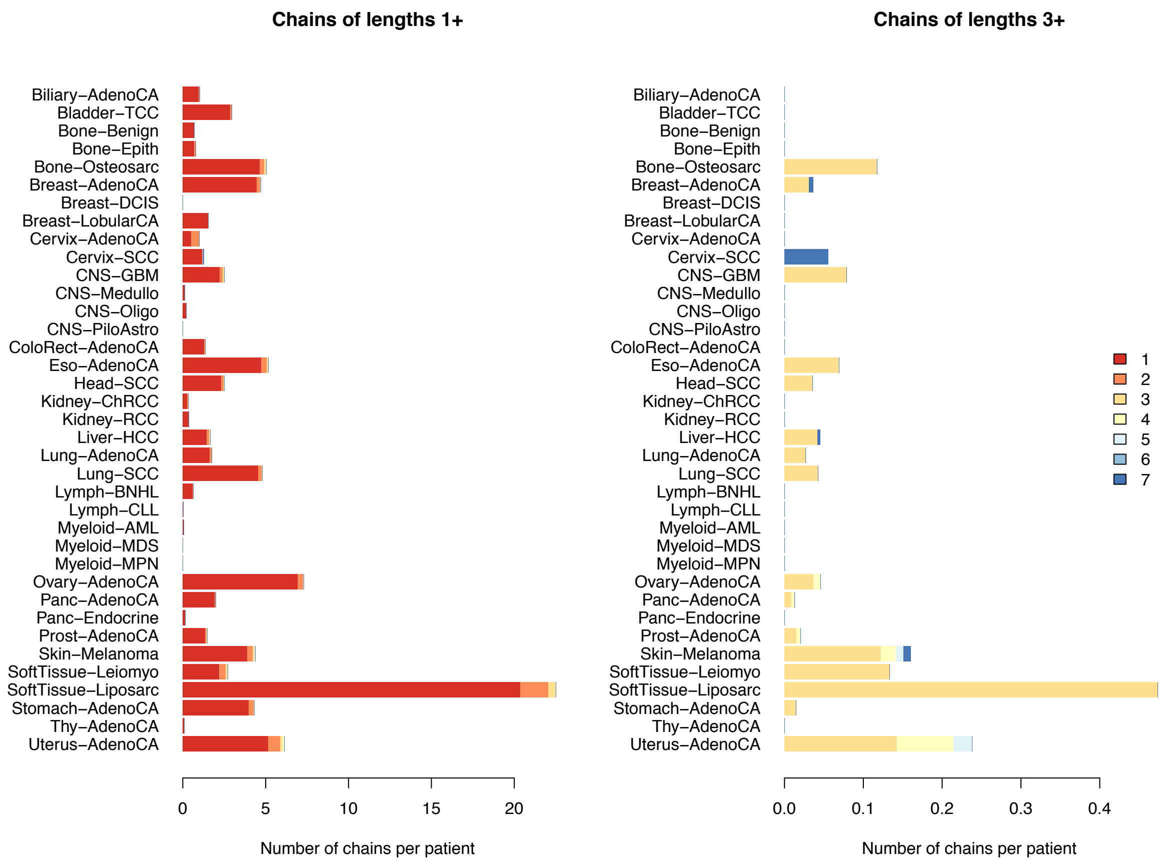

Supplementary Figure 35: Distribution of templated insertion chain lengths in each histology. Stacked bar chart showing the average number of chains of templated insertions of different lengths per patient, split by tumour type (y axis). The right-hand side panel is a zoomed-in version of the left-hand side full distribution.

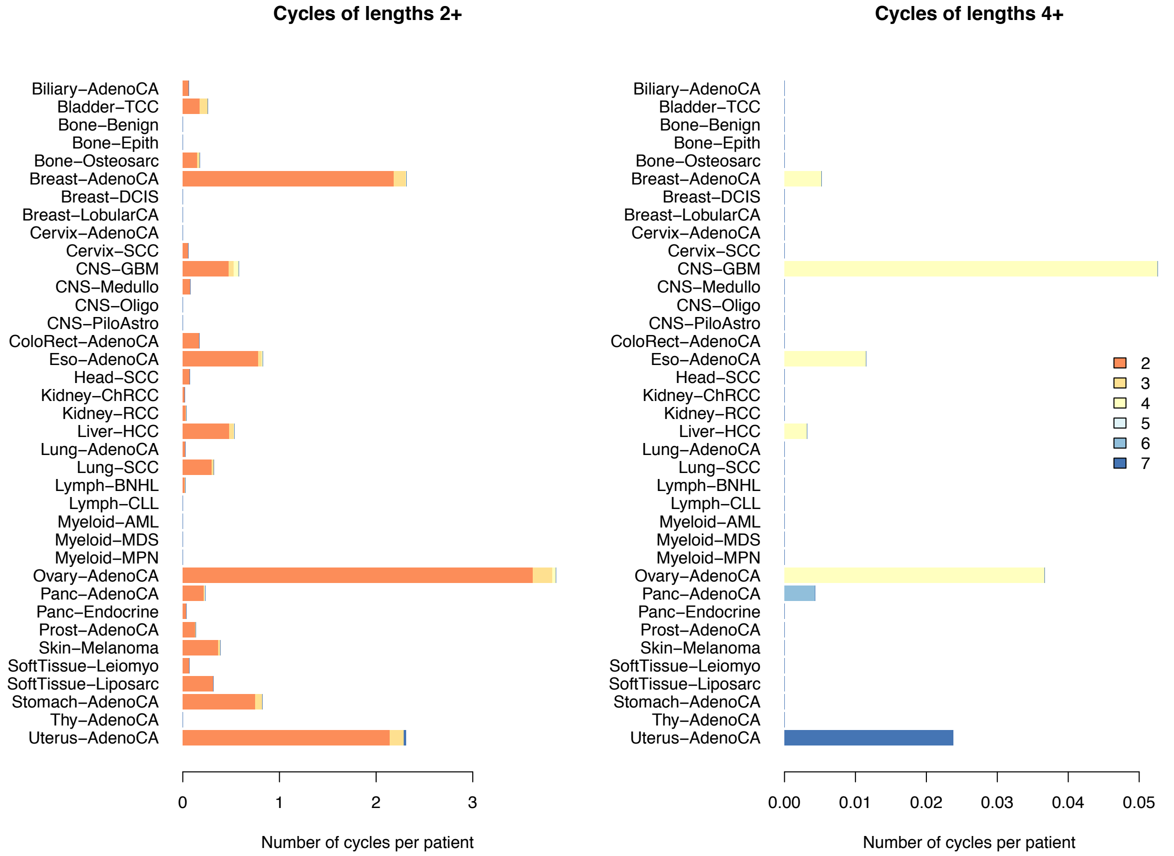

Supplementary Figure 36: Distribution of templated insertion cycle lengths in each histology. Stacked bar chart showing the average number of cycles of templated insertions of different lengths per patient, split by tumour type (y axis). The right-hand side panel is a zoomed-in version of the left-hand side full distribution.

Most templated insertion cycles are formed of two templated insertion footprints. However, longer cycles of templated insertions do occur sometimes: we found a single uterine cancer sample with a cycle of seven templated insertions (Supplementary Figure 36).

Interesting patterns can be gleaned from the distribution of templated insertion chain and cycle lengths and size distributions. Firstly, all histologies with substantial amounts of simple templated insertions typically also have templated insertion chains and cycles (Supplementary Figure 37). The contrary is not true however, as exemplified by the high frequency of templated insertion chains but absence of simple insertions or templated insertion cycles in bone leiomyoma (Supplementary Figure 37). Since bone leiomyoma has a high rate of templated insertions (Supplementary Figure 37), but they are not simple insertions (Supplementary Figure 37), the implication is that complex rearrangements in bone leiomyoma often get repaired with templated insertions at the repair junctions.

The sizes of simple insertions are typically one to few hundred base pairs (Supplementary Figure 37). Ovarian cancer, breast adenocarcinoma and liver cancer form an exception in that simple insertions in these cancers can often reach 10kb (Supplementary Figure 37).

The size distribution for templated insertions within large SV clusters appear to form two clusters, one in the <1kb size range and another peaking around 100kb (Supplementary Figure 37). Yet more different is the size distribution of templated insertions that form cycles, which

in several cancer types (e.g. oesophageal, pancreatic, skin, stomach and uterine cancer) tends to fall into the ~100kb cluster more often than the <1kb cluster.

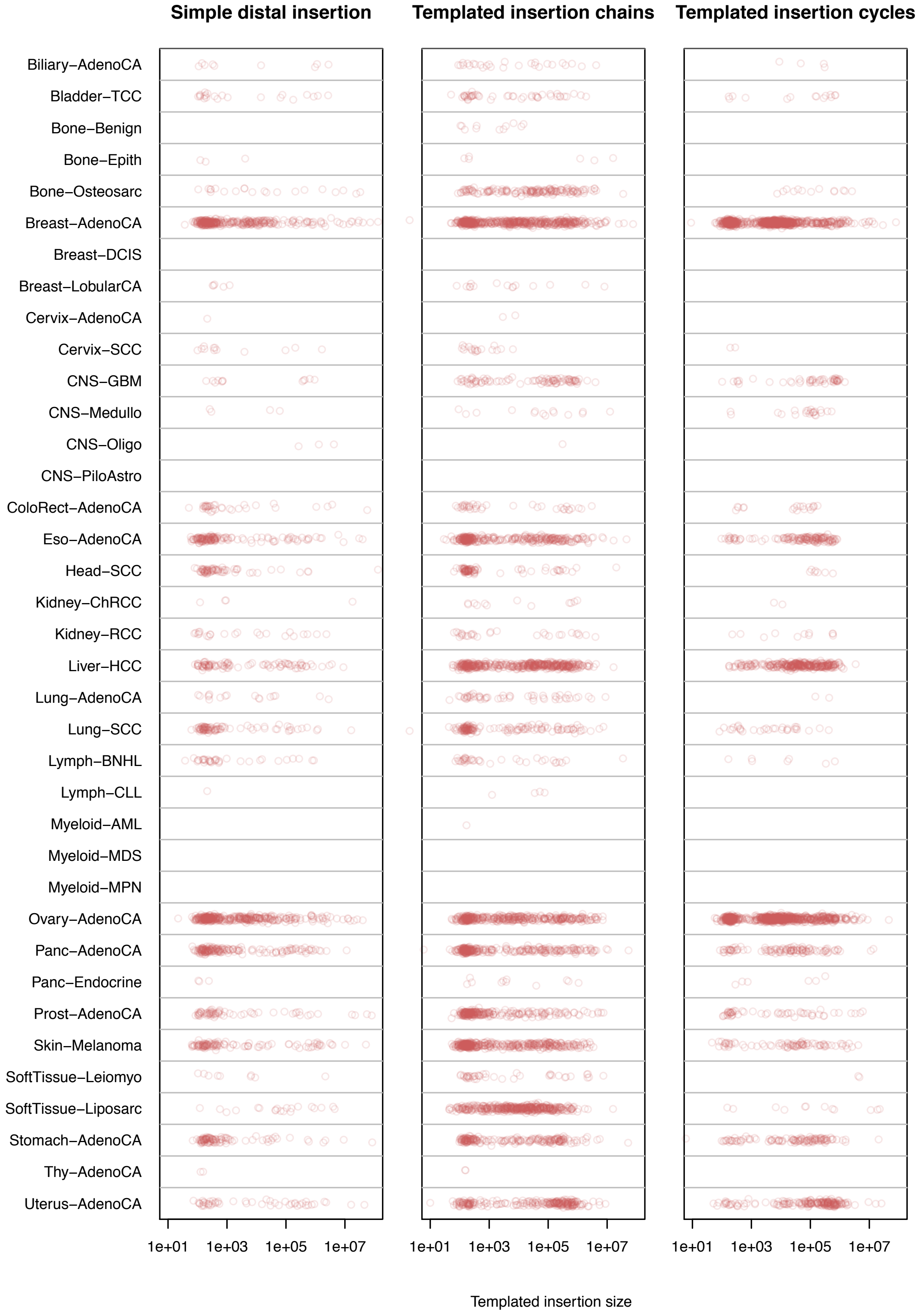

Supplementary Figure 37: Size distribution of templated insertions. Shown are simple distal insertions are events comprising a simple templated insertion from a distal location. Each point comprises one such event, with distance on the x axis, split by tumour type on the y axis.

We found a total of 1,372 instances of cycles of two templated insertions. In addition, we found 88 instances two 'B-/B+'-type footprints linked together as a cycle, but with a copy number pattern consistent with an unbalanced translocation followed by an overlapping tandem duplication (see Supplementary Methods). Were all 'B-/B+'-type footprints interpreted naively, the unbalanced translocation plus TD pattern, representing about 6% of all cycles of two B-/B+ footprints, would have been misclassified.

Footprints with four breakpoints

We will now postpone our study of footprints with three breakpoints and focus on footprints with four breakpoints. We found a total of 6,646 footprints involving four breakpoints in the PCAWG cohort (Supplementary Table 7).

Supplementary Table 7: The 40 most frequent footprints involving four breakpoints in the PCAWG dataset, with descriptions on some notable patterns. Note that these footprint counts include both solo footprints that for their own SV cluster as well as footprints as part of larger SV clusters.

Footprint pattern	Frequency	Description of pattern
A+^C+/C-^E-	1227	Reciprocal inversion
B-^D-/B+^D+	531	Interlocked inverted duplication
B-/B+/D-/D+	507	Two templated insertions
A+/C-/C+/E-	478	Two balanced breakpoints
A+^D+/C-^D-	472	Inserted inverted duplication
B-^C-/C+^D+	349	Duplication – inverted triplication – duplication
A+/C-/D-/D+	213	Balanced breakpoint and templated insertion
B-/C-/C+/D+	158	Nested templated insertions
A+^C+/C-/E-	142	Local + distal reciprocal inversion
A+/B+/D-/D+	135	
B-^D-/B+/D+	134	Local + distal inverted duplication
A+^E-/C-/C+	123	Templated insertion within a deletion
A+/C-/C+/D+	121	
A+^B+/C+^D+	115	Two unphased fold-back SVs
A+^D-/B+^D+	105	
B-/B+/C+/D+	95	
A+^D+/B+^D-	88	
B-^D+/B+^C+	88	

A+^C+/B+^E-	86	
B-^D+/C-/C+	81	Templated insertion within a TD
A+^C+/C-^D+	79	
B-^C+/C-/D+	72	
B-^C-/C+/D+	69	Local + distal dup-inv trp-dup
B-^D+/B+/D-	67	Balanced breakpoint within TD
B-/B+^D-/D+	65	Deletion within templated insertion
B-^C+/B+^D+	64	
A+^B+/D-^E-	54	
A+/C-^D-/D+	41	
A+/C-/C+^D+	38	
A+/B+/C+/E-	37	
B-^C-/D-/D+	37	
A+^E-/B+^C+	34	
B-/C-^C+/D+	34	
A+^D+/C-/D-	33	Local + distal insertion of inverted duplication
A+^D+/B+^C+	32	
A+^D+/C-^C+	31	
A+^B+/D-/D+	30	
A+/B+^D-/D+	23	
A+^D-/C-/D+	23	
A+^C+/B+^D+	22	

Local events involving two inversion-type SVs

The most frequent of these events are all phased in a sense that a derivative chromosome can walk through all the SV junctions of the footprint. We thus term such events “local two-jumps”. The most frequent footprint involving four breakpoints is that of the reciprocal inversion. Interestingly, there three other footprint types also involving two inversion-type SVs but consistent with copy number gain were frequently observed in the cohort (Supplementary Figure 38).

Note that for the B-^C-/C+^D+ pattern to be classified as a duplication – inverted triplication – duplication event, we required the two involved SVs to have the same copy number. When one of the inversion SVs has twice the copy number as the other one, the B-^C-/C+^D+ pattern was classified as a footprint with two rounds of fold-back SVs derived from BFB. See Supplementary Methods for details.

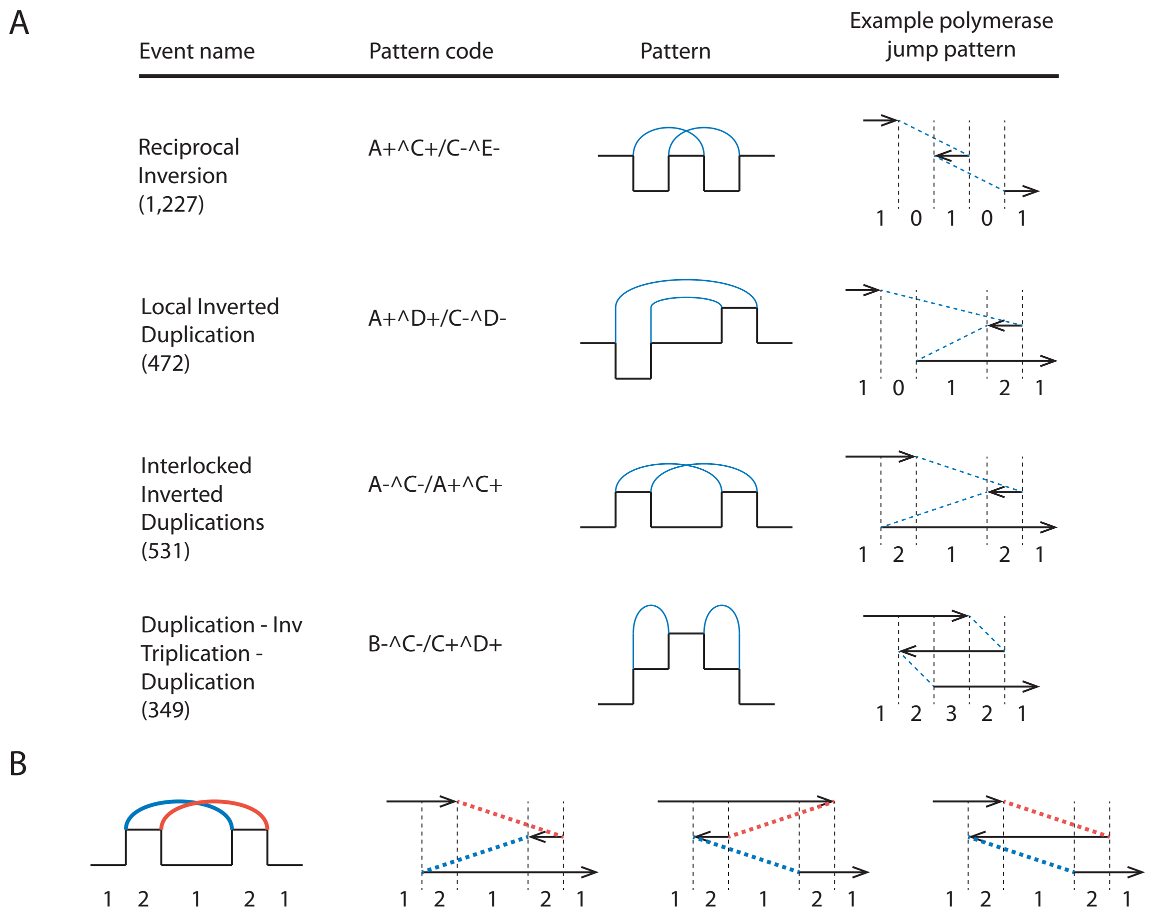

Supplementary Figure 38: (A) SV clusters consisting of a single footprint involving two inversion-type SVs. The numbers in parenthesis indicate the number of each respective event in the PCAWG cohort. (B) Three possible trajectories that a polymerase can take to generate the inverted duplication pattern. Numbers under each schematic indicate the copy number of each section. Note that the frequencies here differ from those in Supplementary Table 7, because here only footprints constituting an entire SV cluster are counted.

The relative copy number of these footprint types are shown in Supplementary Figure 39. As expected, the rearrangement breakpoints in reciprocal inversions are largely at the same CN level as the background arm-level CN. In local inverted duplications, there can be seen an enrichment of breakpoints with an increased copy number compared with the background. In the “interlocked inverted duplication” and “duplication – inverted triplication – duplication”, majority of the breakpoints are at a higher copy than the background chromosomal arm. In interlocked inverted duplication events, the prevailing relative CN is a single copy above the background, whereas in duplication – inverted triplication – duplication, the average relative CN is at around 1.5 copies. Therefore, in each of the patterns except the reciprocal inversion, relative CN analysis shows that these “local two-jump” patterns involve CN gain.

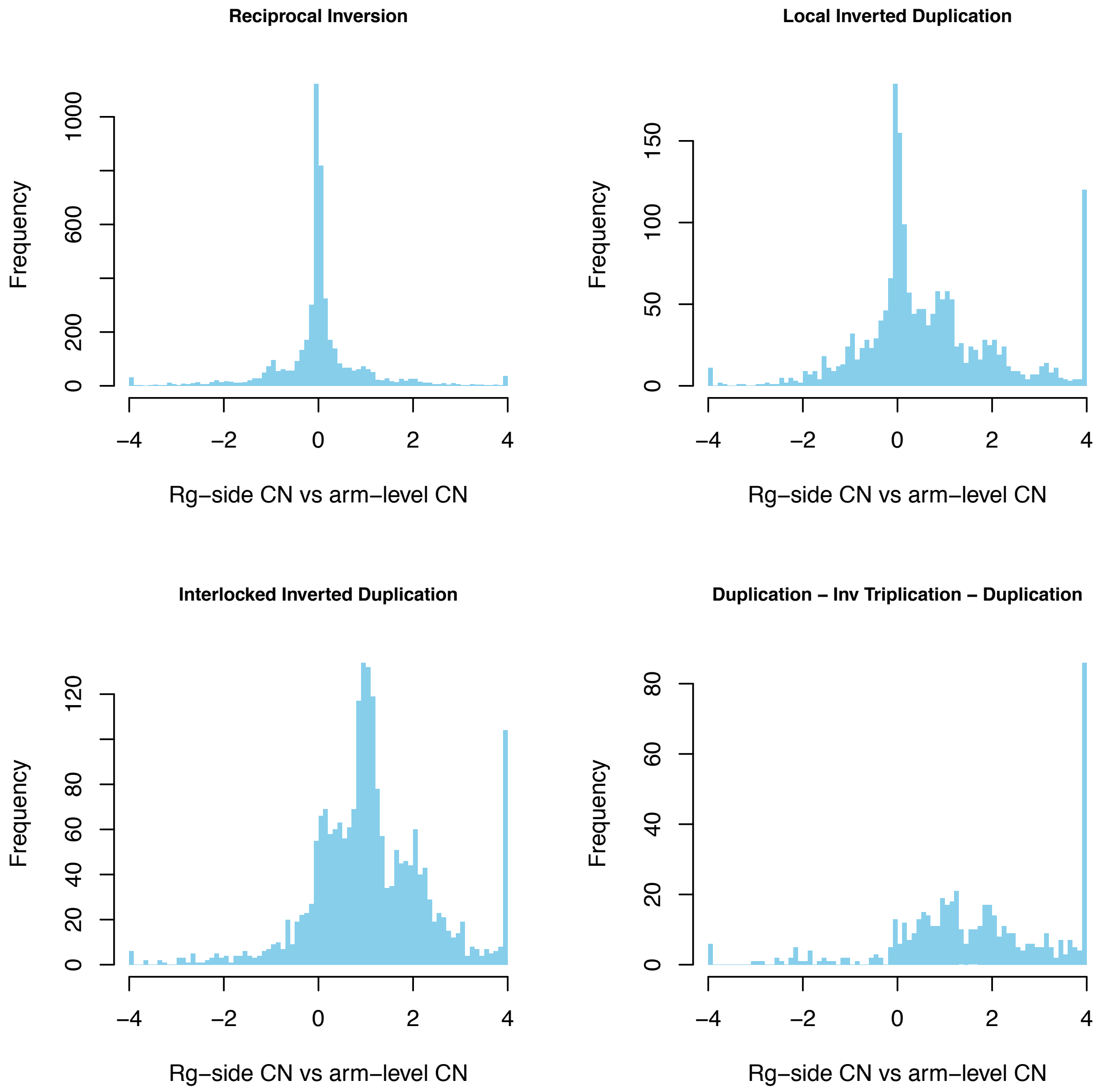

Supplementary Figure 39: Relative CNs of rearrangement breakpoints involved in local footprints comprising two inversion-type SVs. Frequency distribution of the copy number differences of local inversion-type footprints. The copy number difference between the copy number inside the rearrangement versus the background copy number of the chromosome arm is shown on the x axis.

What rearrangement event or sequence or rearrangement events could generate these inverted two-jumps with copy number gain? Different models could explain these patterns. The inversions could be

1. fold-back inversions derived from BFBs,
2. generated through a stepwise process through simpler “classical” rearrangement mechanisms,
3. in theory also arise from an extrachromosomal ring.

In the following sections, we examine the above hypotheses one by one and show that they cannot satisfactorily explain the CN patterns and frequency of these inverted CN-gain

footprints. As an example, we focus on the interlocked inverted duplication pattern, but similar reasoning can be applied to the other inverted two-jump CN-gain patterns.

The inverted copy number gain patterns are poorly explained by BFB-associated fold-back rearrangement events

In theory the footprints presented above could be explained as two fold-back type inversion rearrangements being clustered together. However, several lines of evidence presented below argue against this alternative model as the mechanism behind the CN-gain inverted two-jumps.

If the two inversion-type rearrangements were in phase, i.e. in the same derivative chromosome, then given their proximity it is highly likely that majority of such footprints arose through two cycles of a single BFB cycle event. If this was the case, the copy number segment immediately upstream of the footprint should have a different copy number compared to the segment immediately downstream of the footprint[1]. This requirement is mandatory if one of the two inversion-type rearrangements were the initiating fold-back rearrangement of a BFB cycle. As expected, two rounds of BFBs often involve a CN change from the segment upstream to the segment downstream of the footprint (Supplementary Figure 40). In contrast, almost none of the inverted CN-gain two-jump footprints involve a copy number change across the footprint. This suggests that the footprints classified as local inverted duplication, interlocked inverted duplication and dup – inv trp – dup cannot be explained by clusters of two FBs caused by BFBs.

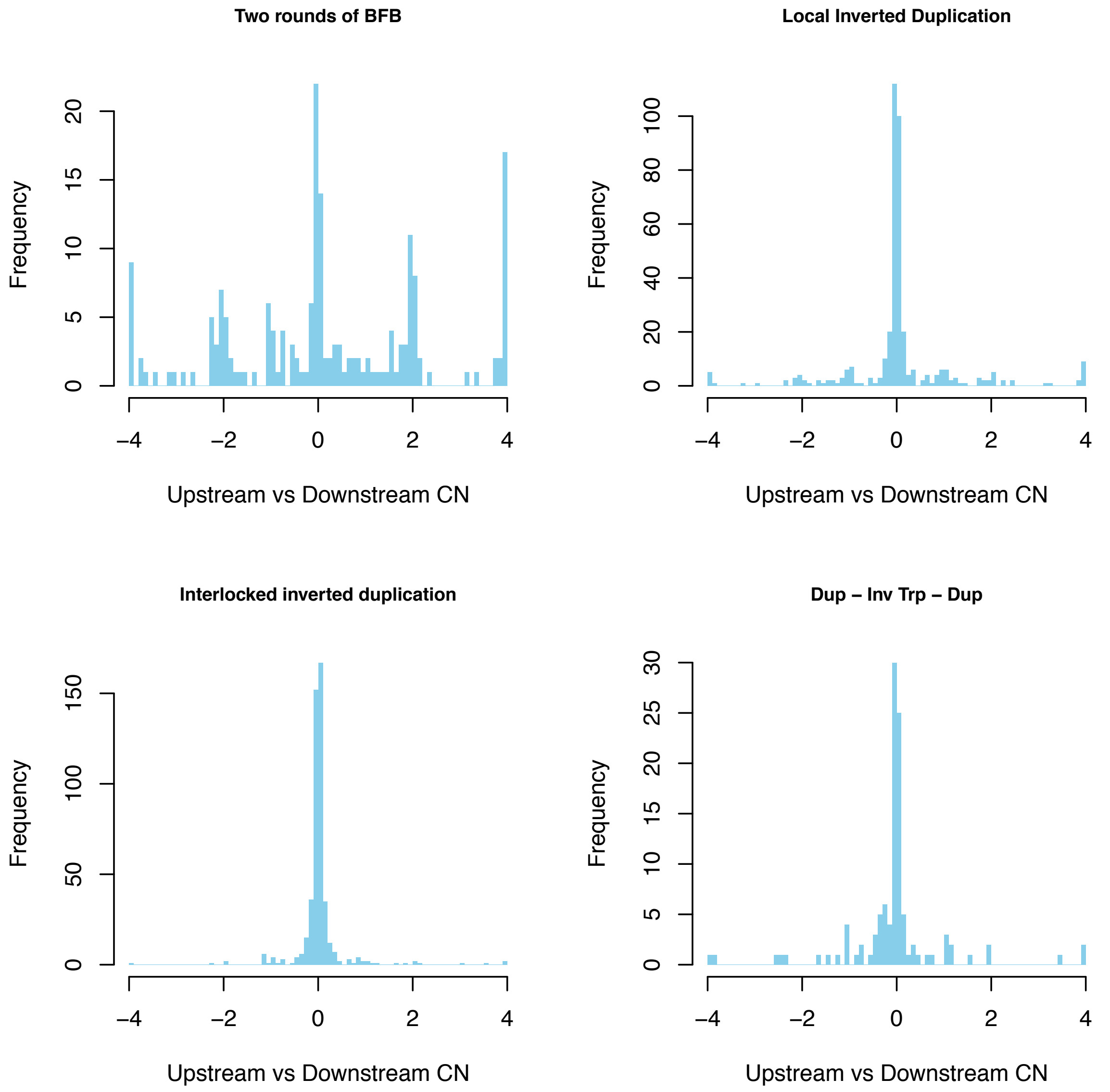

Supplementary Figure 40: Difference in copy number between segments directly upstream and directly downstream of the footprints. Only footprints with both upstream and downstream segments >100kb are considered. Footprints of two successive fold-backs (top-left) with a copy number difference of around 0 copies might be misclassified, patterns, for example when a breakpoint is missing within the footprint.

Alternatively, it is possible that in the CN-gain two-jump footprints the two inversion-type SVs are generated through *independent* BFBs, which got erroneously clustered together into a footprint given their proximity by chance. Two have two arguments for why this is unlikely to be the case. First, of the footprints involving two inversion SVs of opposite orientations, the three most frequent cases (B-^D-/B+^D+, A+^D+/C-^D- and B-^C-/C+^D+) can in theory be phased. If these footprints were merely of unphased and erroneously clustered inversion SVs, one would expect their frequency to the equal to the obligate unphased patterns involving two inversion-type SVs (A+^B+/C+^D+ and A+^B+/D-^E-). However, this is not the case: while we found hundreds of the former three patterns (531, 472 and 349, respectively), there were an order of magnitude lower number (115 and 54, respectively) of the latter unphased patterns (Supplementary Table 7).

Secondly, if two inversions in a footprint were unphased, then by definition the two inversions must be on different derivative chromosomes. In addition, since a fold-back inversion connects two homologous chromosomes, two unphased fold-back inversions of opposite orientation must involve two chromosomal copies on either side. However, we did find several examples of the above described footprints on a single copy background (Supplementary Note 4). In these cases, the only possible explanation is that the two inversion-type rearrangements are in the same derivative chromosome.

The inverted copy number gain patterns are unlikely to be generated through a stepwise application of independent simple rearrangement events

Two key aspects of the local inversion footprints are presented above. First, they involve two inversion rearrangements of opposite orientations, and secondly, they involve copy number gain with respect to the background chromosomal CN. Could such footprints be generated through a sequence of simple events. After all, reciprocal inversions can generate inverted SVs, and tandem duplications could generate CN gains.

In order to assess this model, we computed a list of all possible derivative chromosome patterns obtainable through a sequence of up to five simple SVs (such as reciprocal inversions, deletions and tandem duplications; see Supplementary Methods for details) that could generate the inverted CN-gain two-jump footprint patterns. It turns out that this pattern can indeed by generated through sequential application of classical rearrangement events (Supplementary Note 4). The insertion of inverted duplication and interlocked inverted duplication patterns can be generated through tandem duplication followed by an interstitial inversion and a deletion (Supplementary Note 4), with the difference in the position of the deletion breakpoints relative to the earlier SV breakpoints. The duplication – inverted triplication – duplication pattern can be generated in a similar fashion, except it requires two initiating tandem duplications to bring the maximum CN to 3 copies first.

Interestingly, each of these parsimonious sequences generating the inverted copy number gain patterns involve an intermediate state with a direct inversion nested within a tandem duplication. Therefore, if the observed inserted copy number gain patterns indeed arose through such sequences of simpler events, then there must have been an intermediate state involving a direct inversion nested within a tandem duplication. Furthermore, since deletions overall cover only a small fraction of a given tumour genome, only few direct inversions within tandem duplications are expected to be afflicted by a subsequent deletion, whereas most of them are expected to remain in this intermediate state. In other words, if the inverted copy number gain patterns were indeed generated through these sequences of simple events, then one would expect to observe a large number of these intermediate states involving direct inversions nested within a tandem duplication.

In contrast to this prediction, across tumours in all tissue types we only observed 33 instances of direct inversions nested within TDs, which is an order of magnitude lower than the supposed downstream product manifesting as inverted CN gain patterns. The high frequency of the inverted CN gain patterns relative to the supposed intermediate structures that led to them contradicts the hypothesis that they are generated through these sequences of simpler events.

The local copy number gain patterns are poorly explained by other well characterised rearrangement processes

Any two given SVs can be trivially generated through two independent unbalanced translocations. In the case of local inverted two-jump patterns, both unbalanced translocations would create fold-back-type inversions between two homologous copies of the same chromosome (as opposed to sister chromatids as is the case in conventional BFB). Such an SV can result in one of the two possible configurations. In the first configuration the derivative chromosome with the initial translocations has no centromeres, in which case its copy number should be wildly unstable. In the second the derivative chromosome would have two centromeres and essentially be the same structure as after a single cycle of BFB. In the latter case, we can use the same reasoning as for fold-back SVs above to justify why it is unlikely that such a fold-back SV-mediated dicentric structure is unlikely to be behind the local inverted two-jump patterns.

In theory the two inversion-type rearrangement could also form an extra-chromosomal ring of DNA. However, we believe this to be unlikely, as this ring will vast majority of the time have no centromeres and thus have a very unstable copy number, which is not something we observe, as in most cases the inverted copy number gain patterns have a clear integer copy number gain of 1-2 copies (Supplementary Figures 4, 39).

All local inversion copy number gain patterns can be explained as polymerase template switch events

We noticed that all the local inverted two-jump copy number gain patterns as well as the reciprocal inversion pattern can be explained by the same process. In this process, the polymerase makes two local template switches to the opposite strand. The first switch causes the polymerase to replicate DNA "backwards" and the second restores the original orientation of the polymerase. Depending on the relative positions of the template switch origin and target positions, the four different inversion patterns can be generated (Supplementary Figure 38). As none of the simpler rearrangement mechanisms can satisfactorily explain these observed patterns, we hypothesize that the inverted two-jump patterns, in particular those involving copy number gain are generated through this polymerase switching process.

Footprints with four breakpoints from different SVs

Among the most frequent footprint patterns involving four breakpoints were those involving four breakpoints from four different SVs. These footprints were consistent with different combinations of templated insertions and balanced breakpoints (Supplementary Figure 41). The relative CN pattern of the footprint with two templated insertions resembles that of a single templated insertion (Supplementary Figure 29), whereas the footprint involving two balanced breakpoints has the same relative CN pattern as a single balanced breakpoint. The footprint with a balanced breakpoint and a templated insertion has a combined relative copy number pattern of balanced breakpoints and templated insertions, and the nested templated insertion pattern has a relative CN similar to the duplication – inverted triplication – duplication footprint (Supplementary Figure 39).

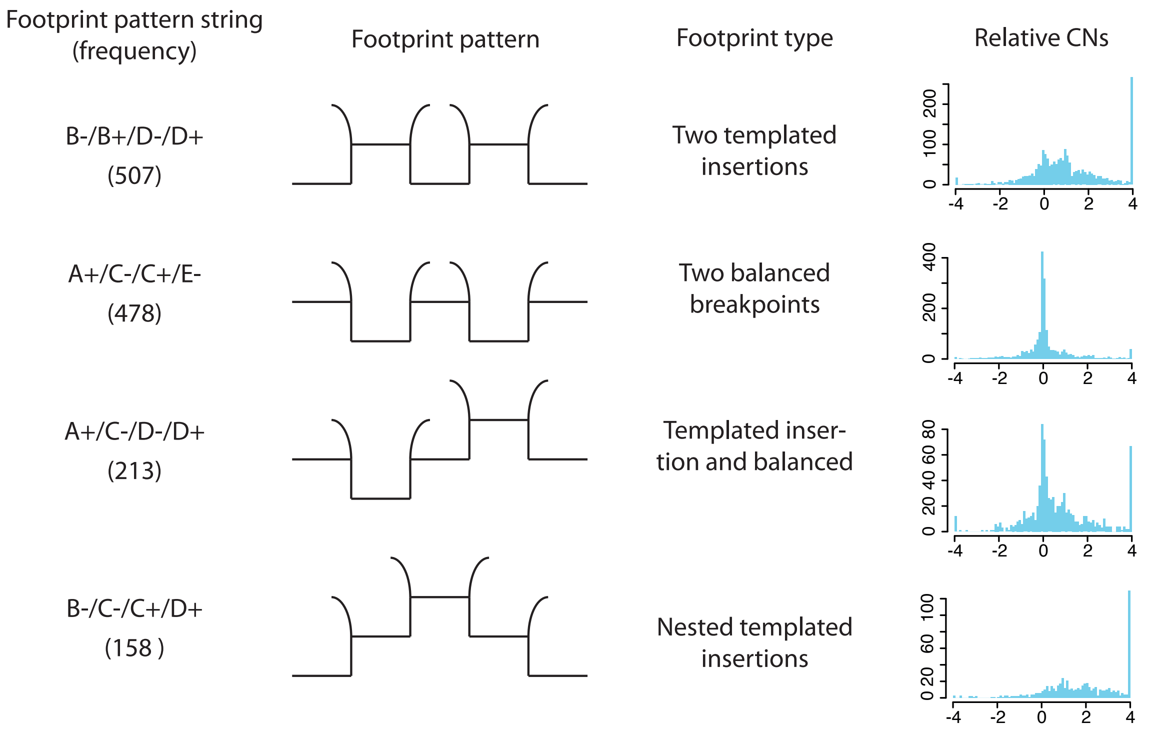

Supplementary Figure 41: Footprint involving four breakpoints from different SVs. The numbers in parentheses indicate the frequency of each respective footprint in the PCAWG cohort.

Footprints with breakpoints from three SVs

The third class of footprints with four breakpoints are those involving three SVs. There were many composite patterns, which could be explained as a simple deletion or tandem duplication, two of the most frequent SV types, being closely located with other independently occurring SV.

Interestingly, several patterns among the most frequent footprints involving three SVs closely resembled the local inverted two-jump patterns described above (Supplementary Figure 42). Each of them had one local inversion-type SV, and their relative CNs are very similar to their local two-jump counterparts (Supplementary Figure 38) as well as balanced breakpoints and templated insertions (Supplementary Figure 29), showing that these three-SV patterns are also copy number gain patterns with respect to the overall background chromosomal arm. Two of the patterns, A+^C+/C-/E- and A+^D+/C-/D- can only be unphased balanced breakpoints, one of which involving a local templated insertion. The other two patterns, B-^D-/B+/D+ and B-^C-/C+/D+, can be phased into a single structure resembling two linked and closely positioned templated insertions (Supplementary Figure 42).

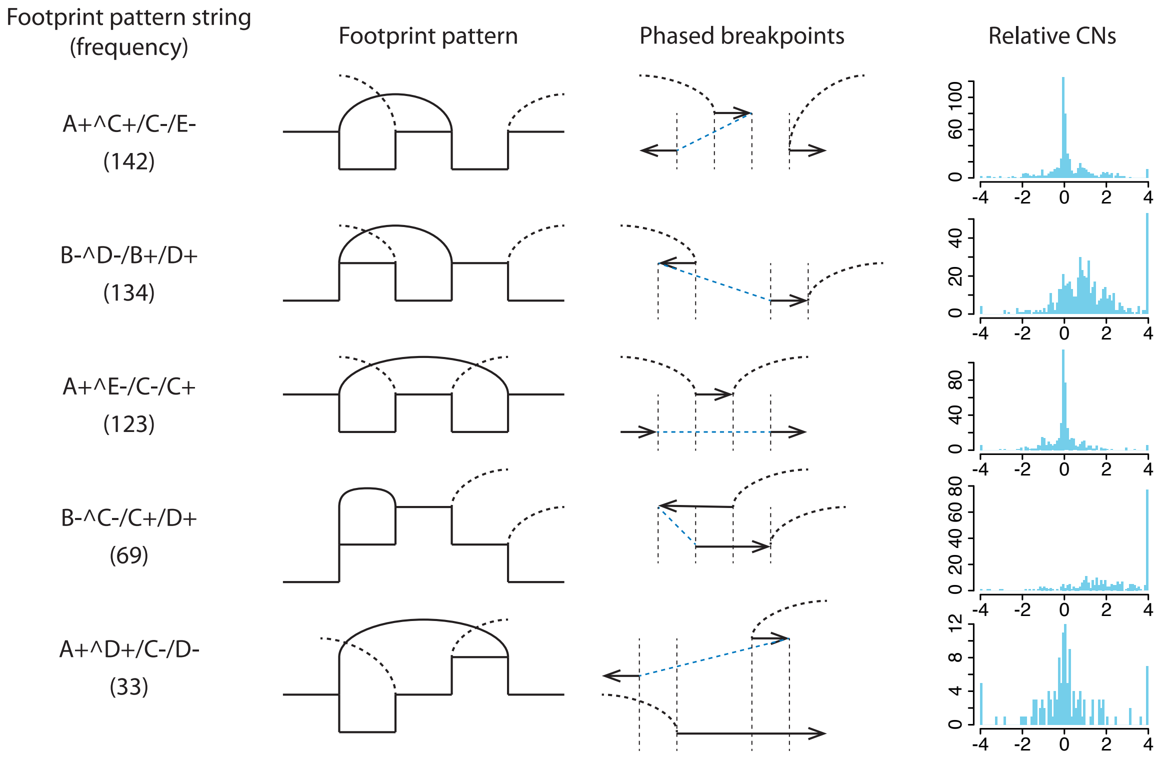

Supplementary Figure 42: Notable footprints involving four breakpoints from three different SVs. In A+^C+/C-/E- and A+^D+/C-/D-, the breakpoints cannot all be phased into a single derivative structure, but form obligate balanced breakpoints with a local templated insertion. In B-^D-/B+/D+ and B-^C-/C+/D+, the involved breakpoints can both be phased (shown in the "Phased breakpoints" column) and unphased (not shown).

In addition to relative copy number, these footprints involving a local inversion and two breakpoints shared other characteristics with balanced breakpoints and templated insertions. A substantial fraction of these footprints was connected to either balanced breakpoints or templated insertions through one or both of the outreaching breakpoints (Supplementary Figures 43-44). These rates exceeded the expected rates substantially. A randomly chosen SV in the PCAWG cohort has a chance of 4.1% and 6.6% of being involved in a balanced breakpoint or a templated insertion in at least one of its breakpoints, respectively (Supplementary Table 8). In contrast, the outreaching four-breakpoint footprints we are examining have a 19-46% chance of being connected to a balanced breakpoint or a templated insertion through their two outreaching breakpoints (with the exception of B-^C-/C+/D+ connected to balanced breakpoints). This rate is an order of magnitude higher than those for a randomly chosen SV, but in the same range as the rates for SVs with one of their ends already involved in a balanced breakpoint or a templated insertion (Supplementary Table 8). In other words, the outreaching footprints with four breakpoints are equally likely to be associated with balanced breakpoints and templated insertions as balanced breakpoints and templated insertions themselves, and this rate is an order of magnitude higher than the chance of a randomly chosen SV being associated with a balanced breakpoint or a templated insertion.

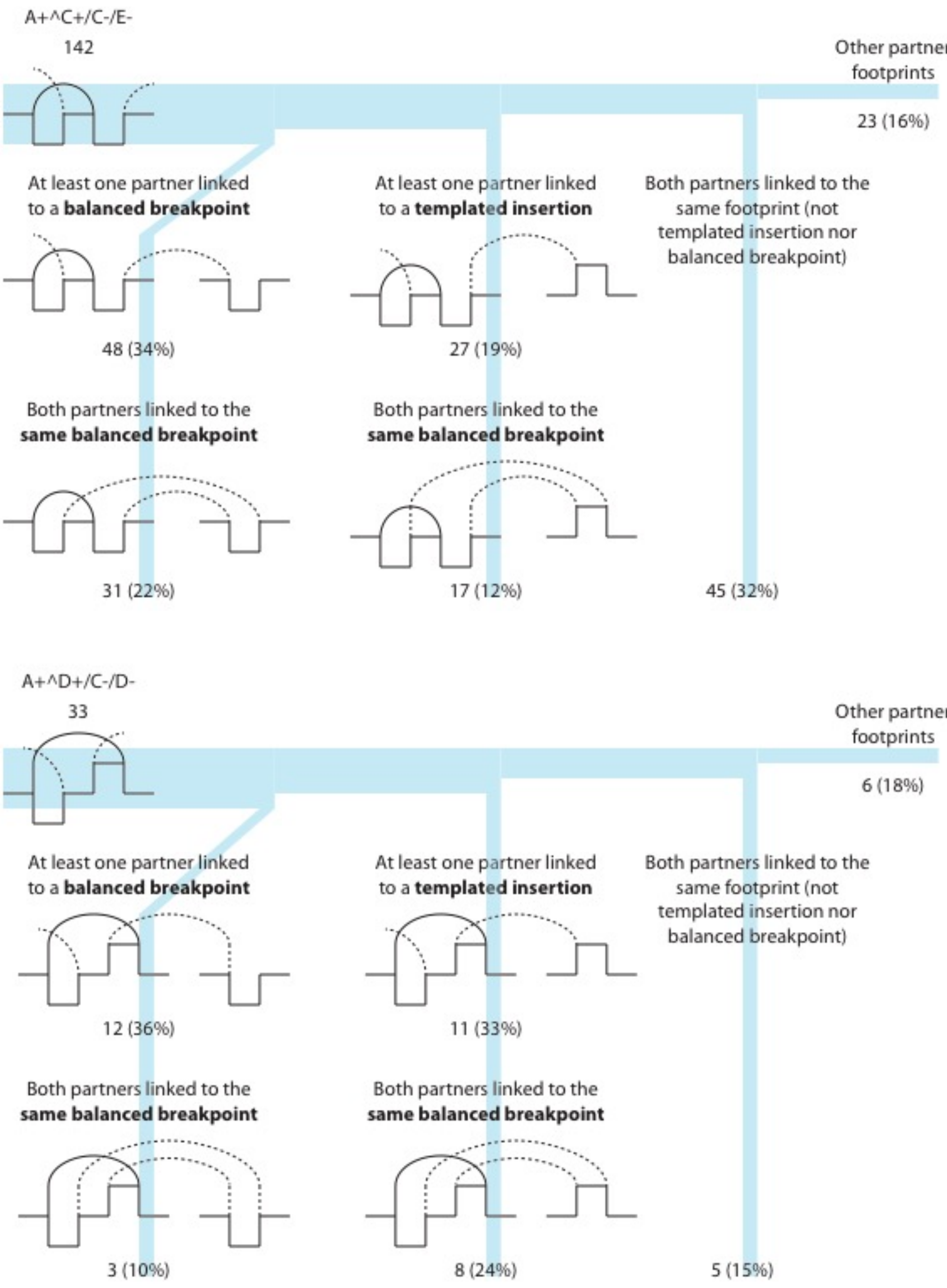

Supplementary Figure 43: Footprint types linked to the outreaching breakpoints of the A+^C+/C-/E- and A+^D+/C-/D- footprints. Numbers indicate each pattern's frequency in the PCAWG cohort and percentages are out of the total number of each respective footprint type.

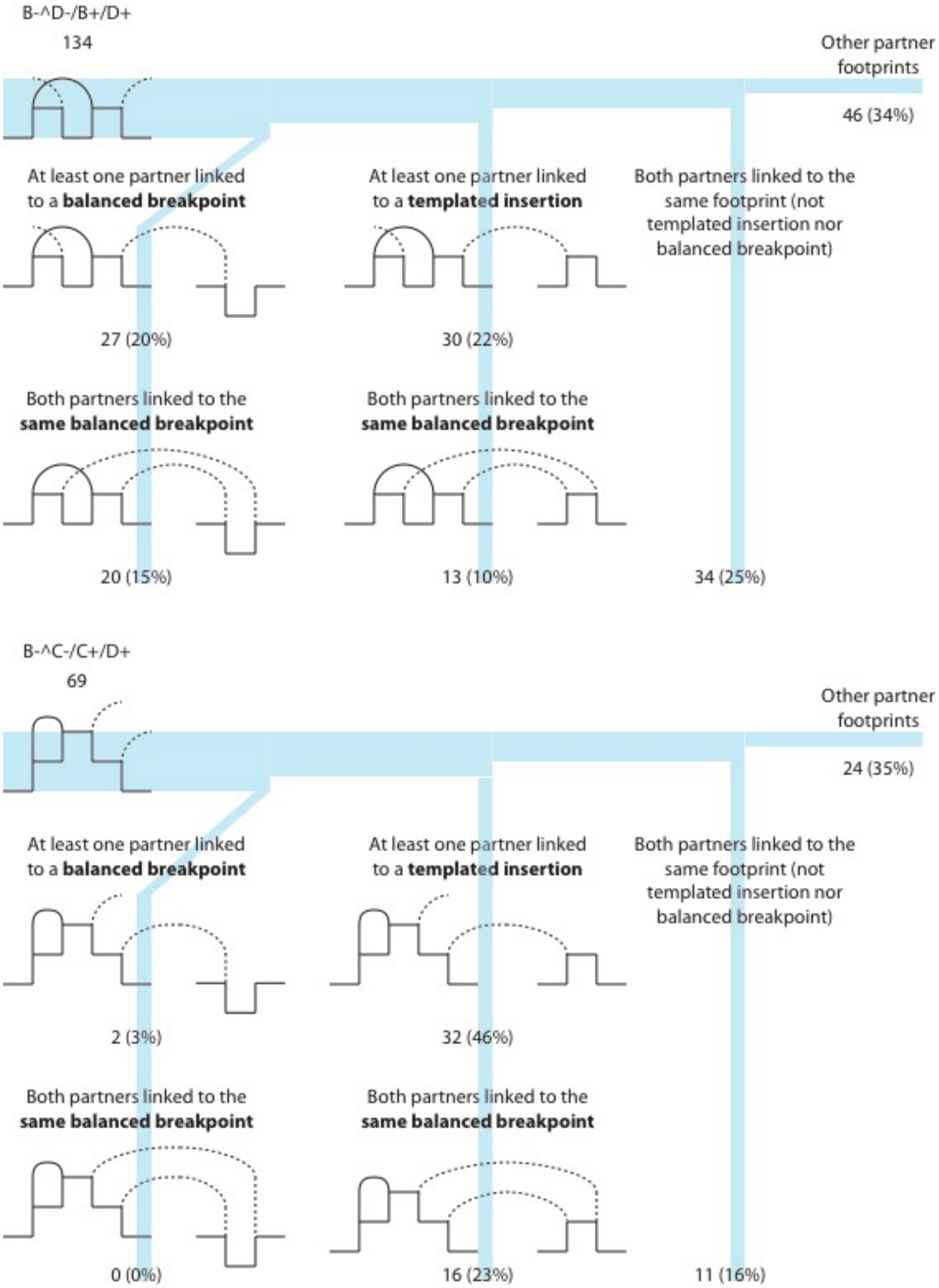

Supplementary Figure 44: Footprint types linked to the outreaching breakpoints of the B-^D-/B+/D+ and B-^C-/C+/D+ footprints. Numbers indicate each pattern's frequency in the PCAWG cohort and percentages are out of the total number of each respective footprint type.

Supplementary Table 8: Rates of being connected to balanced breakpoints of templated insertions for SVs and several footprints of interest. The description of the connection type is shown in the leftmost column, and its frequency in the dataset is shown in the rightmost column.

SV or footprint connection type	Symbol	Proportion
At least one breakpoint linked to a **balanced** breakpoint	*a*	4.1 %
Both breakpoints are a **balanced**	*b*	0.9 %
Second breakpoint is **balanced** given the first breakpoint is at **balanced**	*b / a*	22.6 %
At least one breakpoint linked to a **templated** **insertion**	*c*	6.6 %
Both breakpoints are at a **templated** **insertion**	*d*	1.6 %
Second breakpoint is on a **templated** **insertion** given the first breakpoint is on a **templated** **insertion**	*d / c*	24.3 %
One breakpoint is **balanced**, the other breakpoint is on a **templated insertion**	*e*	1.1 %
Second breakpoint is **balanced** given the first breakpoint is on a **templated insertion**	*e / c*	16.6 %
Second breakpoint is on a **templated insertion** given the first breakpoint is **balanced**	*e / a*	26.7 %
A+^C+/C-/E- connected to a **balanced** **breakpoint**		34 %
A+^C+/C-/E- connected to a **templated** **insertion**		19 %
A+^D+/C-/D- connected to a **balanced** **breakpoint**		36 %
A+^D+/C-/D- connected to a **templated** **insertion**		33 %
B-^D-/B+/D+ connected to a **balanced** **breakpoint**		20 %
B-^D-/B+/D+ connected to a **templated** **insertion**		22 %
B-^C-/C+/D+ connected to a **balanced** **breakpoint**		3 %
B-^C-/C+/D+ connected to a **templated** **insertion**		46 %

On the basis of the fact that these outreaching four-breakpoint footprints have both relative copy number patterns and rates of association with balanced breakpoints and templated insertions similar to balanced breakpoints and templated insertions, we suggest that these footprints are de facto balanced breakpoints and templated insertions, but with a local inverted polymerase switch event analogous to those in the local inverted two-jump events. If this model were true, the occurrence of these somewhat complex outreaching four-breakpoint footprints can be explained elegantly with simple events (Supplementary Table 9).

Supplementary Table 9: Simple rearrangement events explaining the connection of certain outreaching four-breakpoint footprints to balanced breakpoints or templated insertions. The footprint is shown on the left, and its interpretation on the right.

Footprint of interest	Connected to distal footprint	Rearrangement event
A+^C+/C-/E-, A+^D+/C-/D-	Balanced breakpoint	Reciprocal translocation, in which one of the balanced breakpoints has a local inverted polymerase switch event.
A+^C+/C-/E-, A+^D+/C-/D-	Templated insertion	Insertion of a templated sequence. Insertion point has an additional local inverted polymerase switch event.
B-^D-/B+/D+, B-^C-/C+/D+	Balanced breakpoint	Insertion of a templated sequence. There is an inverted polymerase switch event at the template donor locus.
B-^D-/B+/D+, B-^C-/C+/D+	Templated insertion	A chain or cycle of templated insertions, in which one of the template donor loci has an inverted polymerase switch event.

Footprints with three breakpoints

We now return to the study or footprints with three breakpoints. In the PCAWG cohort we found a total of 5,148 three-breakpoint footprints, which are tabulated by frequency and schematically illustrated in Supplementary Figure 45.

Many patterns are “composite events” involving unrelated SVs that got inadvertently clustered together due to their chance proximity. These footprints include an unbalanced translocation within a deleted region (A+^D-/B+) and a balanced translocation breakpoint near an unbalanced translocation (A+/B+/D-). However, the most commonly observed three-breakpoint footprints have interesting commonalities.

The two most common three-breakpoint footprints involve a templated insertion near an unbalanced translocation. In pattern ‘A+/C-/C+’, the inserted template is immediately after the breakpoint, while in ‘B-/B+/C+’ it is upstream of the unbalanced translocation.

Footprint pattern string (frequency)	Footprint pattern	Type	Relative CNs
A+/C-/C+ (1619)		Templated insertion near translocation	
B-/B+/C+ (950)		Templated insertion near translocation	
B-^C-/C+ (706)		Inversion near translocation	
A+^C+/C- (561)		Inversion near translocation	
A+/B+/D- (367)		Balanced breakpoint near translocation	
B-^C+/B+ (204)		Tandem duplication overlap-ping translocation	
A+^B+/D- (118)		Unphased inversion and translocation	
A+/B+/C+ (112)		Three unphased transloca-tions	
A+^D-/B+ (110)		Unphased translocation within deletion	
A+^B+/C+ (70)		Simple fold-back + simple unbalanced translocation	
A+/B+^C+ (59)		Simple fold-back + simple unbalanced translocation	
A+^C+/B+ (23)		Simple fold-back + simple unbalanced translocation	

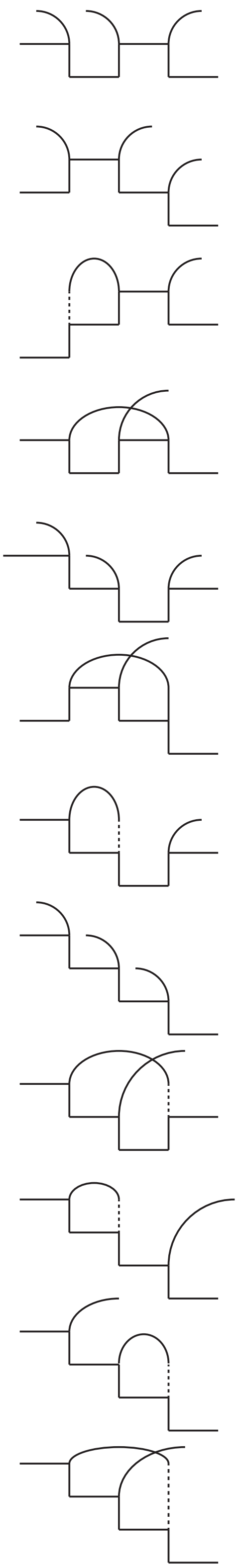

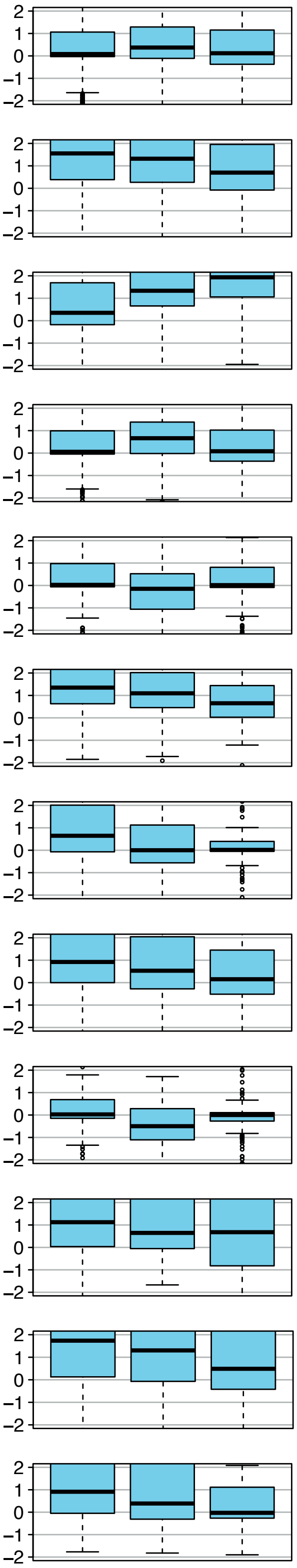

Supplementary Figure 45: Patterns and frequencies of footprints involving three breakpoints. Numbers below rearrangement pattern codes indicate the sample size (each SV pattern's frequency in the PCAWG cohort). For the box-and-whisker plots on the right, the box shows the median level of relative copy number (CN) as a thick black line, with the box's range denoting the interquartile range. The whiskers show the range of data or 1.5x the interquartile range, whichever is lesser. Outliers are shown as points outside the whiskers.

Interestingly, about 15% of the footprints involving an adjacent templated insertion (A+/C-/C+ and B-/B+/C+) are linked to these two footprint types themselves (Supplementary Tables 10-11). Strikingly, around 5% of these two footprint types are linked to the footprint of the same type in a reciprocal manner, such that the templated insertion on each footprint is linked to the outreaching breakpoint of the other footprint (Supplementary Figure 46). These frequencies are much higher than expected. Over the entire cohort, only 1.7% and 1.0% of the SVs in the cohort are connected to an A+/C-/C+ and a B-/B+/C+ footprint, respectively. Otherwise the outreaching breakpoint of these two footprint types frequently reach out to unbalanced translocations, templated insertions, and balanced breakpoints (Supplementary Tables 10-11).

Supplementary Table 10: The target footprint of the outreaching SV in the four most frequently encountered footprints with three breakpoints. Every column corresponds to one of the four footprints of interest and the outreaching breakpoint of each footprint is highlighted in bold.

	Outreaching footprint and breakpoint			
Reached footprint	**A+**/C−/C+	B−/B+/**C+**	B−^C−/**C+**	A+^C+/**C−**
A+/C−/C+	14.84%	5.83%	1.37%	1.32%
B−/B+/C+	3.78%	9.80%	1.22%	0.56%
B−^C−/C+	0.36%	0.13%	0.91%	1.32%
A+^C+/C−	0.15%	0.26%	1.06%	3.77%
Deletion	3.20%	2.12%	3.65%	12.81%
Tandem duplication	1.53%	7.42%	2.89%	5.08%
Inversion	2.69%	2.78%	6.53%	9.23%
Transloc	8.51%	10.60%	51.22%	38.04%
Templated insertion	10.91%	26.49%	10.49%	7.16%
Balanced breakpoint	7.71%	3.97%	2.28%	4.14%
other	46.33%	30.60%	18.39%	16.57%

Supplementary Table 11: Fold enrichments of the outreaching partner frequencies listed in Supplementary Table 10, normalised against the background rates of rearrangement breakpoint footprint types.

	Outreaching footprint and breakpoint			
Reached footprint	**A+**/C-/C+	B-/B+/**C+**	B-^C-/**C+**	A+^C+/**C-**
A+/C-/C+	16.8	6.6	1.5	1.5
B-/B+/C+	7.3	18.9	2.3	1.1
B-^C-/C+	0.9	0.3	2.4	3.4
A+^C+/C-	0.5	0.9	3.5	12.3
Deletion	0.2	0.1	0.2	0.6
Tandem duplication	0.1	0.4	0.2	0.3
Inversion	3.4	3.5	8.1	11.5
Transloc	3.3	4.1	19.6	14.6
Templated insertion	2.7	6.5	2.6	1.7
Balanced breakpoint	3.1	1.6	0.9	1.6
other	0.9	0.6	0.4	0.3

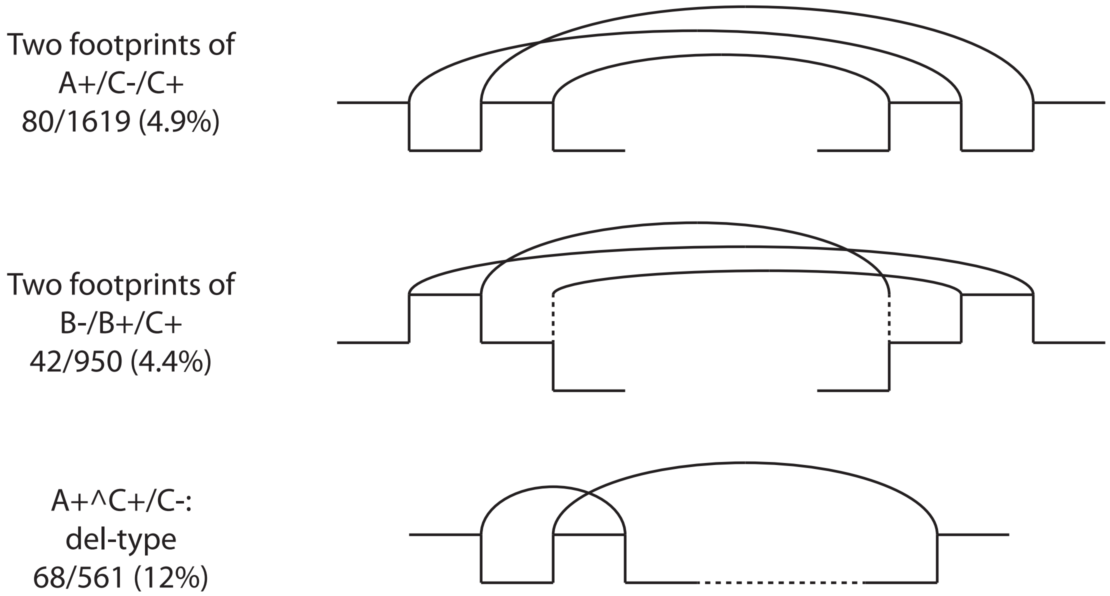

Supplementary Figure 46: Patterns and frequencies of three footprint-to-footprint partner types of interest. The copy number changes are shown as straight black lines, and the arcs denote breakpoint junctions explaining the copy number changes. The percentages are out of the total frequency of each respective footprint.

The third and fourth most frequent footprints with three breakpoints, B-^C-/C+ and A+^C+/C-, involve an inverted SV before an outreaching breakpoint. These footprints have a similar relative CN profile compared to local inverted two-jump patterns (Supplementary Figures 39, 45). For these two footprints, 80% of the footprints that the outreaching breakpoint links to are relatively simple. Roughly 60% of these target footprints are consistent with single-SV

rearrangements (deletion, tandem duplication, intra-chromosomal inversion or translocation. Another 10% of the target footprints are templated insertions or balanced breakpoints. Given the resemblance of these “inversion then outreaching breakpoint” footprints to local inverted two-jumps (Supplementary Figure 42), we suggest that these footprints in fact are analogous to local inverted two-jumps. Both types of footprints involve an initial inverted template switch, after which the second template switch event determines the resulting footprint pattern. If the second template switch is also a local inverted event, a local inverted two-jump pattern is produced (Supplementary Figure 38). If the second template switch event is outreaching, the one of the B-^C-/C+ or A+^C+/C- patterns (Supplementary Figure 45) are produced.

Analysis of footprint A+/C-/C+

Footprint A+/C-/C+ warrants extra attention. It is the most frequent footprint type with three breakpoints, and involves three breakpoints from three different SVs. On face value, the footprint is consistent with a single unbalanced translocation (A+) upstream of a templated insertion (C-/C+). On the other hand, one could also argue that this pattern was generated from a chromothripsis-like shattering event, where the fragment demarcated by the C-/C+ breakpoints is simply a shattered genomic fragment. We first took on to explore these two models.

Several features argue that the segment demarcated by C-/C+ is typically generated by a templated insertion. Firstly, across the cohort, the rate of A+/C-/C+ footprints correlates strongly with the rate of templated insertion footprints (Supplementary Figure 47, Spearman correlation coefficient = 0.52, $P < 2.2 \times 10^{16}$). Secondly, the size distribution of the segment demarcated by the C-/C+ breakpoints has two noticeable peaks at ~100bp and ~10kb, followed by the segment size tailing off (Supplementary Figure 47). This size distribution pattern is remarkably similar to that observed in simple templated insertion footprints (Supplementary Figure 37). Strikingly, the joint distribution between the two distances in the footprint is remarkably similar to the equivalent distances in the A+^C+/C- footprint (Supplementary Figure 47). The latter footprint has a local inverted templated insertion linked to a translocation. This observation is consistent with the fact that the A+/C-/C+ footprint is analogous to the A+^C+/C- footprint with the difference that the templated insertion comes from a distant site as opposed to locally.

Lastly, we do not believe that most A+/C-/C+ footprints are caused by inadvertent clustering of a templated insertion together with an unrelated, independent SV breakpoint. The partner footprints of the outreaching A+ breakpoint have a very particular distribution, being fivefold depleted in SVs consistent with a simple deletion, but enriched with other structure involving templated insertions, balanced breakpoints and translocations (Supplementary Table 11). If the clustering was accidental, we would expect the outreaching breakpoint to have a footprint distribution closer to the respective background rates of footprints.

A significant fraction of the breakpoints between the A+ and C- breakpoints are ≤100bp (Supplementary Figure 47) and are essential balanced breakpoints. This raises the interesting possibility that in many A+/C-/C+ footprints, the strand invasion that generates a templated insertion also causes an adjacent unbalanced translocation.

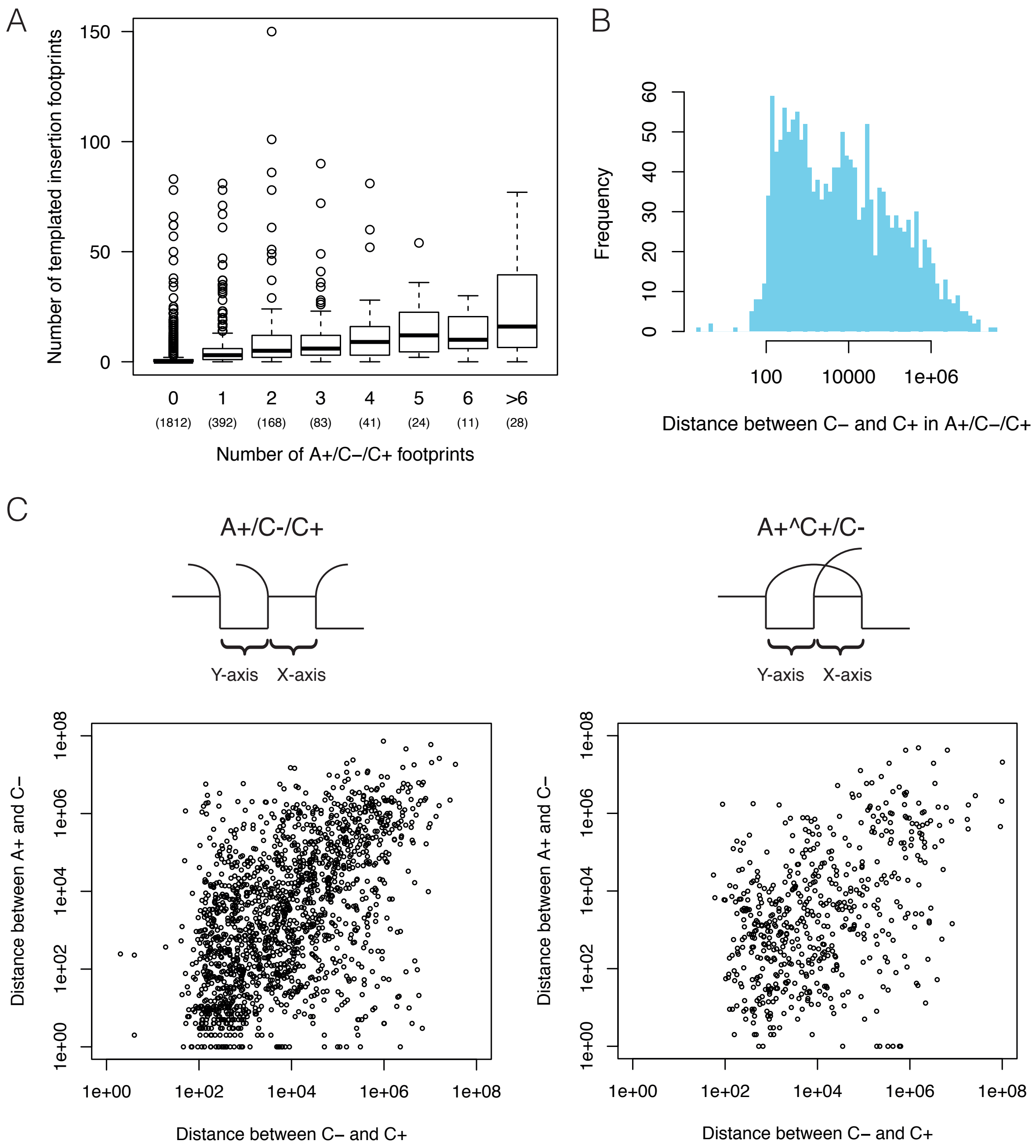

Supplementary Figure 47: (A) Correlation between the per-sample rate of A+/C-/C+ footprints and templated insertion footprints. For the box-and-whisker plots, the box shows the median number of templated insertions as a thick black line, with the box's range denoting the interquartile range. The whiskers show the range of data or 1.5x the interquartile range, whichever is lesser. Outliers are shown as points outside the whiskers. The x axis is distributed by the number of A+/C-/C+ footprints, with the sample size shown in brackets under the label denoting the number of patients in that category. (B) Distance distribution between the C- and C+ breakpoints in A+/C-/C+. (C) Distance distributions between the three breakpoints in A+/C-/C+ and A+^C+/C- footprints.

Five breakpoint and six breakpoint footprints

The more complex the footprint patterns become, the more distinct patterns there can combinatorically be, and the less recurrent they become. We found a total of 1,937 footprints with five breakpoints, of which only one footprint type with five breakpoints has a recurrence

above 100 in the dataset (Supplementary Figure 48). All these footprints appear to be a single translocation breakpoint clustered with adjacent templated insertions or balanced breakpoints.

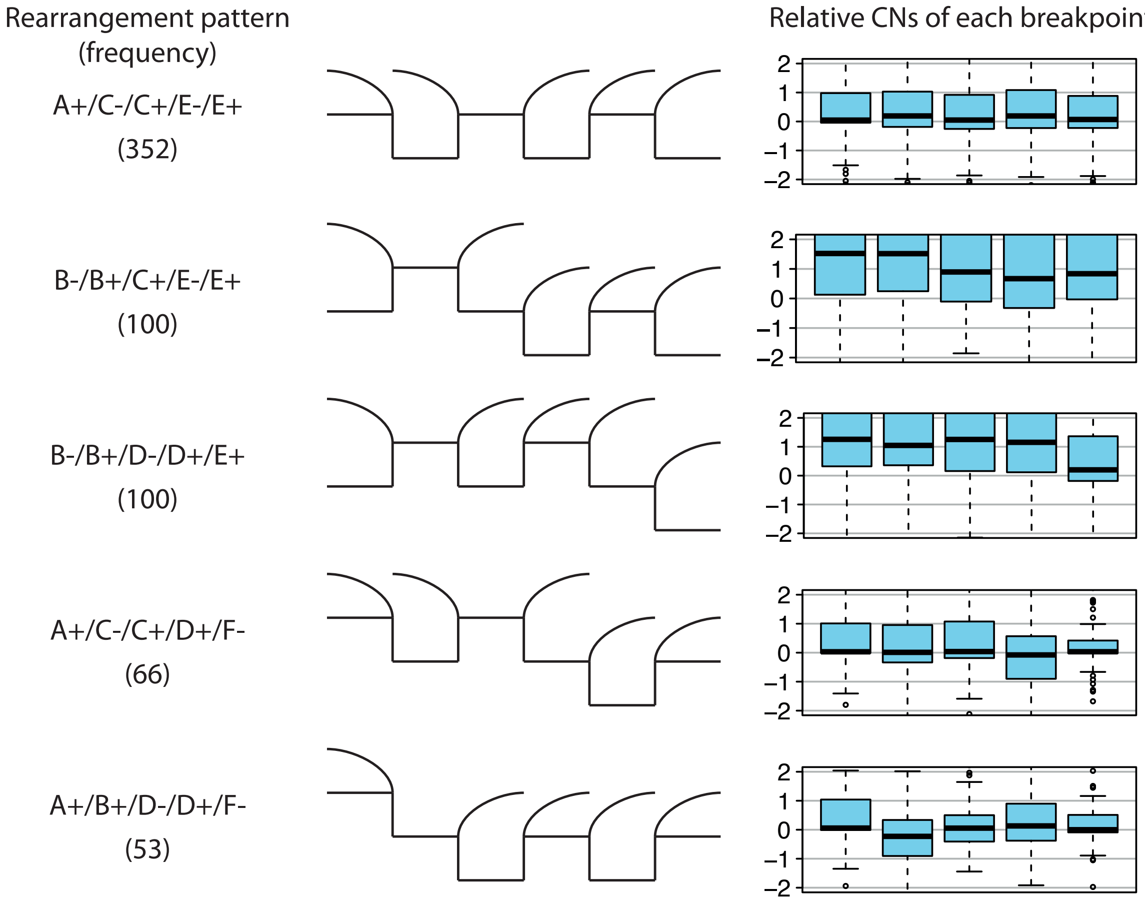

Supplementary Figure 48: Footprint patterns and relative CNs of footprints with breakpoints. In the relative CN plot, boxes one to five correspond to breakpoints one to five in the schematic representations in the middle. For the box-and-whisker plots, the box shows the relative copy numbers as a thick black line, with the box's range denoting the interquartile range. The whiskers show the range of data or 1.5x the interquartile range, whichever is lesser. Outliers are shown as points outside the whiskers. The sample size shown in brackets under each category on the left represents the numbers of instances of each footprint in the dataset.

Given the high frequency of local two-jump events, we also wondered whether there would be local three-jump events, i.e. footprints formed entirely of three local SVs. We found a total of 1,037 such footprints, of which only one had a recurrence above 50 in the cohort (Supplementary Figure 49). Both the footprint patterns and the relative copy number analysis suggest that three of the patterns are essentially local reciprocal inversions but with an additional template switch event. Two patterns are consistent with the interlocked inverted duplication pattern, except that there are three local inserted templates strung together. In addition, one footprint seems to be three rounds of BFB, as suggested by phasable fold-back-type SVs and significantly increased relative copy number (Supplementary Figure 49). Therefore, footprints involving more than two local SVs appear to merely be more complex versions of footprints involving two local SVs.

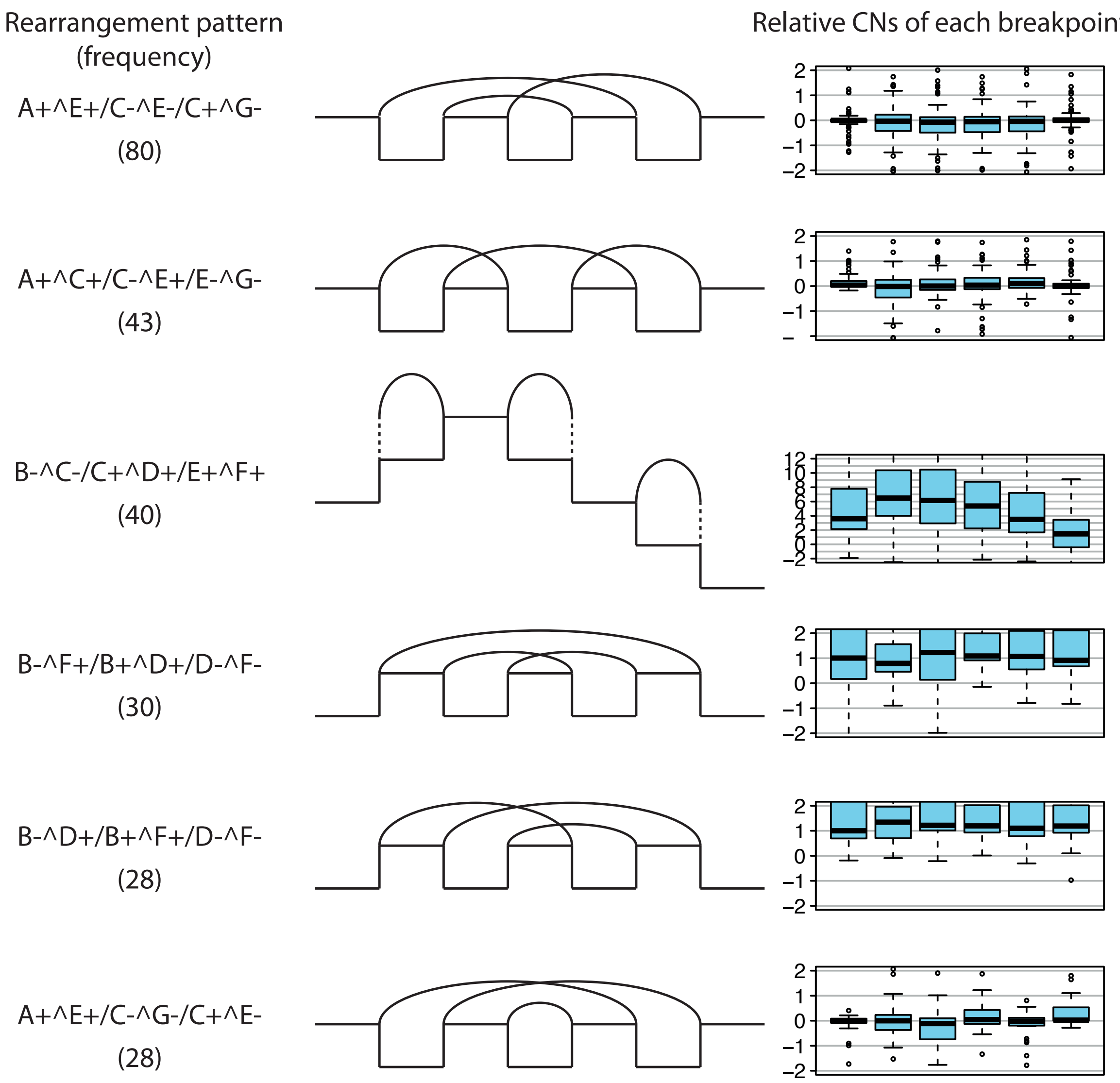

Supplementary Figure 49: Six most frequent footprints involving three local SVs. For the box-and-whisker plots, the box shows the relative copy numbers as a thick black line, with the box's range denoting the interquartile range. The whiskers show the range of data or 1.5x the interquartile range, whichever is lesser. Outliers are shown as points outside the whiskers. The sample size shown in brackets under each category on the left represents the numbers of instances of each footprint in the dataset.

Analysis of somatic rearrangement mutational signatures

Above we have described a collection of rearrangement patterns with up to six footprints. While some of the footprints at self-contained rearrangement events (e.g. reciprocal inversions), others have outreaching SVs. Therefore, a rearrangement event itself may involve many different footprint types. Classifying rearrangement clusters on the basis of its constituent footprint therefore gets complex combinatorically. To simplify the SV signatures analysis, we broke SV clusters down into footprints and decomposed the footprints using NMF instead. There is some biological rationale to this approach too.

We fitted a nonnegative matrix factorization model on the matrix of per-sample footprint counts. We used Kullback-Leibler divergence as the cost function for fitting the model, which

corresponds to assuming that each footprint count is generated from a Poisson distribution[6]. As an additional benefit, when the footprint counts are modelled as generated from Poisson, each fitted NMF model will provide an overall model likelihood for the observed data, which makes many classical model selection approaches possible.

We fitted NMF on the footprint counts data with ranks ranging from 1 to 20. The optimal model based on AIC was given by nine signatures, although the AICs for eight and ten signatures were very similar (Supplementary Figure 50). Below we discuss the footprints generated by each signature.

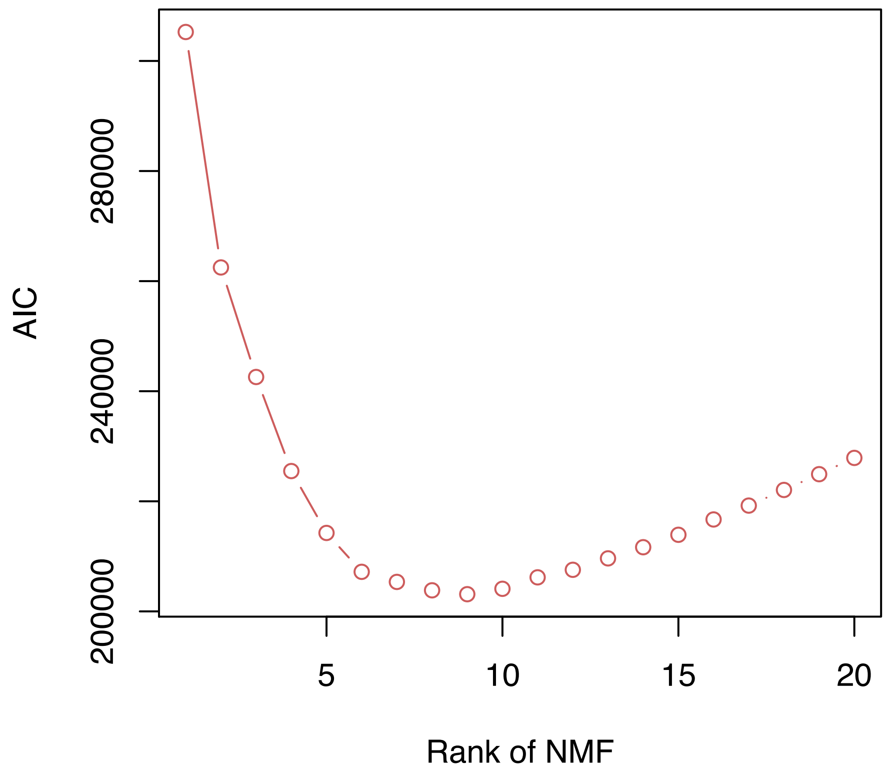

Supplementary Figure 50: AIC for NMF models with ranks ranging from 1 to 20. The x axis shows the number of different signatures fitted and the y axis shows the estimated Akaike Information Criterion (AIC) of the resulting NMF fit.

The small deletions signature generates all deletions 10kb, as well as small reciprocal inversions and small local inverted duplications (Supplementary Figure 38). This signature seems to be consistent with a template switch ahead of replication blockage. Most of the time the template switch event is codirectional, leaving a small deletion. Occasionally, the template switch is in inverted orientation, leading to a balanced inversion or a local inverted duplication. After the first local inversion junction, the second template switch can also be outreaching. When this happens, a Trans + Ins (A+^C+/C-) is generated. This signature resembles the recently described small deletions signature, which was reported to be associated with *BRCA1* and *BRCA2* inactivation[10].

Another deletion signature generates all medium-size deletions (10kb-3Mb), more than half of reciprocal inversions >100kb and 25% of local inverted duplications >100kb. This signature is consistent with the same model as the previous signature, except that the initial template switch happens at a further distance from the initial breakpoint. This resembles a previously described signature associated with large deletions[10].

The third signature generates all small tandem duplications <55kb, as well as templated insertions that are part of templated insertion cycles. Interestingly, this signature is not the main generator of templated insertions that are in chains. This signature also generates a small number of interlocked inverted duplication events. The possible molecular model for this signature, as opposed to the deletion signatures, is that a template switch goes behind the initial replication blockage. When the template switch results in the polymerase proceeding in inverted orientation, an interlocked inverted duplication is generated. It is surprising that this signature does not appear to generate many duplication - inv triplication – duplication events. If the first breakpoint is outreaching and produces a templated insertion before returning to the original template, then a templated insertion cycle is produced, with the actual tandem duplication manifesting as a templated insertion. Finally, if the first template switch is backwards in inverted orientation but the second breakpoint is outreaching, then a translocation with a fold-back (B-^C-/C+) is produced. This signature is reminiscent of the small tandem duplication signature described recently[10].

The fourth signature is very similar to the previous one, generating most medium-size TDs (55kb-10Mb), as well as templated insertion cycles where the templates are larger than 100kb. Again, templated insertions as part of chains does not appear to be generated by this signature. Around half of all interlocked inverted duplication events are generated by this signature, as well as some duplication - inverted triplication – duplication events. A composite footprint of a tandem duplication partially overlapping with a templated insertion (B−^C+/C−/D+) is also most frequently generated by this signature.

Templated insertions as part of templated insertion chains are most commonly generated by the fifth signature. Interestingly, virtually none of the templated insertion footprints as part of cycles are generated by this signature. In contrast, this signature suggests frequent polymerase template switches during DNA repair. Balanced breakpoints are also often generated by this signature, as they form the genomic insertion points for chains of templated insertions. Curiously, this signature also yields unbalanced translocations and fold-back inversion-type SVs as part of more complex SV clusters (as opposed to in isolation). The two most recurrent three-breakpoint footprints, are generated by this signature, supporting the model that these footprints indeed involve templated insertions as opposed to chromothripsis-type genomic shattering (Supplementary Figure 45). As suggested above, certain four breakpoint footprints can be generated by templated insertions forming a local inverted template switch event (Supplementary Figure 42). Indeed, we find these footprints (B−^C−/C+/D+ and B−^D−/D+/D+) generated by this signature. Finally, this signature is associated with many footprints that are consistent with multiple templated insertions or a templated insertion clustered with other breakpoints.

The sixth signature involves long distance intra-chromosomal fusions, such as large deletion, TD and unbalanced inversion-type SVs. More than half of large local inverted duplication and duplication - inverted triplication - duplication events are generated by this signature. The local inversion then outreaching SV-type footprints (Supplementary Figure 45) are mostly generated by this signature, as are fold-back inversions presumably derived from BFB. Most

complex footprints generated by this signature appear to be composite events of a fold-back inversion clustered together with a simple SV such as a deletion or a tandem duplication.

Interestingly, signature six has many features of long-distance intra-chromosomal fusions, but simple nonreciprocal inter-chromosomal translocations are generated from a separate, highly specific signature that barely generates any other SV types.

The eight signature generates all balanced rearrangements, whether in balanced breakpoint cycles or in chains, with the exception of the balanced breakpoints generated as part of templated insertions (signature five). This includes all conventional reciprocal translocations, as well as more complex chains or cycles of balanced breakpoints (i.e. chromoplexy). The balanced breakpoint pattern with a local inverted templated insertion (Supplementary Figure 42) was also mostly generated by this signature, as expected.

The final signature is the fragile site signature. This signature generates all deletions located within fragile sites, as well as about 75% of tandem duplications within fragile sites. It is interesting that apart from deletions, fragile sites are also prone to generating tandem duplications.

Footprint connectivity analysis

That footprints are not generated independently but are linked together through SVs allows the study of how they are connected to form complex rearrangements. We collected all footprints used in the signatures analysis that contain at least one outreaching SV as well as all complex footprints. For each resulting "anchor" footprint type, we computed the rate at which they were connected to different "target" footprint types through an outreaching SV (Supplementary Tables 12-13).

Several interesting observations can be made from this analysis. The footprints can be divided roughly into five different clusters. The first cluster of footprints four-fold increased rate to be connected to simple unbalanced translocation breakpoints (Supplementary Table 13). These footprints include the unbalanced translocation breakpoint itself, composite erroneously clustered footprints (unbalanced breakpoint clustered with a deletion, tandem duplication or a local inversion) as well as footprints consistent with a local template switch followed by an outreaching unbalanced breakpoint (B-^C-/C+ and A+^C+/C-). This group of footprints are rarely linked to complex footprints of six of more breakpoints.

The second group of footprints are preferentially linked to balanced breakpoints or templated insertions. These footprints include the balanced breakpoint and templated insertion breakpoints themselves. The footprints within this group seem fall into a gradient of connectivity from balanced breakpoints to templated insertions. The balanced breakpoint, balanced breakpoint with a local switch (A+^C+/C-/E-) and two local balanced breakpoint (A+,E-/C-/C+) are often linked to balanced breakpoints. On the other hand, inserted templates with a local inverted template switch (B-^D-/B+/D+ and B-^C-/C+/D+, see Supplementary Figure 42) have a stronger tendency to be linked to templated insertions, similar with composite footprints of templated insertions clustered with tandem duplications (B-^C+/C-/D+ and B-^D+/C-/C+). This group of balanced breakpoint and templated insertion-linked footprints have a slightly elevated albeit still low rate of being connected to complex footprints with ≥6 breakpoints.

The third group of footprints comprises complex footprints of 1-5 breakpoints. In this group, none of the footprints with a total frequency of ≥50 in the PCAWG cohort (i.e. those not labelled "complex (1-5)") had a local SV, but all breakpoints were outreaching, with their partner footprints in another footprint. This is contrary to the first and second group of footprints, most of which consist of at least one local SV. This group is less connected to simple unbalanced translocations than expected and clearly more connected to footprints in the same group than by chance (Supplementary Table 13).

The fourth group of footprints are complex ones involving six or more breakpoints. Interestingly, there is a correlation even within this group, and these complex footprints tend to be connected to other complex footprints of similar number of breakpoints. For example, footprints with more than 50 breakpoints are connected to footprints with 6-10 breakpoints only 40% of the expected rate, but have a 3.9-fold rate of being connected to another footprint of >50 footprints.

There is a curious tendency for like to like footprint connectivity. For example, although only six percent of B-^C+/C-/D+ footprints are linked to a footprint of the same type through an SV, this represents a 76-fold enrichment due to the rarity of B-^C+/C-/D+ footprints. Similarly, footprint types A+/C-/C+/D+/F-, A+/B+/D-/D+ and A+/B+/C+ are 45, 38 and 25-fold more likely to be connected to like footprint types than by chance (Supplementary Table 13).

Supplementary Table 12: A connectivity map of footprints. Every row corresponds to an anchor footprint type, and every column indicates the percentage that the anchor footprint is linked to a target footprint. Entries in each cell are percentage values. For example, 65% of the outreaching SVs in B-^C-/C+ type footprints are linked to single breakpoints, but only 2% of single breakpoints are linked to B-^C-/C+ type footprints. The set of footprint types considered for anchors and targets was the same, but only target footprints that are linked to at >5% frequency by at least one anchor footprint are shown. Footprints labelled as "complex" are further divided based on the number of clustered breakpoints, indicated in parentheses.

Footprint type	single bkpt	B-^C-/C+	bal bkpt	templated ins	A+/B+	B-^C+/C-/D+	A+/C-/C+	B-/B+/C+	A+/B+/C+	A+/B+/D-	B-/C-/C+/D+	A+/C-/C+/E-	A+/B+/D-/D+	A+/C-/C+/D+/F-	A+/C-/C+/E-/E+	complex (1-5)	complex (6-10)	complex (11-20)	complex (20+)	complex (50+)
A+^D-/B+	65	0	4	7	2	0	4	0	0	0	0	0	0	0	0	9	3	1	0	1
B-^C+/B+	68	1	1	8	4	0	1	1	0	0	1	1	1	0	0	4	2	2	1	1
B-^C-/C+	64	1	2	10	1	0	1	1	0	0	0	0	0	0	0	7	2	3	2	1
A+^C+/C-	66	1	4	7	1	0	1	1	0	1	0	1	0	0	0	5	2	2	1	1
single bkpt	60	2	4	6	2	0	2	2	0	0	0	0	0	0	0	6	4	3	2	1
A+/B+^C+	60	6	2	4	4	0	2	4	0	0	0	0	0	0	0	8	4	4	0	0
A+^B+/C+	64	3	8	5	3	0	0	0	0	0	0	2	0	0	0	9	2	3	0	2
A+^B+/D-	63	1	4	4	2	0	0	1	0	0	0	1	0	1	0	13	2	2	2	0
A+^E-/C-/C+	6	0	49	9	3	0	0	0	1	1	0	3	0	1	1	13	4	2	2	0
A+^C+/C-/E-	14	0	32	15	3	0	5	2	0	1	0	3	0	0	0	11	2	3	0	0
bal bkpt	7	0	39	22	2	0	2	1	0	1	0	2	0	1	0	8	5	3	1	1
B-^D+/B+/D-	6	0	30	35	1	0	1	2	0	0	1	0	0	0	0	13	5	2	1	2
B-^D-/B+/D+	16	1	20	18	5	0	3	0	0	0	0	1	0	0	0	11	5	6	2	3
A+/B+	14	0	11	26	10	0	4	2	0	1	0	0	0	1	0	10	5	5	3	1
B-/B+^D-/D+	15	0	16	28	7	0	3	1	2	0	0	0	0	1	0	12	4	7	1	0
B-^C-/C+/D+	21	3	2	44	3	0	2	1	0	0	0	0	0	0	0	4	3	7	6	1
templated ins	8	0	16	40	4	0	2	3	0	0	1	0	0	0	0	7	4	3	3	2
B-^C+/C-/D+	13	1	7	43	1	6	2	5	1	0	1	0	0	0	0	9	1	4	1	1
B-^D+/C-/C+	7	0	4	56	1	1	2	9	0	0	3	0	0	0	0	6	1	4	1	1
A+/C-/C+	12	0	7	8	3	0	14	3	1	2	0	2	1	3	0	13	13	8	4	2
A+/B+/D-	12	0	9	7	4	0	8	6	1	6	0	2	0	1	1	15	15	5	3	1
B-/B+/C+	14	0	4	19	2	0	5	8	0	2	1	1	0	0	0	11	8	9	5	4
A+/B+/C+	13	0	2	14	3	0	8	3	6	4	2	0	1	1	0	13	8	8	7	4
A+/C-/D-/D+	9	0	10	11	2	0	4	2	0	1	0	3	1	1	0	15	12	10	4	3
A+/C-/C+/E-	6	0	13	2	1	0	5	1	0	1	0	10	1	3	1	17	15	10	5	2
B-/B+/D-/D+	5	0	5	12	2	0	5	2	0	0	1	2	0	1	0	13	14	13	8	7
B-/B+/C+/D+	12	1	2	12	5	0	3	3	0	1	0	2	1	1	0	15	11	7	8	9
A+/C-/C+/D+	7	0	5	6	2	0	4	2	0	1	0	2	1	1	0	17	19	10	7	3
B-/C-/C+/D+	6	0	4	23	2	0	1	5	1	0	5	0	0	1	0	11	9	12	7	6
A+/B+/D-/D+	7	0	2	7	2	0	5	1	0	0	0	4	7	3	0	12	13	15	8	4
A+/B+/D-/D+/F-	4	0	10	3	1	0	6	1	0	5	0	2	0	4	2	15	20	12	9	6
A+/C-/C+/E-/E+	5	0	5	4	1	0	7	0	0	0	0	3	1	6	0	14	20	17	8	4
A+/C-/C+/D+/F-	5	0	8	3	1	0	5	1	0	3	0	6	0	1	7	16	18	14	6	0
B-/B+/D-/D+/E+	6	0	3	9	4	0	4	5	0	0	0	0	1	1	0	10	17	16	13	4
B-/B+/C+/E-/E+	7	0	5	6	1	0	2	2	0	0	2	2	0	2	0	10	15	17	15	8
complex (1-5)	11	0	7	9	2	0	4	2	0	1	0	2	0	2	0	17	15	10	6	3
complex (6-10)	4	0	3	4	1	0	3	1	0	1	0	1	0	2	0	11	26	22	11	6
complex (11-20)	3	0	2	2	1	0	1	1	0	0	0	1	0	1	0	6	18	30	21	10
complex (20-50)	2	0	1	2	0	0	1	0	0	0	0	0	0	1	0	3	8	20	35	24
complex (51+)	1	0	0	2	0	0	0	0	0	0	0	0	0	0	0	2	4	10	25	52

Supplementary Table 13: The observed/expected connectivity rates, i.e. the rates in Supplementary Table 12 normalised by the relative frequency of each target footprint type.

Footprint type	single bkpt	B-^C-/C+	bal bkpt	templated ins	A+/B+	B-^C+/C-/D+	A+/C-/C+	B-/B+/C+	A+/B+/C+	A+/B+/D-	B-/C-/C+/D+	A+/C-/C+/E-	A+/B+/D-/D+	A+/C-/C+/D+/F-	A+/C-/C+/E-/E+	complex (1-5)	complex (6-10)	complex (11-20)	complex (20+)	complex (50+)
A+^D-/B+	4.8	0.0	0.6	0.7	1.2	0.0	1.7	0.0	0.0	0.0	0.0	0.0	0.0	0.0	0.0	1.2	0.3	0.1	0.0	0.1
B-^C+/B+	5.0	1.5	0.1	0.7	2.3	0.0	0.4	0.4	0.0	0.0	1.6	0.6	1.9	0.0	0.0	0.6	0.2	0.1	0.0	0.1
B-^C-/C+	4.7	2.6	0.3	0.9	0.8	2.0	0.6	0.9	0.9	0.9	0.5	0.2	0.6	0.0	0.0	0.9	0.2	0.2	0.1	0.0
A+^C+/C-	4.9	3.7	0.6	0.7	0.8	0.0	0.5	0.4	0.0	1.4	0.6	0.6	0.7	0.2	0.0	0.6	0.2	0.2	0.1	0.1
single bkpt	4.5	4.7	0.5	0.6	1.0	1.0	0.9	1.0	1.0	0.9	0.5	0.4	0.5	0.4	0.4	0.8	0.3	0.2	0.1	0.1
A+/B+^C+	4.4	15.8	0.3	0.4	2.3	0.0	0.8	2.7	0.0	0.0	0.0	0.0	0.0	0.0	0.0	1.0	0.4	0.3	0.0	0.0
A+^B+/C+	4.7	8.6	1.1	0.5	1.9	0.0	0.0	0.0	0.0	0.0	0.0	1.6	0.0	0.0	0.0	1.2	0.1	0.2	0.0	0.1
A+^B+/D-	4.7	2.7	0.5	0.4	1.2	0.0	0.0	0.7	0.0	0.0	0.0	1.0	0.0	1.1	0.0	1.6	0.2	0.1	0.1	0.0
A+^E-/C-/C+	0.5	1.2	6.6	0.8	1.6	0.0	0.2	0.0	5.2	2.4	0.0	3.6	0.0	0.9	5.2	1.6	0.4	0.2	0.1	0.0
A+^C+/C-/E-	1.1	0.0	4.3	1.5	1.6	0.0	2.0	1.3	0.0	2.1	0.0	2.7	1.4	0.4	0.0	1.4	0.2	0.2	0.0	0.0
bal bkpt	0.5	0.3	5.4	2.2	1.5	1.0	0.9	0.5	0.2	1.2	0.5	1.8	0.3	0.6	1.2	1.0	0.4	0.2	0.1	0.0
B-^D+/B+/D-	0.4	0.0	4.1	3.5	0.5	0.0	0.3	1.6	0.0	0.0	2.4	0.0	0.0	0.0	0.0	1.6	0.4	0.1	0.1	0.1
B-^D-/B+/D+	1.2	3.4	2.7	1.8	2.7	0.0	1.2	0.3	0.0	0.0	0.0	0.9	0.0	0.0	0.0	1.4	0.5	0.5	0.2	0.2
A+/B+	1.0	0.8	1.5	2.5	6.2	0.9	1.7	1.3	1.8	2.7	1.1	0.4	1.5	0.7	0.4	1.3	0.5	0.4	0.2	0.1
B-/B+^D-/D+	1.1	0.0	2.1	2.7	3.9	0.0	1.3	0.6	9.9	0.0	0.0	0.0	0.0	0.9	0.0	1.6	0.4	0.5	0.1	0.0
B-^C-/C+/D+	1.5	6.8	0.2	4.3	2.0	0.0	0.7	0.6	0.0	0.0	0.0	0.0	0.0	0.0	0.0	0.5	0.2	0.5	0.4	0.1
templated ins	0.6	0.9	2.2	4.0	2.5	4.2	0.8	1.8	1.4	0.7	2.3	0.2	0.6	0.3	0.3	0.9	0.4	0.2	0.2	0.2
B-^C+/C-/D+	1.0	2.0	1.0	4.2	0.9	74.9	0.9	3.5	4.4	0.0	2.2	0.0	0.0	0.0	0.0	1.2	0.1	0.3	0.1	0.1
B-^D+/C-/C+	0.5	0.0	0.6	5.5	0.4	17.3	0.8	6.5	0.0	0.0	8.2	0.0	0.0	0.0	0.0	0.8	0.1	0.3	0.0	0.1
A+/C-/C+	0.9	0.6	0.9	0.8	1.7	0.9	5.5	2.2	3.0	3.1	0.4	2.1	2.1	2.8	2.1	1.6	1.2	0.6	0.3	0.2
A+/B+/D-	0.9	0.9	1.2	0.7	2.7	0.0	3.1	4.1	7.0	11.0	0.0	2.3	0.8	0.7	5.0	1.9	1.4	0.4	0.2	0.1
B-/B+/C+	1.0	0.9	0.5	1.8	1.3	3.5	2.2	5.4	2.1	4.1	3.7	0.6	0.7	0.3	0.9	1.4	0.8	0.6	0.3	0.3
A+/B+/C+	1.0	0.9	0.2	1.4	1.8	4.4	3.0	2.1	37.1	7.0	5.2	0.0	2.5	1.1	0.0	1.7	0.7	0.6	0.5	0.3
A+/C-/D-/D+	0.7	0.4	1.3	1.1	1.4	0.0	1.5	1.6	0.0	2.7	0.8	2.6	4.3	1.4	0.0	1.9	1.1	0.7	0.3	0.2
A+/C-/C+/E-	0.4	0.2	1.8	0.2	0.4	0.0	2.1	0.6	0.0	2.3	0.4	10.2	4.6	3.5	6.3	2.2	1.4	0.8	0.3	0.1
B-/B+/D-/D+	0.4	0.3	0.6	1.2	1.0	2.0	1.8	1.6	0.6	0.5	2.7	2.2	1.7	1.6	1.9	1.7	1.3	0.9	0.5	0.5
B-/B+/C+/D+	0.9	1.6	0.3	1.2	3.2	0.0	1.3	2.0	0.0	2.2	0.9	2.4	4.2	0.9	1.7	1.9	1.0	0.5	0.5	0.6
A+/C-/C+/D+	0.5	0.6	0.7	0.6	1.5	0.0	1.7	1.6	0.0	1.3	0.0	2.6	4.1	0.7	2.7	2.2	1.7	0.7	0.5	0.2
B-/C-/C+/D+	0.5	0.5	0.5	2.3	1.1	2.2	0.4	3.7	5.2	0.0	15.8	0.4	0.0	0.9	0.0	1.4	0.8	0.9	0.5	0.5
A+/B+/D-/D+	0.5	0.6	0.3	0.6	1.5	0.0	2.1	0.7	2.5	0.8	0.0	4.6	25.0	3.1	0.0	1.6	1.3	1.1	0.6	0.3
A+/B+/D-/D+/F-	0.3	0.0	1.3	0.3	0.6	0.0	2.3	0.6	0.0	8.7	0.0	1.9	0.0	4.0	11.1	1.9	1.9	0.9	0.7	0.4
A+/C-/C+/E-/E+	0.4	0.0	0.6	0.3	0.7	0.0	2.8	0.3	1.1	0.7	0.9	3.5	3.1	5.9	1.1	1.8	1.9	1.2	0.5	0.3
A+/C-/C+/D+/F-	0.4	0.0	1.2	0.3	0.4	0.0	2.1	0.9	0.0	5.0	0.0	6.3	0.0	1.1	44.4	2.1	1.6	1.0	0.4	0.0
B-/B+/D-/D+/E+	0.4	0.6	0.4	0.9	2.2	0.0	1.5	3.5	2.6	0.4	1.3	0.2	3.1	0.7	0.0	1.3	1.5	1.2	0.9	0.3
B-/B+/C+/E-/E+	0.5	0.6	0.6	0.6	0.6	0.0	0.8	1.0	2.6	0.4	5.9	1.8	0.8	2.3	0.0	1.3	1.4	1.3	1.1	0.6
complex (1-5)	0.8	0.9	1.0	0.9	1.3	1.2	1.6	1.4	1.7	1.9	1.4	2.2	1.6	1.8	2.1	2.1	1.4	0.8	0.4	0.2
complex (6-10)	0.3	0.2	0.4	0.4	0.5	0.1	1.2	0.8	0.7	1.4	0.8	1.4	1.3	1.9	1.6	1.4	2.4	1.6	0.8	0.4
complex (11-20)	0.2	0.2	0.2	0.2	0.4	0.3	0.6	0.6	0.6	0.4	0.9	0.8	1.1	1.2	1.0	0.8	1.6	2.2	1.5	0.8
complex (20-50)	0.1	0.1	0.1	0.2	0.2	0.1	0.3	0.3	0.5	0.2	0.5	0.3	0.6	0.5	0.4	0.4	0.8	1.5	2.5	1.8
complex (51)	0.1	0.0	0.0	0.2	0.1	0.1	0.2	0.3	0.3	0.1	0.5	0.1	0.3	0.3	0.0	0.2	0.4	0.8	1.8	3.9

References

1. Li, Y. *et al.* Constitutional and somatic rearrangement of chromosome 21 in acute lymphoblastic leukaemia. *Nature* **508**, 98–102 (2014).

2. Li, H. & Durbin, R. Fast and accurate long-read alignment with Burrows-Wheeler transform. *Bioinformatics* **26**, 589–595 (2010).

3. Raine, K. M. *et al.* ascatNgs: Identifying Somatically Acquired Copy-Number Alterations from Whole-Genome Sequencing Data. in *Current Protocols in Bioinformatics* **2016**, 15.9.1-15.9.17 (John Wiley & Sons, Inc., 2016).

4. Tsai, I. J. *et al.* Summarizing specific profiles in illumina sequencing from whole-genome amplified DNA. *DNA Res.* **21**, 243–254 (2014).

5. Nilsen, G. *et al.* Copynumber: Efficient algorithms for single- and multi-track copy number segmentation. *BMC Genomics* **13**, (2012).

6. Févotte, C., Bertin, N. & Durrieu, J.-L. Nonnegative matrix factorization with the Itakura-Saito divergence: with application to music analysis. *Neural Comput.* **21**, 793–830 (2009).

7. Bignell, G. R. *et al.* Signatures of mutation and selection in the cancer genome. *Nature* **463**, 893–898 (2010).

8. Le Tallec, B. *et al.* Common fragile site profiling in epithelial and erythroid cells reveals that most recurrent cancer deletions lie in fragile sites hosting large genes. *Cell Rep.* **4**, 420–428 (2013).

9. Berger, M. F. *et al.* The genomic complexity of primary human prostate cancer. *Nature* **470**, 214–220 (2011).

10. Nik-Zainal, S. *et al.* Landscape of somatic mutations in 560 breast cancer whole-genome sequences. *Nature* **534**, 47–54 (2016).

TCGA/ICGC Pan-Cancer Analysis of Whole Genomes Network: list of participants

Steering committee

Peter J Campbell#[1,2], **Gad Getz#**[3,4,5,6], **Jan O Korbel#**[7,8], **Lincoln D Stein#**[9,10] and **Joshua M Stuart#**[11]

Executive committee

Sultan T Al-Sedairy[12], Axel Aretz[13], Cindy Bell[14], Miguel Betancourt[15], Christiane Buchholz[16], Fabien Calvo[17], Christine Chomienne[18], Michael Dunn[19], Stuart Edmonds[20], Eric Green[21], Shailja Gupta[22], Carolyn M Hutter[21], Karine Jegalian[23], Jennifer L Jennings[24,25], Nic Jones[26], Hyung-Lae Kim[27], Youyong Lu[28,29,30], Hitoshi Nakagama[31], Gerd Nettekoven[32], Laura Planko[32], David Scott[26], Tatsuhiro Shibata[33,34], Kiyo Shimizu[35], Lincoln D Stein[9,10], Michael Rudolf Stratton[2], Takashi Yugawa[36], Giampaolo Tortora[37,38], K VijayRaghavan[22], Huanming Yang[39] and Jean C Zenklusen[40]

Ethics and legal working group

Don Chalmers#[41], Yann Joly[42], **Bartha M Knoppers#**[42], Fruzsina Molnár-Gábor[43], Mark Phillips[42], Adrian Thorogood[42] and David Townend[44]

Technical working group

Brice Aminou[45], Javier Bartolome[46], Keith A Boroevich[47,48], Rich Boyce[7], Angela N Brooks[3,49,50], Alex Buchanan[51], Ivo Buchhalter[52,53,54], Adam P Butler[2], Niall J Byrne[45], Andy Cafferkey[7], Peter J Campbell[1,2], Zhaohong Chen[55], Sunghoon Cho[56], Wan Choi[57], Peter Clapham[2], Brandi N Davis-Dusenbery[58], Francisco M De La Vega[59,59,60,61,62], Jonas Demeulemeester[63,64], Michelle T Dow[55], Lewis Jonathan Dursi[9,65], Juergen Eils[66,67], Roland Eils[52,54,66,67], Kyle Ellrott[51], Claudiu Farcas[55], Francesco Favero[68], Nodirjon Fayzullaev[45], Vincent Ferretti[45,69], Paul Flicek[7], Nuno A Fonseca[7,70], Josep Ll Gelpi[46,71], Gad Getz[3,4,5,6], Bob Gibson[45], Robert L Grossman[72], Olivier Harismendy[73], Allison P Heath[74], Michael C Heinold[52,54], Julian M Hess[3,75], Oliver Hofmann[76], Jongwhi H Hong[77], Thomas J Hudson[78,79], Barbara Hutter[80,81,82], Carolyn M Hutter[21], Daniel Hübschmann[54,66,83,84,85], Seiya Imoto[86,87], Sinisa Ivkovic[88], Seung-Hyup Jeon[57], Wei Jiao[9], Jongsun Jung[89], Rolf Kabbe[52], Andre Kahles[90,91,92,93,94], Jules NA Kerssemakers[52], Hyung-Lae Kim[27], Hyunghwan Kim[57], Jihoon Kim[95], Youngwook Kim[96,97], Kortine Kleinheinz[52,54], Jan O Korbel[7,8], Michael Koscher[98], Antonios Koures[55], Milena Kovacevic[88], Chris Lawerenz[67], Ignaty Leshchiner[3], Jia Liu[99], Dimitri Livitz[3], George L Mihaiescu[45], Sanja Mijalkovic[88], Ana Mijalkovic Mijalkovic-Lazic[88], Satoru Miyano[87], Naoki Miyoshi[87], Hardeep K Nahal-Bose[45], Hidewaki Nakagawa[48], Mia Nastic[88], Steven J Newhouse[7], Jonathan Nicholson[2], **Brian D O'Connor#**[45,50], David Ocana[7], Kazuhiro Ohi[86], Lucila Ohno-Machado[55], Larsson Omberg[100], BF Francis Ouellette[101,102], Nagarajan Paramasivam[52,81], Marc D Perry[45,103], Todd D Pihl[104], Manuel Prinz[52], Montserrat Puiggròs[105], Petar Radovic[88], Keiran M Raine[2], Esther Rheinbay[3,6,106], Mara Rosenberg[3,106], Romina Royo[105], Gunnar Rätsch[90,93,94,107,108,109], Gordon Saksena[3], Matthias Schlesner[52,110], Solomon I Shorser[9], Charles Short[7], Heidi J Sofia[21], Jonathan Spring[72], **Lincoln D Stein#**[9,10], Adam J Struck[51], Grace Tiao[3], Nebojsa Tijanic[88], David

Torrents[105,111], Peter Van Loo[63,64], Miguel Vazquez[105,112], David Vicente[105], Jeremiah A Wala[3,6,49], Zhining Wang[40], Sebastian M Waszak[8], Joachim Weischenfeldt[8,113,114], Johannes Werner[52,115], Ashley Williams[55], Youngchoon Woo[57], Adam J Wright[9], Qian Xiang[116], **Sergei Yakneen#**[8], Liming Yang[40], Denis Yuen[9], **Christina K Yung#**[45] and **Junjun Zhang#**[45]

Annotations working group

Angela N Brooks#[3,49,50], Ivo Buchhalter[52,53,54], Peter J Campbell[1,2], Priyanka Dhingra[117,118], Lars Feuerbach[119], Mark Gerstein[120,121,122,123], Gad Getz[3,4,5,6], Mark P Hamilton[124], Henrik Hornshøj[125], Todd A Johnson[47], Andre Kahles[90,91,92,93,94], Abdullah Kahraman[126,127,128], Manolis Kellis[3,129], **Ekta Khurana#**[117,118,130,131], Jan O Korbel[7,8], Morten Muhlig Nielsen[125], Jakob Skou Pedersen[125,132], Paz Polak[3,4,6], Jüri Reimand[9,133], Esther Rheinbay[3,6,106], Nicola D Roberts[2], Gunnar Rätsch[90,93,94,107,108,109], Richard Sallari[3], Nasa Sinnott-Armstrong[3,61], Alfonso Valencia[105,111], Miguel Vazquez[105,112], Sebastian M Waszak[8], Joachim Weischenfeldt[8,113,114] and Christian von Mering[128,134]

Quality control working group

Sergi Beltran[135,136], Ivo Buchhalter[52,53,54], Peter J Campbell[1,2], Roland Eils[52,54,66,67], Daniela S Gerhard[137], Gad Getz[3,4,5,6], **Ivo G Gut#**[135,136], Marta Gut[135,136], Barbara Hutter[80,81,82], Daniel Hübschmann[54,66,83,84,85], Kortine Kleinheinz[52,54], Jan O Korbel[7,8], Dimitri Livitz[3], Marc D Perry[45,103], Keiran M Raine[2], Esther Rheinbay[3,6,106], Mara Rosenberg[3,106], Gordon Saksena[3], Matthias Schlesner[52,110], Miranda D Stobbe[135,136], Jean-Rémi Trotta[135], Johannes Werner[52,115] and Justin P Whalley[135]

Novel somatic mutation calling methods

Matthew H Bailey[138,139], Beifang Niu[140], Matthias Bieg[81,141], Paul C Boutros[9,133,142,143], Ivo Buchhalter[52,53,54], Adam P Butler[2], Ken Chen[144], Zechen Chong[145], **Li Ding#**[138,139,146], Oliver Drechsel[136,147], Lewis Jonathan Dursi[9,65], Roland Eils[52,54,66,67], Kyle Ellrott[51], Shadrielle MG Espiritu[9], Yu Fan[148], Robert S Fulton[138,139,146], Shengjie Gao[149], Josep Ll Gelpi[46,71], Mark Gerstein[120,121,122,123], Gad Getz[3,4,5,6], Santiago Gonzalez[7,8], Ivo G Gut[135,136], Faraz Hach[150,151], Michael C Heinold[52,54], Julian M Hess[3,75], Jonathan Hinton[2], Taobo Hu[152], Vincent Huang[9], Yi Huang[153,154], Barbara Hutter[80,81,82], David R Jones[2], Jongsun Jung[89], Natalie Jäger[52], Hyung-Lae Kim[27], Kortine Kleinheinz[52,54], Sushant Kumar[122,123], Yogesh Kumar[152], Christopher M Lalansingh[9], Ignaty Leshchiner[3], Ivica Letunic[155], Dimitri Livitz[3], Eric Z Ma[152], Yosef E Maruvka[3,75,106], R Jay Mashl[139,156], Michael D McLellan[138,139,146], Andrew Menzies[2], Ana Milovanovic[46], Morten Muhlig Nielsen[125], Stephan Ossowski[136,147,157], Nagarajan Paramasivam[52,81], Jakob Skou Pedersen[125,132], Marc D Perry[45,103], Montserrat Puiggròs[105], Keiran M Raine[2], Esther Rheinbay[3,6,106], Romina Royo[105], S Cenk Sahinalp[151,158,159], Gordon Saksena[3], Iman Sarrafi[151,159], Matthias Schlesner[52,110], **Jared T Simpson#**[9,160], Lucy Stebbings[2], Chip Stewart[3], Miranda D Stobbe[135,136], Jon W Teague[2], Grace Tiao[3], David Torrents[105,111], Jeremiah A Wala[3,6,49], Jiayin Wang[139,154,161], Wenyi Wang[148], Sebastian M Waszak[8], Joachim Weischenfeldt[8,113,114], Michael C Wendl[139,162,163], Johannes Werner[52,115], David A Wheeler[164,165], Zhenggang Wu[152], Hong Xue[152], Sergei Yakneen[8], Takafumi N Yamaguchi[9], Kai Ye[161,166], Venkata D Yellapantula[167,168], Christina K Yung[45] and Junjun Zhang[45]

Drivers and functional interpretation

Federico Abascal[2], Samirkumar B Amin[169,170,171], Gary D Bader[10], Jonathan Barenboim[9], Rameen Beroukhim[3,6,172], Johanna Bertl[125,173], Keith A Boroevich[47,48], Søren Brunak[174,175], Peter J Campbell[1,2], Joana Carlevaro-Fita[176,177,178], Dimple Chakravarty[179], Calvin Wing Yiu Chan[52,180], Ken Chen[144], Jung Kyoon Choi[181], Jordi Deu-Pons[182,183], Priyanka Dhingra[117,118], Klev Diamanti[184], Lars Feuerbach[119], J Lynn Fink[105,185], Nuno A Fonseca[7,70], Joan Frigola[182], Carlo Gambacorti-Passerini[186], Dale W Garsed[187,188], **Mark Gerstein#**[120,121,122,123], **Gad Getz#**[3,4,5,6], Abel Gonzalez-Perez[183,189,190], Qianyun Guo[132], Ivo G Gut[135,136], David Haan[11], Mark P Hamilton[124], Nicholas J Haradhvala[3,106], Arif O Harmanci[123,191], Mohamed Helmy[192], Carl Herrmann[52,54,193], Julian M Hess[3,75], Asger Hobolth[132,173], Ermin Hodzic[159], Chen Hong[119,180], Henrik Hornshøj[125], Keren Isaev[9,133], Jose MG Izarzugaza[174], Rory Johnson[177,194], Todd A Johnson[47], Malene Juul[125], Randi Istrup Juul[125], Andre Kahles[90,91,92,93,94], Abdullah Kahraman[126,127,128], Manolis Kellis[3,129], Ekta Khurana[117,118,130,131], Jaegil Kim[3], Jong K Kim[195], Youngwook Kim[96,97], Jan Komorowski[184,196], Jan O Korbel[7,8], Sushant Kumar[122,123], Andrés Lanzós[177,178,194], **Erik Larsson#**[90], **Michael S Lawrence#**[3,47,106], Donghoon Lee[123], Kjong-Van Lehmann[90,92,93,94,197], Shantao Li[123], Xiaotong Li[123], Ziao Lin[3,198], Eric Minwei Liu[117,118,199], Lucas Lochovsky[170,200,201,202], Shaoke Lou[122,123], Tobias Madsen[125], Kathleen Marchal[203,204], Iñigo Martincorena[2], Alexander Martinez-Fundichely[117,118,130], Yosef E Maruvka[3,75,106], Patrick D McGillivray[122], William Meyerson[123,205], Ferran Muiños[183,190], Loris Mularoni[183,190], Hidewaki Nakagawa[48], Morten Muhlig Nielsen[125], Marta Paczkowska[9], Keunchil Park[206,207], Kiejung Park[208], **Jakob Skou Pedersen#**[125,132], Oriol Pich[183,190], Tirso Pons[209], Sergio Pulido-Tamayo[203,204], **Benjamin J Raphael#**[120], Jüri Reimand[9,133], Iker Reyes-Salazar[190], Matthew A Reyna[120], Esther Rheinbay[3,6,106], Mark A Rubin[131,194,210,211,212], Carlota Rubio-Perez[183,190,213], Radhakrishnan Sabarinathan[183,190,214], S Cenk Sahinalp[151,158,159], Gordon Saksena[3], Leonidas Salichos[122,123], Chris Sander[49,90,215,216], Steven E Schumacher[3,217], Mark Shackleton[188,218], Ofer Shapira[3,49], Ciyue Shen[216,219], Raunak Shrestha[151], Shimin Shuai[9,10], Nikos Sidiropoulos[113], Lina Sieverling[119,180], Nasa Sinnott-Armstrong[3,61], Lincoln D Stein[9,10], **Joshua M Stuart#**[11], David Tamborero[183,190], Grace Tiao[3], Tatsuhiko Tsunoda[47,220,221,222], Husen M Umer[184,223], Liis Uusküla-Reimand[224,225], Alfonso Valencia[105,111], Miguel Vazquez[105,112], Lieven PC Verbeke[204,226], Claes Wadelius[227], Lina Wadi[9], Jiayin Wang[139,154,161], Jonathan Warrell[122,123], Sebastian M Waszak[8], Joachim Weischenfeldt[8,113,114], **David A Wheeler#**[164,165], Guanming Wu[228], Jun Yu[229], [230], Jing Zhang[123], Xuanping Zhang[154,231], Yan Zhang[123,232,233], Zhongming Zhao[234], Lihua Zou[235] and Christian von Mering[128,134]

Integration of transcriptome and genome

Samirkumar B Amin[169,170,171], Philip Awadalla[9,10], Peter J Bailey[236], **Alvis Brazma#**[7], **Angela N Brooks#**[3,49,50], Claudia Calabrese[7,8], Aurélien Chateigner[45], Isidro Cortés-Ciriano[237,238,239], Brian Craft[240], David Craft[3], Chad J Creighton[241], Natalie R Davidson[90,92,93,109,197], Deniz Demircioğlu[242,243], Serap Erkek[8], Nuno A Fonseca[7,70], Milana Frenkel-Morgenstern[244], Mary J Goldman[240], Liliana Greger[7], Jonathan Göke[242,245], Yao He[246], Katherine A Hoadley[247,248], Yong Hou[39,249], Matthew R Huska[250], Andre Kahles[90,91,92,93,94], Ekta Khurana[117,118,130,131], Helena Kilpinen[251], Jan O Korbel[7,8], Fabien C Lamaze[9], Kjong-Van Lehmann[90,92,93,94,197], Chang Li[39,249], Siliang Li[39,249], Xiaobo Li[39,249], Xinyue Li[39], Dongbing Liu[39,249], Fenglin Liu[246,252], Xingmin Liu[39,249], Maximillian G Marin[50], Julia Markowski[250], Matthew Meyerson[3,6,49,177,253], Tannistha Nandi[254], Morten Muhlig Nielsen[125], Akinyemi I Ojesina[255,256,257], BF Francis Ouellette[101,102], Qiang Pan-Hammarström[39,258], Peter J Park[238,259], Chandra Sekhar Pedamallu[3,6,172], Jakob Skou Pedersen[125,132], Marc D Perry[45,103], **Gunnar Rätsch#**[90,93,94,107,108,109], Roland F Schwarz[7,84,250,260], Yuichi Shiraishi[87], Reiner Siebert[261,262], Cameron M Soulette[50], Stefan G Stark[93,197,263,264], Oliver Stegle[7,8,265], Hong Su[39,249], Patrick Tan[254,266,267,268], Bin Tean Teh[266,267,268,269,270], Lara Urban[7,8], Jian Wang[39], Sebastian M Waszak[8], Kui Wu[39,249], Qian Xiang[116], Heng Xiong[39,249], Sergei

Yakneen[8], Huanming Yang[39], Chen Ye[39,249], Christina K Yung[45], Fan Zhang[246], Junjun Zhang[45], Xiuqing Zhang[39], Zemin Zhang[246,271], Liangtao Zheng[246], Jingchun Zhu[240] and Shida Zhu[39,249]

Integration of epigenome and genome

Hiroyuki Aburatani[272], **Benjamin P Berman#**[273,274,275], Hans Binder[276,277], **Benedikt Brors#**[82,119,278], Huy Q Dinh[273], Lars Feuerbach[119], Shengjie Gao[149], Ivo G Gut[135,136], Simon C Heath[135,136], Steve Hoffmann[277,279,280,281], Charles D Imbusch[119], Ekta Khurana[117,118,130,131], Helene Kretzmer[277,281], Peter W Laird[282], Jose I Martin-Subero[111,283], Genta Nagae[272,284], **Christoph Plass#**[285], Paz Polak[3,4,6], Hui Shen[286], Reiner Siebert[261,262], Nasa Sinnott-Armstrong[3,61], Miranda D Stobbe[135,136], Qi Wang[98], Dieter Weichenhan[285], Sergei Yakneen[8] and Wanding Zhou[286]

Patterns of structural variations, signatures, genomic correlations, retrotransposons, mobile elements

Kadir C Akdemir[144], Eva G Alvarez[287,288,289], Adrian Baez-Ortega[290], **Rameen Beroukhim#**[3,6,172], Paul C Boutros[9,133,142,143], David D L Bowtell[187,291], Benedikt Brors[82,119,278], Kathleen H Burns[292], **Peter J Campbell#**[1,2], Kin Chan[293], Ken Chen[144], Isidro Cortés-Ciriano[237,238,239], Ana Dueso-Barroso[46], Andrew J Dunford[3], Paul A Edwards[294,295], Xavier Estivill[296], Dariush Etemadmoghadam[187,188], Lars Feuerbach[119], J Lynn Fink[105,185], Milana Frenkel-Morgenstern[244], Dale W Garsed[187,188], Mark Gerstein[120,121,122,123], Dmitry A Gordenin[297], David Haan[11], James E Haber[298], Julian M Hess[3,75], Barbara Hutter[80,81,82], Marcin Imielinski[299,300], David TW Jones[301,302], Young Seok Ju[2,181], Marat D Kazanov[303,304,305], Leszek J Klimczak[306], Youngil Koh[307,308], Jan O Korbel[7,8], Kiran Kumar[3], Eunjung Alice Lee[309], Jake June-Koo Lee[238,259], Yilong Li[2], Andy G Lynch[294,295,310], Geoff Macintyre[294], Florian Markowetz[294,295], Iñigo Martincorena[2], Alexander Martinez-Fundichely[117,118,130], Matthew Meyerson[3,6,49,177,253], Satoru Miyano[87], Hidewaki Nakagawa[48], Fabio CP Navarro[122], Stephan Ossowski[136,147,157], Peter J Park[238,259], John V Pearson[311,312], Montserrat Puiggròs[105], Karsten Rippe[84], Nicola D Roberts[2], Steven A Roberts[313], Bernardo Rodriguez-Martin[287,288,289], Steven E Schumacher[3,217], Ralph Scully[314], Mark Shackleton[188,218], Nikos Sidiropoulos[113], Lina Sieverling[119,180], Chip Stewart[3], David Torrents[105,111], Jose MC Tubio[287,288,289], Izar Villasante[105], Nicola Waddell[311,312], Jeremiah A Wala[3,6,49], Joachim Weischenfeldt[8,113,114], Lixing Yang[315], Xiaotong Yao[299,316], Sung-Soo Yoon[308], Jorge Zamora[2,287,288,289] and Cheng-Zhong Zhang[3,6,49]

Mutation signatures and processes

Ludmil B Alexandrov[2,317], Erik N Bergstrom[318], Arnoud Boot[267,319], Paul C Boutros[9,133,142,143], Kin Chan[293], Kyle Covington[165], Akihiro Fujimoto[48], Gad Getz[3,4,5,6], Dmitry A Gordenin[297], Nicholas J Haradhvala[3,106], Mi Ni Huang[267,319], S. M. Ashiqul Islam[317], Marat D Kazanov[303,304,305], Jaegil Kim[3], Leszek J Klimczak[306], Michael S Lawrence[3,47,106], Iñigo Martincorena[2], John R McPherson[267,319], Sandro Morganella[2], Ville Mustonen[320,321,322], Hidewaki Nakagawa[48], Avlin Wei Tian Ng[323], Serena Nik-Zainal[2,324,325,326], Paz Polak[3,4,6], Stephenie D Prokopec[9], Steven A Roberts[313], **Steven G Rozen#**[267,268,319], Radhakrishnan Sabarinathan[183,190,214], Natalie Saini[297], Tatsuhiro Shibata[33,34], Yuichi Shiraishi[87], **Michael Rudolf Stratton#**[2], **Bin Tean Teh#**[266,267,268,269,270], Ignacio Vázquez-García[2,167,327,328], Yang Wu[267,319], Fouad Yousif[9] and Willie Yu[329]

Germline cancer genome

Ludmil B Alexandrov[2,317], Eva G Alvarez[287,288,289], Adrian Baez-Ortega[290], Matthew H Bailey[138,139], Mattia Bosio[46,136,147], G Steven Bova[330], Alvis Brazma[7], Alicia L Bruzos[287,288,289], Ivo Buchhalter[52,53,54], Carlos D Bustamante[60,61], Atul J Butte[331], Andy Cafferkey[7], Claudia Calabrese[7,8], Peter J Campbell[1,2], Stephen J Chanock[332], Nilanjan Chatterjee[333,334], Jieming Chen[123,335], Francisco M De La Vega[59,59,60,61,62], Olivier Delaneau[336,337,338], German M Demidov[136,147,339], Anthony DiBiase[340], Li Ding[138,139,146], Oliver Drechsel[136,147], Lewis Jonathan Dursi[9,65], Douglas F Easton[341,342], Serap Erkek[8], Georgia Escaramis[147,343,344], **Xavier Estivill#**[296], Erik Garrison[2], Mark Gerstein[120,121,122,123], Gad Getz[3,4,5,6], Dmitry A Gordenin[297], Nina Habermann[8], Olivier Harismendy[73], Eoghan Harrington[345], Shuto Hayashi[87], José María Heredia-Genestar[346], Aliaksei Z Holik[147], Xing Hua[332], Kuan-lin Huang[139,347], Seiya Imoto[86,87], Sissel Juul[345], Ekta Khurana[117,118,130,131], Hyung-Lae Kim[27], Youngwook Kim[96,97], Leszek J Klimczak[306], **Jan O Korbel#**[7,8], Roelof Koster[348], Sushant Kumar[122,123], Ivica Letunic[155], Yilong Li[2], Tomas Marques-Bonet[111,135,346,349], R Jay Mashl[139,156], Simon Mayes[350], Michael D McLellan[138,139,146], Lisa Mirabello[332], Francesc Muyas[136,147,339], Hidewaki Nakagawa[48], Arcadi Navarro[111,135,346], Steven J Newhouse[7], Stephan Ossowski[136,147,157], Esa Pitkänen[8], Aparna Prasad[136], Raquel Rabionet[136,147,351], Benjamin Raeder[8], Tobias Rausch[8], Steven A Roberts[313], Bernardo Rodriguez-Martin[287,288,289], Gunnar Rätsch[90,93,94,107,108,109], Natalie Saini[297], Matthias Schlesner[52,110], Roland F Schwarz[7,84,250,260], Ayellet V Segre[3,3,352], Tal Shmaya[59], Suyash S Shringarpure[61], Nikos Sidiropoulos[113], Reiner Siebert[261,262], Jared T Simpson[9,160], Lei Song[332], Oliver Stegle[7,8,265], Hana Susak[136,147], Tomas J Tanskanen[353], Grace Tiao[3], Marta Tojo[289], Jose MC Tubio[287,288,289], Daniel J Turner[350], Lara Urban[7,8], Sebastian M Waszak[8], David C Wedge[2,354,355], Joachim Weischenfeldt[8,113,114], David A Wheeler[164,165], Mark H Wright[61], Dai-Ying Wu[59], Tian Xia[356], Sergei Yakneen[8], Kai Ye[161,166], Venkata D Yellapantula[167,168], Jorge Zamora[2,287,288,289] and Bin Zhu[332]

Tumor subtypes and clinical translation

Fatima Al-Shahrour[357], Gurnit Atwal[9,10,358], Peter J Bailey[236], **Andrew V Biankin#**[359,360,361,362], Paul C Boutros[9,133,142,143], Peter J Campbell[1,2], David K Chang[360,362], Susanna L Cooke[362], Vikram Deshpande[106], Bishoy M Faltas[109], William C Faquin[106], **Levi Garraway#**[49], Gad Getz[3,4,5,6], **Sean M Grimmond#**[363], Syed Haider[9], **Katherine A Hoadley#**[247,248], Wei Jiao[9], Vera B Kaiser[364], Rosa Karlić[365], Mamoru Kato[366], Kirsten Kübler[3,6,106], Alexander J Lazar[367], Constance H Li[9,133], David N Louis[106], Adam Margolin[368], Sancha Martin[2,369], Hardeep K Nahal-Bose[45], G Petur Nielsen[106], Serena Nik-Zainal[2,324,325,326], Larsson Omberg[100], Christine P'ng[9], Marc D Perry[45,103], Paz Polak[3,4,6], Esther Rheinbay[3,6,106], Mark A Rubin[131,194,210,211,212], Colin A Semple[364], Dennis C Sgroi[106], Tatsuhiro Shibata[33,34], Reiner Siebert[261,262], Jaclyn Smith[368], **Lincoln D Stein#**[9,10], Miranda D Stobbe[135,136], Ren X Sun[9], Kevin Thai[45], Derek W Wright[370,371], Chin-Lee Wu[106], Ke Yuan[294,369,372] and Junjun Zhang[45]

Evolution and heterogeneity

David J Adams[2], Pavana Anur[373], Rameen Beroukhim[3,6,172], Paul C Boutros[9,133,142,143], David D L Bowtell[187,291], Peter J Campbell[1,2], Shaolong Cao[148], Elizabeth L Christie[187], Marek Cmero[374,375,376], Yupeng Cun[377], Kevin J Dawson[2], Jonas Demeulemeester[63,64], Stefan C Dentro[2,64,354], Amit G Deshwar[378], Nilgun Donmez[151,159], Ruben M Drews[294], Roland Eils[52,54,66,67], Yu Fan[148], Matthew W Fittall[64], Dale W Garsed[187,188], Moritz Gerstung[7,8], Gad Getz[3,4,5,6], Santiago Gonzalez[7,8], Gavin Ha[3], Kerstin Haase[64], Marcin Imielinski[299,300], Lara Jerman[8,379], Yuan Ji[380,381], Clemency Jolly[64], Kortine Kleinheinz[52,54], Juhee Lee[382], Henry Lee-Six[2], Ignaty Leshchiner[3], Dimitri Livitz[3], Geoff Macintyre[294], Salem Malikic[151,159], Florian Markowetz[294,295], Iñigo Martincorena[2], Thomas J Mitchell[2,295,383], Quaid D Morris[358,384], Ville Mustonen[320,321,322], Layla Oesper[385], Martin Peifer[377], Myron

Peto[386], Benjamin J Raphael[120], Daniel Rosebrock[3], Yulia Rubanova[160,358], S Cenk Sahinalp[151,158,159], Adriana Salcedo[9], Matthias Schlesner[52,110], Steven E Schumacher[3,217], Subhajit Sengupta[387], Ruian Shi[384], Seung Jun Shin[264], **Paul T Spellman#**[388], Oliver Spiro[3], Lincoln D Stein[9,10], Maxime Tarabichi[2,64], **Peter Van Loo#**[63,64], Shankar Vembu[384,389], Ignacio Vázquez-García[2,167,327,328], Wenyi Wang[148], **David C Wedge#**[2,354,355], David A Wheeler[164,165], Jeffrey A Wintersinger[192,358,390], Tsun-Po Yang[377], Xiaotong Yao[299,316], Kaixian Yu[391], Ke Yuan[294,369,372] and Hongtu Zhu[392,393]

Exploratory: portals, visualization and software infrastructure

Fatima Al-Shahrour[357], Elisabet Barrera[7], Wojciech Bazant[7], Alvis Brazma[7], Isidro Cortés-Ciriano[237,238,239], Brian Craft[240], David Craft[3], Vincent Ferretti[45,69], Nuno A Fonseca[7,70], Anja Füllgrabe[7], Mary J Goldman[240], **David Haussler#**[240,394], Wolfgang Huber[8], Maria Keays[7], Alfonso Muñoz[7], Brian D O'Connor[45,50], Irene Papatheodorou[7], Robert Petryszak[7], Elena Piñeiro-Yáñez[357], Alfonso Valencia[105,111], **Miguel Vazquez#**[105,112], John N Weinstein[395,396], Qian Xiang[116], Junjun Zhang[45] and **Jingchun Zhu#**[240]

Exploratory: mitochondrial variants and HLA/immunogenicity

Peter J Campbell[1,2], Yiwen Chen[148], Chad J Creighton[241], Li Ding[138,139,146], Akihiro Fujimoto[48], Masashi Fujita[48], Gad Getz[3,4,5,6], Leng Han[231], Takanori Hasegawa[87], Shuto Hayashi[87], Seiya Imoto[86,87], Young Seok Ju[2,181], Hyung-Lae Kim[27], Youngwook Kim[96,97], Youngil Koh[307,308], Mitsuhiro Komura[87], Jun Li[148], **Han Liang#**[397], Iñigo Martincorena[2], Satoru Miyano[87], Shinichi Mizuno[398], **Hidewaki Nakagawa#**[48], Keunchil Park[206,207], Eigo Shimizu[87], Yumeng Wang[148,399], John N Weinstein[395,396], Yanxun Xu[400], Rui Yamaguchi[87], Fan Yang[384], Yang Yang[231], Christopher J Yoon[181], Sung-Soo Yoon[308], Yuan Yuan[148], Fan Zhang[246] and Zemin Zhang[246,271]

Exploratory: pathogens

Malik Alawi[401,402], Ivan Borozan[9], Daniel S Brewer[403,404], Colin S Cooper[404,405,406], Nikita Desai[45], **Roland Eils#**[52,54,66,67], Vincent Ferretti[45,69], Adam Grundhoff[401,407], Murat Iskar[408], Kortine Kleinheinz[52,54], Peter Lichter[408], **Hidewaki Nakagawa#**[48], Akinyemi I Ojesina[255,256,257], Chandra Sekhar Pedamallu[3,6,172], Matthias Schlesner[52,110], Xiaoping Su[144] and Marc Zapatka[408]

Tumor Specific Providers – Australia (Ovarian cancer)

Kathryn Alsop[409,410], Australian Ovarian Cancer Study Group[187,311,411], **David D L Bowtell#**[187,291], Timothy JC Bruxner[185], Angelika N Christ[185], Elizabeth L Christie[187], Stephen M Cordner[412], Prue A Cowin[187], Ronny Drapkin[413], Dariush Etemadmoghadam[187,188], Sian Fereday[414], Dale W Garsed[187,188], Joshy George[170], Sean M Grimmond[363], Anne Hamilton[187], Oliver Holmes[311,312], Jillian A Hung[415,416], Karin S Kassahn[185,417], Stephen H Kazakoff[311,312], Catherine J Kennedy[418,419], Conrad R Leonard[311,312], Linda Mileshkin[187], David K Miller[185,360,420], Gisela Mir Arnau[187], Chris Mitchell[187], Felicity Newell[311,312], Katia Nones[311,312], Ann-Marie Patch[311,312], John V Pearson[311,312], Michael C Quinn[311,312], Mark Shackleton[188,218], Darrin F Taylor[185], Heather Thorne[187], Nadia Traficante[187], Ravikiran Vedururu[187], Nick M Waddell[312], Nicola Waddell[311,312], Paul M Waring[253], Scott Wood[311,312], Qinying Xu[311,312] and Anna deFazio[421,422,423]

Tumor Specific Providers – Australia (Pancreatic cancer)

Matthew J Anderson[185], Davide Antonello[424], Andrew P Barbour[425,426], Claudio Bassi[424], Samantha Bersani[427], **Andrew V Biankin#**[359,360,361,362], Timothy JC Bruxner[185], Ivana Cataldo[427,428], David K Chang[360,362], Lorraine A Chantrill[360], Yoke-Eng Chiew[421], Angela Chou[360,429], Angelika N Christ[185], Sara Cingarlini[37], Nicole Cloonan[430], Vincenzo Corbo[428,431], [432], Fraser R Duthie[433,434], J Lynn Fink[105,185], Anthony J Gill[360,435], Janet S Graham[362,436], **Sean M Grimmond#**[363], Ivon Harliwong[185], Oliver Holmes[311,312], Nigel B Jamieson[361,362,437], Amber L Johns[360,420], Karin S Kassahn[185,417], Stephen H Kazakoff[311,312], James G Kench[360,435,438], Luca Landoni[424], Rita T Lawlor[428], Conrad R Leonard[311,312], Andrea Mafficini[428], Neil D Merrett[424,439], David K Miller[185,360,420], Marco Miotto[424], Elizabeth A Musgrove[362], Adnan M Nagrial[360], Felicity Newell[311,312], Katia Nones[311,312], Karin A Oien[253,440], Marina Pajic[360], Ann-Marie Patch[311,312], John V Pearson[311,312], Mark Pinese[360], Andreia V Pinho[360], Michael C Quinn[311,312], Alan J Robertson[185], Ilse Rooman[360], Borislav C Rusev[428], Jaswinder S Samra[424,435], Maria Scardoni[427], Christopher J Scarlett[360,441], Aldo Scarpa[428], Elisabetta Sereni[424], Katarzyna O Sikora[428], Michele Simbolo[431], Morgan L Taschuk[45], Christopher W Toon[360], Giampaolo Tortora[37,38], Caterina Vicentini[428], Nick M Waddell[312], Nicola Waddell[311,312], Scott Wood[311,312], Jianmin Wu[360], Qinying Xu[311,312] and Nikolajs Zeps[442]

Tumor Specific Providers – Australia (Skin cancer)

Lauri A Aaltonen[443], Andreas Behren[444], Hazel Burke[445], Jonathan Cebon[444], Rebecca A Dagg[446], Ricardo De Paoli-Iseppi[447], Ken Dutton-Regester[311], Matthew A Field[448], Anna Fitzgerald[449], Sean M Grimmond[363], **Nicholas K Hayward#**[311,445], Peter Hersey[445], Oliver Holmes[311,312], Valerie Jakrot[445], Peter A Johansson[311], Hojabr Kakavand[447], Stephen H Kazakoff[311,312], Richard F Kefford[450], Loretta MS Lau[451], Conrad R Leonard[311,312], Georgina V Long[452], **Graham J Mann#**[453,454], Felicity Newell[311,312], Katia Nones[311,312], Ann-Marie Patch[311,312], John V Pearson[311,312], Hilda A Pickett[451], Antonia L Pritchard[311], Gulietta M Pupo[455], Robyn PM Saw[452], Sarah-Jane Schramm[456], **Richard A Scolyer#**[422,452,457,458], Mark Shackleton[188,218], Catherine A Shang[459], Ping Shang[452], Andrew J Spillane[452], Jonathan R Stretch[452], Varsha Tembe[456], John F Thompson[452], Ricardo E Vilain[457], Nick M Waddell[312], Nicola Waddell[311,312], James S Wilmott[452], Scott Wood[311,312], Qinying Xu[311,312] and Jean Y Yang[460]

Tumor Specific Providers – Canada (Pancreatic cancer)

John Bartlett[461,462], Prashant Bavi[463], Ivan Borozan[9], Dianne E Chadwick[464], Michelle Chan-Seng-Yue[463], Sean Cleary[463,465], Ashton A Connor[466,467], Karolina Czajka[468], Robert E Denroche[463], Neesha C Dhani[469], Jenna Eagles[79], Vincent Ferretti[45,69], Steven Gallinger[463,466,467], Robert C Grant[463,470], David Hedley[469], Michael A Hollingsworth[471], **Thomas J Hudson#**[78,79], Gun Ho Jang[463], Jeremy Johns[79], Sangeetha Kalimuthu[463], Sheng-Ben Liang[472], Ilinca Lungu[463,473], Xuemei Luo[9], Faridah Mbabaali[79], **John D McPherson#**[79,463,474], Treasa A McPherson[470], Jessica K Miller[79], Malcolm J Moore[469], Faiyaz Notta[463,475], Danielle Pasternack[79], Gloria M Petersen[476], Michael H A Roehrl[133,463,477,478,479], Michelle Sam[79], Iris Selander[470], Stefano Serra[253], Sagedeh Shahabi[472], **Lincoln D Stein#**[9,10], Morgan L Taschuk[45], Sarah P Thayer[106], Lee E Timms[79], Gavin W Wilson[9,463], Julie M Wilson[463] and Bradly G Wouters[480]

Tumor Specific Providers – Canada (Prostate cancer)

Timothy A Beck[45], Vinayak Bhandari[9], Paul C Boutros[9,133,142,143], **Robert G Bristow#**[133,481,482,483,484], Colin C Collins[151], Shadrielle MG Espiritu[9], Neil E Fleshner[485], Natalie S Fox[9], Michael Fraser[9], Syed Haider[9], Lawrence E Heisler[486], Vincent Huang[9], Emilie Lalonde[9], Julie Livingstone[9], John D McPherson[79,463,474], Alice Meng[487], Veronica Y Sabelnykova[9], Adriana Salcedo[9], Yu-Jia Shiah[9], Theodorus Van der Kwast[488] and Takafumi N Yamaguchi[9]

Tumor Specific Providers – China (Gastric cancer)

Shuai Ding[489], Daiming Fan[490], Yong Hou[39,249], Yi Huang[153,154], Lin Li[39], Siliang Li[39,249], Dongbing Liu[39,249], Xingmin Liu[39,249], **Youyong Lu#**[28,29,30], Yongzhan Nie[490,491], Hong Su[39,249], Jian Wang[39], Kui Wu[39,249], Xiao Xiao[154], Rui Xing[29,492], **Huanming Yang#**[39], Shanlin Yang[489], Yingyan Yu[493], [230], Xiuqing Zhang[39], Yong Zhou[39] and Shida Zhu[39,249]

Tumor Specific Providers – EU: France (Renal cancer)

Rosamonde E Banks[494], Guillaume Bourque[495,496], Alvis Brazma[7], Paul Brennan[497], **Mark Lathrop#**[496], Louis Letourneau[498], Yasser Riazalhosseini[496], Ghislaine Scelo[497], **Jörg Tost#**[499], Naveen Vasudev[500] and Juris Viksna[501]

Tumor Specific Providers – EU: United Kingdom (Breast cancer)

Sung-Min Ahn[502], Ludmil B Alexandrov[2,317], Samuel Aparicio[503], Laurent Arnould[504], MR Aure[505], Shriram G Bhosle[2], E Birney[7], Ake Borg[506], S Boyault[507], AB Brinkman[508], JE Brock[509], A Broeks[510], Adam P Butler[2], AL Børresen-Dale[505], C Caldas[511,512], Peter J Campbell[1,2], Suet-Feung Chin[511,512], Helen Davies[2], C Desmedt[513], L Dirix[514], S Dronov[2], Anna Ehinger[515], JE Eyfjord[516], GG Van den Eynden[517], A Fatima[217], Jorge Reis Filho[518], JA Foekens[519], PA Futreal[520], Øystein Garred[521,522], Moritz Gerstung[7,8], Dilip D Giri[518], D Glodzik[2], Dorthe Grabau[523], Holmfridur Hilmarsdottir[516], GK Hooijer[524], Jocelyne Jacquemier[525], SJ Jang[526], Jon G Jonasson[516], Jos Jonkers[527], HY Kim[525], Tari A King[528,529], Stian Knappskog[2], G Kong[525], S Krishnamurthy[530], S Van Laere[514], SR Lakhani[531], A Langerød[505], Denis Larsimont[532], HJ Lee[526], JY Lee[533], Ming Ta Michael Lee[520], Yilong Li[2], Ole Christian Lingjærde[534], Gaetan MacGrogan[535], JW Martens[536], Sancha Martin[2,369], Iñigo Martincorena[2], Andrew Menzies[2], Sandro Morganella[2], Ville Mustonen[320,321,322], Serena Nik-Zainal[2,324,325,326], Sarah O'Meara[2], I Pauporté[18], Sarah Pinder[537], X Pivot[538], Elena Provenzano[539], CA Purdie[540], Keiran M Raine[2], M Ramakrishna[2], K Ramakrishnan[2], AL Richardson[217], M Ringnér[506], Javier Bartolomé Rodriguez[105], FG Rodríguez-González[175], G Romieu[541], Roberto Salgado[253], Torill Sauer[534], R Shepherd[2], AM Sieuwerts[177], PT Simpson[531], M Smid[542], C Sotiriou[55], PN Span[543], J Staaf[506], Lucy Stebbings[2], Ólafur Andri Stefánsson[544], Alasdair Stenhouse[545], **Michael Rudolf Stratton#**[2], HG Stunnenberg[249,546], Fred Sweep[547], BK Tan[548], Jon W Teague[2], Gilles Thomas[549], AM Thompson[545], S Tommasi[550], I Treilleux[551,552], Andrew Tutt[217], NT Ueno[393], Peter Van Loo[63,64], P Vermeulen[514], Alain Viari[428], MJ van de Vijver[253], A Vincent-Salomon[546], David C Wedge[2,354,355], Bernice Huimin Wong[553], Lucy Yates[2], X Zou[2], CHM van Deurzen[536] and L van't Veer[554,555]

Tumor Specific Providers – Germany (Malignant lymphoma)

Ole Ammerpohl[556,557], Sietse Aukema[558,559], Anke K Bergmann[560], Stephan H Bernhart[276,277,281], Hans Binder[276,277], Arndt Borkhardt[561], Christoph Borst[562], Benedikt Brors[82,119,278], Birgit Burkhardt[563],

Alexander Claviez[564], Roland Eils[52,54,66,67], Maria Elisabeth Goebler[565], Andrea Haake[556], Siegfried Haas[562], Martin Hansmann[566], Jessica I Hoell[561], Steve Hoffmann[277,279,280,281], Michael Hummel[567], Daniel Hübschmann[54,66,83,84,85], Dennis Karsch[568], Wolfram Klapper[559], Kortine Kleinheinz[52,54], Michael Kneba[568], Jan O Korbel[7,8], Helene Kretzmer[277,281], Markus Kreuz[569], Dieter Kube[570], Ralf Küppers[571], Chris Lawerenz[67], Dido Lenze[567], Peter Lichter[408], Markus Loeffler[569], Cristina López[262,556], Luisa Mantovani-Löffler[572], Peter Möller[573], German Ott[574], Bernhard Radlwimmer[408], Julia Richter[556,559], Marius Rohde[575], Philip C Rosenstiel[576], Andreas Rosenwald[577], Markus B Schilhabel[576], Matthias Schlesner[52,110], Stefan Schreiber[578], **Reiner Siebert#**[261,262], Peter F Stadler[276,277,281], Peter Staib[579], Stephan Stilgenbauer[580], Stephanie Sungalee[8], Monika Szczepanowski[559], Umut H Toprak[54,581], Lorenz HP Trümper[570], Rabea Wagener[262,556] and Thorsten Zenz[82]

Tumor Specific Providers – Germany (Pediatric Brain cancer)

Ivo Buchhalter[52,53,54], Juergen Eils[66,67], Roland Eils[52,54,66,67], Volker Hovestadt[408], Barbara Hutter[80,81,82], David TW Jones[301,302], Natalie Jäger[52], Christof von Kalle[84], Marcel Kool[98,301], Jan O Korbel[7,8], Andrey Korshunov[98], Pablo Landgraf[582], Chris Lawerenz[67], Hans Lehrach[583], **Peter Lichter#**[408], Paul A Northcott[584], Stefan M Pfister[98,301,585], Bernhard Radlwimmer[408], Guido Reifenberger[582], Matthias Schlesner[52,110], Hans-Jörg Warnatz[583], Joachim Weischenfeldt[8,113,114], Stephan Wolf[586], Marie-Laure Yaspo[583] and Marc Zapatka[408]

Tumor Specific Providers – Germany (Prostate cancer)

Yassen Assenov[587], Benedikt Brors[82,119,278], Juergen Eils[66,67], Roland Eils[52,54,66,67], Lars Feuerbach[119], Clarissa Gerhauser[285], Jan O Korbel[7,8], Chris Lawerenz[67], Hans Lehrach[583], Sarah Minner[588], Christoph Plass[285], **Guido Sauter#**[589], Thorsten Schlomm[114,590], Nikos Sidiropoulos[113], Ronald Simon[589], **Holger Sültmann#**[82,591], Hans-Jörg Warnatz[583], Dieter Weichenhan[285], Joachim Weischenfeldt[8,113,114] and Marie-Laure Yaspo[583]

Tumor Specific Providers – India (Oral cancer)

Nidhan K Biswas[592], Luca Landoni[424], Arindam Maitra[592], **Partha P Majumder#**[592] and **Rajiv Sarin#**[593]

Tumor Specific Providers – Italy (Pancreatic cancer)

Davide Antonello[424], Stefano Barbi[431], Claudio Bassi[424], Samantha Bersani[427], Giada Bonizzato[428], Cinzia Cantù[428], Ivana Cataldo[427,428], Sara Cingarlini[37], Vincenzo Corbo[428,431], [432], Angelo P Dei Tos[594], Matteo Fassan[595], Sonia Grimaldi[428], Luca Landoni[424], Rita T Lawlor[428], Claudio Luchini[427], Andrea Mafficini[428], Giuseppe Malleo[424], Giovanni Marchegiani[424], Michele Milella[37], Marco Miotto[424], Salvatore Paiella[424], Antonio Pea[424], Paolo Pederzoli[424], Borislav C Rusev[428], Andrea Ruzzenente[424], Roberto Salvia[424], Maria Scardoni[427], **Aldo Scarpa#**[428], Elisabetta Sereni[424], Michele Simbolo[431], Nicola Sperandio[428], Giampaolo Tortora[37,38] and Caterina Vicentini[428]

Tumor Specific Providers – Japan (Biliary tract cancer)

Yasuhito Arai[33], Natsuko Hama[33], Nobuyoshi Hiraoka[596], Fumie Hosoda[33,597], Mamoru Kato[366], Hiromi Nakamura[33], Hidenori Ojima[598], Takuji Okusaka[599], **Tatsuhiro Shibata#**[33,34], Yasushi Totoki[33] and Tomoko Urushidate[34]

Tumor Specific Providers – Japan (Gastric cancer)

Hiroyuki Aburatani#[272], Yasuhito Arai[33], Masashi Fukayama[600], Natsuko Hama[33], Fumie Hosoda[33,597], Shumpei Ishikawa[601], Hitoshi Katai[602], Mamoru Kato[366], Hiroto Katoh[603], Daisuke Komura[601], Genta Nagae[272,284], Hiromi Nakamura[33], Hirofumi Rokutan[604], Mihoko Saito-Adachi[33], **Tatsuhiro Shibata#**[33,34], Akihiro Suzuki[272,605], Hirokazu Taniguchi[606], Kenji Tatsuno[272], Yasushi Totoki[33], Tetsuo Ushiku[600], Shinichi Yachida[33,607] and Shogo Yamamoto[272]

Tumor Specific Providers – Japan (Liver cancer)

Hiroyuki Aburatani[272], Hiroshi Aikata[608], Koji Arihiro[608], Shun-ichi Ariizumi[609], Keith A Boroevich[47,48], Kazuaki Chayama[608], Akihiro Fujimoto[48], Masashi Fujita[48], Mayuko Furuta[48], Kunihito Gotoh[610], Natsuko Hama[33], Takanori Hasegawa[87], Shinya Hayami[611], Shuto Hayashi[87], Satoshi Hirano[612], Seiya Imoto[86,87], Mamoru Kato[366], Yoshiiku Kawakami[608], Kazuhiro Maejima[48], Satoru Miyano[87], Genta Nagae[272,284], **Hidewaki Nakagawa#**[48], Hiromi Nakamura[33], Toru Nakamura[612], Kaoru Nakano[48], Hideki Ohdan[608], Aya Sasaki-Oku[48], **Tatsuhiro Shibata#**[33,34], Yuichi Shiraishi[87], Hiroko Tanaka[87], Yasushi Totoki[33], Tatsuhiko Tsunoda[47,220,221,222], Masaki Ueno[611], Rui Yamaguchi[87], Masakazu Yamamoto[609] and Hiroki Yamaue[611]

Tumor Specific Providers – Singapore (Biliary tract cancer)

Su Pin Choo[613], Ioana Cutcutache[267,319], Narong Khuntikeo[424,614], John R McPherson[267,319], Choon Kiat Ong[615], Chawalit Pairojkul[253], Irinel Popescu[616], **Steven G Rozen#**[267,268,319], **Patrick Tan#**[254,266,267,268] and **Bin Tean Teh#**[266,267,268,269,270]

Tumor Specific Providers – South Korea (Blood cancer)

Keun Soo Ahn[617], Hyung-Lae Kim[27], Youngil Koh[307,308] and **Sung-Soo Yoon#**[308]

Tumor Specific Providers – Spain (Chronic Lymphocytic Leukemia)

Marta Aymerich[618], **Elias Campo#**[619,620], Josep Ll Gelpi[46,71], Ivo G Gut[135,136], Marta Gut[135,136], Armando Lopez-Guillermo[621], Carlos López-Otín[622], Xose S Puente[623], Romina Royo[105] and David Torrents[105,111]

Tumor Specific Providers – United Kingdom (Bone cancer)

Fernanda Amary[624], Daniel Baumhoer[625], Sam Behjati[2], Bodil Bjerkehagen[626], **Peter J Campbell#**[1,2], **Adrienne M Flanagan#**[627], PA Futreal[520], Ola Myklebost[628], Nischalan Pillay[629], Patrick Tarpey[630], Roberto Tirabosco[631] and Olga Zaikova[632]

Tumor Specific Providers – United Kingdom (Chronic myeloid disorders)

Jacqueline Boultwood[633], David T Bowen[2], Adam P Butler[2], **Peter J Campbell#**[1,2], Mario Cazzola[634], Carlo Gambacorti-Passerini[186], Anthony R Green[295], Eva Hellstrom-Lindberg[635], Luca Malcovati[634], Sancha Martin[2,369], Jyoti Nangalia[636], Elli Papaemmanuil[2] and Paresh Vyas[311,637]

Tumor Specific Providers – United Kingdom (Esophageal cancer)

Yeng Ang[638], Hugh Barr[639], Duncan Beardsmore[640], Matthew Eldridge[294], **Rebecca C Fitzgerald#**[325], James Gossage[641], Nicola Grehan[325], George B Hanna[642], Stephen J Hayes[643,644], Ted R Hupp[645], David Khoo[646], Jesper Lagergren[635,647], Laurence E Lovat[251], Shona MacRae[395], Maria O'Donovan[325], J Robert O'Neill[648], Simon L Parsons[649], Shaun R Preston[650], Sonia Puig[651], Tom Roques[652], Grant Sanders[248], Sharmila Sothi[653], Simon Tavaré[294], Olga Tucker[654], Richard Turkington[655], Timothy J Underwood[656] and Ian Welch[657]

Tumor Specific Providers – United Kingdom (Prostate cancer)

Nicholas Van As[658], Daniel M Berney[659], Johann S De Bono[405], G Steven Bova[330], Daniel S Brewer[403,404], Adam P Butler[2], Declan Cahill[658], Niedzica Camacho[405], **Colin S Cooper#**[404,405,406], Nening M Dennis[658], Tim Dudderidge[658], Sandra E Edwards[405], **Rosalind A Eeles#**[405,658], Cyril Fisher[658], Christopher S Foster[660,661], Mohammed Ghori[2], Pelvender Gill[637], Vincent J Gnanapragasam[383,662], Gunes Gundem[2], Freddie C Hamdy[663], Steve Hawkins[294], Steven Hazell[658], William Howat[383], William B Isaacs[292], Katalin Karaszi[637], Jonathan D Kay[251], Vincent Khoo[658], Zsofia Kote-Jarai[405], Barbara Kremeyer[2], Pardeep Kumar[658], Adam Lambert[637], Daniel A Leongamornlert[2,405], Naomi Livni[658], Hayley J Luxton[251], Andy G Lynch[294,295,310], Luke Marsden[637], Charlie E Massie[294], Lucy Matthews[405], Erik Mayer[658,664], Ultan McDermott[2], Sue Merson[405], Thomas J Mitchell[2,295,383], David E Neal[294,383], Anthony Ng[665], David Nicol[658], Christopher Ogden[658], Edward W Rowe[658], Nimish C Shah[383], Jon W Teague[2], Sarah Thomas[658], Alan Thompson[658], Peter Van Loo[63,64], Clare Verrill[637,666], Tapio Visakorpi[330], Anne Y Warren[383,667], David C Wedge[2,354,355], Hayley C Whitaker[251], Yong-Jie Yu[659], Yongwei Yu[230], Jorge Zamora[2,287,288,289] and Hongwei Zhang[230]

Tumor Specific Providers – United States (TCGA)

Adam Abeshouse[199], Nishant Agrawal[72], Rehan Akbani[325,668], Hikmat Al-Ahmadie[199], Monique Albert[462], Kenneth Aldape[253,646,669], Adrian Ally[670], Yeng Ang[638], Elizabeth L Appelbaum[139,251], Joshua Armenia[671], Sylvia Asa[649,672], J Todd Auman[673], Matthew H Bailey[138,139], Miruna Balasundaram[670], Saianand Balu[248], Jill Barnholtz-Sloan[674,675], Hugh Barr[639], John Bartlett[461,462], Oliver F Bathe[676,677], Stephen B Baylin[656,678], Duncan Beardsmore[640], Christopher Benz[679], Andrew Berchuck[680], Benjamin P Berman[273,274,275], Rameen Beroukhim[3,6,172], Mario Berrios[681], Darell Bigner[294,682], Michael Birrer[106], Tom Bodenheimer[248], Lori Boice[651], Moiz S Bootwalla[683], Marcus Bosenberg[684], Reanne Bowlby[670], Jeffrey Boyd[685], Russell R Broaddus[669], Malcolm Brock[686], Denise Brooks[670], Susan Bullman[3,172], Samantha J Caesar-Johnson[40], Thomas E Carey[687], Rebecca Carlsen[670], Robert Cerfolio[688], Vishal S Chandan[689], Hsiao-Wei Chen[638,671], Andrew D Cherniack[3,3,49,172], Jeremy Chien[690], Juok Cho[3], Eric Chuah[670], Carrie Cibulskis[3], Kristian Cibulskis[3], Leslie Cope[691], Matthew G Cordes[139,652], Kyle Covington[165], Erin Curley[692], Bogdan Czerniak[646,669], Ludmila Danilova[691], Ian J

Davis[693], Timothy Defreitas[3], John A Demchok[40], Noreen Dhalla[670], Rajiv Dhir[694], Li Ding[138,139,146], HarshaVardhan Doddapaneni[165], Adel El-Naggar[646,669], Ina Felau[40], Martin L Ferguson[695], Gaetano Finocchiaro[696], Kwun M Fong[697], Scott Frazer[3], William Friedman[698], Catrina C Fronick[139,652], Lucinda A Fulton[139], Robert S Fulton[138,139,146], Stacey B Gabriel[3], Jianjiong Gao[671], Nils Gehlenborg[3,699], Jeffrey E Gershenwald[700,701], Gad Getz[3,4,5,6], Ronald Ghossein[518], Nasra H Giama[702], Richard A Gibbs[165], Carmen Gomez[703], James Gossage[641], Ramaswamy Govindan[138], Nicola Grehan[325], George B Hanna[642], D Neil Hayes[248,704,705], Stephen J Hayes[643,644], Apurva M Hegde[395,668], David I Heiman[3], Zachary Heins[199], Austin J Hepperla[248], Katherine A Hoadley[247,248], Andrea Holbrook[706], Robert A Holt[670], Alan P Hoyle[248], Ralph H Hruban[707], Jianhong Hu[165], Mei Huang[651], David Huntsman[708], Ted R Hupp[645], Jason Huse[199], **Carolyn M Hutter#**[21], Christine A Iacobuzio-Donahue[518], Michael Ittmann[709,710], Joy C Jayaseelan[165], Stuart R Jefferys[248], Corbin D Jones[711], Steven JM Jones[712], Hartmut Juhl[713], Koo Jeong Kang[714], Beth Karlan[715], Katayoon Kasaian[716], Electron Kebebew[717,718], David Khoo[646], Hark Kim[31], Jaegil Kim[3], Tari A King[528,529], Viktoriya Korchina[165], Ritika Kundra[638,671], Jesper Lagergren[635,647], Phillip H Lai[706], Peter W Laird[282], Eric Lander[3], Michael S Lawrence[3,47,106], Alexander J Lazar[367], Xuan Le[719], Darlene Lee[670], Douglas A Levine[199,720], Lora Lewis[165], Tim Ley[721], Haiyan Irene Li[670], Pei Lin[3], W Marston Linehan[722], Eric Minwei Liu[117,118,199], Fei Fei Liu[384], Laurence E Lovat[251], Yiling Lu[723], Lisa Lype[724], Yussanne Ma[670], Shona MacRae[395], Dennis T Maglinte[706], Elaine R Mardis[139,685,725], Jeffrey Marks[424,726], Marco A Marra[670], Thomas J Matthew[50], Michael Mayo[670], Karen McCune[727], Michael D McLellan[138,139,146], Samuel R Meier[3], Shaowu Meng[248], Matthew Meyerson[3,6,49,177,253], Piotr A Mieczkowski[247], Tom Mikkelsen[728], Christopher A Miller[139], Gordon B Mills[368,395,668], Richard A Moore[670], Carl Morrison[253,729], Lisle E Mose[248], Catherine D Moser[702], Andrew J Mungall[670], Karen Mungall[670], David Mutch[730], Donna M Muzny[165], Jerome Myers[731], Yulia Newton[50], Michael S Noble[3], Peter O'Donnell[732], Brian Patrick O'Neill[733], Angelica Ochoa[199], Akinyemi I Ojesina[255,256,257], Joong Won Park[31], Joel S Parker[734], Simon L Parsons[649], Harvey Pass[735], Alessandro Pastore[90], Chandra Sekhar Pedamallu[3,6,172], Nathan A Pennell[736], Charles M Perou[737], Gloria M Petersen[476], Nicholas Petrelli[738], Olga Potapova[739], Shaun R Preston[650], Sonia Puig[651], Janet S Rader[740], Suresh Ramalingam[741], W Kimryn Rathmell[742], Victor Reuter[253,518], Sheila M Reynolds[724], Matthew Ringel[743], Jeffrey Roach[744], Lewis R Roberts[702], A Gordon Robertson[670], Tom Roques[652], Mark A Rubin[131,194,210,211,212], Sara Sadeghi[670], Gordon Saksena[3], Charles Saller[745], Francisco Sanchez-Vega[638,671], Chris Sander[49,90,215,216], Grant Sanders[248], Dirk Schadendorf[80,746], Jacqueline E Schein[670], Heather K Schmidt[139], Nikolaus Schultz[671], Steven E Schumacher[3,217], Richard A Scolyer[422,452,457,458], Raja Seethala[747], Yasin Senbabaoglu[90], Troy Shelton[692], Yan Shi[248], Juliann Shih[3,172,177], Ilya Shmulevich[724], Craig Shriver[748], Sabina Signoretti[172,177,749], Janae V Simons[248], Samuel Singer[424,750], Payal Sipahimalani[670], Tara J Skelly[247], Karen Smith-McCune[727], Nicholas D Socci[90], Heidi J Sofia[21], Matthew G Soloway[734], Anil K Sood[751], Sharmila Sothi[653], Angela Tam[670], Donghui Tan[247], Roy Tarnuzzer[40], Nina Thiessen[670], R Houston Thompson[752], Leigh B Thorne[651], Ming Tsao[649,672], Olga Tucker[654], Richard Turkington[655], Christopher Umbricht[640,753], Timothy J Underwood[656], David J Van Den Berg[681], Erwin G Van Meir[754], Umadevi Veluvolu[247], Douglas Voet[3], Jiayin Wang[139,154,161], Linghua Wang[165], Zhining Wang[40], Paul Weinberger[755], John N Weinstein[395,396], Daniel J Weisenberger[706], Ian Welch[657], David A Wheeler[164,165], Dennis Wigle[756], Matthew D Wilkerson[247], Richard K Wilson[139,757], Boris Winterhoff[758], Maciej Wiznerowicz[759,760], Tina Wong[139,670], Winghing Wong[139], Liu Xi[165], Liming Yang[40], Christina Yau[294,679,680], Venkata D Yellapantula[167,168], **Jean C Zenklusen#**[40], Hailei Zhang[3], Hongxin Zhang[671] and Jiashan Zhang[40]

# Denotes **working group or project co-leader**

Author Affiliations

1. Department of Haematology, University of Cambridge, Cambridge CB2 2XY, UK.
2. Wellcome Sanger Institute, Wellcome Genome Campus, Hinxton, Cambridge, CB10 1SA, UK.
3. Broad Institute of MIT and Harvard, Cambridge, MA 02142, USA.
4. Center for Cancer Research, Massachusetts General Hospital, Boston, MA 02129, USA.
5. Department of Pathology, Massachusetts General Hospital, Boston, MA 02115, USA.
6. Harvard Medical School, Boston, MA 02115, USA.
7. European Molecular Biology Laboratory, European Bioinformatics Institute (EMBL-EBI), Wellcome Genome Campus, Hinxton, Cambridge, CB10 1SD, UK.
8. Genome Biology Unit, European Molecular Biology Laboratory (EMBL), Heidelberg 69117, Germany.
9. Computational Biology Program, Ontario Institute for Cancer Research, Toronto, ON M5G 0A3, Canada.
10. Department of Molecular Genetics, University of Toronto, Toronto, ON M5S 1A8, Canada.
11. Biomolecular Engineering Department, University of California, Santa Cruz, Santa Cruz, CA 95064, USA.
12. King Faisal Specialist Hospital and Research Centre, Al Maather, Riyadh 12713, Saudi Arabia.
13. DLR Project Management Agency, Bonn 53227, Germany.
14. Genome Canada, Ottawa, ON K2P 1P1, Canada.
15. Instituto Carlos Slim de la Salud, Mexico City, Mexico.
16. Federal Ministry of Education and Research, Berlin 10117, Germany.
17. Institut Gustave Roussy, Villejuif 94805, France.
18. Institut National du Cancer (INCA), Boulogne-Billancourt 92100, France.
19. The Wellcome Trust, London NW1 2BE, UK.
20. Prostate Cancer Canada, Toronto, ON M5C 1M1, Canada.
21. National Human Genome Research Institute, National Institutes of Health, Bethesda, MD 20892, USA.
22. Department of Biotechnology, Ministry of Science & Technology, Government of India, New Delhi, Delhi 110003, India.
23. Science Writer, Garrett Park, MD 20896, USA.
24. International Cancer Genome Consortium (ICGC)/ICGC Accelerating Research in Genomic Oncology (ARGO) Secretariat, Toronto, ON M5G 0A3, Canada.
25. Adaptive Oncology Initiative, Ontario Institute for Cancer Research, Toronto, ON M5G 0A3, Canada.
26. Cancer Research UK, London EC1V 4AD, UK.
27. Department of Biochemistry, College of Medicine, Ewha Womans University, Seoul 07895, South Korea.
28. Chinese Cancer Genome Consortium, Shenzhen 518083, China.
29. Laboratory of Molecular Oncology, Beijing,100142, China.
30. Peking University Cancer Hospital & Institute, Key Laboratory of Carcinogenesis and Translational Research (Ministry of Education), Peking University Cancer Hospital & Institute, Beijing 100142, China.
31. National Cancer Center, Tokyo 104-0045, Japan.

32. German Cancer Aid, Bonn 53113, Germany.
33. Division of Cancer Genomics, National Cancer Center Research Institute, Tokyo 104-0045, Japan.
34. Laboratory of Molecular Medicine, Human Genome Center, The Institute of Medical Science, The University of Tokyo, Minato-ku, Tokyo 108-8639, Japan.
35. Japan Agency for Medical Research and Development, Chiyoda-ku, Tokyo 100-0004 Japan.
36. Japan Agency for Medical Research and Development, Chiyoda-ku, Tokyo 100-0004, Japan.
37. Medical Oncology, University and Hospital Trust of Verona, Verona 37134, Italy.
38. University of Verona, Verona 37129, Italy.
39. BGI-Shenzhen, Shenzhen 518083, China.
40. National Cancer Institute, National Institutes of Health, Bethesda, MD 20892, USA.
41. Centre for Law and Genetics, University of Tasmania, Sandy Bay Campus, Hobart, Tasmania 7001 Australia.
42. Centre of Genomics and Policy, McGill University and Génome Québec Innovation Centre, Montreal, QC H3A 1A4, Canada.
43. Heidelberg Academy of Sciences and Humanities, Heidelberg 69120, Germany.
44. CAPHRI Research School, Maastricht University, Maastricht, ER 6229, The Netherlands.
45. Genome Informatics Program, Ontario Institute for Cancer Research, Toronto, ON M5G 0A3, Canada.
46. Barcelona Supercomputing Center (BSC), Barcelona 08034, Spain.
47. Laboratory for Medical Science Mathematics, RIKEN Center for Integrative Medical Sciences, Yokohama, Kanagawa 230-0045, Japan.
48. RIKEN Center for Integrative Medical Sciences, Yokohama, Kanagawa 230-0045, Japan.
49. Dana-Farber Cancer Institute, Boston, MA 02215, USA.
50. University of California Santa Cruz, Santa Cruz, CA 95064, USA.
51. Oregon Health and Science University, Portland, OR 97239, USA.
52. Division of Theoretical Bioinformatics, German Cancer Research Center (DKFZ), Heidelberg 69120, Germany.
53. Heidelberg Center for Personalized Oncology (DKFZ-HIPO), German Cancer Research Center, Heidelberg 69120, Germany.
54. Institute of Pharmacy and Molecular Biotechnology and BioQuant, Heidelberg University, Heidelberg 69120, Germany.
55. University of California San Diego, San Diego, CA 92093, USA.
56. PDXen Biosystems Inc, Seoul 4900, South Korea.
57. Electronics and Telecommunications Research Institute, Daejoen 34129, South Korea.
58. Seven Bridges Genomics, Charlestown, MA 02129, USA.
59. Annai Systems, Inc, Carlsbad, CA 92013, USA.
60. Department of Biomedical Data Science, Stanford University School of Medicine, Stanford, CA 94305, USA.
61. Department of Genetics, Stanford University School of Medicine, Stanford, CA 94305, USA.
62. Departments of Genetics and Biomedical Data Science, Stanford University School of Medicine, Stanford, CA 94305, USA.
63. University of Leuven, Leuven B-3000, Belgium.
64. The Francis Crick Institute, London NW1 1AT, UK.

65. The Hospital for Sick Children, Toronto, ON M5G 0A4, Canada.
66. Heidelberg University, Heidelberg 69120, Germany.
67. New BIH Digital Health Center, Berlin Institute of Health (BIH) and Charité - Universitätsmedizin Berlin, Berlin 10117, Germany.
68. Rigshospitalet, Copenhagen 2200, Denmark.
69. Department of Biochemistry and Molecular Medicine, University of Montreal, Montreal, QC H3C 3J7, Canada.
70. CIBIO/InBIO - Research Center in Biodiversity and Genetic Resources, Universidade do Porto, Vairão 4485-601, Portugal.
71. Department Biochemistry and Molecular Biomedicine, University of Barcelona, Barcelona 08028, Spain.
72. University of Chicago, Chicago, IL 60637, USA.
73. Division of Biomedical Informatics, Department of Medicine, & Moores Cancer Center, UC San Diego School of Medicine, San Diego, CA 92093, USA.
74. Children's Hospital of Philadelphia, Philadelphia, PA 19146, USA.
75. Massachusetts General Hospital Center for Cancer Research, Charlestown, MA 02129, USA.
76. University of Melbourne Centre for Cancer Research, University of Melbourne, Melbourne, VIC 3010, Australia.
77. Syntekabio Inc, Daejon 34025, South Korea.
78. AbbVie, North Chicago, IL 60064, USA.
79. Genomics Program, Ontario Institute for Cancer Research, Toronto, ON M5G 0A3, Canada.
80. German Cancer Consortium (DKTK), Heidelberg 69120, Germany.
81. Heidelberg Center for Personalized Oncology (DKFZ-HIPO), German Cancer Research Center (DKFZ), Heidelberg 69120, Germany.
82. National Center for Tumor Diseases (NCT) Heidelberg, Heidelberg 69120, Germany.
83. Department of Pediatric Immunology, Hematology and Oncology, University Hospital, Heidelberg 69120, Germany.
84. German Cancer Research Center (DKFZ), Heidelberg 69120, Germany.
85. Heidelberg Institute for Stem Cell Technology and Experimental Medicine (HI-STEM), Heidelberg 69120, Germany.
86. Institute of Medical Science, University of Tokyo, Tokyo 108-8639, Japan.
87. The Institute of Medical Science, The University of Tokyo, Tokyo 108-8639, Japan.
88. Seven Bridges, Charlestown, MA 02129, USA.
89. Genome Integration Data Center, Syntekabio, Inc, Daejon, 34025, South Korea.
90. Computational Biology Center, Memorial Sloan Kettering Cancer Center, New York, NY 10065, USA.
91. ETH Zurich, Department of Biology, Zürich 8093, Switzerland.
92. ETH Zurich, Department of Computer Science, Zurich 8092, Switzerland.
93. SIB Swiss Institute of Bioinformatics, Lausanne 1015, Switzerland.
94. University Hospital Zurich, Zurich, 8091, Switzerland.
95. Health Sciences Department of Biomedical Informatics, University of California San Diego, La Jolla, CA 92093, USA.
96. Department of Health Sciences and Technology, Sungkyunkwan University School of Medicine, Seoul 06351, South Korea.
97. Samsung Genome Institute, Seoul 06351, South Korea.

98. Functional and Structural Genomics, German Cancer Research Center (DKFZ), Heidelberg 69120, Germany.
99. Leidos Biomedical Research, Inc, McLean, VA 22102, USA.
100. Sage Bionetworks, Seattle WA 98109, USA.
101. Genome Informatics, Ontario Institute for Cancer Research, Toronto, ON M5G 2C4, Canada.
102. Department of Cell and Systems Biology, University of Toronto, Toronto, ON M5S 3G5, Canada.
103. Department of Radiation Oncology, University of California San Francisco, San Francisco, CA 94518, USA.
104. CSRA Incorporated, Fairfax, VA 22042, USA.
105. Barcelona Supercomputing Center, Barcelona 08034, Spain.
106. Massachusetts General Hospital, Boston, MA 02114, USA.
107. Department of Biology, ETH Zurich, Wolfgang-Pauli-Strasse 27, 8093 Zürich, Switzerland.
108. Department of Computer Science, ETH Zurich, Zurich 8092, Switzerland.
109. Weill Cornell Medical College, New York, NY 10065, USA.
110. Bioinformatics and Omics Data Analytics, German Cancer Research Center (DKFZ), Heidelberg 69120, Germany.
111. Institució Catalana de Recerca i Estudis Avançats (ICREA), Barcelona 08010, Spain.
112. Department of Clinical and Molecular Medicine, Faculty of Medicine and Health Sciences, Norwegian University of Science and Technology, Trondheim 7030, Norway.
113. Finsen Laboratory and Biotech Research & Innovation Centre (BRIC), University of Copenhagen, Copenhagen 2200, Denmark.
114. Department of Urology, Charité Universitätsmedizin Berlin, Berlin 10117, Germany.
115. Department of Biological Oceanography, Leibniz Institute of Baltic Sea Research, Seestraße 15, Rostock 18119, Germany.
116. Ontario Institute for Cancer Research, Toronto, ON M5G 0A3, Canada.
117. Department of Physiology and Biophysics, Weill Cornell Medicine, New York, NY 10065, USA.
118. Institute for Computational Biomedicine, Weill Cornell Medicine, New York, NY 10021, USA.
119. Division of Applied Bioinformatics, German Cancer Research Center (DKFZ), Heidelberg 69120, Germany.
120. Department of Computer Science, Princeton University, Princeton, NJ 08540, USA.
121. Department of Computer Science, Yale University, New Haven, CT 06520, USA.
122. Department of Molecular Biophysics and Biochemistry, Yale University, New Haven, CT 06520, USA.
123. Program in Computational Biology and Bioinformatics, Yale University, New Haven, CT 06520, USA.
124. Department of Internal Medicine, Stanford University, Stanford, CA 94305, USA.
125. Department of Molecular Medicine (MOMA), Aarhus University Hospital, Aarhus N 8200, Denmark.
126. Clinical Bioinformatics, Swiss Institute of Bioinformatics, Geneva 1202, Switzerland.
127. Institute for Pathology and Molecular Pathology, University Hospital Zurich, Zurich 8091, Switzerland.
128. Institute of Molecular Life Sciences, University of Zurich, Zurich 8057, Switzerland.

129. MIT Computer Science and Artificial Intelligence Laboratory, Massachusetts Institute of Technology, Cambridge, MA 02139, USA.
130. Englander Institute for Precision Medicine, Weill Cornell Medicine, New York, NY 10065, USA.
131. Meyer Cancer Center, Weill Cornell Medicine, New York, NY 10065, USA.
132. Bioinformatics Research Centre (BiRC), Aarhus University, Aarhus 8000, Denmark.
133. Department of Medical Biophysics, University of Toronto, Toronto, ON M5S 1A8, Canada.
134. Institute of Molecular Life Sciences and Swiss Institute of Bioinformatics, University of Zurich, Zurich 8057, Switzerland.
135. CNAG-CRG, Centre for Genomic Regulation (CRG), Barcelona Institute of Science and Technology (BIST), Barcelona 08028, Spain.
136. Universitat Pompeu Fabra (UPF), Barcelona 08003, Spain.
137. Office of Cancer Genomics, National Cancer Institute, US National Institutes of Health, Bethesda, MD 20892, USA.
138. Alvin J. Siteman Cancer Center, Washington University School of Medicine, St Louis, MO 63110, USA.
139. The McDonnell Genome Institute at Washington University, St Louis, MO 63108, USA.
140. Computer Network Information Center,Chinese Academy of Sciences, Beijing 100190, China.
141. Center for Digital Health, Berlin Institute of Health and Charitè - Universitätsmedizin Berlin, Berlin 10117, Germany.
142. Department of Pharmacology, University of Toronto, Toronto, ON M5S 1A8, Canada.
143. University of California Los Angeles, Los Angeles, CA 90095, USA.
144. University of Texas MD Anderson Cancer Center, Houston, TX 77030, USA.
145. Department of Genetics and Informatics Institute, University of Alabama at Birmingham, Birmingham, AL 35294, USA.
146. Department of Genetics, Department of Medicine, Washington University in St Louis, St Louis, MO 63110, USA.
147. Centre for Genomic Regulation (CRG), The Barcelona Institute of Science and Technology, Barcelona 08003, Spain.
148. Department of Bioinformatics and Computational Biology, The University of Texas MD Anderson Cancer Center, Houston, TX 77030, USA.
149. Beijing Genomics Institute, Shenzhen 518083, China.
150. Department of Urologic Sciences, University of British Columbia, Vancouver, BC V5Z 1M9, Canada.
151. Vancouver Prostate Centre, Vancouver, BC V6H 3Z6, Canada.
152. Division of Life Science and Applied Genomics Center, Hong Kong University of Science and Technology, Clear Water Bay, Hong Kong, China.
153. Geneplus-Shenzhen, Shenzhen 518122, China.
154. School of Computer Science and Technology, Xi'an Jiaotong University, Xi'an 710048, China.
155. Biobyte solutions GmbH, Heidelberg 69126, Germany.
156. Division of Oncology, Washington University School of Medicine, St Louis, MO 63110, USA.
157. Institute of Medical Genetics and Applied Genomics, University of Tübingen, Tübingen 72074, Germany.

158. Indiana University, Bloomington, IN 47405, USA.
159. Simon Fraser University, Burnaby, BC V5A 1S6, Canada.
160. Department of Computer Science, University of Toronto, Toronto, ON M5S 1A8, Canada.
161. School of Electronic and Information Engineering, Xi'an Jiaotong University, Xi'an 710048, China.
162. Department of Genetics, Washington University School of Medicine, St Louis, MO 63110, USA.
163. Department of Mathematics, Washington University in St Louis, St Louis, MO 63130, USA.
164. Department of Molecular and Human Genetics, Baylor College of Medicine, Houston, TX 77030, USA.
165. Human Genome Sequencing Center, Baylor College of Medicine, Houston, TX 77030, USA.
166. The First Affiliated Hospital, Xi'an Jiaotong University, Xi'an 710049, China.
167. Department of Epidemiology and Biostatistics, Memorial Sloan Kettering Cancer Center, New York, NY 10065, USA.
168. The McDonnell Genome Institute at Washington University, Department of Genetics, Department of Medicine, Siteman Cancer Center, Washington University in St Louis, St Louis, MO 63108, USA.
169. Department of Genomic Medicine, The University of Texas MD Anderson Cancer Center, Houston, TX 77030, USA.
170. The Jackson Laboratory for Genomic Medicine, Farmington, CT 06032, USA.
171. Quantitative & Computational Biosciences Graduate Program, Baylor College of Medicine, Houston, TX 77030, USA.
172. Department of Medical Oncology, Dana-Farber Cancer Institute, Boston, MA 02115, USA.
173. Department of Mathematics, Aarhus University, Aarhus 8000, Denmark.
174. Technical University of Denmark, Lyngby 2800, Denmark.
175. University of Copenhagen, Copenhagen 2200, Denmark.
176. Department for BioMedical Research, University of Bern, Bern 3008, Switzerland.
177. Department of Medical Oncology, Inselspital, University Hospital and University of Bern, Bern 3010, Switzerland.
178. Graduate School for Cellular and Biomedical Sciences, University of Bern, Bern 3012, Switzerland.
179. Department of Genitourinary Medical Oncology - Research, Division of Cancer Medicine, The University of Texas MD Anderson Cancer Center, Houston, TX 77030, USA.
180. Faculty of Biosciences, Heidelberg University, Heidelberg 69120, Germany.
181. Korea Advanced Institute of Science and Technology, Daejeon 34141, South Korea.
182. Institute for Research in Biomedicine (IRB Barcelona), The Barcelona Institute of Science and Technology, Barcelona 8003, Spain.
183. Research Program on Biomedical Informatics, Universitat Pompeu Fabra, Barcelona 08002, Spain.
184. Science for Life Laboratory, Department of Cell and Molecular Biology, Uppsala University, Uppsala SE-75124, Sweden.
185. Queensland Centre for Medical Genomics, Institute for Molecular Bioscience, The University of Queensland, St Lucia, QLD 4072, Australia.

186. University of Milano Bicocca, Monza 20052, Italy.
187. Peter MacCallum Cancer Centre, Melbourne, VIC 3000, Australia.
188. Sir Peter MacCallum Department of Oncology, The University of Melbourne, Melbourne, VIC 3052, Australia.
189. Institut Hospital del Mar d'Investigacions Mèdiques (IMIM), Barcelona 08003, Spain.
190. Institute for Research in Biomedicine (IRB Barcelona), Barcelona 08028, Spain.
191. Center for Precision Health, School of Biomedical Informatics, University of Texas Health Science Center, Houston, TX 77030, USA.
192. The Donnelly Centre, University of Toronto, Toronto, ON M5S 3E1, Canada.
193. Health Data Science Unit, University Clinics, Heidelberg 69120, Germany.
194. Department for Biomedical Research, University of Bern, Bern 3008, Switzerland.
195. Research Core Center, National Cancer Centre Korea, Goyang-si 410-769, South Korea.
196. Institute of Computer Science, Polish Academy of Sciences, Warsawa 01-248, Poland.
197. ETH Zurich, Department of Biology, Wolfgang-Pauli-Strasse 27, 8093 Zürich, Switzerland.
198. Harvard University, Cambridge, MA 02138, USA.
199. Memorial Sloan Kettering Cancer Center, New York, NY 10065, USA.
200. Department of Molecular Biophysics and Biochemistry, New Haven, CT 06520, USA.
201. Program in Computational Biology and Bioinformatics, New Haven, CT 06520, USA.
202. Yale University, New Haven, CT 06520, USA.
203. Department of Information Technology, Ghent University, Ghent B-9000, Belgium.
204. Department of Plant Biotechnology and Bioinformatics, Ghent University, Ghent B-9000, Belgium.
205. Yale School of Medicine, Yale University, New Haven, CT 06520, USA.
206. Division of Hematology-Oncology, Samsung Medical Center, Sungkyunkwan University School of Medicine, Seoul, 06351, South Korea.
207. Samsung Advanced Institute for Health Sciences and Technology, Sungkyunkwan University School of Medicine, Seoul 06351, South Korea.
208. Cheonan Industry-Academic Collaboration Foundation, Sangmyung University, Cheonan 31066, South Korea.
209. Spanish National Cancer Research Centre, Madrid 28029, Spain.
210. Bern Center for Precision Medicine, University Hospital of Bern, University of Bern, Bern 3008, Switzerland.
211. Englander Institute for Precision Medicine, Weill Cornell Medicine and NewYork Presbyterian Hospital, New York, NY 10021, USA.
212. Pathology and Laboratory, Weill Cornell Medical College, New York, NY 10021, USA.
213. Vall d'Hebron Institute of Oncology: VHIO, Barcelona 08035, Spain.
214. National Centre for Biological Sciences, Tata Institute of Fundamental Research, Bangalore 560065, India.
215. cBio Center, Dana-Farber Cancer Institute, Harvard Medical School, Boston, MA 02115, USA.
216. Department of Cell Biology, Harvard Medical School, Boston, MA 02115, USA.
217. Department of Cancer Biology, Dana-Farber Cancer Institute, Boston, MA 02215, USA.
218. Peter MacCallum Cancer Centre and University of Melbourne, Melbourne, VIC 3000, Australia.
219. cBio Center, Dana-Farber Cancer Institute, Boston, MA 02215, USA.
220. CREST, Japan Science and Technology Agency, Tokyo 113-0033, Japan.

221. Department of Medical Science Mathematics, Medical Research Institute, Tokyo Medical and Dental University, Bunkyo-ku, Tokyo 113-8510, Japan.
222. Laboratory for Medical Science Mathematics, Department of Biological Sciences, Graduate School of Science, The University of Tokyo, Bunkyo-ku, Tokyo 113-0033, Japan.
223. Science for Life Laboratory, Department of Oncology-Pathology, Karolinska Institutet, Stockholm 17121, Sweden.
224. Department of Gene Technology, Tallinn University of Technology, Tallinn 12616, Estonia.
225. Genetics & Genome Biology Program, SickKids Research Institute, The Hospital for Sick Children, Toronto, ON M5G 1X8, Canada.
226. Department of Information Technology, Ghent University, Interuniversitair Micro-Electronica Centrum (IMEC), Ghent B-9000, Belgium.
227. Science for Life Laboratory, Department of Immunology, Genetics and Pathology, Uppsala University, Uppsala SE-75108, Sweden.
228. Oregon Health & Sciences University, Portland, OR 97239, USA.
229. Department of Medicine and Therapeutics, The Chinese University of Hong Kong, Shatin, NT, Hong Kong, China SAR.
230. Second Military Medical University, Shanghai 200433, China.
231. The University of Texas Health Science Center at Houston, Houston, TX 77030, USA.
232. Department of Biomedical Informatics, College of Medicine, The Ohio State University, Columbus, OH 43210, USA.
233. The Ohio State University Comprehensive Cancer Center (OSUCCC – James), Columbus, OH 43210, USA.
234. School of Biomedical Informatics, The University of Texas Health Science Center at Houston, Houston, TX 77030, USA.
235. Department of Biochemistry and Molecular Genetics, Feinberg School of Medicine, Northwestern University, Chicago, IL 60637, USA.
236. University of Glasgow, CRUK Beatson Institute for Cancer Research, Bearsden, Glasgow G61 1BD, UK.
237. Centre for Molecular Science Informatics, Department of Chemistry, University of Cambridge, Cambridge CB2 1EW, UK.
238. Department of Biomedical Informatics, Harvard Medical School, Boston, MA 02115, USA.
239. Ludwig Center, Harvard Medical School, Boston, MA 02115, USA.
240. UC Santa Cruz Genomics Institute, University of California Santa Cruz, Santa Cruz, CA 95064, USA.
241. Department of Medicine, Baylor College of Medicine, Houston, TX 77030, USA.
242. Computational and Systems Biology, Genome Institute of Singapore, Singapore 138672, Singapore.
243. School of Computing, National University of Singapore, Singapore 117417, Singapore.
244. The Azrieli Faculty of Medicine, Bar-Ilan University, Safed 13195, Israel.
245. National Cancer Centre Singapore, Singapore 169610, Singapore.
246. Peking University, Beijing 100871, China.
247. Department of Genetics, University of North Carolina at Chapel Hill, Chapel Hill, NC 27599, USA.
248. Lineberger Comprehensive Cancer Center, University of North Carolina at Chapel Hill, Chapel Hill, NC 27599, USA.

249. China National GeneBank-Shenzhen, Shenzhen 518083, China.
250. Berlin Institute for Medical Systems Biology, Max Delbrück Center for Molecular Medicine, Berlin 13125, Germany.
251. University College London, London WC1E 6BT, UK.
252. School of Life Sciences, Peking University, Beijing 100180, China.
253. Department of Pathology, The University of Melbourne, Melbourne, VIC 3052, Australia.
254. Genome Institute of Singapore, Singapore 138672, Singapore.
255. Department of Epidemiology, University of Alabama at Birmingham, Birmingham, AL 35294, USA.
256. HudsonAlpha Institute for Biotechnology, Huntsville, AL 35806, USA.
257. O'Neal Comprehensive Cancer Center, University of Alabama at Birmingham, Birmingham, AL 35294, USA.
258. Department of Biosciences and Nutrition, Karolinska Institutet, Stockholm 14183, Sweden.
259. Ludwig Center at Harvard, Boston, MA 02115, USA.
260. German Cancer Consortium (DKTK), Partner site Berlin.
261. Finsen Laboratory and Biotech Research & Innovation Centre (BRIC), University of Copenhagen, Kiel 24118, Germany.
262. Institute of Human Genetics, Ulm University and Ulm University Medical Center, Ulm 89081, Germany.
263. Computational & Systems Biology Program, Memorial Sloan Kettering Cancer Center, New York, NY 10065, USA.
264. Korea University, Seoul 02481, South Korea.
265. Division of Computational Genomics and Systems Genetics, German Cancer Research Center (DKFZ), Heidelberg 69120, Germany.
266. Cancer Science Institute of Singapore, National University of Singapore, Singapore 169609, Singapore.
267. Programme in Cancer & Stem Cell Biology, Duke-NUS Medical School, Singapore 169857, Singapore.
268. SingHealth, Duke-NUS Institute of Precision Medicine, National Heart Centre Singapore, Singapore 169609, Singapore.
269. Institute of Molecular and Cell Biology, Singapore 169609, Singapore.
270. Laboratory of Cancer Epigenome, Division of Medical Science, National Cancer Centre Singapore, Singapore 169610, Singapore.
271. BIOPIC, ICG and College of Life Sciences, Peking University, Beijing 100871, China.
272. Genome Science Division, Research Center for Advanced Science and Technology, The University of Tokyo, Tokyo 153-8904, Japan.
273. Center for Bioinformatics and Functional Genomics, Cedars-Sinai Medical Center, Los Angeles, CA 90048, USA.
274. Department of Biomedical Sciences, Cedars-Sinai Medical Center, Los Angeles, CA 90048, USA.
275. The Hebrew University Faculty of Medicine, Jerusalem 91120, Israel.
276. Bioinformatics Group, Department of Computer, University of Leipzig, Leipzig 04109, Germany, Leipzig 04109, Germany.
277. Interdisciplinary Center for Bioinformatics, University of Leipzig, Leipzig 04109, Germany.

278. German Cancer Consortium (DKTK), German Cancer Research Center (DKFZ), Heidelberg 69120, Germany.
279. Bioinformatics Group, Department of Computer Science, University of Leipzig, Leipzig 04109, Germany.
280. Computational Biology, Leibniz Institute on Aging - Fritz Lipmann Institute (FLI), Jena 07745, Germany.
281. Transcriptome Bioinformatics, LIFE Research Center for Civilization Diseases, University of Leipzig, Leipzig 04109, Germany.
282. Center for Epigenetics, Van Andel Research Institute, Grand Rapids, MI 49503, USA.
283. Institut d'Investigacions Biomèdiques August Pi i Sunyer (IDIBAPS), Barcelona 08036, Spain.
284. Research Center for Advanced Science and Technology, The University of Tokyo, Minato-ku, Tokyo 108-8639, Japan.
285. Cancer Epigenomics, German Cancer Research Center (DKFZ), Heidelberg 69120, Germany.
286. Van Andel Research Institute, Grand Rapids, MI 49503, USA.
287. Department of Zoology, Genetics and Physical Anthropology, Universidade de Santiago de Compostela, Santiago de Compostela 15706, Spain.
288. Centre for Research in Molecular Medicine and Chronic Diseases (CIMUS), Universidade de Santiago de Compostela, Santiago de Compostela 15706, Spain.
289. The Biomedical Research Centre (CINBIO), Universidade de Vigo, Vigo 36310, Spain.
290. Transmissible Cancer Group, Department of Veterinary Medicine, University of Cambridge, Cambridge CB3 0ES, UK.
291. Sir Peter MacCallum Department of Oncology, University of Melbourne, Melbourne, VIC 3052, Australia.
292. Johns Hopkins School of Medicine, Baltimore, MD 21205, USA.
293. University of Ottawa Faculty of Medicine, Department of Biochemistry, Microbiology and Immunology, Ottawa, ON K1H 8M5, Canada.
294. Cancer Research UK Cambridge Institute, University of Cambridge, Cambridge CB2 0RE, UK.
295. University of Cambridge, Cambridge CB2 1TN, UK.
296. Sidra Medicine, Doha 26999, Qatar.
297. Genome Integrity and Structural Biology Laboratory, National Institute of Environmental Health Sciences (NIEHS), Durham, NC 27709, USA.
298. Brandeis University, Waltham, MA 02254, USA.
299. New York Genome Center, New York, NY 10013, USA.
300. Weill Cornell Medicine, New York, NY 10065, USA.
301. Hopp Children's Cancer Center (KiTZ), Heidelberg 69120, Germany.
302. Pediatric Glioma Research Group, German Cancer Research Center (DKFZ), Heidelberg 69120, Germany.
303. Skolkovo Institute of Science and Technology, Moscow 121205, Russia.
304. A.A.Kharkevich Institute of Information Transmission Problems, Moscow 127051, Russia.
305. Dmitry Rogachev National Research Center of Pediatric Hematology, Oncology and Immunology, Moscow, 117997, Russia.
306. Integrative Bioinformatics Support Group, National Institute of Environmental Health Sciences (NIEHS), Durham, NC 27709, USA.

307. Center For Medical Innovation, Seoul National University Hospital, Seoul 03080, South Korea.
308. Department of Internal Medicine, Seoul National University Hospital, Seoul 03080, South Korea.
309. Division of Genetics and Genomics, Boston Children's Hospital and Harvard Medical School, Boston, MA 02115, USA.
310. School of Medicine/School of Mathematics and Statistics, University of St Andrews, St Andrews, Fife KY16 9SS, UK.
311. Department of Genetics and Computational Biology, QIMR Berghofer Medical Research Institute, Brisbane 4006, Australia.
312. Institute for Molecular Bioscience, University of Queensland, St Lucia, Brisbane, QLD 4072, Australia.
313. School of Molecular Biosciences and Center for Reproductive Biology, Washington State University, Pullman, WA 99164, USA.
314. Cancer Research Institute, Beth Israel Deaconess Medical Center, Boston, MA 02215, USA.
315. Ben May Department for Cancer Research, Department of Human Genetics, The University of Chicago, Chicago, IL 60637, USA.
316. Tri-institutional PhD program of computational biology and medicine, Weill Cornell Medicine, New York, NY 10065, USA.
317. Department of Cellular and Molecular Medicine and Department of Bioengineering and Moores Cancer Center, University of California San Diego, La Jolla, California 92093, USA.
318. Department of Cellular and Molecular Medicine and Department of Bioengineering and Moores Cancer Center, University of California, San Diego, La Jolla, California 92093, USA.
319. Centre for Computational Biology, Duke-NUS Medical School, Singapore 169857, Singapore.
320. Department of Computer Science, University of Helsinki, Helsinki 00014, Finland.
321. Institute of Biotechnology, University of Helsinki, Helsinki 00014, Finland.
322. Organismal and Evolutionary Biology Research Programme, University of Helsinki, Helsinki 00014, Finland.
323. Programme in Cancer & Stem Cell Biology, Centre for Computational Biology, Duke-NUS Medical School, Singapore 169857, Singapore.
324. Academic Department of Medical Genetics, University of Cambridge, Addenbrooke’s Hospital, Cambridge CB2 0QQ, UK.
325. MRC Cancer Unit, University of Cambridge, Cambridge CB2 0XZ, UK.
326. The University of Cambridge School of Clinical Medicine, Cambridge CB2 0SP, UK.
327. Department of Applied Mathematics and Theoretical Physics, Centre for Mathematical Sciences, University of Cambridge, Cambridge CB3 0WA, UK.
328. Department of Statistics, Columbia University, New York, NY 10027, USA.
329. Duke-NUS Medical School, Singapore 169857, Singapore.
330. Faculty of Medicine and Health Technology, Tampere University and Tays Cancer Center, Tampere University Hospital, Tampere FI-33014, Finland.
331. Institute for Computational Health Sciences and Department of Pediatrics, University of California, San Francisco, CA USA.
332. Division of Cancer Epidemiology and Genetics, National Cancer Institute, National Institutes of Health, Bethesda, MD 20892, USA.

333. Department of Biostatistics, Bloomberg School of Public Health, Johns Hopkins University, Baltimore, MD 21287, USA.
334. Department of Oncology, School of Medicine, Johns Hopkins University, Baltimore, MD 21230, USA.
335. Integrated Graduate Program in Physical and Engineering Biology, Yale University, New Haven, CT 06520, USA.
336. Department of Computational Biology, University of Lausanne, Lausanne 1015, Switzerland.
337. Department of Genetic Medicine and Development, University of Geneva Medical School, Geneva CH1211, Switzerland.
338. Swiss Institute of Bioinformatics, University of Geneva, Geneva CH1211, Switzerland.
339. Institute of Medical Genetics and Applied Genomics, University of Tübingen, Tübingen 72076, Germany.
340. Independent Consultant, Wellesley 02481, USA.
341. Centre for Cancer Genetic Epidemiology, Department of Oncology, University of Cambridge, Cambridge CB1 8RN, UK.
342. Centre for Cancer Genetic Epidemiology, Department of Public Health and Primary Care, University of Cambridge, Cambridge CB1 8RN, UK.
343. CIBER Epidemiología y Salud Pública (CIBERESP), Spain.
344. Research Group on Statistics, Econometrics and Health (GRECS), UdG, Barcelona 8041, Spain.
345. Oxford Nanopore Technologies, New York, NY 10013, USA.
346. Institute of Evolutionary Biology (UPF-CSIC), Department of Experimental and Health Sciences, Universitat Pompeu Fabra, Barcelona 08003, Spain.
347. Icahn School of Medicine at Mount Sinai, New York, NY 10029, USA.
348. Laboratory of Translational Genomics, Division of Cancer Epidemiology and Genetics, National Cancer Institute, National Institutes of Health, Bethesda, MD 20892, USA.
349. Institut Català de Paleontologia Miquel Crusafont, Universitat Autònoma de Barcelona, Barcelona 08193, Spain.
350. Applications Department, Oxford Nanopore Technologies, Oxford OX4 4DQ, UK.
351. Institut de Recerca Sant Joan de Déu; Institut de Biomedicina de la Universitat de Barcelona (IBUB) & Department of Genetics, Microbiology & Statistics, Faculty of Biology, University of Barcelona, Barcelona 08028, Spain.
352. Department of Ophthalmology and Ocular Genomics Institute, Massachusetts Eye and Ear, Harvard Medical School, Boston, MA 02114, USA.
353. Department of Medical and Clinical Genetics, Genome-Scale Biology Research Program, University of Helsinki, Helsinki 00100, Finland.
354. Big Data Institute, Li Ka Shing Centre, University of Oxford, Oxford OX3 7LF, UK.
355. Oxford NIHR Biomedical Research Centre, University of Oxford, Oxford OX4 2PG, UK.
356. School of Electronic Information and Communications, Huazhong University of Science and Technology, Wuhan, Hubei 430074, China.
357. Bioinformatics Unit, Spanish National Cancer Research Centre (CNIO), Madrid 28029, Spain.
358. Vector Institute, Toronto, ON M5G 0A3, Canada.
359. South Western Sydney Clinical School, Faculty of Medicine, University of NSW, Liverpool, NSW 2170, Australia.

360. The Kinghorn Cancer Centre, Cancer Division, Garvan Institute of Medical Research, University of NSW, Sydney, NSW 2010, Australia.
361. West of Scotland Pancreatic Unit, Glasgow Royal Infirmary, Glasgow G31 2ER, UK.
362. Wolfson Wohl Cancer Research Centre, Institute of Cancer Sciences, University of Glasgow, Bearsden, Glasgow G61 1QH, UK.
363. University of Melbourne Centre for Cancer Research, The University of Melbourne, Melbourne, VIC 3052, Australia.
364. MRC Human Genetics Unit, MRC IGMM, University of Edinburgh, Edinburgh EH4 2XU, UK.
365. Bioinformatics Group, Division of Molecular Biology, Department of Biology, Faculty of Science, University of Zagreb, Bioinformatics Group, Division of Molecular Biology, Department of Biology, Faculty of Science, University of Zagreb.
366. Department of Bioinformatics, Research Institute, National Cancer Center Japan, Tokyo 104-0045, Japan.
367. Departments of Pathology, Genomic Medicine, and Translational Molecular Pathology, The University of Texas MD Anderson Cancer Center, Houston, TX 77030, USA.
368. Oregon Health & Science University, Portland, OR 97239, USA.
369. University of Glasgow, Glasgow G61 1BD, UK.
370. MRC-University of Glasgow Centre for Virus Research, Glasgow G61 1QH, UK.
371. Wolfson Wohl Cancer Research Centre, Institute of Cancer Sciences, University of Glasgow, Bearsden G61 1QH, United Kingdom.
372. School of Computing Science, University of Glasgow, Glasgow G12 8RZ, UK.
373. Molecular and Medical Genetics, Oregon Health and Science University, Portland, OR 97201, USA.
374. Department of Surgery, University of Melbourne, Parkville VIC 3010, Australia.
375. The Murdoch Children's Research Institute, Royal Children’s Hospital, Parkville, VIC 3052, Australia.
376. Walter + Eliza Hall Institute, Parkville, VIC 3052, Australia.
377. University of Cologne, Cologne 50931, Germany.
378. The Edward S. Rogers Sr. Department of Electrical and Computer Engineering, University of Toronto, Toronto, ON M5S 3G4, Canada.
379. University of Ljubljana, Ljubljana 1000, Slovenia.
380. Research Institute, NorthShore University HealthSystem, Evanston, IL 60201, USA.
381. Department of Public Health Sciences, The University of Chicago, Chicago IL 60637.
382. Department of Statistics, University of California Santa Cruz, Santa Cruz, CA 95064, USA.
383. Cambridge University Hospitals NHS Foundation Trust, Cambridge CB2 0QQ, UK.
384. University of Toronto, Toronto, ON M5G 2M9, Canada.
385. Department of Computer Science, Carleton College, Northfield, MN 55057, USA.
386. Molecular and Medical Genetics, Oregon Health & Science University, Portland, OR 97239, USA.
387. Center for Psychiatric Genetics, NorthShore University HealthSystem, Evanston, IL 60201, USA.
388. Molecular and Medical Genetics, Knight Cancer Institute, Oregon Health & Science University, Portland, OR 97219, USA.
389. Argmix Consulting, North Vancouver BC V7M 2J5, Canada.

390. University of Toronto, Department of Computer Science, Toronto, ON M5S 2E4, Canada.
391. Department of Biostatistics, The University of Texas MD Anderson Cancer Center, Houston, TX 77030, USA.
392. Department of Biostatistics, University of North Carolina at Chapel Hill, Chapel Hill, NC 27599, USA.
393. The University of Texas MD Anderson Cancer Center, Houston, TX 77030, USA.
394. Howard Hughes Medical Institute, University of California Santa Cruz, Santa Cruz, CA 95065, USA.
395. Cancer Unit, MRC University of Cambridge, Cambridge CB2 0XZ, UK.
396. Department of Bioinformatics and Computational Biology and Department of Systems Biology, The University of Texas MD Anderson Cancer Center, Houston, TX 77030, USA.
397. Department of Bioinformatics and Computational Biology, Department of Systems Biology, The University of Texas MD Anderson Cancer Center, Houston, TX 77030, USA.
398. Department of Health Sciences, Faculty of Medical Sciences, Kyushu University, Fukuoka 812-8582, Japan.
399. Baylor College of Medicine, Houston, TX 77030, USA.
400. Department of Applied Mathematics and Statistics, Johns Hopkins University, Baltimore, MD 21218, USA.
401. Heinrich Pette Institute, Leibniz Institute for Experimental Virology, Hamburg 20251, Germany.
402. University Medical Center Hamburg-Eppendorf, Bioinformatics Core, Hamburg 20246, Germany.
403. Earlham Institute, Norwich NR4 7UZ, UK.
404. Norwich Medical School, University of East Anglia, Norwich NR4 7TJ, UK.
405. The Institute of Cancer Research, London SW7 3RP, UK.
406. University of East Anglia, Norwich NR4 7TJ, UK.
407. German Center for Infection Research (DZIF), Partner Site Hamburg-Borstel-Lübeck-Riems, Hamburg, Germany.
408. Division of Molecular Genetics, German Cancer Research Center (DKFZ), Heidelberg 69120, Germany.
409. Peter MacCallum Cancer Centre, Melbourne, Victoria 3000, Australia.
410. Sir Peter MacCallum Department of Oncology, The University of Melbourne, Melbourne VIC 3000, Australia.
411. QIMR Berghofer Medical Research Institute, Brisbane, QLD 4006, Australia.
412. Victorian Institute of Forensic Medicine, Southbank, Victoria 3006, Australia.
413. University of Pennsylvania, Philadelphia, PA 19104, USA.
414. Peter MacCallum Cancer Centre, Melbourne VIC 3000, Australia.
415. Centre for Cancer Research, The Westmead Institute for Medical Research, Sydney 2145, Australia.
416. Department of Gynaecological Oncology, Westmead Hospital, Sydney 2145, Australia.
417. Genetics and Molecular Pathology, SA Pathology, Adelaide, SA 5000, Australia.
418. Centre for Cancer Research, The Westmead Institute for Medical Research, The University of Sydney, Sydney, NSW 2145, Australia.
419. Department of Gynaecological Oncology, Westmead Hospital, Sydney, NSW 2006, Australia.
420. Garvan Institute of Medical Research, Darlinghurst, NSW 2010 Australia.

421. Centre for Cancer Research, The Westmead Institute for Medical Research, and Department of Gynaecological Oncology, Westmead Hospital, Sydney, NSW 2145, Australia.
422. The University of Sydney, Sydney, NSW 2006, Australia.
423. The Westmead Institute for Medical Research. The University of Sydney. The Department of Gynaecological Oncology, Westmead Hospital, Westmead, NSW 2145, Australia.
424. Department of Surgery, Pancreas Institute, University and Hospital Trust of Verona, Verona 37134, Italy.
425. Department of Surgery, Princess Alexandra Hospital, Woolloongabba QLD 4102, Australia.
426. Surgical Oncology Group, Diamantina Institute, The University of Queensland, Woolloongabba, Brisbane, QLD 4102, Australia.
427. Department of Diagnostics and Public Health, University and Hospital Trust of Verona, Verona 37134, Italy.
428. ARC-Net Centre for Applied Research on Cancer, University and Hospital Trust of Verona, Verona 37134, Italy.
429. Department of Anatomical Pathology, St Vincent's Hospital, Sydney NSW 2010, Australia.
430. School of Biological Sciences, The University of Auckland, Auckland 1010, New Zealand.
431. Department of Pathology and Diagnostics, University and Hospital Trust of Verona, Verona 37134, Italy.
432. Department of Medicine, Section of Endocrinology, University and Hospital Trust of Verona, Verona 37134, Italy.
433. Department of Pathology, Queen Elizabeth University Hospital, Glasgow G51 4TF, UK.
434. Wolfson Wohl Cancer Research Centre, Bearsden, Glasgow G61 1QH, UK.
435. University of Sydney, Sydney, NSW 2006, Australia.
436. Department of Medical Oncology, Beatson West of Scotland Cancer Centre, Glasgow G12 0YN, UK.
437. Academic Unit of Surgery, School of Medicine, College of Medical, Veterinary and Life Sciences, University of Glasgow, Glasgow Royal Infirmary, Glasgow G4 OSF, UK.
438. Tissue Pathology and Diagnostic Oncology, Royal Prince Alfred Hospital, Camperdown, NSW 2050, Australia.
439. Discipline of Surgery, Western Sydney University, Penrith NSW 2751, Australia.
440. Institute of Cancer Sciences, College of Medical Veterinary and Life Sciences, University of Glasgow, Glasgow G12 8QQ, UK.
441. School of Environmental & Life Sciences, University of Newcastle, Ourimbah, NSW 2258, Australia.
442. School of Surgery M507, University of Western Australia, Nedlands 6009, Australia.
443. Applied Tumor Genomics Research Program, Research Programs Unit, University of Helsinki, Helsinki 00290, Finland.
444. Olivia Newton-John Cancer Research Institute, La Trobe University, Heidelberg, Victoria 3084, Australia.
445. Melanoma Institute Australia, The University of Sydney, Wollstonecraft NSW 2065, Australia.
446. Children's Hospital at Westmead, The University of Sydney, Westmead, NSW 2145, Australia.
447. Melanoma Institute Australia, The University of Sydney, Sydney 2065, Australia.

448. Australian Institute of Tropical Health and Medicine, James Cook University, Douglas QLD 4814, Australia.
449. Bioplatforms Australia, North Ryde, NSW 2109, Australia.
450. Melanoma Institute Australia, Macquarie University, Wollstonecraft NSW, 2109, Australia.
451. Children's Medical Research Institute, Westmead, NSW 2145 Australia.
452. Melanoma Institute Australia, The University of Sydney, Wollstonecraft 2065, NSW, Australia.
453. Westmead Institute for Medical Research, University of Sydney, Westmead, NSW 2145 Australia.
454. Melanoma Institute Australia, The University of Sydney, Wollstonecraft, NSW 2065, Australia.
455. Centre for Cancer Research, The Westmead Millennium Institute for Medical Research, University of Sydney, Westmead Hospital, Westmead NSW 2145, Australia.
456. Centre for Cancer Research, Westmead Institute for Medical Research, Westmead, NSW 2145, Australia.
457. Discipline of Pathology, Sydney Medical School, The University of Sydney, Sydney 2065, Australia.
458. Royal Prince Alfred Hospital, Sydney, NSW 2050, Australia.
459. Bioplatforms Australia, North Ryde, NSW 2109 Australia.
460. School of Mathematics and Statistics, The University of Sydney, Sydney, NSW 2006 Australia.
461. Diagnostic Development, Ontario Institute for Cancer Research, Toronto, ON M5G 0A3, Canada.
462. Ontario Tumour Bank, Ontario Institute for Cancer Research, Toronto, ON M5G 0A3, Canada.
463. PanCuRx Translational Research Initiative, Ontario Institute for Cancer Research, Toronto, ON M5G 0A3, Canada.
464. BioSpecimen Sciences Program, University Health Network, Toronto, ON M5G 2C4, Canada, Toronto, ON M5G 2C4, Canada.
465. Hepatobiliary/Pancreatic Surgical Oncology Program, University Health Network, Toronto, ON M5G 2C4, Canada.
466. Hepatobiliary/pancreatic Surgical Oncology Program, University Health Network, Toronto, ON M5G 2C4, Canada.
467. Lunenfeld-Tanenbaum Research Institute, Mount Sinai Hospital, Toronto, ON M5G 1X5, Canada.
468. Genomics, Ontario Institute for Cancer Research, Toronto, ON M5G 0A3, Canada.
469. Division of Medical Oncology, Princess Margaret Cancer Centre, Toronto, ON M5G 2M9, Canada.
470. Lunenfeld-Tanenbaum Research Institute, Toronto, ON M5G 1X5, Canada.
471. University of Nebraska Medical Centre, Omaha, NE 68198, USA.
472. BioSpecimen Sciences Program, University Health Network, Toronto, ON M5G 2C4, Canada.
473. Transformative Pathology, Ontario Institute for Cancer Research, Toronto, ON M5G 0A3, Canada.
474. Department of Biochemistry and Molecular Medicine, University California at Davis, Sacramento, CA 95817 USA.

475. University Health Network, Princess Margaret Cancer Centre, Toronto, ON M5G 1L7.
476. Department of Health Sciences Research, Mayo Clinic, Rochester, MN 55905, USA.
477. Department of Pathology, Human Oncology and Pathogenesis Program, Memorial Sloan Kettering Cancer Center, New York, NY 10053, USA.
478. Department of Laboratory Medicine and Pathobiology, University of Toronto, Toronto, ON M5S 1A8, Canada.
479. BioSpecimen Sciences, Laboratory Medicine (Toronto), Medical Biophysics, PanCuRX, Toronto, ON M5S 1A8, Canada.
480. Department of Medical Biophysics, University of Toronto, Toronto, ON M5G 1L7, Canada.
481. CRUK Manchester Institute and Centre, Manchester M204GJ, UK.
482. Department of Radiation Oncology, University of Toronto, Toronto, ON M5S 1A8, Canada.
483. Manchester Cancer Research Centre, Cancer Division, FBMH, University of Manchester, Manchester M204GJ, UK.
484. Radiation Medicine Program, Princess Margaret Cancer Centre, Toronto, ON M5G 2M9, Canada.
485. Department of Surgical Oncology, Princess Margaret Cancer Centre, Toronto, ON M5G 2M9, Canada.
486. Genome Informatics Program, Ontario Institute for Cancer Research, Toronto, ON M5G 2C4, Canada.
487. STTARR Innovation Facility, Princess Margaret Cancer Centre, Toronto, ON M5G 1L7, Canada.
488. Department of Pathology, Toronto General Hospital, Toronto, ON M5G 2C4, Canada.
489. Hefei University of Technology, Anhui 230009, China.
490. State key Laboratory of Cancer Biology, and Xijing Hospital of Digestive Diseases, Fourth Military Medical University, Shaanxi 710032, China.
491. Fourth Military Medical University, Shaanxi 710032, China.
492. Peking University Cancer Hospital & Institute, Beijing 100142, China.
493. Department of Surgery, Ruijin Hospital, Shanghai Jiaotong University School of Medicine, Shanghai, 200025, China.
494. Leeds Institute of Medical Research @ St James's, University of Leeds, St James's University Hospital, Leeds LS9 7TF, UK.
495. Canadian Center for Computional Genomics, McGill University, Montreal, QC H3A 0G1, Canada.
496. Department of Human Genetics, McGill University, Montreal, QC H3A 1B1, Canada.
497. International Agency for Research on Cancer, Lyon 69008, France.
498. McGill University and Genome Quebec Innovation Centre, Montreal, QC H3A 0G1, Canada.
499. Centre National de Génotypage, CEA - Institute de Génomique, Evry 91000, France.
500. Leeds Institute of Medical Research @ St James's, University of Leeds, St James's University Hospital, Leeds LS9 7TF, UK.
501. Institute of Mathematics and Computer Science, University of Latvia, Riga LV1459, Latvia.
502. Department of Oncology, Gil Medical Center, Gachon University, Incheon, South Korea.
503. Department of Molecular Oncology, BC Cancer Agency, Vancouver, BC V5Z 1L3, Canada.

504. Los Alamos National Laboratory, Los Alamos, NM 87545, USA.
505. Department of Genetics, Institute for Cancer Research, Oslo University Hospital, The Norwegian Radium Hospital, Oslo O310, Norway.
506. Lund University, Lund 223 62, Sweden.
507. Translational Research Lab, Centre Léon Bérard, Lyon 69373, France.
508. Radboud University, Department of Molecular Biology, Faculty of Science, Nijmegen Centre for Molecular Life Sciences, Nijmegen 6500 HB, The Netherlands.
509. Department of Pathology, Brigham and Women's Hospital, Harvard Medical School, Boston, MA 02115, USA.
510. Department Experimental Therapy, The Netherlands Cancer Institute, Amsterdam 1066 CX, The Netherlands.
511. Cancer Research UK Cambridge Institute, University of Cambridge, Li Ka Shing Centre, Cambridge CB2 0RE, UK.
512. Department of Oncology, University of Cambridge, Cambridge CB2 1TN, UK.
513. Breast Cancer Translational Research Laboratory JC Heuson, Institut Jules Bordet, Brussels 1000, Belgium.
514. Translational Cancer Research Unit, Center for Oncological Research, Faculty of Medicine and Health Sciences, University of Antwerp, Antwerp 2000, Belgium.
515. Department of Gynecology & Obstetrics, Department of Clinical Sciences, Skåne University Hospital, Lund University, Lund SE-221 85, Sweden.
516. Icelandic Cancer Registry, Icelandic Cancer Society, Reykjavik 125, Iceland.
517. Translational Cancer Research Unit, GZA Hospitals St.-Augustinus, Antwerp 2000, Belgium.
518. Department of Pathology, Memorial Sloan Kettering Cancer Center, New York, NY 10065, USA.
519. Department of Medical Oncology, Josephine Nefkens Institute and Cancer Genomics Centre, Erasmus Medical Center, Rotterdam 3015CN, The Netherlands.
520. National Genotyping Center, Institute of Biomedical Sciences, Academia Sinica, Taipei 115, Taiwan.
521. Department of Pathology, Oslo University Hospital Ulleval, Oslo 0450, Norway.
522. Faculty of Medicine and Institute of Clinical Medicine, University of Oslo, Oslo NO-0316, Norway.
523. Department of Pathology, Skåne University Hospital, Lund University, Lund SE-221 85, Sweden.
524. Department of Pathology, Academic Medical Center, Amsterdam 1105 AZ, The Netherlands.
525. Department of Pathology, College of Medicine, Hanyang University, Seoul 133-791, South Korea.
526. Department of Pathology, Asan Medical Center, College of Medicine, Ulsan University, Songpa-gu, Seoul 05505, South Korea.
527. Netherlands Cancer Institute, Lund University, Lund 223 62, Sweden.
528. Department of Surgery, Memorial Sloan-Kettering Cancer Center, New York, NY 10065, USA.
529. Department of Surgery, Brigham and Women’s Hospital/Dana Farber Cancer Insititute, Boston, MA 02115, USA.
530. Morgan Welch Inflammatory Breast Cancer Research Program and Clinic, The University of Texas MD Anderson Cancer Center, Houston, TX 77030, USA.

531. The University of Queensland Centre for Clinical Research, The Royal Brisbane & Women's Hospital, Herston, QLD 4029, Australia.
532. Department of Pathology, Jules Bordet Institute, Brussels 1000, Belgium.
533. Institute for Bioengineering and Biopharmaceutical Research (IBBR), Hanyang University, Seoul, South Korea.
534. University of Oslo, Oslo 0316, Norway.
535. Institut Bergonié, Bordeaux 33076, France.
536. Department of Pathology, Erasmus Medical Center Rotterdam, Rotterdam 3015 GD, The Netherlands.
537. Department of Research Oncology, Guy’s Hospital, King’s Health Partners AHSC, King’s College London School of Medicine, London SE1 9RT, UK.
538. University Hospital of Minjoz, INSERM UMR 1098, Besançon 25000, France.
539. Cambridge Breast Unit, Addenbrooke’s Hospital, Cambridge University Hospital NHS Foundation Trust and NIHR Cambridge Biomedical Research Centre, Cambridge CB2 2QQ, UK.
540. East of Scotland Breast Service, Ninewells Hospital, Aberdeen AB25 2XF, UK.
541. Oncologie Sénologie, ICM Institut Régional du Cancer, Montpellier 34298, France.
542. Los Almos National Laboratory, Los Alamos, NM 87545, USA.
543. Department of Radiation Oncology, Radboud University Medical Centre, Nijmegen 6525 GA, The Netherlands.
544. University of Iceland, Reykjavik 101, Iceland.
545. Dundee Cancer Centre, Ninewells Hospital, Dundee DD2 1SY, UK.
546. Institut Curie, INSERM Unit 830, Paris 75248, France.
547. Department of Laboratory Medicine, Radboud University Nijmegen Medical Centre, Nijmegen GA 6525, The Netherlands.
548. Department of General Surgery, Singapore General Hospital, Outram Rd, Singapore 169608, Singapore.
549. Universite Lyon, INCa-Synergie, Centre Léon Bérard, Lyon 69008, France.
550. Giovanni Paolo II / I.R.C.C.S. Cancer Institute, Bari BA 70124, Italy.
551. Department of Biopathology, Centre Léon Bérard, Lyon 69008, France.
552. Université Claude Bernard Lyon 1, Villeurbanne 69100, France.
553. NCCS-VARI Translational Research Laboratory, National Cancer Centre Singapore, Singapore 169610, Singapore.
554. Division of Molecular Carcinogenesis, The Netherlands Cancer Institute, Amsterdam 1066 CX, The Netherlands.
555. Division of Molecular Carcinogenesis, The Netherlands Cancer Institute, Amsterdam, The Netherlands.
556. Institute of Human Genetics, Christian-Albrechts-University, Kiel 24118, Germany.
557. Institute of Human Genetics, Ulm University and Ulm University Medical Center of Ulm, Ulm 89081, Germany.
558. Institute of Human Genetics, University of Ulm and University Hospital of Ulm, Ulm 89081, Germany.
559. Hematopathology Section, Institute of Pathology, Christian-Albrechts-University, Kiel 24118, Germany.
560. Department of Human Genetics, Hannover Medical School, Hannover 30625, Germany.
561. Department of Pediatric Oncology, Hematology and Clinical Immunology, Heinrich-Heine-University, Düsseldorf 40225, Germany.

562. Department of Internal Medicine/Hematology, Friedrich-Ebert-Hospital, Neumünster 24534, Germany.
563. University Hospital Muenster - Pediatric Hematology and Oncology, Muenster 24534, Germany.
564. Department of Pediatrics, University Hospital Schleswig-Holstein, Kiel 24105, Germany.
565. Department of Medicine II, University of Würzburg, Würzburg, Germany.
566. Senckenberg Institute of Pathology, University of Frankfurt Medical School, Frankfurt 60596, Germany.
567. Institute of Pathology, Charité – University Medicine Berlin, Berlin 10117, Germany.
568. Department for Internal Medicine II, University Hospital Schleswig-Holstein, Kiel 24105, Germany.
569. Institute for Medical Informatics Statistics and Epidemiology, University of Leipzig, Leipzig 04109, Germany.
570. Department of Hematology and Oncology, Georg-Augusts-University of Göttingen, Göttingen 37073, Germany.
571. Institute of Cell Biology (Cancer Research), University of Duisburg-Essen, Essen D-45147, Germany.
572. MVZ Department of Oncology, PraxisClinic am Johannisplatz, Leipzig 04109, Germany.
573. Institute of Pathology, Ulm University and University Hospital of Ulm, Ulm 89081, Germany.
574. Department of Pathology, Robert-Bosch-Hospital, Stuttgart, Germany, Stuttgart 70376, Germany.
575. University Hospital Giessen, Pediatric Hematology and Oncology, Giessen 35392, Germany.
576. Institute of Clinical Molecular Biology, Christian-Albrechts-University, Kiel 24118, Germany.
577. Institute of Pathology, University of Wuerzburg, Wuerzburg 97070, Germany.
578. Department of General Internal Medicine, University Kiel, Kiel 24118, Germany.
579. Clinic for Hematology and Oncology, St.-Antonius-Hospital, Eschweiler D-52249, Germany.
580. Department for Internal Medicine III, University of Ulm and University Hospital of Ulm, Ulm 89081, Germany.
581. Neuroblastoma Genomics, German Cancer Research Center (DKFZ), Heidelberg 69120, Germany.
582. University of Düsseldorf, Düsseldorf 40225, Germany.
583. Department of Vertebrate Genomics/Otto Warburg Laboratory Gene Regulation and Systems Biology of Cancer, Max Planck Institute for Molecular Genetics, Berlin 14195, Germany.
584. St. Jude Children's Research Hospital, Memphis, TN 38105-3678, USA.
585. Heidelberg University Hospital, Heidelberg 69120, Germany.
586. Genomics and Proteomics Core Facility High Throughput Sequencing Unit, German Cancer Research Center (DKFZ), Heidelberg 69120, Germany.
587. Epigenomics and Cancer Risk Factors, German Cancer Research Center (DKFZ), Heidelberg 69120, Germany.
588. University Medical Center Hamburg-Eppendorf, Hamburg 20251, Germany.
589. Institute of Pathology, University Medical Center Hamburg-Eppendorf, Hamburg 20251, Germany.

590. Martini-Clinic, Prostate Cancer Center, University Medical Center Hamburg-Eppendorf, Hamburg 20095, Germany.
591. Division of Cancer Genome Research, German Cancer Research Center (DKFZ), Heidelberg 69120, Germany.
592. National Institute of Biomedical Genomics, Kalyani 741235, West Bengal, India.
593. Advanced Centre for Treatment Research & Education in Cancer, Tata Memorial Centre, Navi Mumbai, Maharashtra 410210, India.
594. Department of Pathology, General Hospital of Treviso, Department of Medicine, University of Padua, Italy, Treviso 31100, Italy.
595. Department of Medicine (DIMED), Surgical Pathology Unit, University of Padua, Padua 35121, Italy.
596. Division of Pathology and Clinical Laboratories, Department of Hepatobiliary and Pancreatic Oncology, Hepatobiliary and Pancreatic Surgery Division, National Cancer Center Hospital, Chuo-ku, Tokyo, 104-0045, Japan.
597. Division of Cancer Genomics, Department of Bioinformatics, National Cancer Center, Tokyo 104-0045, Japan.
598. Department of Pathology, Keio University School of Medicine, Tokyo 160-8582, Japan.
599. Department of Hepatobiliary and Pancreatic Oncology, National Cancer Center Hospital (NCCH), Tokyo, 104-0045 Japan.
600. Department of Pathology, Graduate School of Medicine, The University of Tokyo, Bunkyo-ku, Tokyo 113-0033, Japan.
601. Preventive Medicine, Graduate School of Medicine, The University of Tokyo, Tokyo 113-0033, Japan, Tokyo 113-0033, Japan.
602. Gastric Surgery Division, Division of Pathology and Clinical Laboratories, National Cancer Center Hospital, Tokyo 104-0045, Japan.
603. Department of Preventive Medicine, Graduate School of Medicine, the University of Tokyo, Tokyo 113-0033, Japan.
604. Division of Cancer Genomics, Department of Bioinformatics, National Cancer Center Research Institute, National Cancer Center, Tokyo 104-0045, Japan.
605. Department of Gastroenterology and Hepatology, Yokohama City University Graduate School of Medicine, Kanagawa 236-0004, Japan.
606. Laboratory of Molecular Medicine, Human Genome Center, The Institute of Medical Science, University of Tokyo, Shirokane-dai, Minato-ku, Tokyo 108-8639, Japan.
607. Department of Cancer Genome Informatics, Graduate School of Medicine, Osaka University, Osaka 565-0871, Japan.
608. Hiroshima University, Hiroshima 734-8553, Japan.
609. Tokyo Women’s Medical University, Tokyo 162-8666, Japan.
610. Osaka International Cancer Center, Osaka 541-8567, Japan.
611. Wakayama Medical University, Wakayama 641-8509, Japan.
612. Hokkaido University, Sapporo 060-8648, Japan.
613. Division of Medical Oncology, National Cancer Centre, Singapore 169610, Singapore.
614. Cholangiocarcinoma Screening and Care Program and Liver Fluke and Cholangiocarcinoma Research Centre, Faculty of Medicine, Khon Kaen University, Khon Kaen, Thailand.
615. Lymphoma Genomic Translational Research Laboratory, National Cancer Centre, Singapore 169610, Singapore.

616. Center of Digestive Diseases and Liver Transplantation, Fundeni Clinical Institute, Bucharest 022328, Romania.
617. Division of Hepatobiliary and Pancreatic Surgery, Department of Surgery, School of Medicine, Keimyung University Dongsan Medical Center, Daegu 41931, South Korea.
618. Pathology, Hospital Clinic, Institut d'Investigacions Biomèdiques August Pi i Sunyer (IDIBAPS), Universiy of Barcelona, Barcelona 8034, Spain.
619. Anatomia Patológica, Hospital Clinic, Institut d'Investigacions Biomèdiques August Pi i Sunyer (IDIBAPS), Universiy of Barcelona, Barcelona 8036, Spain.
620. Spanish Ministry of Science and Innovation, Madrid 28046, Spain.
621. Hematology, Hospital Clinic, Institut d'Investigacions Biomèdiques August Pi i Sunyer (IDIBAPS), Universiy of Barcelona, Barcelona 8034, Spain.
622. Departamento de Bioquımicay Biologıa Molecular, Facultad de Medicina, Instituto Universitario de Oncologıa-IUOPA, Oviedo 33006, Spain.
623. Departamento de Bioquímica y Biología Molecular, Instituto Universitario de Oncología (IUOPA), Universidad de Oviedo, Oviedo 33006, Spain.
624. Royal National Orthopaedic Hospital - Bolsover, London W1W 5AQ, UK.
625. Department of Pathology, Oslo University Hospital, Oslo O310, Norway.
626. Department of Pathology, Oslo University Hospital, Norway and University of Oslo, Norway, Oslo O310, Norway.
627. Department of Pathology (Research), University College London Cancer Institute, London WC1E 6BT, UK.
628. Department for Clinical Science, University of Bergen, Bergen 5020, Norway.
629. Research Department of Pathology, University College London Cancer Institute, 72 Huntley Street, London, WC1E 6BT.
630. East Anglian Medical Genetics Service, Cambridge University Hospitals NHS Foundation Trust, Cambridge CB2 0QQ, UK.
631. Royal National Orthopaedic Hospital - Stanmore, Stanmore, Middlesex HA7 4LP, UK.
632. Division of Orthopaedic Surgery, Oslo University Hospital, Oslo 0379, Norway.
633. Radcliffe Department of Medicine, University of Oxford, Oxford OX3 9DU, UK.
634. University of Pavia, Pavia 27100, Italy.
635. Karolinska Institute, Stockholm SE-171 76, Sweden.
636. Wellcome Sanger Institute, Wellcome Genome Campus, Hinxton, Cambridge, CB10 1SD, UK.
637. University of Oxford, Oxford OX3 9DU, UK.
638. Salford Royal NHS Foundation Trust, Salford M6 8HD, UK.
639. Gloucester Royal Hospital, Gloucester, GL1 3NL, UK.
640. Royal Stoke University Hospital, Stoke-on-Trent ST4 6QG, UK.
641. St Thomas's Hospital, London SE1 7EH, UK.
642. Imperial College NHS Trust, Imperial College, London W2 INY, UK.
643. Department of Histopathology, Salford Royal NHS Foundation Trust, Salford M6 8HD, UK.
644. Faculty of Biology, Medicine and Health, University of Manchester, Salford M6 8HD, UK.
645. Edinburgh Royal Infirmary, Edinburgh EH16 4SA, UK.
646. Barking Havering and Redbridge University Hospitals NHS Trust, Romford, RM7 0AG, UK.

647. King's College London and Guy's and St Thomas' NHS Foundation Trust, London SE1 7EH, UK.
648. Cambridge Oesophagogastric Centre, Cambridge University Hospitals NHS Foundation Trust, Cambridge, CB2 0QQ.
649. Nottingham University Hospitals NHS Trust, Nottingham NG7 2UH, UK.
650. St Luke's Cancer Centre, Royal Surrey County Hospital NHS Foundation Trust, Guildford GU2 7XX, UK.
651. University of North Carolina at Chapel Hill, Chapel Hill, NC 27599, USA.
652. Norfolk and Norwich University Hospital NHS Trust, Norwich NR4 7UY, UK.
653. University Hospitals Coventry and Warwickshire NHS Trust, Coventry CV2 2DX, UK.
654. University Hospitals Birmingham NHS Foundation Trust, Birmingham B15 2GW, UK.
655. Centre for Cancer Research and Cell Biology, Queen's University, Belfast BT9 7AB, UK.
656. University Hospital Southampton NHS Foundation Trust, Southampton, SO16 6YD, UK.
657. Wythenshawe Hospital, Manchester M23 9LT, UK.
658. Royal Marsden NHS Foundation Trust, London and Sutton SW3 6JJ, UK.
659. Barts Cancer Institute, Barts and the London School of Medicine and Dentistry, Queen Mary University of London, London EC1M 6BQ, UK.
660. HCA Laboratories, London W1G 8AQ, UK.
661. University of Liverpool, Liverpool L69 3BX, UK.
662. Academic Urology Group, Department of Surgery, University of Cambridge, Cambridge CB2 0QQ, UK.
663. University of Oxford, Oxford, OX3 9DU, UK.
664. Department of Surgery and Cancer, Imperial College, London W2 INY, UK.
665. The Chinese University of Hong Kong, Shatin, Hong Kong, China.
666. Nuffield Department of Surgical Sciences, John Radcliffe Hospital, University of Oxford, Headington, Oxford OX3 9DU, UK.
667. Department of Histopathology, Cambridge University Hospitals NHS Foundation Trust, Cambridge CB2 0QQ, UK.
668. Department of Bioinformatics and Computational Biology / Department of Systems Biology, The University of Texas MD Anderson Cancer Center, Houston, TX 77030, USA.
669. Department of Pathology, The University of Texas MD Anderson Cancer Center, Houston, TX 77030, USA.
670. Canada's Michael Smith Genome Sciences Center, BC Cancer Agency, Vancouver, BC V5Z 4S6, Canada.
671. Center for Molecular Oncology, Memorial Sloan Kettering Cancer Center, New York, NY 10065, USA.
672. University Health Network, Toronto, ON M5G 2C4, Canada.
673. Department of Pathology and Laboratory Medicine, School of Medicine, University of North Carolina at Chapel Hill, Chapel Hill, NC 27599, USA.
674. Department of Population and Quantitative Health Sciences, Case Western Reserve University School of Medicine, Cleveland, OH 44016, USA.
675. Research Health Analytics and Informatics, University Hospitals Cleveland Medical Center, Cleveland, OH 44106, USA.
676. Arnie Charbonneau Cancer Institute, University of Calgary, Calgary, AB T2N 4N2, Canada.
677. Centre for Cancer Research and Cell Biology, Queens University, Belfast BT9 7AB, UK.

678. Sidney Kimmel Cancer Center, The Johns Hopkins Medical Institutions, Baltimore, MD 21230, USA.
679. Buck Institute for Research on Aging, Novato, CA 94945, USA.
680. Duke University Medical Center, Durham, NC 27710, USA.
681. Norris Comprehensive Cancer Center, University of Southern California, Los Angeles, CA 90033, USA.
682. The Preston Robert Tisch Brain Tumor Center, Duke University Medical Center, Durham, NC 27710, USA.
683. University of Southern California, USC/Norris Comprehensive Cancer Center, Los Angeles, CA 90033, USA.
684. Departments of Dermatology and Pathology, Yale University, New Haven, CT 06510, USA.
685. Fox Chase Cancer Center, Philadelphia, PA 19111, USA.
686. Johns Hopkins University, Baltimore, MD 21287, USA.
687. University of Michigan Comprehensive Cancer Center, Ann Arbor, MI 48109, USA.
688. University of Alabama at Birmingham, Birmingham, AL 35294, USA.
689. Division of Anatomic Pathology, Mayo Clinic, Rochester, MN 55905, USA.
690. Division of Experimental Pathology, Mayo Clinic, Rochester, MN 55905 USA.
691. The Sidney Kimmel Comprehensive Cancer Center at Johns Hopkins University, Baltimore, MD 21287, USA.
692. International Genomics Consortium, Phoenix, AZ 85004, USA.
693. Departments of Pediatrics and Genetics, University of North Carolina at Chapel Hill, Chapel Hill, NC 27599, USA.
694. Department of Pathology, UPMC Shadyside, Pittsburgh, PA 15232, USA.
695. Center for Cancer Genomics, National Cancer Institute, National Institutes of Health, Bethesda, MD 20892, USA.
696. Istituto Neurologico Besta, Department of Neuro-Oncology, Milano 20133, Italy.
697. University of Queensland Thoracic Research Centre, The Prince Charles Hospital, Brisbane, QLD 4032, Australia.
698. Department of Neurosurgery, University of Florida, Gainesville, FL 32610, USA.
699. Center for Biomedical Informatics, Harvard Medical School, Boston, MA 02115, USA.
700. Department of Cancer Biology, The University of Texas MD Anderson Cancer Center, Houston, TX 77030, USA.
701. Department of Surgical Oncology, The University of Texas MD Anderson Cancer Center, Houston, TX 77030, USA.
702. Division of Gastroenterology and Hepatology, Mayo Clinic, Rochester, MN 55905, USA.
703. University of Miami, Sylvester Comprehensive Cancer Center, Miami, FL 33136, USA.
704. Department of Internal Medicine, Division of Medical Oncology, University of North Carolina at Chapel Hill, Chapel Hill, NC 27599, USA.
705. Department of Internal Medicine, Division of Medical Oncology, Lineberger Comprehensive Cancer Center, University of North Carolina at Chapel Hill, Chapel Hill, NC 27599, USA.
706. University of Southern California, Norris Comprehensive Cancer Center, Los Angeles, CA 90033, USA.
707. The Sol Goldman Pancreatic Cancer Research Center, Department of Pathology, Johns Hopkins Hospital, Baltimore, MD 21287, USA.

708. Centre for Translational and Applied Genomics, British Columbia Cancer Agency, Vancouver, BC V5Z 1L3, Canada.
709. Department of Pathology & Immunology, Baylor College of Medicine, Houston, TX 770230, USA.
710. Michael E. DeBakey Veterans Affairs Medical Center, Houston, TX 770230, USA.
711. Carolina Center for Genome Sciences, University of North Carolina at Chapel Hill, Chapel Hill, NC 27599, USA.
712. Canada’s Michael Smith Genome Sciences Centre, BC Cancer, Vancouver, BC V5Z 4S6, Canada.
713. Indivumed GmbH, Hamburg 20251, Germany.
714. Division of Hepatobiliary and Pancreatic Surgery, Department of Surgery, School of Medicine, Keimyung University Dong-san Medical Center, Daegu 41931, South Korea.
715. Women's Cancer Program at the Samuel Oschin Comprehensive Cancer Institute, Cedars-Sinai Medical Center, Los Angeles, CA 90048, USA.
716. Canada’s Michael Smith Genome Sciences Centre, BC Cancer Agency, Vancouver, BC V5Z 4S6, Canada.
717. Department of Surgery, The George Washington University, School of Medicine and Health Science, Washington, DC 20052, USA.
718. Endocrine Oncology Branch, Center for Cancer Research, National Cancer Institute, National Institutes of Health, Bethesda, MD 20892, USA.
719. ILSbio, LLC Biobank, Chestertown, MD 21620, USA.
720. Gynecologic Oncology, NYU Laura and Isaac Perlmutter Cancer Center, New York University, New York, NY 10016, USA.
721. Division of Oncology, Stem Cell Biology Section, Washington University School of Medicine, St. Louis, MO 63110, USA.
722. Urologic Oncology Branch, Center for Cancer Research, National Cancer Institute, National Institutes of Health, Bethesda, MD 20892, USA.
723. Department of Systems Biology, University of Texas MD Anderson Cancer Center, Houston, TX 77030, USA.
724. Institute for Systems Biology, Seattle, WA 98109, USA.
725. Institute for Genomic Medicine, Nationwide Children's Hospital, Columbus, OH 43215, USA.
726. Department of Surgery, Duke University, Durham, NC 27710, USA.
727. Department of Obstetrics, Gynecology and Reproductive Services, University of California San Francisco, San Francisco, CA 94143, USA.
728. Departments of Neurology and Neurosurgery, Henry Ford Hospital, Detroit, MI 48202, USA.
729. Department of Pathology, Roswell Park Cancer Institute, Buffalo, NY 14263, USA.
730. Department of Obstetrics and Gynecology, Division of Gynecologic Oncology, Washington University School of Medicine, St. Louis, MO 3110, USA.
731. Penrose St. Francis Health Services, Colorado Springs, CO 80907, USA.
732. The University of Chicago, Chicago, IL 60637, USA.
733. Department of Neurology, Mayo Clinic, Rochester, MN 55905, USA.
734. Department of Genetics and Lineberger Comprehensive Cancer Center, University of North Carolina at Chapel Hill, Chapel Hill, NC 27599, USA.
735. NYU Langone Medical Center, New York, NY 10016, USA.

736. Department of Hematology and Medical Oncology, Cleveland Clinic, Cleveland, OH 44195, USA.
737. Department of Genetics, Department of Pathology and Laboratory Medicine, School of Medicine, University of North Carolina at Chapel Hill, Chapel Hill, NC 27599, USA.
738. Helen F. Graham Cancer Center at Christiana Care Health Systems, Newark, DE 19713, USA.
739. Cureline, Inc, South San Francisco, CA 94080, USA.
740. Department of Obstetrics and Gynecology, Medical College of Wisconsin, Milwaukee, WI 53226, USA.
741. Emory University, Atlanta, GA 30322, USA.
742. Vanderbilt University, Vanderbilt Ingram Cancer Center, Nashville, TN 37232, USA.
743. Ohio State University College of Medicine and Arthur G. James Comprehensive Cancer Center, Columbus, OH 43210, USA.
744. Research Computing Center, University of North Carolina at Chapel Hill, Chapel Hill, NC 27599, USA.
745. Analytical Biological Services, Inc, Wilmington, DE 19801, USA.
746. Department of Dermatology, University Hospital Essen, Westdeutsches Tumorzentrum & German Cancer Consortium, Essen 45122, Germany.
747. University of Pittsburgh, Pittsburgh, PA 15213, USA.
748. Murtha Cancer Center, Walter Reed National Military Medical Center, Bethesda, MD 20889, USA.
749. Brigham and Women's Hospital, Harvard Medical School, Boston, MA 02115, USA.
750. Department of Surgery, Memorial Sloan Kettering Cancer Center, New York, NY 10065, USA.
751. Department of Gynecologic Oncology & Reproductive Medicine, and Center for RNA Interference and Non-Coding RNA, the University of Texas MD Anderson Cancer Center, Houston, TX 77030, USA.
752. Department of Urology, Mayo Clinic, Rochester, MN 55905, USA.
753. Johns Hopkins Medical Institutions, Baltimore, MD 21205, USA.
754. Departments of Neurosurgery and Hematology and Medical Oncology, Winship Cancer Institute and School of Medicine, Emory University, Atlanta, GA 30322, USA.
755. Georgia Regents University Cancer Center, Augusta, GA 30912, USA.
756. Thoracic Oncology Laboratory, Mayo Clinic, Rochester, MN 55905, USA.
757. Institute for Genomic Medicine, Nationwide Children's Hospital, Columbus, OH 43205, USA.
758. Department of Obstetrics & Gynecology, Division of Gynecologic Oncology, Mayo Clinic, Rochester, MN 55905, USA.
759. International Institute for Molecular Oncology, Poznań 60-203, Poland.
760. Poznan University of Medical Sciences, Poznań 61-701, Poland.
