# Supplementary material for: Patterns of somatic structural variation in human cancer genomes
Source: Nature. 2020 Feb 5;578(7793):112–21. doi: 10.1038/s41586-019-1913-9 (PMC7025897; doi:10.1038/s41586-019-1913-9)
Supplement: Supplementary file 2 — Reporting Summary [file 41586_2019_1913_MOESM2_ESM.pdf]

nature research

Corresponding author(s): Peter J Campbell; Pameen Beroukhim

Last updated by author(s): Sep 10, 2019

Reporting Summary

Nature Research wishes to improve the reproducibility of the work that we publish. This form provides structure for consistency and transparency in reporting. For further information on Nature Research policies, see Authors & Referees and the Editorial Policy Checklist.

Statistics

For all statistical analyses, confirm that the following items are present in the figure legend, table legend, main text, or Methods section.

n/a	Confirmed	
☐	☒	The exact sample size (*n*) for each experimental group/condition, given as a discrete number and unit of measurement
☒	☐	A statement on whether measurements were taken from distinct samples or whether the same sample was measured repeatedly
☐	☒	The statistical test(s) used AND whether they are one- or two-sided<br>*Only common tests should be described solely by name; describe more complex techniques in the Methods section.*
☐	☒	A description of all covariates tested
☐	☒	A description of any assumptions or corrections, such as tests of normality and adjustment for multiple comparisons
☐	☒	A full description of the statistical parameters including central tendency (e.g. means) or other basic estimates (e.g. regression coefficient) AND variation (e.g. standard deviation) or associated estimates of uncertainty (e.g. confidence intervals)
☐	☒	For null hypothesis testing, the test statistic (e.g. *F*, *t*, *r*) with confidence intervals, effect sizes, degrees of freedom and *P* value noted<br>*Give P values as exact values whenever suitable.*
☐	☒	For Bayesian analysis, information on the choice of priors and Markov chain Monte Carlo settings
☒	☐	For hierarchical and complex designs, identification of the appropriate level for tests and full reporting of outcomes
☐	☒	Estimates of effect sizes (e.g. Cohen's *d*, Pearson's *r*), indicating how they were calculated

Our web collection on statistics for biologists contains articles on many of the points above.

Software and code

Policy information about availability of computer code

Data collection

Workflow Step Algorithm Version Dockstore Package*
WGS Alignment BWA-MEM v0.7.8-r455 https://goo.gl/oqp4Xd
EMBL/DKFZ SV caller DELLY v0.6.6 https://goo.gl/Y46MCo
EMBL/DKFZ SCNA caller ACEseq v1.0.189 https://goo.gl/4zoV42
EMBL/DKFZ SNV caller DKFZ somatic SNV workflow 1.0.132-1
EMBL/DKFZ indel caller Platypus v0.7.4
Sanger SCNA caller ascatNgs v1.5.2 https://goo.gl/9DSrbA
Sanger SV caller BRASS v4.012
grass v1.1.6
Sanger SNV caller CaVEMan v1.50
Sanger indel caller Pindel v1.5.7
Broad SCNA caller ABSOLUTE/JaBbA v1.5/? https://goo.gl/YkdtDt
Broad SV caller SvABA/dRanger/BreakPointer 2015-05-20/2016-03-13/2015-12-22
Broad SNV caller MuTect v1.1.4
Broad indel caller SvABA 2015-05-20
MuSE SNV caller MuSE v1.0rc https://goo.gl/5SR4bF
SMuFIN indel caller SMuFIN 2014-10-26 https://goo.gl/EuUP5k
Oxidative artefact filter OxoG 2016-4- 28 https://goo.gl/cUKP9K
SNV/Indel annotation VAGrENT v2.1.2 https://goo.gl/9DSrbA
ANNOVAR v2014Nov12 https://goo.gl/4zoV42
miniBAM generation VariantBAM v2017Dec12 https://goo.gl/S8h8e5
SNV/Indel merging and consensus generation SNV-MERGE v2017May26 https://goo.gl/TETSB8
SV merging and consensus generation SV-MERGE v2017Dec12 https://goo.gl/A9CEup
Strand bias filter DKFZ Strand Bias Filter v2016Dec15 https://goo.gl/8jXrvZ

Data analysis	We have described the algorithms in detail throughout the manuscript. Signatures analysis used the published NMF algorithm (PMID: 23318258) and an unpublished Bayesian method, released on github (https://github.com/nicolaroberts/hdp).

For manuscripts utilizing custom algorithms or software that are central to the research but not yet described in published literature, software must be made available to editors/reviewers. We strongly encourage code deposition in a community repository (e.g. GitHub). See the Nature Research guidelines for submitting code & software for further information.

Data

Policy information about availability of data

All manuscripts must include a data availability statement. This statement should provide the following information, where applicable:

- Accession codes, unique identifiers, or web links for publicly available datasets
- A list of figures that have associated raw data
- A description of any restrictions on data availability

The PCAWG-generated alignments, variant calls, annotations and derived data sets are available for general research use for browsing and download at http://dcc.icgc.org/pcawg/. In accordance with the data access policies of the ICGC and TCGA projects, most molecular, clinical and specimen data are in an open tier which does not require access approval. To access potentially identifying information, such as germline alleles and underlying read data, researchers will need to apply to the TCGA Data Access Committee (DAC) via dbGaP (https://dbgap.ncbi.nlm.nih.gov/aa/wga.cgi?page=login) for access to the TCGA portion of the data set, and to the ICGC Data Access Compliance Office (DACO; http://icgc.org/daco) for the ICGC portion.

Field-specific reporting

Please select the one below that is the best fit for your research. If you are not sure, read the appropriate sections before making your selection.

☒ Life sciences ☐ Behavioural & social sciences ☐ Ecological, evolutionary & environmental sciences

For a reference copy of the document with all sections, see nature.com/documents/nr-reporting-summary-flat.pdf

Life sciences study design

All studies must disclose on these points even when the disclosure is negative.

Sample size	We compiled an inventory of matched tumour/normal whole cancer genomes in the ICGC Data Coordinating Centre. Most samples came from treatment-naïve, primary cancers, but there were a small number of donors with multiple samples of primary, metastatic and/or recurrent tumours. Our inclusion criteria were: (i) matched tumour and normal specimen pair; (ii) a minimal set of clinical fields; and (iii) characterisation of tumour and normal whole genomes using Illumina HiSeq paired-end sequencing reads.<br>We collected genome data from 2,834 donors, representing all ICGC and TCGA donors that met these criteria at the time of the final data freeze in autumn 2014.<br><br>No formal power calculations were performed to decide sample size for PCAWG - we aggregated all genomes available at the time.
Data exclusions	After quality assurance, data from 176 donors were excluded as unusable. Reasons for data exclusions included inadequate coverage, extreme bias in coverage across the genome, evidence for contamination in samples and excessive sequencing errors (for example, through 8-oxoguanine). Data exclusion criteria were pre-established.
Replication	In order to evaluate the performance of each of the mutation-calling pipelines and determine an integration strategy, we performed a large-scale deep sequencing validation experiment. We selected a pilot set of 63 representative tumour/normal pairs, on which we ran the three core pipelines, together with a set of 10 additional somatic variant-calling pipelines contributed by members of the SNV Calling Working Group. To assess accuracy of SV calls, we therefore used the property that an SV must either generate a copy number change or be balanced, whereas artefactual calls will not respect this property. For individual SV callers, we estimated the true positive rate to be in the range 80-95% for samples in the pilot-63 dataset.
Randomization	Not applicable - this was a descriptive study.
Blinding	Not applicable - this was a descriptive study.

Reporting for specific materials, systems and methods

We require information from authors about some types of materials, experimental systems and methods used in many studies. Here, indicate whether each material, system or method listed is relevant to your study. If you are not sure if a list item applies to your research, read the appropriate section before selecting a response.

Materials & experimental systems

n/a	Involved in the study
☒	☐ Antibodies
☒	☐ Eukaryotic cell lines
☒	☐ Palaeontology
☒	☐ Animals and other organisms
☐	☒ Human research participants
☒	☐ Clinical data

Methods

n/a	Involved in the study
☒	☐ ChIP-seq
☒	☐ Flow cytometry
☒	☐ MRI-based neuroimaging

Human research participants

Policy information about studies involving human research participants

Population characteristics	Patient-by-patient clinical data are provided in the marker paper for the PCAWG consortium (Extended Data Table 1 of that manuscript). Demographically, the cohort included 1,469 males (55%) and 1,189 females (45%), with a mean age of 56 years (range, 1-90 years). Using population ancestry-differentiated single nucleotide polymorphisms (SNPs), the ancestry distribution was heavily weighted towards donors of European descent (77% of total) followed by East Asians (16%), as expected for large contributions from European, North American and Australian projects. We consolidated histopathology descriptions of the tumour samples, using the ICD-0-3 tumour site controlled vocabulary. Overall, the PCAWG data set comprises 38 distinct tumour types. While the most common tumour types are included in the dataset, their distribution does not match the relative population incidences, largely due to differences among contributing ICGC/TCGA groups in numbers sequenced.
Recruitment	Patients were recruited by the participating centres following local protocols. Samples obtained had to meet criteria on amount of tumour DNA available, meaning that the cohort is potentially somewhat biased towards larger tumours. Otherwise, we anticipate no major recruitment biases.
Ethics oversight	The Ethics oversight for the PCAWG protocol was undertaken by the TCGA Program Office and the Ethics and Governance Committee of the ICGC.

Note that full information on the approval of the study protocol must also be provided in the manuscript.
